# Multi-omics reveal the neuroprotective mechanisms of Xinshubao tablet against scopolamine-induced cognitive dysfunction in mice

**DOI:** 10.3389/fphar.2025.1596728

**Published:** 2025-07-04

**Authors:** Zhe Yang, Feng-Rong Zhang, Lu Ren, Jia-Ming Bai, Shi-Cong Wang, Xian-Yu Li, Hong-Jun Yang, Hong-He Xiao

**Affiliations:** ^1^ School of Pharmacy, Liaoning University of Traditional Chinese Medicine, Dalian, China; ^2^ Department of Oncology, Second Affiliated Hospital of Liaoning University of Traditional Chinese Medicine, Shenyang, China; ^3^ Beijing Key Laboratory of Traditional Chinese Medicine Basic Research on Prevention and Treatment for Major DisCMCeases, Experimental Research Center, China Academy of Chinese Medical Sciences, Beijing, China; ^4^ Mental Disorders Research Laboratory, Liaoning University of Traditional Chinese Medicine, Shenyang, China; ^5^ Fujian Pien Tze Huang Enterprise Key Laboratory of Natural Medicine Research and Development, Zhangzhou Pien Tze Huang Pharmaceutical Co., Ltd., Zhangzhou, China

**Keywords:** Alzheimer’s disease, Xinshubao tablet, neuroinflammation, 16s rDNA sequencing, metabolomics analysis, mRNA sequencing

## Abstract

**Introduction:**

Alzheimer’s disease (AD) is a progressive neurodegenerative disorder with limited treatments. Xinshubao tablet (XSB), a traditional Chinese medicine, contains several bioactive compounds with notable neuroprotective effects. Our previous studies have demonstrated that XSB can alleviate cognitive deficits in vascular dementia (VaD) models, suggesting its potential as a therapeutic candidate for AD.

**Methods:**

In this study, scopolamine-induced AD-like mice were orally administered with varying doses of XSB (0.13 g/kg, 0.26 g/kg and 0.52 g/kg) for 28 days. Behavior tests, H&E, Nissl, immunofluorescence staining, and Western blot assays were performed to evaluate the neuroprotection of XSB on AD-like mice. Then, fecal 16S rDNA sequencing, serum metabolomics, and hippocampal mRNA sequencing (mRNA-seq) analysis were performed to investigate the underlying mechanisms.

**Results and discussion:**

The results revealed that oral administration of XSB improved cognitive function, mitigated neuropathological damage, and alleviated dysfunction in the cholinergic system in AD-like mice. XSB treatment also enhanced gut microbiota diversity, increased the abundance of *Enterococcus*, *Actinobacteriota*, *Coriobacteriales*, and *Eggerthellaceae*, but reduced the abundance of *Helicobacter rodentium* and *Lachnospiraceae*. Integrating mRNA-seq and metabolomics data highlighted key regulatory pathways including the biosynthesis of unsaturated fatty acids, tyrosine metabolism, and glycerophospholipid metabolism. Furthermore, XSB treatment reduced the expression of TNF-α, IL-1β, MPO, enhanced SOD, GSH activities, reduced malondialdehyde (MDA) levels, upregulated the expression of BDNF, SYN, PSD95, and improved synaptic density. Transformation of XSB derived fecal microbiota (XSB-FM) effectively alleviated cognitive dysfunction and intestinal barrier injures. In conclusion, XSB may exert its neuroprotective effects via the microbiota-metabolite-brain axis, thereby improving neuroinflammation, neurotransmission, and synaptic integrity. These findings support the potential of XSB as a multifactorial therapeutic strategy for cognitive deficits in AD.

## Graphical Abstract

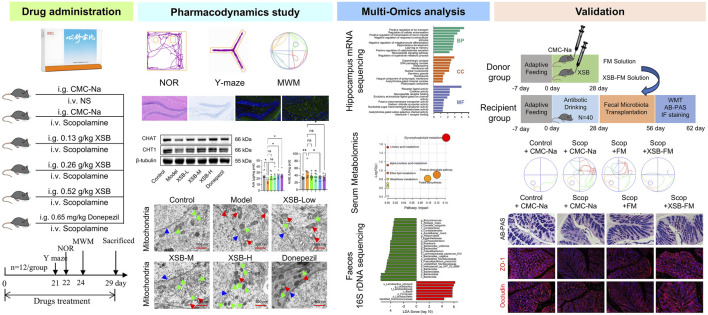

## 1 Introduction

Alzheimer’s disease (AD) is a complex neurodegenerative disorder characterized by multiple pathological features, including extracellular amyloid-beta (Aβ) plaques, neurofibrillary tangles (NFTs) composed of hyperphosphorylated tau protein, neuroinflammation, synaptic dysfunction, neuronal loss, glial activation, and impaired neurogenesis. Due to the multifactorial etiology of AD, involving genetic, environmental, and lifestyle factors, its pathogenesis remains poorly understood, and effective treatments are still lacking (Golde et al., 2018; Jia et al., 2020). Therefore, there is an urgent need for the development of novel therapeutic strategies to more effectively address AD.

Traditional Chinese medicine (TCM), with its unique multi-component and multi-target characteristics, has attracted growing attention for its potential in treating multifactorial diseases like AD. Recent studies suggest that TCM-based interventions may regulate multiple pathological processes simultaneously, making them well-suited for managing complex diseases and offering new hope for AD treatment. Xinshubao tablet (XSB), a marketed Chinese patent medicine, is composed of five botanical drugs. These include the air-dried radix and rhizoma of *Salvia miltiorrhiza* Bunge. (Danshen in Chinese), air-dried radix of *Paeonia lactiflora* Pall. (Baishao in Chinese), air-dried radix *and* rhizome *Eleutherococcus senticosus* (Rupr. & Maxim.) Maxim (Ciwujia in Chinese), air-dried radix of *Curcuma wenyujin* Y. H. Chen et C. Ling (Yujin in Chinese), and air-dried mature fructus of *Crataegus oxyacantha var. pinnatifida* (Bunge) Regel (Shanzha in Chinese). The botanical names of these plants have been verified using resources from Medicinal Plant Names Services (https://mpns.science.kew.org/). The chemical profile of XSB has been characterized in our pervious study (Xiao et al., 2024). Clinically, XSB is primarily prescribed for the treatment of coronary heart disease, angina pectoris, chest tightness due to qi deficiency and blood stasis, as well as hypertension, hyperlipidemia, and atherosclerosis.

Numerous studies have demonstrated that the active components of XSB, including Tanshinones (Dong et al., 2017), Salvianolic acids (Liu et al., 2020), Eleutherosides (Huang et al., 2013), and flavonoids from Crataegus (Lee et al., 2019), exhibit significant neuroprotective effects. For example, Tanshinone IIA, a key metabolite of Danshen, has been shown to reduce amyloid deposition and neuroinflammation in APP/PS1 mice (Liu et al., 2024), as well as inhibit neuronal apoptosis induced by ischemia (Chien et al., 2016). Salvianolic Acid B has been demonstrated to rescue cognitive impairment by inhibiting neuroinflammation and decreasing Aβ level in mice (Liu et al., 2020). Eleutheroside E modulates PKA signaling and gut microbiota to alleviate cognitive impairment induced by radiation (Song et al., 2022). The fruit of Crataegus pinnatifida has been reported to ameliorate memory deficits in an AD mouse model induced by intracerebroventricular injection of Aβ (Lee et al., 2019). These neuroprotective effects are beneficial for cognitive recovery, but the efficacy of XSB in improving AD-related cognitive deficits remains unclear.

Our previous research has demonstrated that XSB can mitigate neuroinflammatory responses, alleviate white matter damage, promote hippocampal neurogenesis, and protect against mitochondrial dysfunction, leading to cognitive improvement in vascular dementia (VaD) mice (Xiao et al., 2024). Since AD and VaD are the two most common forms of dementia in clinical practice, often coexisting as mixed dementia. The multiple neuroprotective effects of XSB’ compounds, coupled with its potent therapeutic effects on VaD, prompted us to investigate its potential therapeutic benefits in AD and to explore its underlying mechanisms.

In the present study, a scopolamine-induced amnesia model was established through intraperitoneal injection. Behavioral assessments and histopathological analyses were then employed to evaluate the anti-AD effects of XSB. To further elucidate the underlying pharmacological mechanisms, an integrative multi-omics approach was adopted, encompassing fecal 16S rDNA amplicon sequencing, serum metabolomics, and hippocampal transcriptomics.

## 2 Materials and methods

### 2.1 Materials

Primary antibodies including myeloperoxidase (MPO, 22225-1-AP), tumor necrosis factor-alpha (TNF-α, 60291-1-Ig), interleukin-1 beta (IL-1β, 16806-1-AP), brain-derived neurotrophic factor (BDNF, 66292-1-Ig), PSD95 (20665-1-AP), synaptophysin (SYN, 67864-1-Ig), choline acetyltransferase (CHAT, 20747-1-AP), choline transporter 1(CHT1, 21848-1-AP), zonula occludens-1 (ZO-1, 66452-1-Ig), Occludin (66378-1-Ig) and β-tubulin (80713-1-RR) were purchased from Proteintech Biotechnology Co., Ltd. (Wuhan, China). Commercial kits including total superoxide dismutase (SOD, A001-3-2), malonaldehyde (MDA, A003-1-2), reduced glutathione (GSH, A006-2-1), acetyl choline (ACh, A105-1-1), acetylcholinesterase (AChE, BC2025-100T/48S) were purchased from Nanjing Jiancheng Biotechnology Co., LTD. (Nanjing, China). AB-PAS staining assay (G1285) was purchased form Beijing Solarbio Science & Technology Co.,Ltd. Scopolamine (Scop, HY-N0296) was purchased from MedChemExpress LLC (China) (Shanghai, China). Xinshubao tablets (2011024) were obtained from Zhangzhou Pien Tze Huang Pharmaceutical Co., Ltd. (Zhangzhou, China). Donepezil hydrochloride tablets (2203026) were purchased from Eisai (China) Pharmaceutical Co., LTD.

### 2.2 Animals

Male C57BL/6J mice (6–8 weeks old) were procured from Beijing SPF Biotechnology Co., Ltd. (Certificate No.: SCXK (Beijing) 2019-0010). The animals were housed in a controlled environment with a temperature maintained at 23°C–25°C, relative humidity of 40%–60%, and a 12-h light/dark cycle. Standard laboratory chow and water were provided *ad libitum*. Following a week acclimatization period, the experimental procedures were initiated.

All the experiments were approved by the Ethical Committee of Experimental Animal Welfare of Experimental Research Center China Academy of Chinese Medicine Science (ERCCACMS21-2201-01), and conducted in accordance with the ARRIVE guidelines, and were carried out in accordance with Guide for the Care and Use of Laboratory Animals published by the US National Institutes of Health (NIH Publication No. 85-23, revised 1996).

### 2.3 Animal grouping and drug administration

The experiment was divided into 6 groups: Control group, Model group, XSB low-dose, medium-dose, and high-dose groups, and a Positive Control group, with 12 mice in each group. Mice in the Control group received intraperitoneal injections of normal saline, while all other groups were administered scopolamine hydrobromide solution (3 mg/kg) via intraperitoneal injection once daily for 4 consecutive weeks to induce cognitive impairment (Xu et al., 2019). Simultaneously, the XSB-treated groups were orally administered XSB at doses of 0.13 g/kg, 0.26 g/kg and 0.52 g/kg once daily (Xiao et al., 2024). The Control and Model groups were given an equivalent volume of CMC-Na via oral gavage, whereas the Positive control group received 0.65 mg/kg donepezil hydrochloride suspension in CMC-Na by oral gavage (Xiao et al., 2020). n = 12 mice in each group.

### 2.4 Behavioral assessment of cognitive function in mice

Commencing on the 21st day of drug administration, the cognitive functions of the mice were systematically assessed through a series of three sequential behavioral paradigms: the novel object recognition (NOR) test, the spontaneous alternation Y-maze test, and the Morris water maze (MWM) test.

Spontaneous alternation Y-maze test: The spontaneous alternation Y-maze test was conducted to evaluate working memory in mice. Each mouse was placed at the distal end of one arm and allowed to explore freely for 5 min. Behavioral trajectories were recorded using a video tracking system, and the spontaneous alternation rate was calculated with the ANY-maze software.

NOR test: Following the Y-maze test, each mouse was placed individually in a chamber for 5 min to adapt to the environment. After 24 h, two identical objects were introduced, and mice were allowed to explore freely for 5 min (training phase). One hour later, in the testing phase, one of the identical objects was replaced with a novel object of similar size but different shape. The exploration time for the novel object (T1) and familiar object (T2) was recorded using ANY-maze software, and the recognition index (RI) was calculated as RI = T1/(T1 + T2).

MWM test: MWM test was conducted to evaluate spatial learning and memory. During the spatial acquisition phase, a hidden platform was placed 1 cm below the water surface (20°C–21°C). Mice were trained once daily for 5 days, with trials initiated from random quadrants. If the platform was not located within 60 s, mice were guided to it and allowed to remain for 20 s. On the 6th day, the platform was removed, and a probe trial was performed. The escape latency, platform crossings, and time spent in the target quadrant were recorded to assess learning and memory.

### 2.5 Histologic examination

After the MWM test, 3 mice per group were randomly selected for histological analysis. Paraffin-embedded brain sections (4 μm) were prepared using a rotary microtome and stored at 4°C for histopathological analysis.

#### 2.5.1 H&E staining

Brain sections were deparaffinized, and stained with hematoxylin (60°C, 1 min). Sections were differentiated in 1% acid ethanol, counterstained with 0.5% eosin, dehydrated in graded ethanol, cleared in xylene, and mounted with neutral resin. Histopathological changes were examined under a light microscope.

#### 2.5.2 Nissl staining

Brain sections were deparaffinized, and stained with Nissl solution at 37°C for 10 min. After rinsing, sections were dehydrated in 95% ethanol, cleared in xylene, and mounted with neutral resin. Nissl bodies in the cortex, hippocampal CA1, CA3, and DG regions were imaged and quantified under a light microscope.

#### 2.5.3 Immunofluorescence staining

Brain sections or colon secions were deparaffinized, and permeabilized with 0.5% Triton X-100 for 30 min. After blocking with 5% BSA for 1 h, sections were incubated overnight at 4°C with primary antibodies, including CHAT, CHT1, TNF-α, IL-1β, BDNF, PSD95, SYN, ZO-1 and Occludin (all 1:100). They were then incubated with Alexa Fluor-488 or Cy3-conjugated secondary antibodies for 1.5 h in the dark. Nuclei were counterstained with DAPI and mounted using an anti-fluorescent quencher. Fluorescence images were captured using a fluorescence microscope.

### 2.6 Quantification of SOD, MDA, GSH, ACh and AChE levels in brain tissue

Following the completion of the MWM test, 8 mice from each group were sacrificed under deep anesthesia, and their brains were rapidly harvested. The superoxide dismutase (SOD), malondialdehyde (MDA), glutathione (GSH), acetylcholine (ACh) and acetylcholinesterase (AChE) levels in brain tissue were quantified following the manufacturer’s instructions.

### 2.7 Transmission electron microscopy (TEM) tests

Hippocampal tissues were collected from 3 mice per group. The hippocampal samples were rapidly dissected and fixed in 2% glutaraldehyde (pH 7.2). Tissue blocks from the CA1 region, measuring approximately 1.0 × 1.0 × 1.0 mm^3^, were further fixed in 1% osmium tetroxide at room temperature for 2 h. After sequential dehydration, infiltration, embedding, and polymerization, ultrathin sections (70 nm) were prepared. These sections were stained with 2% uranyl acetate in ethanol and 2.6% lead citrate. Finally, the sections were visualized and imaged using a TEM (HT7700, Hitachi, Tokyo, Japan). Quantitative analysis of mitochondrial density and synaptic numbers was performed using ImageJ software.

### 2.8 Western blot assays

Hippocampal total proteins were separated by SDS-PAGE and subsequently transferred onto PVDF membranes via wet transfer at 250 mA for 80 min. The membranes were blocked with 5% BSA at room temperature for 1 h, followed by incubation with primary antibodies against CHAT, CHT1, BDNF, SYN, PSD95, TNF-α, IL-1β, and MPO at 4°C overnight. After thorough washing, membranes were incubated with the corresponding HRP-conjugated secondary antibodies. The protein bands were visualized using a gel imaging system, and grayscale intensity was quantified using ImageJ software. Protein expression levels were normalized to internal controls (n = 3 in each group).

### 2.9 Gut microbiota 16S rDNA amplicon sequencing analysis

The detailed information was included in Supplementary Material. In short, fecal samples from 6 mice per group were aseptically collected. Amplicon sequence variants (ASVs) were identified through exact sequence variants, and taxonomic classification was performed against the SILVA 16S rDNA gene reference database. Alpha diversity indices, such as Chao1, Shannon, and Simpson, were calculated to assess species richness and diversity. Beta diversity was evaluated using weighted UniFrac distances. Differentially abundant taxa were identified using linear discriminant analysis effect size (LEfSe), with an LDA score threshold of 3.0. Spearman correlation analysis was performed to investigate the associations between differentially abundant taxa and cognitive function-related indicators in the behavior tests, including escape latency, platform crossing number, time spent in the target quadrant, and spontaneous alternation. A correlation coefficient threshold of |R| > 0.3 and a significance level of *P <* 0.05 were used to identify potential associations.

### 2.10 Serum metabolomics analysis

The detailed information was included in Supplementary Material. In short, serum samples from 6 mice per group were collected. Metabolomics profiling was conducted using liquid chromatography coupled with mass spectrometry (LC-MS). Chromatographic separation was performed on an ACQUITY UPLC system, with mass spectrometric detection carried out on a Q Exactive Plus Orbitrap mass spectrometer. Data acquisition was conducted in both positive and negative ion modes, and full-scan mass spectra were recorded across a specified m/z range. Differential metabolites (DMs) were determined based on the variable importance in projection (VIP) scores from the PLS-DA model, with a threshold of VIP >1.0. Pathway enrichment analysis was performed using KEGG database. Pearson correlation analysis was conducted to explore the relationships between DMs and cognitive function-related indicators. A correlation coefficient threshold of |R| > 0.5 and statistical significance of *P <* 0.05 were used to identify significant associations.

### 2.11 Hippocampal transcriptomic analysis

mRNA-seq was conducted to analyze transcriptomic variations in the hippocampal tissues. Total RNA was extracted using a commercial RNA extraction kit (RC101-01, Vazyme Biotech Co., Ltd., Nanjing, China). Gene expression levels were quantified by calculating the TPM value. Differentially expressed genes (DEGs) were identified using DESeq2 with an absolute log_2_ fold change (log_2_ FC) ≥ 1 and *P* value <0.05. Functional annotation of DEGs was conducted through Gene Ontology (GO) and KEGG pathway enrichment using DAVID database (https://davidbioinformatics.nih.gov/tools.jsp). The Volcano, Heatmap, GO and KEGG pathway enrichment plots were generated using the online tool available at https://www.bioinformatics.com.cn.

### 2.12 Fecal microbiota transplantation (FMT) experiments

#### 2.12.1 Experiments design

Donors: Thirty male C57BL/6J mice were randomly divided into two donor groups (n = 15/group). One group received a vehicle control (0.3% CMC-Na solution), and the other was administered XSB (0.52 mg/kg) via oral gavage once daily for 4 weeks. Fresh fecal samples were collected from each group to prepare standardized fecal microbiota suspensions: FM (from the vehicle-treated group) and XSB-FM (from the XSB-treated group). The samples were suspended in sterile PBS, homogenized using a vortex mixer, and centrifuged at 600 *g* for 3 min. The resulting supernatant was used to prepare a bacterial suspension at a concentration of 100 g/L.

Recipients: Forty recipient mice were administered a broad-spectrum antibiotic cocktail *ad libitum* for 4 weeks. Based on previous studies, the antibiotic mixture was prepared at the following concentrations: ampicillin (1 g/L), neomycin (1 g/L), metronidazole (1 g/L), and vancomycin (0.5 g/L) (Wang et al., 2021). Then, the 40 mice was randomized into 4 groups (n = 10/group): (1) Control + CMC-Na: Normal saline (i.p.) + CMC-Na 0.2 mL, oral gavage); (2) Scop + CMC-Na: Scopolamine (3 mg/kg, i.p.) + CMC-Na (0.2 mL, oral gavage); (3) Scop + FM: Scopolamine (3 mg/kg, i.p.) + FM (0.2 mL, oral gavage); (4) Scop + XSB-FM: Scopolamine (3 mg/kg, i.p.) + XSB-FM (0.2 mL, oral gavage). Each mouse received 0.2 mL of the corresponding treatment by oral gavage twice daily for 4 weeks. Cognitive function was assessed using the MWM test. Intestinal barrier integrity was evaluated via Alcian blue-periodic acid-Schiff (AB-PAS) staining, and colonic expression of the tight junction proteins ZO-1 and Occludin was determined by immunofluorescence.

#### 2.12.2 AB-PAS staining

Colon tissue sections were deparaffinized, rehydrated, and sequentially stained with Alcian blue, periodic acid, Schiff’s reagent, and hematoxylin. After dehydration and mounting, the sections were examined under a microscope. The colonic epithelium showed positive staining, with AB-PAS-positive cells appearing blue. n = 3 mice/group.

### 2.13 Statistical analysis

All data were presented as mean ± standard error of the mean (mean ± SEM). Statistical analyses were performed using GraphPad Prism 9.4 software. Escape latency data in the MWM test, collected across multiple trials, were analyzed using repeated-measures two-way ANOVA followed by Bonferroni post-hoc test. For comparisons among multiple groups, one-way ANOVA with Tukey’s post-hoc test was applied. A p-value of less than 0.05 was considered statistically significant. Fluorescence intensity quantification was conducted using ImageJ software.

## 3 Results

### 3.1 XSB improves cognitive function in model mice

**FIGURE 1:**
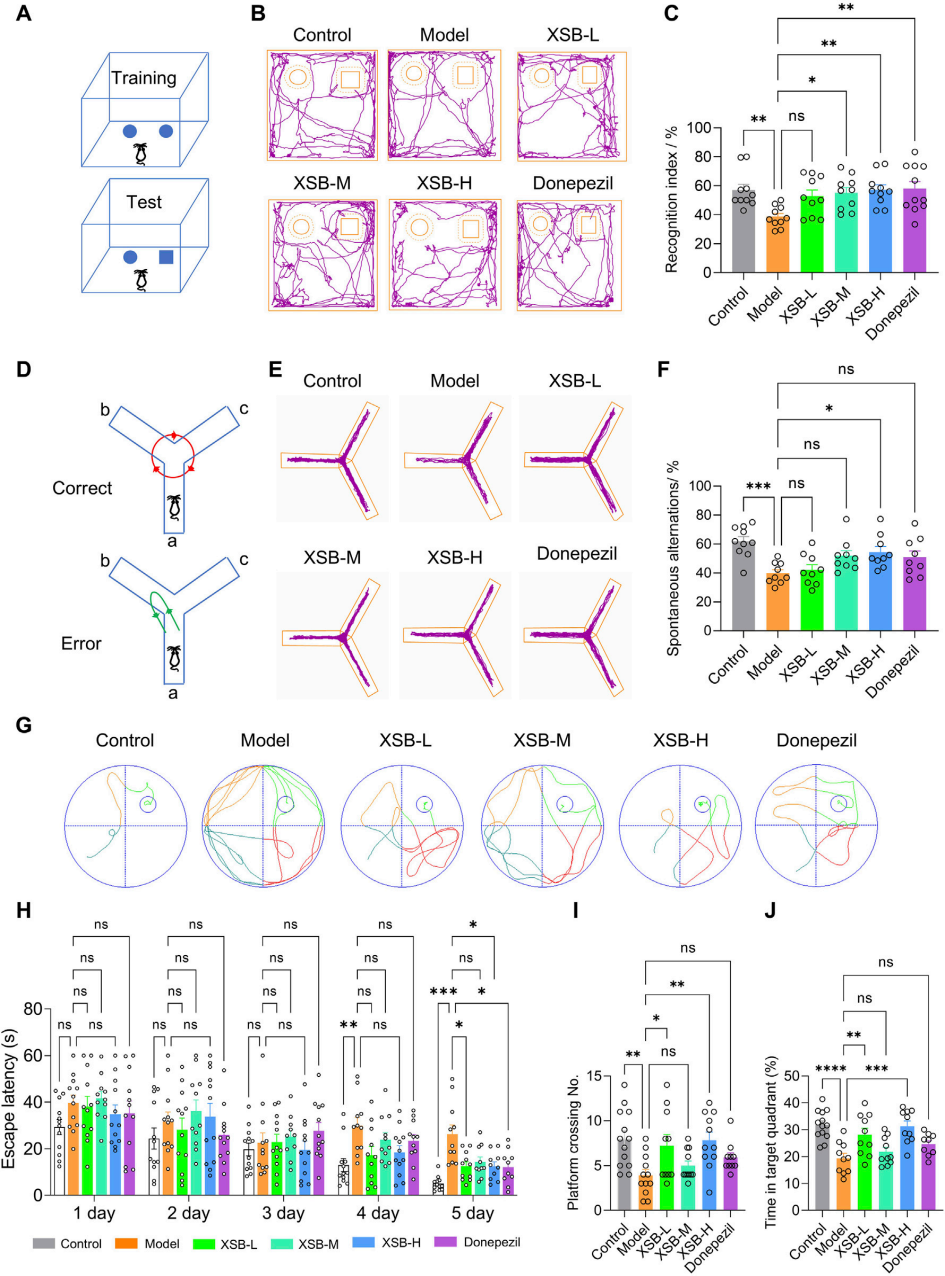
XSB improves cognitive function in AD model mice. **(A)**: Schematic diagram of the NOR test; **(B)**: Representative trajectory of mouse movement in the NOR test; **(C)**: Statistical graph of the recognition index in the NOR test for each group of mice; **(D)**: Schematic diagram of the Y-maze test; **(E)**: Representative trajectory of mouse movement in the Y-maze test; **(F)**: Statistical graph of spontaneous alternation counts in the Y-maze test; **(G)**: Typical swimming track diagrams of mice in the MWM test; **(H-J)**: Statistical graph of escape latency **(H)**, the number of platform crossings **(I)** and the percentage of time spent in the target quadrant **(J)**. Data are presented as mean ± SEM; *: P < 0.05, **: P < 0.01, ***: P < 0.001, ****: P < 0.0001, ns: *P >* 0.05. n = 12 mice/group.

The NOR test results showed treatment with XSB-M (Figures 1A–C, *P <*0.05 vs. Model group), and XSB-H (Figures 1A–C, *P <* 0.01 vs. Model group) significantly improved the recognition index, with effects similar to the positive control drug donepezil (Figures 1A–C *P <* 0.01 vs. Model group). Similarly, in the Y-maze test, XSB-H significantly improved spontaneous alternation behavior (Figures 1D–F, *P <* 0.05 vs. Model group). In the MWM test, the typical swimming track diagrams of mice were as shown in Figure 1G. XSB treatment decreased escape latency over time, test, XSB treatment decreased escape latency over time, with significant differences observed on days 4 and 5 compared to the Model group (Figure 1H, *P <* 0.001 on day 5). Additionally, in the spatial probe trial, XSB treatment increased platform crossings (Figure 1I, *P <* 0.01 vs. Model group) and time spent in the target quadrant compared (Figure 1J, *P <* 0.01 vs. Model group) to the Model group. These results suggest that XSB can enhance short-term memory, working memory, and spatial learning and memory in model mice.

### 3.2 XSB alleviates cortical and hippocampal structural damage in model mice

**FIGURE 2:**
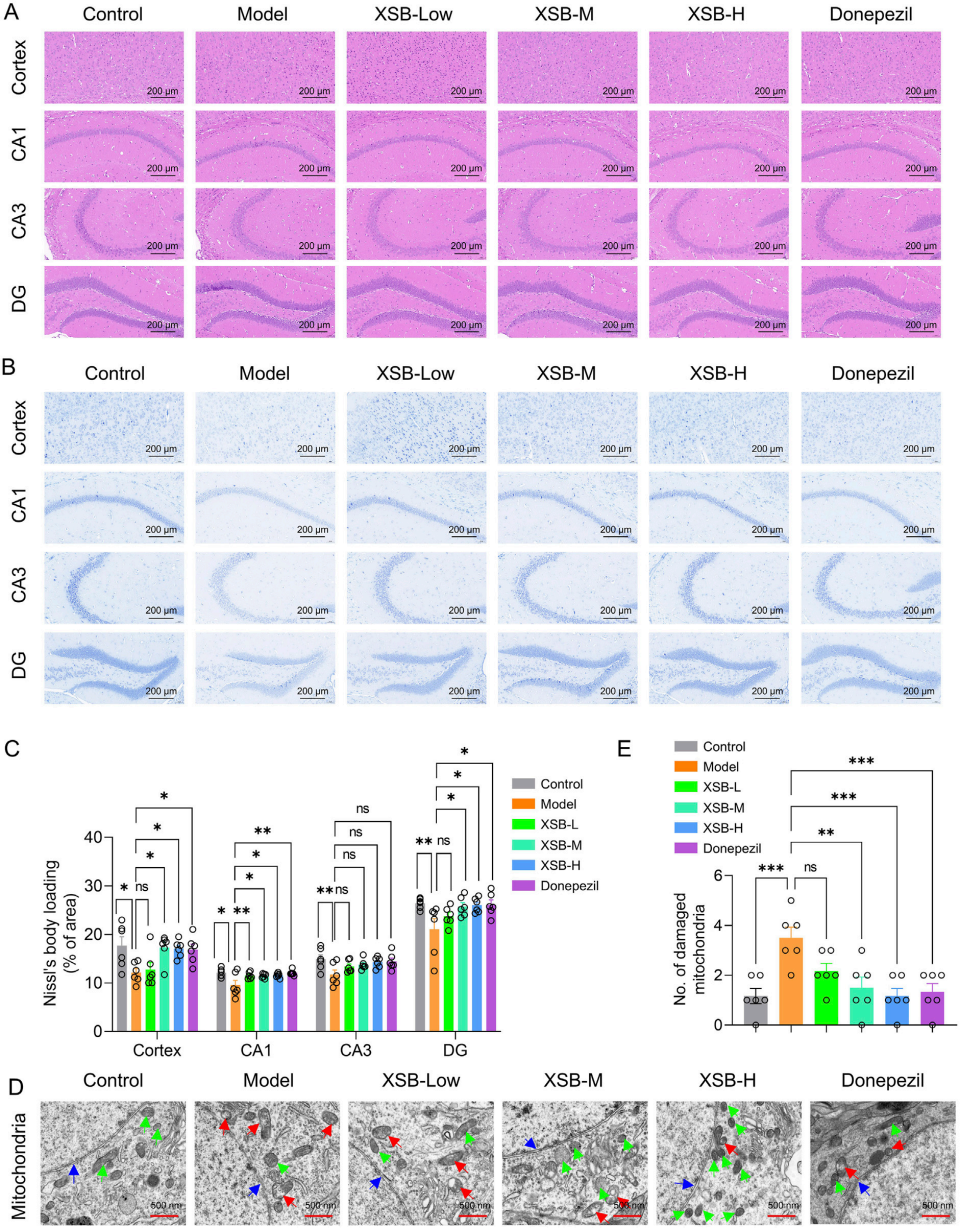
XSB alleviates cortical and hippocampal structural damage in AD model mice. **(A)**: Representative images of H&E staining; **(B)**: Representative images of Nissl staining; **(C)**: Quantitative analysis of Nissl bodies; **(D)**: Representative images of TEM; **(E)**: Quantitative analysis of damaged mitochondria based on D; Green arrows indicate healthy mitochondria, red arrows indicate damaged mitochondria, and blue arrows indicate nuclear envelopes. Data are presented as mean ± SEM; *: P < 0.05, **: P < 0.01, ***: P < 0.001, ****: P < 0.0001, ns: *P >* 0.05. n = 3 mice/group.

H&E staining results revealed that the XSB-L, XSB-M, and XSB-H treatment groups exhibited well-organized and compact neuronal arrangements, with round nuclei and evenly distributed staining in both the cytoplasm and nuclei (Figure 2A). Nissl staining further demonstrated that in the XSB-treated groups, neurons were more densely packed and more intense staining than that in Model group. Notably, the XSB-M and XSB-H groups showed a significant increase in the number of Nissl bodies in the cortex (Figures 2B,C, *P <* 0.05) and hippocampal CA1 (Figures 2B,C, *P <* 0.05) and DG (Figures 2B,C, *P <* 0.05) regions compared to the Model group, with effects comparable to the positive control drug donepezil.

TEM analysis revealed that following XSB treatment, mitochondrial morphology was restored to typical elongated, fusiform shapes, with well-defined mitochondrial cristae and an intact nuclear membrane structure. Furthermore, the number of injured mitochondria was markedly decreased (Figures 2D,E, *P <* 0.001 vs. Model group), with effects similar to the positive control drug donepezil (Figures D,E, *P <* 0.001 vs. Model group). These results demonstrate that XSB effectively alleviates both pathological and ultrastructural damage in the cortex and hippocampus of model mice.

### 3.3 XSB enhances cholinergic system function in the brain of model mice

**FIGURE 3:**
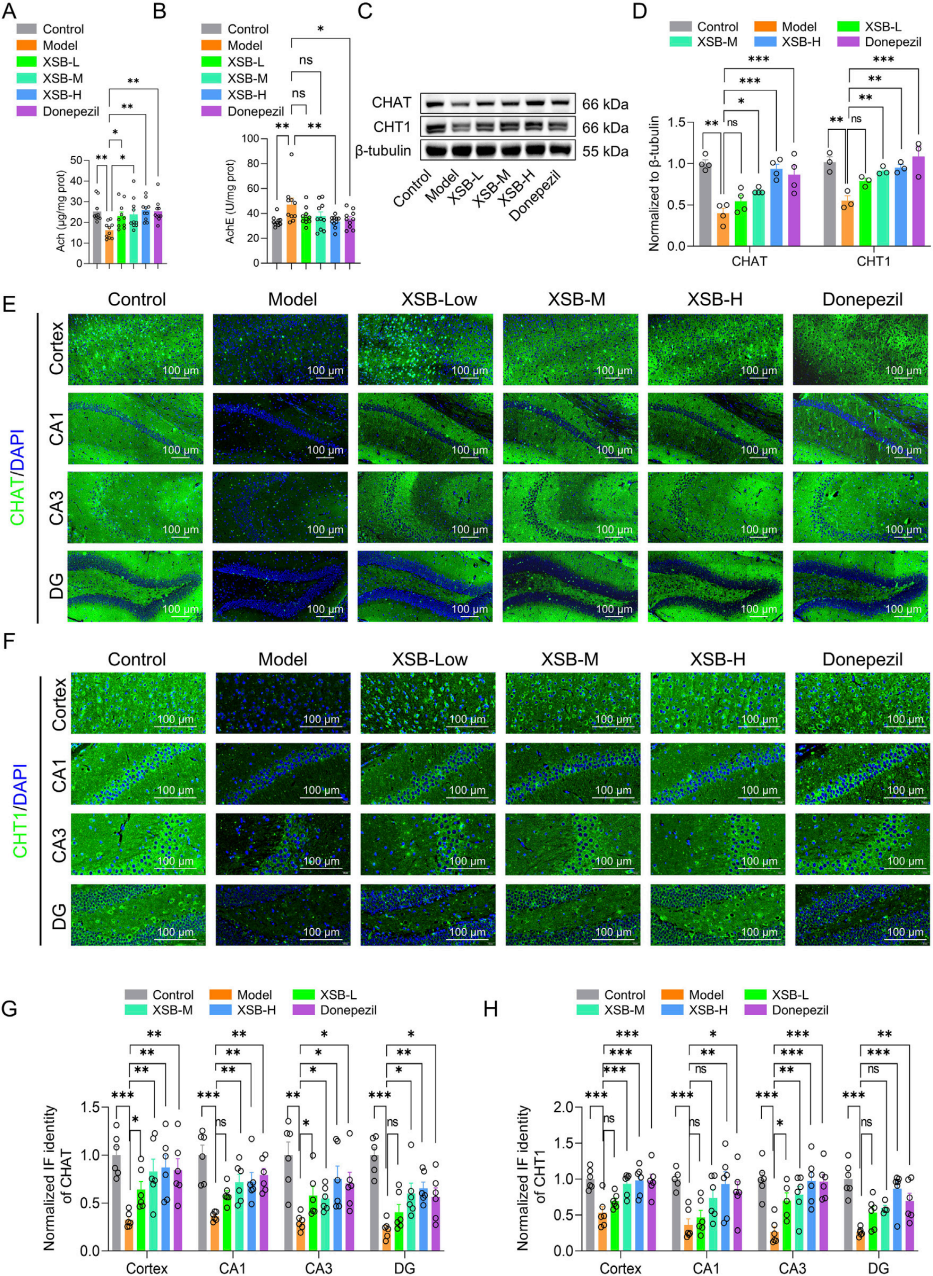
XSB improves the cholinergic system in the brain of AD model mice. **(A)**: ACh levels in the brain of each group; **(B)**: AChE activity; **(C)**: Representative Western blot images of ChAT and CHT1 protein expression in the brain of each group; **(D)**: Quantification of ChAT and CHT1 protein expression based on C; **(E,F)**: Representative immunofluorescence images of ChAT **(E)** and CHT1 **(F)** expressions in the cortex and hippocampus; **(G,H)**: Statistical analysis of ChAT **(G)** and CHT1 **(H)** expressions in the cortex and hippocampal subregions based on **(E,F)**. Data are presented as mean ± SEM; *: P < 0.05, **: P < 0.01, ***: P < 0.001, ****: P < 0.0001, ns: *P >* 0.05. n = 8 mice/group in assays of ACh and AChE, and n = 8 mice/group in Western blot and immunofluorescence assays.

As shown in Figure 3, treatment with XSB-H significantly increased ACh levels (Figure 3A, *P <* 0.05 vs. Model group) and decreased AChE activity (Figure 3B, *P <* 0.05 vs. Model group) in the Model mice. Moreover, both XSB-M and XSB-H treatment significantly elevated the expression of ChAT and CHT1 in the cortex and hippocampus evidenced by western bolt and immunofluorescence data (Figures 3C–H), suggesting that XSB can effectively improve the cholinergic system in the brain of model mice.

### 3.4 XSB rebalances gut microbiota composition in model mice

**FIGURE 4:**
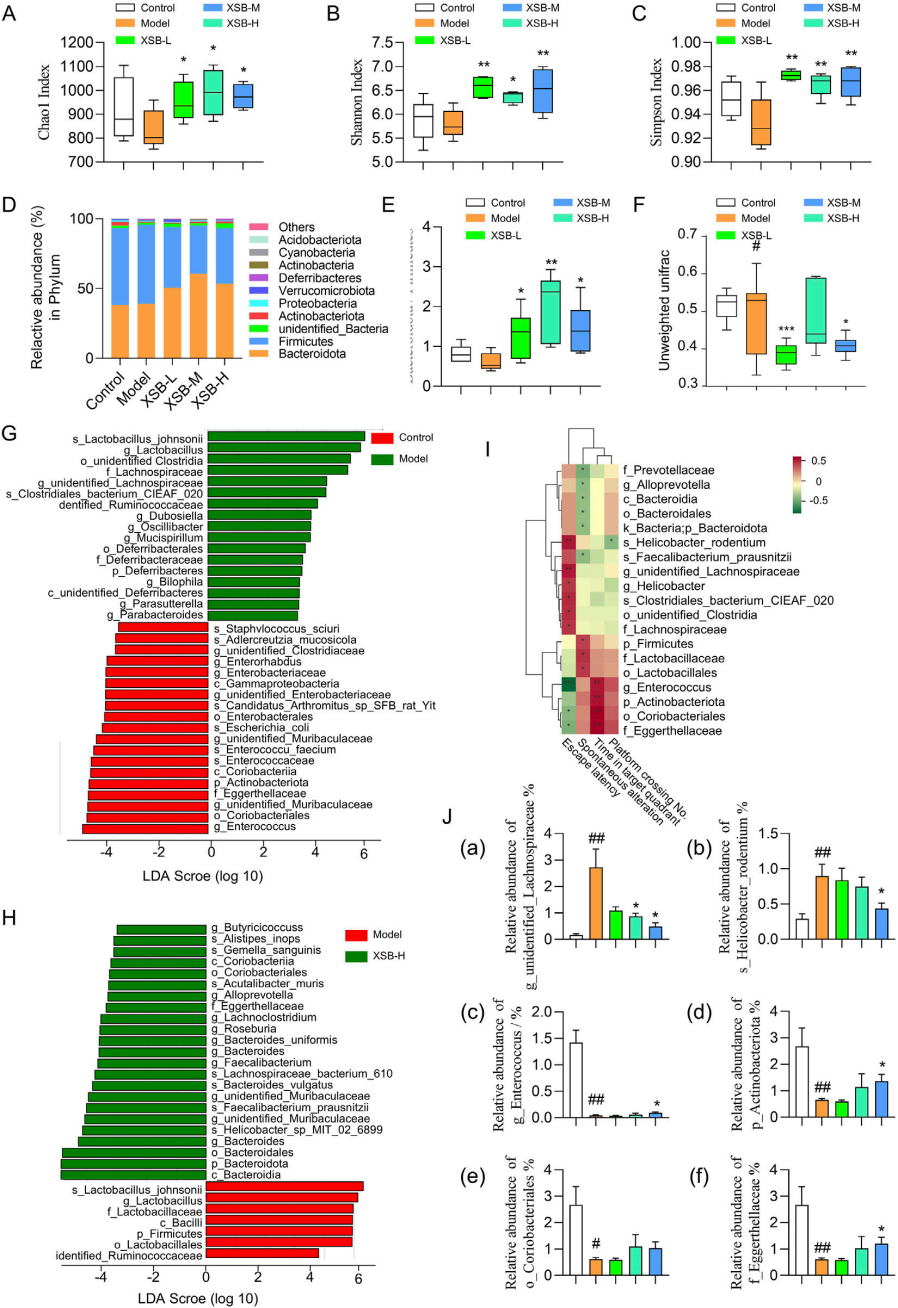
XSB rebalances gut microbiota composition in AD model mice. **(A)**: Chao1 index diversity; **(B)**: Shannon index diversity; **(C)**: Simpson index diversity; **(D)**: Relative abundance of species at the phylum level; **(E)**: ratio of Bacteroidota to Firmicutes relative abundance; **(F)**: Comparison of beta diversity among different groups based on Unweighted UniFrac distance. **(G,H)**: LEfSe analysis revealed significant bacterial differences in fecal microbiota between Model vs. Control **(G)**, and XSB-H vs. Model **(H)**; **(I)**: Spearman correlation analysis between differentially abundant taxa and cognitive function-related indicators. **(J)**: Relative abundance of bacteria associated with cognitive function. (a) g_unidentified_Lachnospiraceae, (b) s_*Helicobacter*_rodentium; (c): g_*Enterococcus*, (d) p_Actinobacteriota, (e) o_Coriobacteriales, (f) f_Eggerthellaceae. Data are presented as mean ± SEM; *: P < 0.05, **: P < 0.01, ***: P < 0.001 vs. Model group; *#: P <* 0.05, #*#: P <* 0.01, ##*#: P <* 0.001 vs. Control group. n = 6 mice/group.

As shown in Figure 4, treatment with XSB, particularly XSB-M and XSB-H, significantly improved microbial diversity, as reflected by lower Chao 1 (Figure 4A), Shannon (Figure 4B) and Simpson indices (Figure 4C). At the phylum level, XSB significantly decreased the relative abundance of *Firmicutes* while increasing *Bacteroidota* (Figures 4D,E), suggesting a shift towards a more balanced microbiota in these groups. The UniFrac distance analysis (Figure 4F) showed that the gut microbiota composition in XSB-treated groups was significantly closer to that of the Control group compared to the Model group.

Additionally, LEfSe analysis identified that *s-Lactobacillus-johnsonii*, g*-Lactobacillus*, *o-unidentified Clostridia*, *f-Lachnospiraceae*, *g-unidentified-Lachnospiraceae*, *s-Clostridiales-bacterium-CIEAF-020*, and *dentified-Ruminococcaceae* were enriched in Model group, while *g-Enterococcus, o-Coriobacteriales*, *s-Enterococcaceae*, *g-unidentified-Muribaculaceae*, *p-Actinobacteriota*, *f-Eggerthellaceae* which were identified beneficial for cognitive function were enriched in Control group (Figure 4G). Compared to Model group, *Bacteroidia*, *Helicobacter*-sp-*MIT*-*02*-*6899*, *g-unidentified*-*Muribaculaceae*, *s-Faecalibacterium-prausnitzii* and *s-Lachnospiraceae-bacterium-610*, associated with gut health and cognitive function, were enriched in XSB-H group (Figure 4H).

Moreover, correlation analysis between cognitive function-related indicators and beneficial bacteria revealed several taxa significantly associated with cognitive performance. For instance, *g_Enterococcus*, *p_Actinobacteriota*, *o_Coriobacteriales*, and *f_Eggerthellaceae* exhibited a positive correlation with cognitive function (Figure 4I, *P <* 0.001), whereas *s_Helicobacter_rodentium* and *g_unidentified_Lachnospiraceae* showed a negative correlation (Figure 4I, *P <* 0.01). Furthermore, in the XSB-treated groups, the abundance of the cognitively harmful taxa, including *s_Helicobacter_rodentium* and *g_unidentified_Lachnospiraceae*, was slightly decreased, while the cognitively beneficial taxa, including *g_Enterococcus*, *p_Actinobacteriota*, *o_Coriobacteriales*, and f*_Eggerthellaceae*, was slightly elevated (Figure 4J). These findings suggest that scopolamine-induced cognitive impairment leads to gut microbiota dysbiosis, while XSB administration partially restores the abundance of beneficial bacteria, thereby re-establishing microbial balance and subsequently improving cognitive function in the model mice.

### 3.5 XSB restores metabolic homeostasis in model mice

**FIGURE 5:**
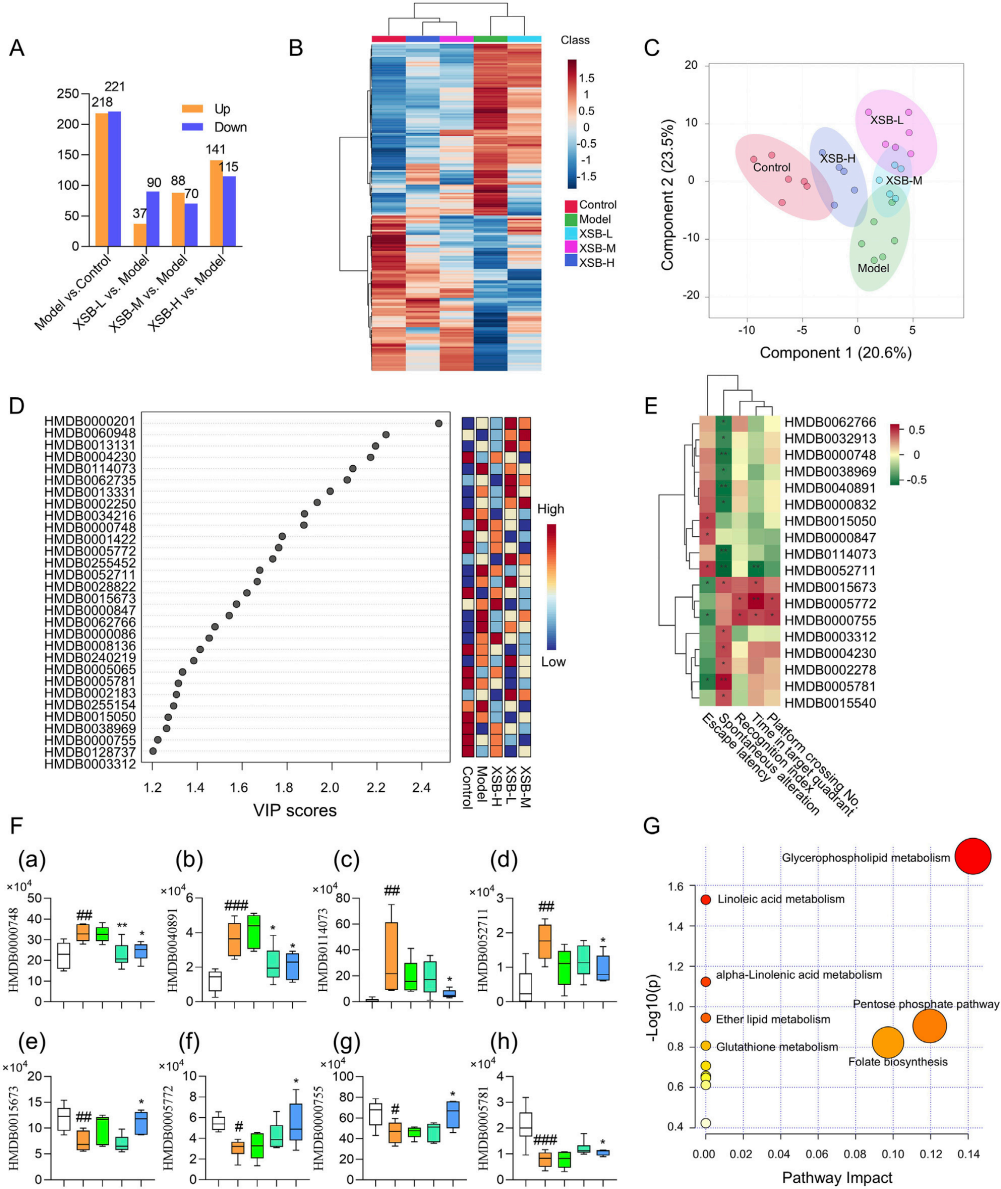
XSB restores metabolic homeostasis in AD model mice. **(A)**: Statistical analysis of the number of differential metabolites among different comparison groups; **(B)**: Heatmap of hierarchical clustering of differential metabolites; **(C)**: PLS-DA score plot of differential metabolites; **(D)**: Heatmap of the top 30 metabolites by VIP values; **(E)**: Pearson correlation analysis between DMs and cognitive function; **(F)**: Relative peak area of metabolites significantly associated with cognitive function, (a) HMDB0000748 (L-3-Phenyllactic acid), (b) HMDB0040891 (3′,4′,5′-Trimethoxycinnamyl alcohol acetate), (c) HMDB0114073 (PE-NMe2 (18:2 (9Z, 12Z)/18:2 (9Z, 12Z))), (d) HMDB0052711 (TG (18:2 (9Z, 12Z)/22:4 (7Z,10Z,13Z, 16Z)/18:2 (9Z, 12Z))), (e) HMDB0015673 (Carglumic acid), (f) HMDB0005772 (Postin), (g) HMDB0000755 (Hydroxyphenyllactic acid); (h) HMDB0005781 (Glycitein); **(G)**: KEGG enrichment analysis of DMs. Data are presented as mean ± SEM; *: P < 0.05, **: P < 0.01, ***: P < 0.001 vs. Model group; *#: P <* 0.05, #*#: P <* 0.01, ##*#: P <* 0.001 vs. Control group. n = 6 mice/group.

As shown in Figure 5A, we identified 439 DMs between the Model and Control groups (218 upregulated and 221 downregulated), 127 DMs between the XSB-L and Model groups (37 upregulated and 90 downregulated), 158 DMs between the XSB-M and Model groups (88 upregulated and 70 downregulated), and 256 DMs between the XSB-H and Model groups (141 upregulated and 115 downregulated). The clustering heatmap in Figure 5B and the PLS-DA score plot in Figure 5C reveal that the XSB-H and XSB-M groups cluster more closely with the Control group. This suggests that intraperitoneal injection of scopolamine induces alterations in serum metabolites, and that XSB treatment can partially restore these metabolic disturbances. Figure 5D shows the top 30 metabolites with VIP >1 from the PLS-DA analysis. Pearson correlation analysis of these metabolites with cognitive function-related indicators indicated several significant correlations. Specifically, HMDB0000748 (L-3-Phenyllactic acid), HMDB0040891 (3′,4′,5′-Trimethoxycinnamyl alcohol acetate), HMDB0114073 (PE-NMe2 (18:2 (9Z, 12Z)/18:2 (9Z, 12Z))), and HMDB0052711 (TG (18:2 (9Z, 12Z)/22:4 (7Z,10Z,13Z, 16Z)/18:2 (9Z, 12Z))) were negatively correlated with cognitive function, whereas HMDB0015673 (Carglumic acid), HMDB0005772 (Postin), HMDB0000755 (Hydroxyphenyllactic acid), and HMDB0005781 (Glycitein) exhibited positive correlations (Figure 5E). Additionally, the abnormal levels of these metabolites were partially corrected by XSB treatment (Figure 5F).

KEGG enrichment analysis of the DMs with VIP >1 further revealed that XSB primarily restores metabolic balance in model mice by modulating key metabolic pathways, including glycerophospholipid metabolism, linoleic acid metabolism, alpha-Linolenic acid metabolism, ether lipid metabolism, glutathione metabolism, pentose phosphate pathway and folate biosynthesis (Figure 5G).

### 3.6 XSB restores hippocampal transcriptomic homeostasis in model mice

**FIGURE 6:**
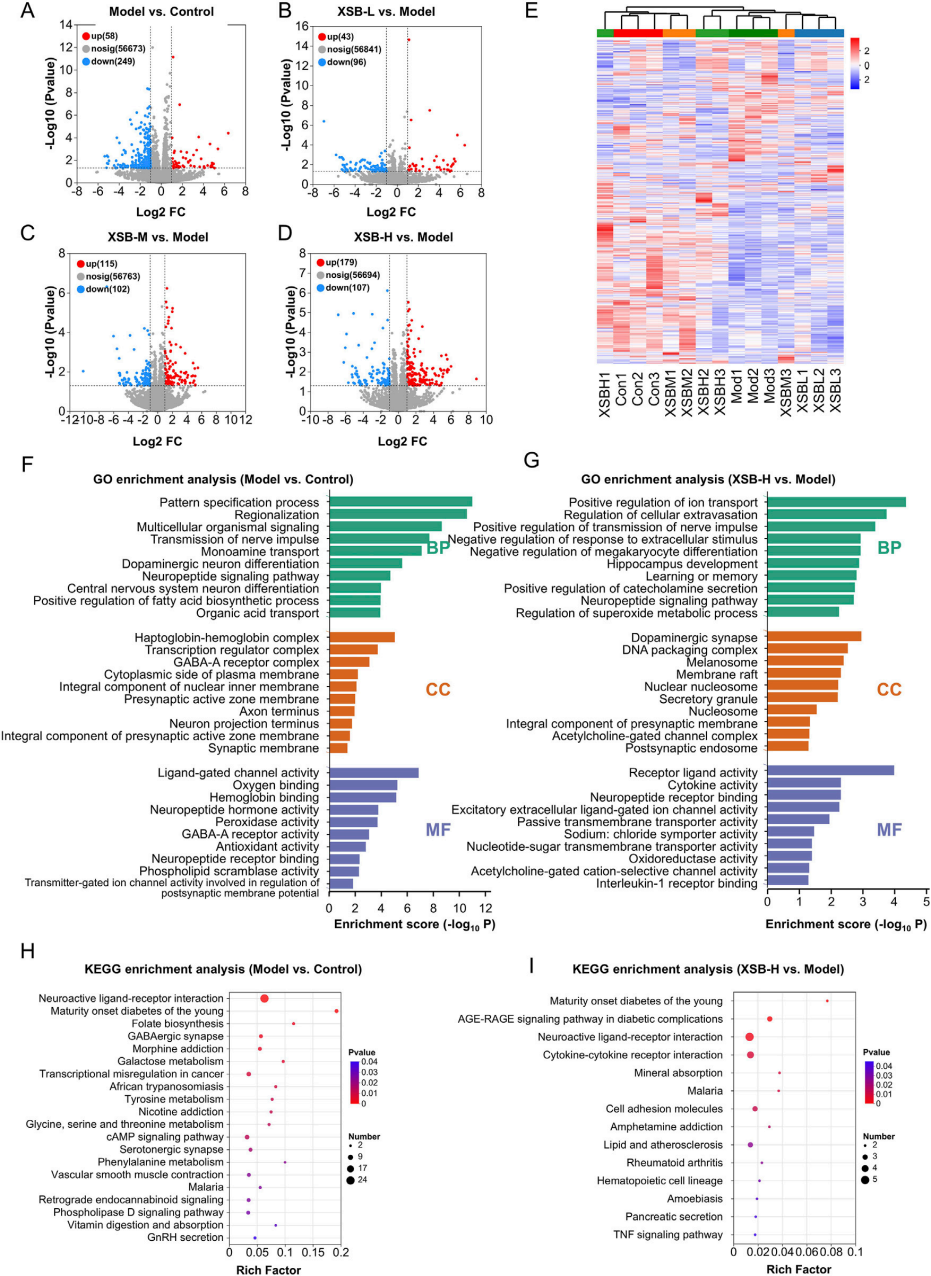
XSB restores hippocampal transcriptomic homeostasis in AD model mice. **(A)**: Volcano maps depicting DEGs in Model vs. Control **(A)**, XSB-L vs. Model **(B)**, XSB-M vs. Model **(C)**, and XSB-H vs. Model **(D)** groups; **(E)** Cluster analysis heat maps of DEGs among different groups; **(F,G)**: GO enrichment analysis of DEGs in the Model vs. Control group **(F)**, and Model XSB-H group **(G)**; **(H,I)**: KEGG enrichment analysis of DEGs in the Model vs. Control group **(H)**, and Model XSB-H group **(I)**. n = 3 mice/group.

As illustrated in Figures 6A–D, the Model group exhibited 307 DEGs compared to the Control group, with 58 genes upregulated and 249 downregulated. In contrast, the XSB-L group demonstrated 139 DEGs relative to the Model group, comprising 43 upregulated and 96 downregulated genes. The XSB-M group exhibited 217 DEGs, with 115 upregulated and 102 downregulated, while the XSB-H group displayed 286 DEGs, including 179 upregulated and 107 downregulated, when compared to the Model group. Subsequently, these DEGs underwent hierarchical clustering analysis. As shown in Figure 6E, the XSB-H and XSB-M groups clustered closely with the Control group, indicating a potential reversal of gene expression patterns by XSB treatment.

The GO functional annotation revealed that the DEGs in the XSB-H vs. Model comparison were predominantly enriched in cellular components such as the dopaminergic synapse, DNA packaging complex, membrane raft, integral component of the presynaptic membrane, and acetylcholine-gated channel complex. Additionally, these genes were associated with diverse molecular functions, including receptor-ligand activity, cytokine activity, neuropeptide receptor binding, passive transmembrane transporter activity, oxidoreductase activity, and acetylcholine-gated cation-selective channel activity. Functionally, these DEGs were implicated in key biological processes such as the regulation of nerve impulse transmission, response to extracellular stimuli, megakaryocyte differentiation, hippocampal development, learning and memory, neuropeptide signaling pathways, superoxide metabolic processes, and interleukin-1 receptor binding (Figure 6G). Furthermore, KEGG pathway enrichment analysis indicated that the DEGs in the XSB vs. Model group were significantly associated with neuroactive ligand-receptor interactions, cytokine-cytokine receptor interactions, cell adhesion molecules, and the TNF signaling pathway (Figure 6I). The GO and KEGG enrichment analysis results of Model vs. Control group were as shown in Figures 6F,H. These findings suggest that intraperitoneal scopolamine injection disrupts hippocampal gene expression, while XSB treatment partially restores homeostasis through the regulation of neuroactive ligand-receptor interactions and modulation of neuroinflammatory pathways, both of which are critical for maintaining cognitive function.

### 3.7 Multi-omics integration: transcriptomics, metabolomics, and gut microbiota-metabolome analysis

**FIGURE 7:**
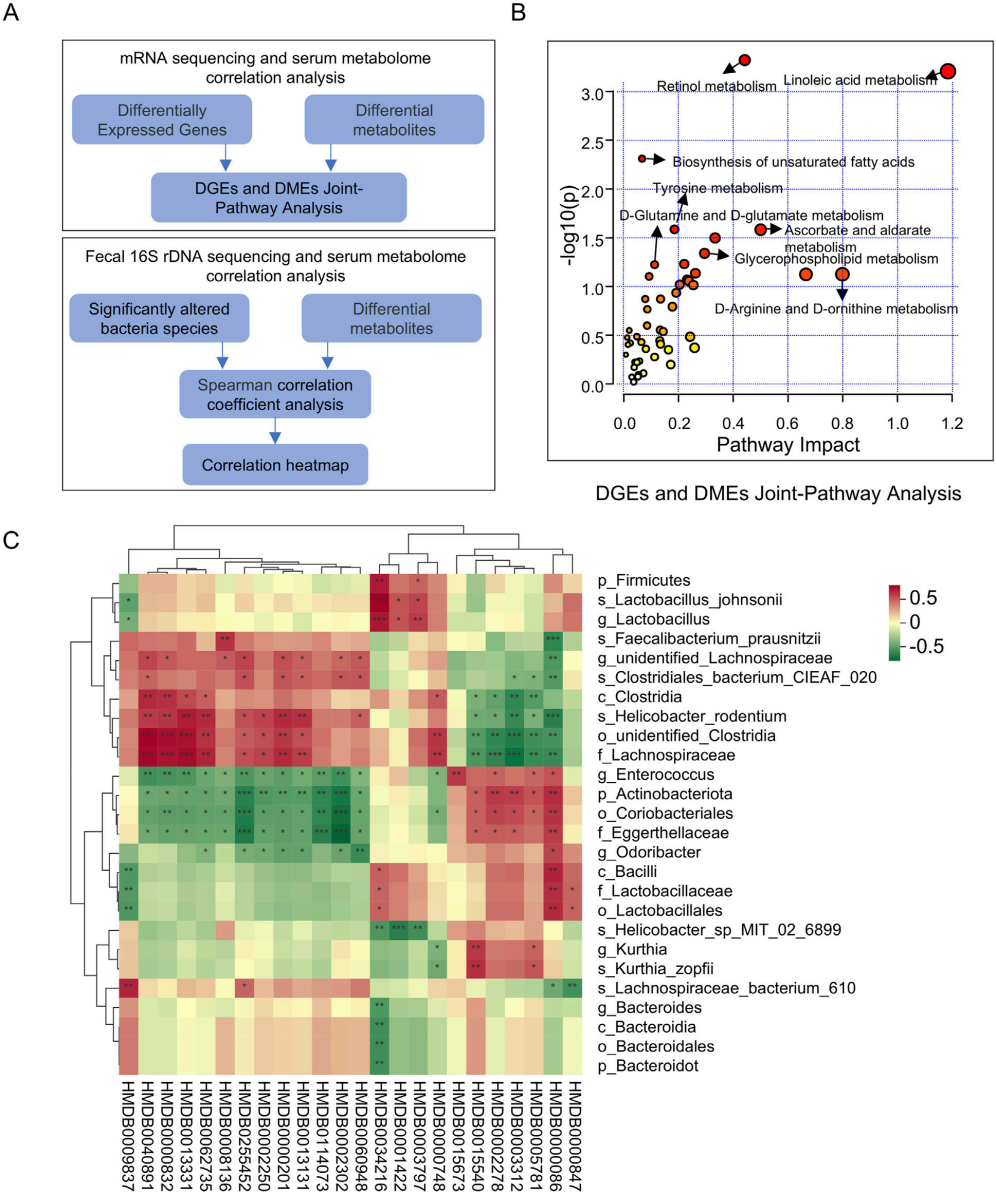
Multi-Omics integration: transcriptomics, metabolomics, and gut microbiota-metabolome analysis. **(A)** Schematic of Multi-Omics Integration Analysis; **(B)** DGEs and DMEs Joint-Pathway Analysis; **(C)** Spearman correlation coefficient analysis of DMs and Significantly altered bacteria species.

Using MetaboAnalyst, joint pathway analysis was performed to integrate DEGs and DMs Figure 7A. The results indicated that XSB primarily regulates metabolic pathways, including linoleic acid metabolism, retinol metabolism, biosynthesis of unsaturated fatty acids, tyrosine metabolism, and glycerophospholipid metabolism (Figure 7B). To explore potential associations between DMs and gut microbiota, Spearman correlation analysis was performed. The results revealed significant associations between specific bacterial species and DMs (Figure 7C). For instance, bacteria beneficial to cognitive function, such as *g_Enterococcus*, *p_Actinobacteriota*, *o_Coriobacteriales*, and *f_Eggerthellaceae*, exhibited negative correlations with metabolites detrimental to cognitive function, including HMDB0114073, HMDB0040891, and HMDB0000748, while showing positive correlations with metabolites beneficial to cognition, such as HMDB0005781 and HMDB0015673. In contrast, bacteria detrimental to cognitive function, such as *g_unidentified_Lachnospiraceae* and *s_Helicobacter_rodentium*, demonstrated positive correlations with HMDB0114073, HMDB0040891, and HMDB0000748, but negative correlations with HMDB0005781 and HMDB0015673. These results highlight the critical role of XSB in modulating key metabolic pathways and the interplay between gut microbiota and metabolites, suggesting a potential mechanism through which XSB improves cognitive function by restoring microbial and metabolic homeostasis.

### 3.8 XSB inhibits neuroinflammation in model mice

**FIGURE 8:**
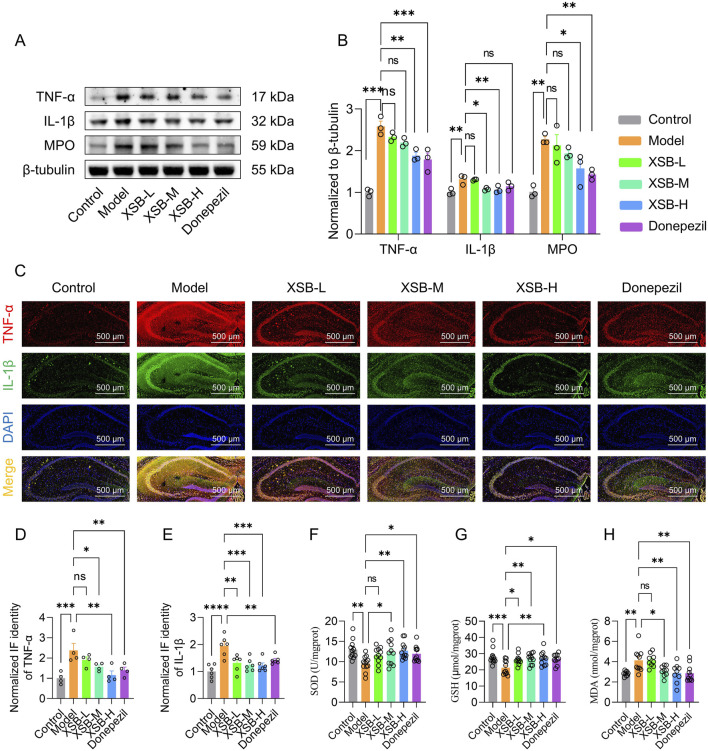
XSB inhibits neuroinflammation in AD model mice. **(A)**: Representative immunoblot images of TNF-α, IL-1β, MPO and β-tubulin. **(B)**: quantitative analysis of TNF-α, IL-1β and MPO expression based on A; **(C)**: Representative immunofluorescence images of TNF-α and IL-1β. **(D,E)**: Quantitative analysis of TNF-α **(D)** and IL-1β **(E)** based on C; **(F-H)**: SOD activity **(F)**, GSH content **(G)** and MDA content **(H)** in brains in different groups. Data are presented as mean ± SEM; *: P < 0.05, **: P < 0.01, ***: P < 0.001, ns: *P >* 0.05, n = 3 mice/group in Western blot and immunofluorescence assays, n = 9–12 mice/group in SOD, GSH and MDA assays.

Based on the enrichment of inflammatory-related signaling pathways, such as the TNFα signaling pathway and arachidonic acid metabolism, identified in the KEGG analysis, we further assessed the levels of key inflammatory mediators-TNF-α, IL-1β, and MPO-in the hippocampus using Western blot analysis. As shown in Figures 8A,B, XSB treatment significantly reduced the protein expression levels of TNF-α (*P <* 0.01 vs. Model group), IL-1β (*P <* 0.05 vs. Model group), and MPO (*P <* 0.05 vs. Model group) in the hippocampus. Similar results were observed in immunofluorescence experiments (Figures 8C–E), further confirming the anti-neuroinflammatory effects of XSB. Moreover, XSB treatment increased the activity of SOD (*P <* 0.01 vs. Model group), elevated the levels of GSH (*P <* 0.01 vs. Model group), and reduced MDA levels (*P <* 0.01 vs. Model group), thereby alleviating oxidative stress damage in the brains of model mice (Figures 8F–H).

### 3.9 XSB enhances synaptic protein expression and synaptic density

**FIGURE 9:**
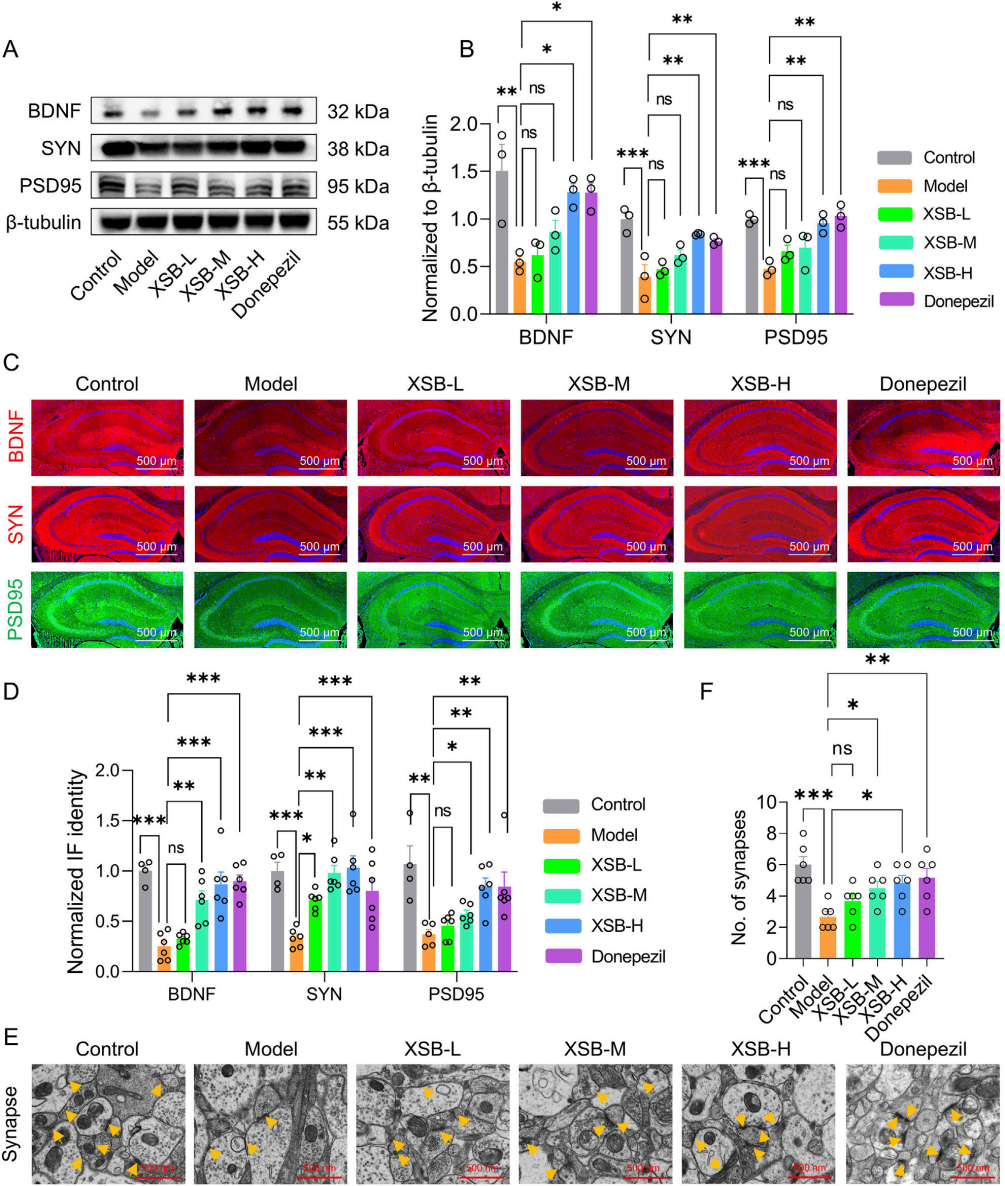
XSB enhances synaptic protein expression and synaptic density. **(A)**: Representative immunoblot images of BNDF, SYN, PSD95 and β-tubulin. **(B)**: Quantitative analysis of BNDF, SYN and PSD95 expression based on A; **(C)**: Representative immunofluorescence images of BNDF, SYN and PSD95; **(D)**: Quantitative analysis of BNDF, SYN and PSD95 expression based on C; **(E)**: Representative TEM images of synapses in neurons in hippocampal CA1 region; yellow arrows indicate synapses; **(F)**: Quantitative analysis of synapse number in different groups. Data are presented as mean ± SEM; *: P < 0.05, **: P < 0.01, ***: P < 0.001, ns: *P >* 0.05, n = 3 mice/group.

Given that KEGG enrichment analysis revealed the involvement of neuroactive ligand-receptor interaction signaling pathways, which are crucial for synaptic signaling, we then evaluated the expression of critical synaptic proteins-BDNF, SYN, and PSD95-using Western blot and immunofluorescence methods. As shown in Figures 9A,B, XSB treatment notably increased the expression of BDNF (*P <* 0.05), SYN (*P <* 0.001), and PSD95 (*P <* 0.001) compared to Model group. Immunofluorescence results (Figures 9C,D) supported these findings, further confirming the beneficial effects of XSB on synaptic damage. Additionally, XSB treatment significantly increased synaptic density (Figures 9E,F, *p <* 0.05 vs. Model group). These results suggest that XSB promotes the expression of BDNF, SYN, and PSD95 and enhances synaptic density, potentially contributing to the improvement of cognitive function in model mice.

### 3.10 Transplantation of XSB-FM rescues cognitive dysfunction and intestinal barrier injures in AD model mice

**FIGURE 10:**
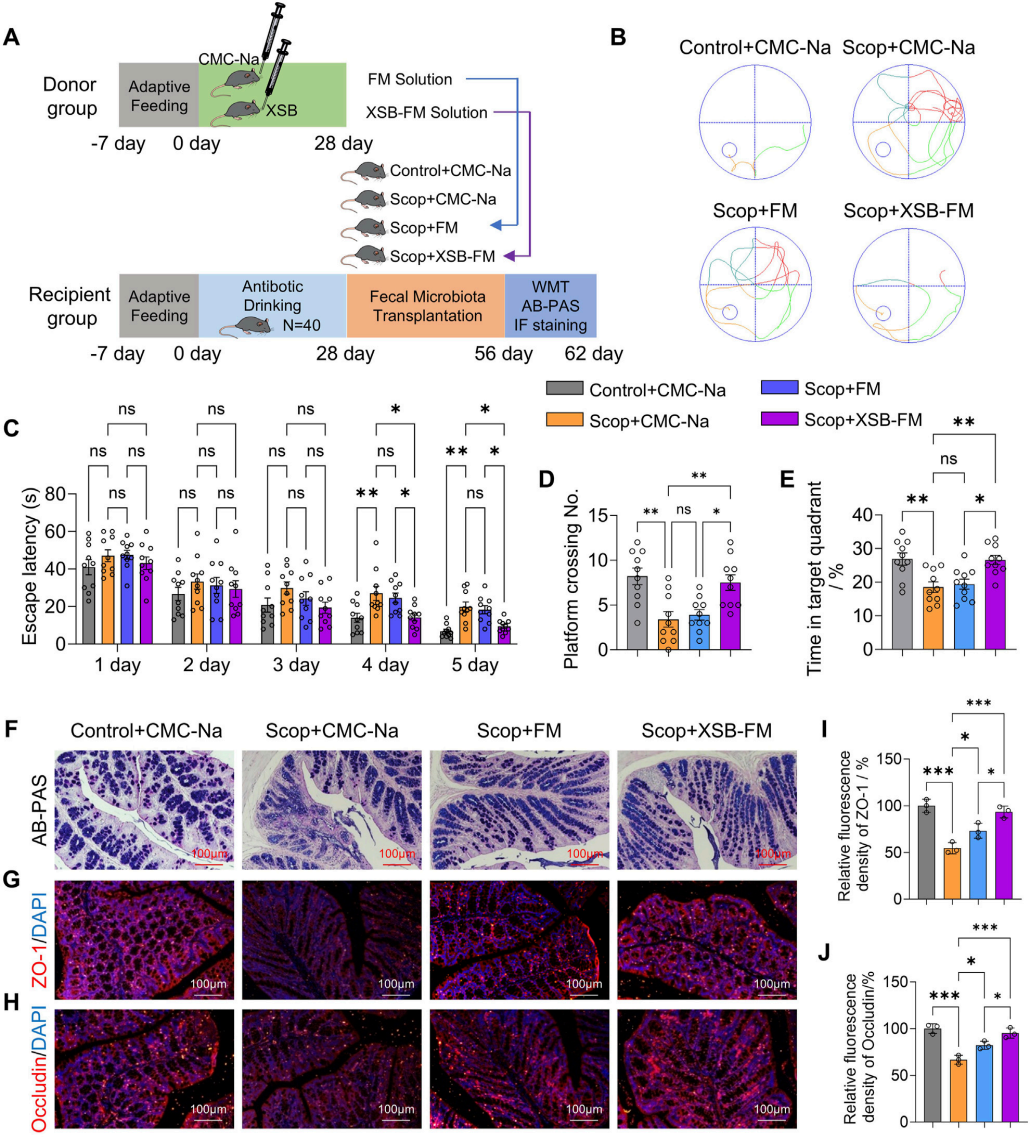
Transplantation of XSB-FM rescues cognitive dysfunction and intestinal barrier injures in AD model mice. **(A)**: Schematic diagram of the FMT experiment; **(B)**: Representative swimming track diagrams of mice in the MWM test; **(C-E)**: Statistical graph of escape latency **(C)**, the number of platform crossings **(D)** and the percentage of time spent in the target quadrant **(E)**. **(F)**: Representative images of AB-PAS staining; **(G,H)**: Representative immunofluorescence images of ZO-1 **(G)** and Occludin **(H)**. **(I,J)**: Statistical graph of ZO-1 **(I)** and Occludin **(J)** based on **(G,H)**, respectively, n = 3 mice/group. Data are presented as mean ± SEM; *: P < 0.05, **: P < 0.01, ***: P < 0.001, ns: *P >* 0.05, n = 10 mice/group in MWM test, n = 3 mice/group in AB-PAS, ZO-1 and Occludin staining tests.

The results demonstrated that transplantation of XSB-FM significantly decreased the escape latency (Figures 10B,C, *p* < 0.05 vs. Scop-FM group on 5th day), increased the platform crossing number (Figure 10D, *P* < 0.05 vs. Scop-FM group) and the time spent in target square (Figure 10E, *P* < 0.05 vs. Scop-FM group). Moreover, compared with Scop-FM group, transplantation of XSB-FM markedly boosted the AB-PAS positive cells (Figure 10F) in the intestinal mucosa, and upregulated ZO-1 (Figures 10G,I, *p* < 0.05) and Occludin (Figures 10H,J, *p* < 0.05) protein expressions in dementia mice induced by scopolamine. These findings indicated that transplantation of XSB-FM rescues cognitive dysfunction and intestinal barrier injures in AD model mice, which further confirmed that gut microbiota remodeling is essential for XSB’s therapeutic efficacy.

## 4 Discussion

AD is a prevalent and devastating neurodegenerative disorder characterized by progressive cognitive decline, synaptic dysfunction, and pathological features such as neuroinflammation and cholinergic system impairment. TCM has been recognized as a promising approach for managing neurodegenerative diseases, with various natural products showing potential therapeutic benefits.

**FIGURE 11:**
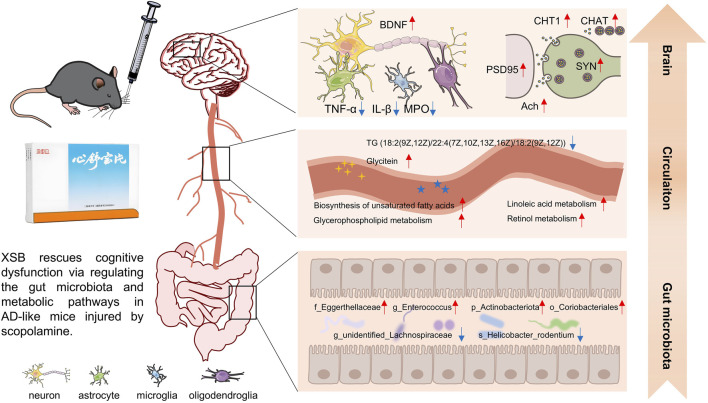
Overview of multiple regulations of XSB on AD model mice induced by scopolamine.

In the present study, we explored the potential therapeutic effects of XSB, a traditional Chinese patent medicine, in an AD mouse model induced by scopolamine. Our findings demonstrated that XSB significantly alleviated cognitive impairments, ameliorated hippocampal neuronal damage, and mitigated key neuropathological hallmarks, including synaptic dysfunction, dysregulation of the cholinergic nervous system, neuroinflammation, and oxidative stress in AD mice. Moreover, XSB increased the abundance of beneficial bacteria (e.g., *g_Enterococcus*, *p_Actinobacteriota*, *o_Coriobacteriales*, and *f_Eggerthellaceae*), while decreasing the abundance of harmful bacteria (e.g., *s_Helicobacter_rodentium* and *g_unidentified*_*Lachnospiraceae*), effectively modulating the gut microbiota balance. XSB also regulated metabolic pathways related to tyrosine metabolism, glycerophospholipid metabolism, and unsaturated fatty acid metabolism, leading to a reduction in harmful metabolites such as HMDB0000748 (L-3-Phenyllactic acid), HMDB0040891 (3′,4′,5′-Trimethoxycinnamyl alcohol acetate), HMDB0114073 (PE-NMe2 (18:2 (9Z, 12Z)/18:2 (9Z, 12Z))), and HMDB0052711 (TG (18:2 (9Z, 12Z)/22:4 (7Z,10Z,13Z, 16Z)/18:2 (9Z, 12Z))), while increasing beneficial metabolites such as HMDB0015673 (Carglumic acid), HMDB0005772 (Postin), HMDB0000755 (Hydroxyphenyllactic acid), and HMDB0005781 (Glycitein) (Figure 11). In addition, XSB-FM transformation successfully alleviated cognitive and intestinal barrier damages. These findings collectively underscore the multifaceted protective effects of XSB in mitigating key pathological processes associated with AD, highlighting its potential as a therapeutic intervention.

The cholinergic system, particularly in the basal forebrain, cortex, and hippocampus, plays a critical role in memory formation and cognition. Dysfunction of this system is regarded as one of the key pathogenic mechanisms underlying AD (Hampel et al., 2018). Cholinergic neurons are primarily located in the basal nucleus and the diagonal band of Broca in the basal forebrain, which provide the main cholinergic innervation to the hippocampus and cerebral cortex (Chen et al., 2018). Neurotransmitters transmit signals from the neurons of the basal forebrain to hippocampal neurons, a process that involves the synthesis, packaging, secretion, and clearance of the neurotransmitters. ACh is synthesized by the enzyme ChAT through the combination of choline and acetyl-CoA. ACh is then transported into synaptic vesicles from the cytoplasm by the vesicular acetylcholine transporter (VAChT). Upon neuronal firing, ACh stored in the vesicles is released into the synaptic cleft, thereby completing signal transduction. Subsequently, some of the ACh is hydrolyzed by AChE into choline and acetate. Choline is then reabsorbed by the high-affinity choline transporter (CHT) located on the presynaptic membrane and is recycled by ChAT to synthesize more ACh (Chen et al., 2018). Any disruption in these processes may lead to cholinergic dysfunction, which in turn causes cognitive impairment.

Scopolamine, a non-selective muscarinic acetylcholine receptor antagonist, induces central cholinergic dysfunction, thereby impairing learning ability and short-term memory (Lee et al., 2014). Moreover, scopolamine has been shown to induce neurotrophic deficits, oxidative stress, neuroinflammation, and increased Aβ accumulation, which closely resemble the pathological features observed in AD patients (Xiao et al., 2020; Tang, 2019; Yun et al., 2022). Thus, scopolamine-induced cognitive impairment models are widely employed for evaluating the anti-dementia efficacy of potential therapeutic agents. In this study, we found that XSB treatment effectively restored the cholinergic dysfunction induced by scopolamine, as evidenced by reduced AChE activity, increased ACh levels, and upregulation of ChAT and CHT1 protein expression (Figure 3).

Gut microbiota plays a crucial role in the development and progression of AD and other neurodegenerative disorders. Dysbiosis, characterized by an imbalance in the gut microbial composition, has been linked to neuroinflammation, oxidative stress, and neuronal dysfunction, all of which contribute to cognitive decline in AD. Our findings demonstrated that treatment with XSB effectively restored microbial diversity, increasing the abundance of beneficial taxa-including *Enterococcus*, *Actinobacteriota*, *Coriobacteriales*, and *Eggerthellaceae* (Figure 4). *Enterococcus* has been reported to be significantly reduced in both APP/PS1 transgenic mice and Aβ-induced mouse models (Jung et al., 2022; Qian et al., 2023). The phylum *Actinobacteriota*, which includes beneficial genera such as *Bifidobacterium*, has demonstrated neuroprotective effects by stimulating BDNF production and reducing pro-inflammatory cytokines such as TNF-α and IL-1β (Wang et al., 2025), thereby alleviating neuroinflammation and supporting cognitive function (Yin et al., 2024). Although elevated levels of *Coriobacteriales_Incertae_Sedis* have been observed in obese individuals and are thought to be negatively associated with cognitive performance (Zhao T. et al., 2023), it is important to recognize that the *Coriobacteriales* order comprises diverse genera and strains with potentially distinct biological functions. Members of the *Eggerthellaceae* family have been positively associated with white matter integrity and memory performance (Hammond et al., 2023), although their abundance tends to decline with aging and in APP/PS1 mice (Qian et al., 2023). These bacteria are capable of converting daidzein, an isoflavone found in soy products, into equol-an estrogen-like compound with neuroprotective properties (Hammond et al., 2023).

Meanwhile, XSB significantly suppressed the abundance of detrimental taxa including *Helicobacter rodentium* and *Lachnospiraceae* (Figure 4). *Helicobacter rodentium* has been associated with intestinal inflammation (Zhao B. et al., 2023), which may exacerbate neuroinflammation and cognitive deficits. Similarly, *Lachnospiraceae UCG-001* has been identified as a hub and keystone bacterium in APP/PS1 mice due to its role in quinolinic acid synthesis, a neurotoxic metabolite implicated in AD pathology (Li et al., 2023). Therefore, XSB-mediated regulation of the gut microbiota not only restores microbial homeostasis but also potentially reduces the production of pro-inflammatory metabolites while increasing neuroprotective metabolite levels.

mRNA-seq analysis revealed that XSB treatment modulates neuroactive ligand-receptor interactions, cytokine-cytokine receptor interactions, and the TNF signaling pathway, thereby restoring the disrupted hippocampal transcriptome. Additionally, Western blot and IF analyses demonstrated that XSB treatment significantly reduced TNF-α and IL-1β protein levels (Figure 8) while increasing BDNF, SYN, and PSD95 expression in the hippocampus of AD model mice (Figure 9). BDNF, a neurotrophin essential for the survival and function of serotonergic, hippocampal, and cortical neurons (Allen et al., 2013) is often reduced in AD, contributing to Aβ accumulation, tau phosphorylation, neuroinflammation, and neuronal apoptosis (Gao et al., 2022; Wang et al., 2023; Wang et al., 2019). Moreover, BDNF plays a crucial role in maintaining synaptic plasticity and cognitive function by promoting the expression of SYN and PSD95, two key proteins vital for synaptic integrity (Wang et al., 2022). Notably, XSB treatment significantly increased BDNF levels and enhanced synaptic density in the hippocampal CA1 region (Figure 9), suggesting that XSB may mitigate synaptic dysfunction and cognitive decline in AD by restoring synaptic integrity and neurotrophic support.

The integration of transcriptomics and metabolic analysis revealed that glycerophospholipid metabolism, linoleic acid metabolism, and the biosynthesis of unsaturated fatty acids are the key metabolic pathways regulated by XSB treatment. Glycerophospholipid metabolism is essential for maintaining neuronal membrane integrity, regulating inflammatory responses, and supporting mitochondrial function (Frisardi et al., 2011). Abnormalities in glycerophospholipid metabolism have been observed in APP/PS1 mice (Qian et al., 2023), where they directly exacerbate AD progression by promoting Aβ deposition and tau protein tangles (Tong et al., 2024), potentially linked to dysregulated gut microbiota and neuroinflammation (Qian et al., 2023; Tian et al., 2022). Linoleic acid, an essential omega-6 polyunsaturated fatty acid, is metabolized into various bioactive molecules, including arachidonic acid and eicosanoids, which can drive inflammatory responses (Alarcon-Gil et al., 2022). In AD, chronic neuroinflammation is a key driver of neuronal damage, and dysregulated fatty acid metabolism-particularly elevated levels of pro-inflammatory metabolites-further exacerbates this pathology. Correlation analysis revealed a significant association between these metabolites and gut microbiota composition (Figure 9), reinforcing the role of XSB in restoring both microbial and metabolic homeostasis. To determine whether the therapeutic effects of XSB are mediated through modulation of the gut microbiota, FMT experiments were performed. The results showed that XSB-FM significantly improved cognitive function (Figures 10B–E) and ameliorated intestinal barrier damage (Figures 10F–J) in AD model mice. These findings highlight gut microbiota remodeling as a critical mechanism underlying the therapeutic efficacy of XSB.

In summary, we systematically explored the potential mechanisms of XSB intervention in AD from three complementary omics perspectives-gut microbiota, metabolomics, and transcriptomics. The modulation of gut microbial composition, characterized by the enrichment of *Actinobacteriota* and *Eggerthellaceae* and the reduction of pathogenic bacteria such as *Helicobacter rodentium*, suggests that XSB may exert regulatory effects on the intestinal microenvironment. These microbial changes were accompanied by significant alterations in metabolites related to amino acid metabolism, bile acid metabolism, and neuroinflammation, including increased levels of Glycitein and Carglumic acid and decreased levels of pro-inflammatory metabolites such as L-3-Phenyllactic acid. These shifts in the metabolic landscape may, in turn, influence central nervous system function, as evidenced by transcriptomic findings showing the involvement of key signaling pathways such as TNFα and neuroactive ligand-receptor interaction. Notably, the upregulation of BDNF, SYN, and PSD95 in the hippocampus indicates enhanced synaptic function and neuroplasticity. By integrating insights from these three omics layers, our findings suggest that XSB may ameliorate AD-related cognitive decline through a microbiota-metabolite-brain axis, ultimately improving neuroinflammatory status, neurotransmission, and synaptic integrity.

## 5 Conclusion

In conclusion, XSB significantly ameliorates scopolamine-induced cognitive impairment in AD model mice through a network of interconnected mechanisms. These include attenuating neuropathological damage, inhibiting neuroinflammation and oxidative stress, restoring cholinergic function, enhancing synaptic activity, and modulating gut microbiota composition. Notably, XSB affects key metabolic pathways, including the biosynthesis of unsaturated fatty acids, tyrosine metabolism, and glycerophospholipid metabolism. Integrated analyses of the gut microbiota, metabolomics, and transcriptomics indicate that XSB may exert its neuroprotective effects via the microbiota-metabolite-brain axis, thereby improving neuroinflammation, neurotransmission, and synaptic integrity. Collectively, these findings underscore the therapeutic potential of XSB as a multifactorial intervention for alleviating cognitive deficits in AD.

## Data Availability

The original contributions presented in the study are included in the article/Supplementary Material, further inquiries can be directed to the corresponding authors.

## Glossary

AD: Alzheimer’s Disease
VaD: Vascular Dementia
XSB: Xinshubao Tablet
Aβ: Amyloid-Beta
NFTs: Neurofibrillary Tangles
H&E: Hematoxylin-Eosin
NOR: Novel Object Recognition
MWM: Morris Water Maze
TCM: Traditional Chinese Medicine
TEM: Transmission Electron Microscopy
mRNA-seq: mRNA Sequencing
ACh: Acetylcholine
AChE: Acetylcholinesterase
CHAT: Choline Acetyltransferase
CHT1: Choline Transporter 1
BDNF: Brain-Derived Neurotrophic Factor
PSD95: Postsynaptic Density Protein 95
AB-PAS: Alcian blue-periodic acid-Schiff
SYN: Synaptophysin
TNF-α: Tumor Necrosis Factor-Alpha
IL-1β: Interleukin-1 Beta
MPO: Myeloperoxidase
SOD: Superoxide Dismutase
GSH: Reduced Glutathione
MDA: Malondialdehyde
DEGs: Differentially Expressed Genes
DMs: Differential Metabolites
GO: Gene Ontology
KEGG: Kyoto Encyclopedia of Genes and Genomes
LC-MS: Liquid Chromatography-Mass Spectrometry
LEfSe: Linear Discriminant Analysis Effect Size
PLS-DA: Partial Least Squares Discriminant Analysis
SEM: Standard Error of the Mean
ANOVA: Analysis of Variance.
ZO-1: Zonula Occludens-1
