# Supplementary figures and images for: Multi-omics reveal the neuroprotective mechanisms of Xinshubao tablet against scopolamine-induced cognitive dysfunction in mice
Source: Front Pharmacol. 2025 Jul 4;16:1596728. doi: 10.3389/fphar.2025.1596728 (PMC12271746; doi:10.3389/fphar.2025.1596728)

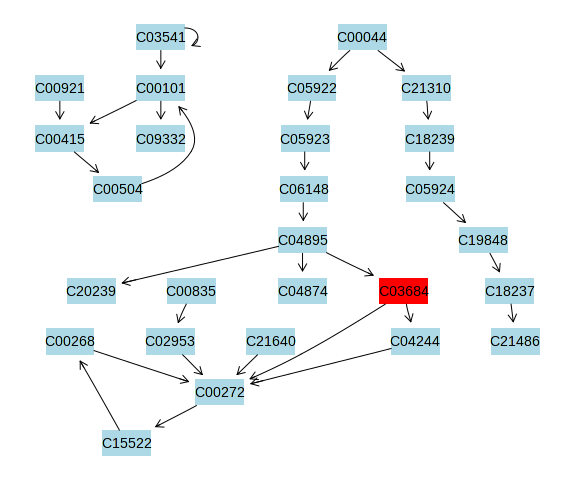

Supplement: Supplementary file 1 [file DataSheet3.zip › Plasma metabolomics analysis/KEGGpathway analysis/Download (2)/Folate biosynthesis.png]

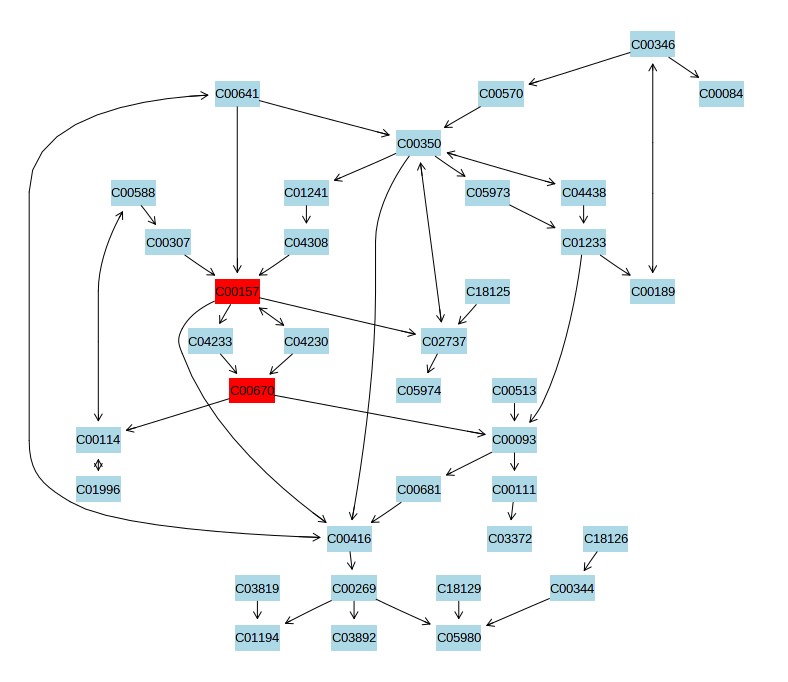

Supplement: Supplementary file 1 [file DataSheet3.zip › Plasma metabolomics analysis/KEGGpathway analysis/Download (2)/Glycerophospholipid metabolism.png]

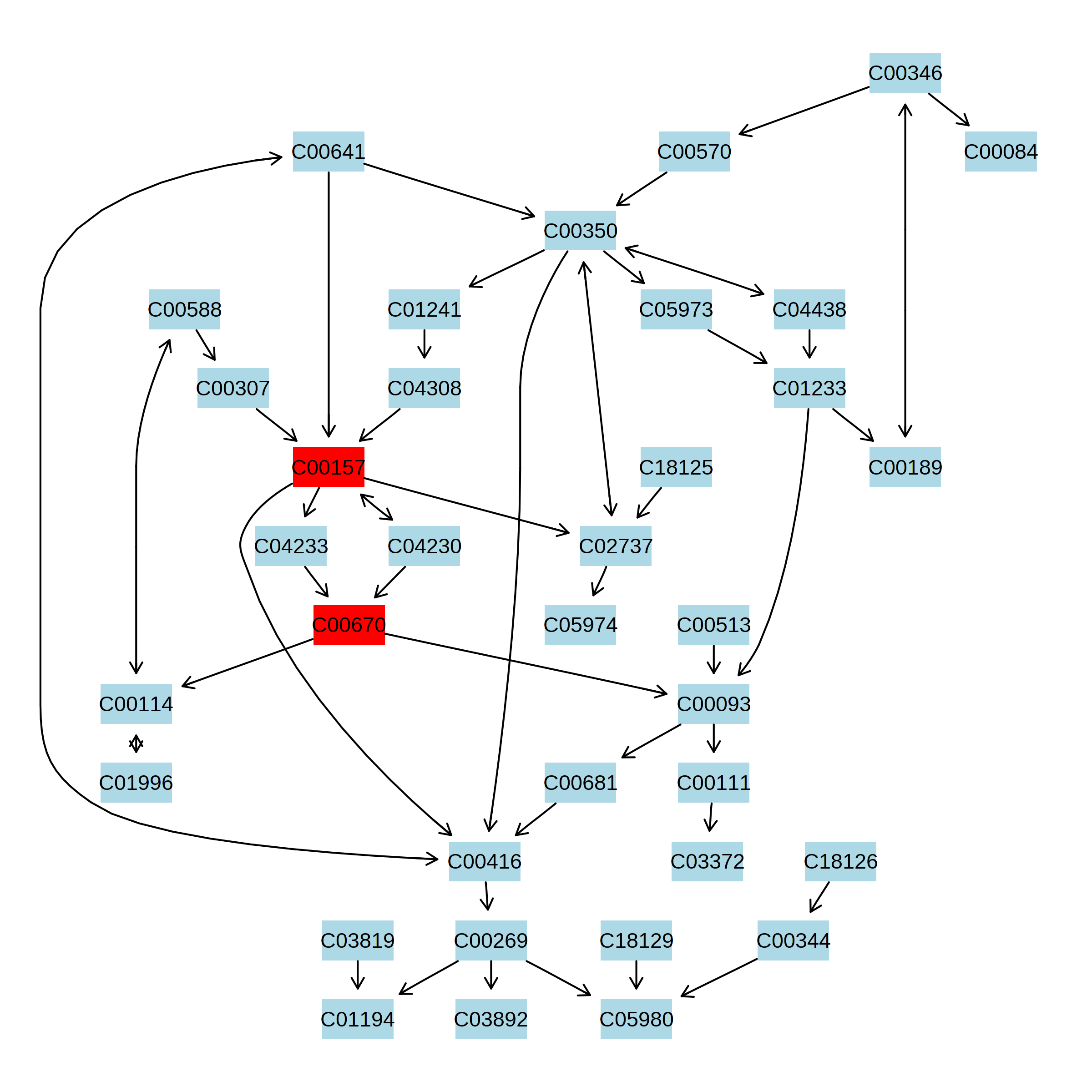

Supplement: Supplementary file 1 [file DataSheet3.zip › Plasma metabolomics analysis/KEGGpathway analysis/Download (2)/Glycerophospholipid_metabolism_dpi300.png]

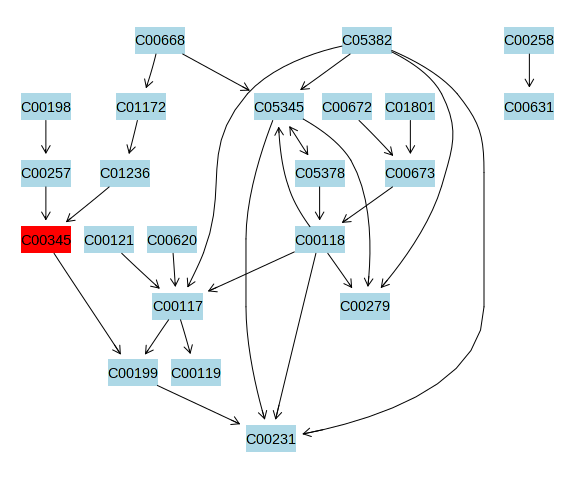

Supplement: Supplementary file 1 [file DataSheet3.zip › Plasma metabolomics analysis/KEGGpathway analysis/Download (2)/Pentose phosphate pathway.png]

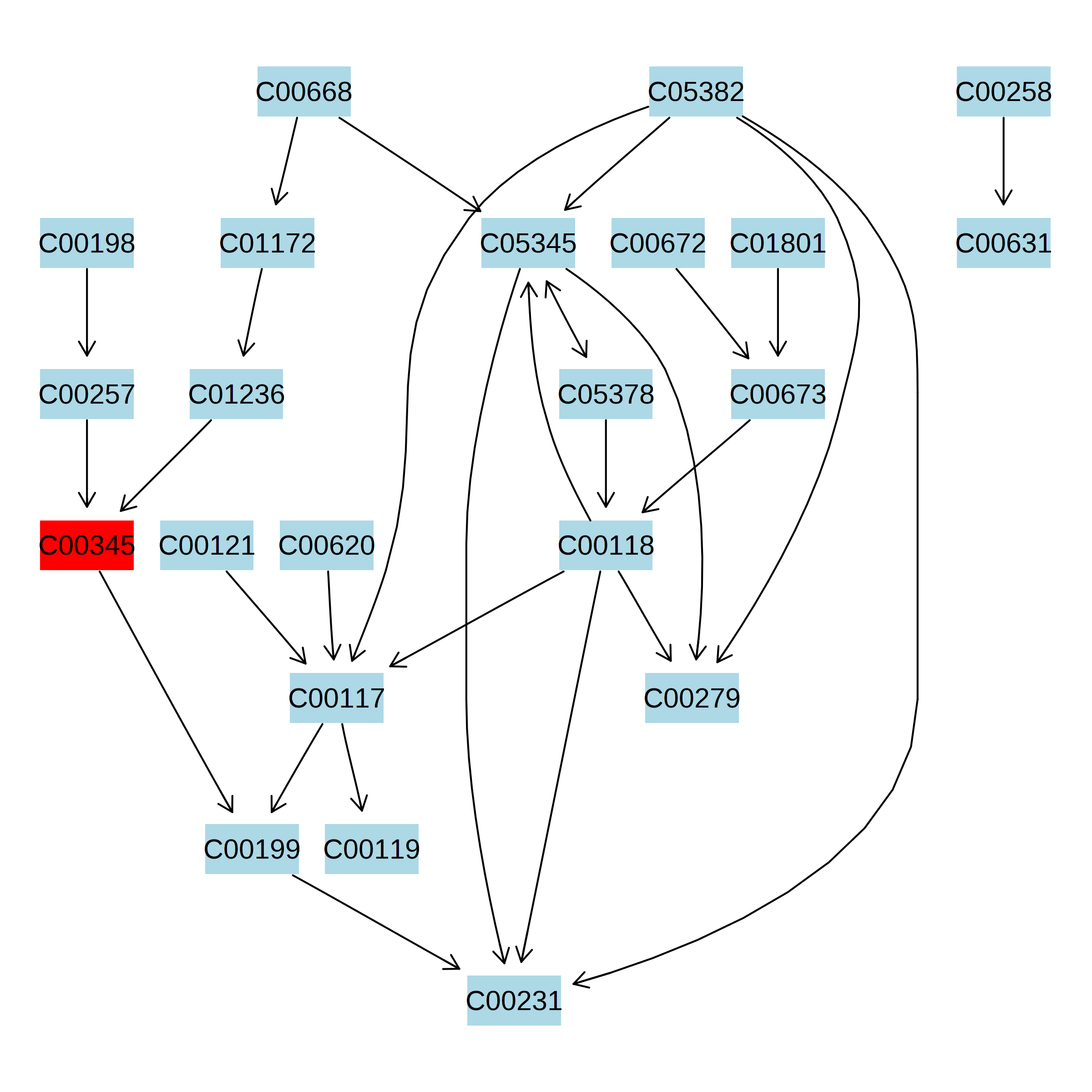

Supplement: Supplementary file 1 [file DataSheet3.zip › Plasma metabolomics analysis/KEGGpathway analysis/Download (2)/Pentose_phosphate_pathway_dpi300.png]

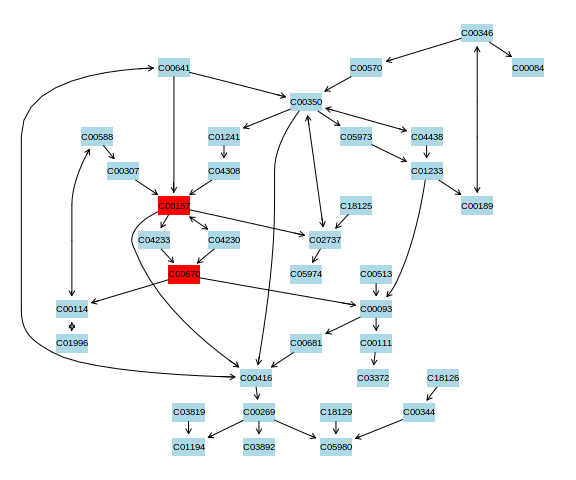

Supplement: Supplementary file 1 [file DataSheet3.zip › Plasma metabolomics analysis/KEGGpathway analysis/Download (2)/crop1687446210604.png]

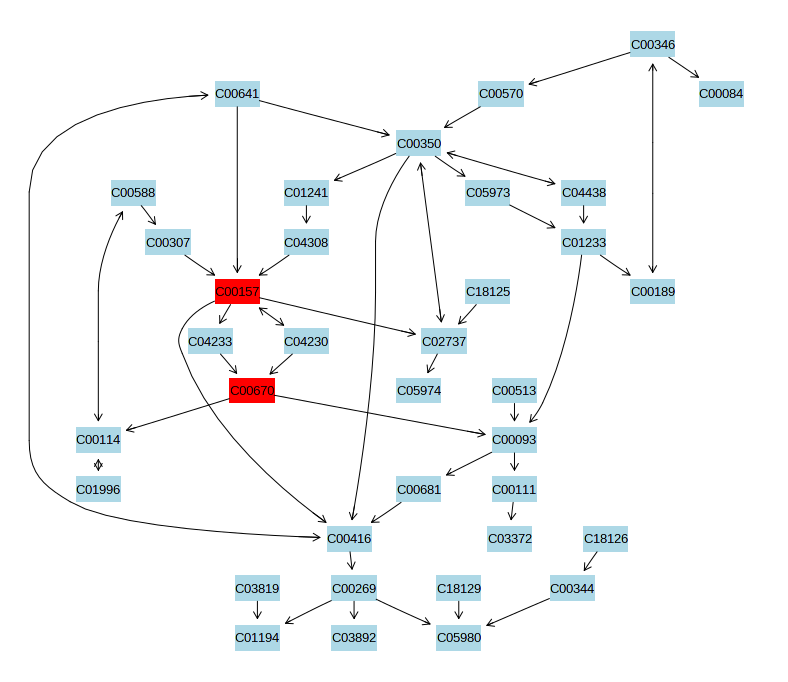

Supplement: Supplementary file 1 [file DataSheet3.zip › Plasma metabolomics analysis/KEGGpathway analysis/Download (2)/crop1687446381480.png]

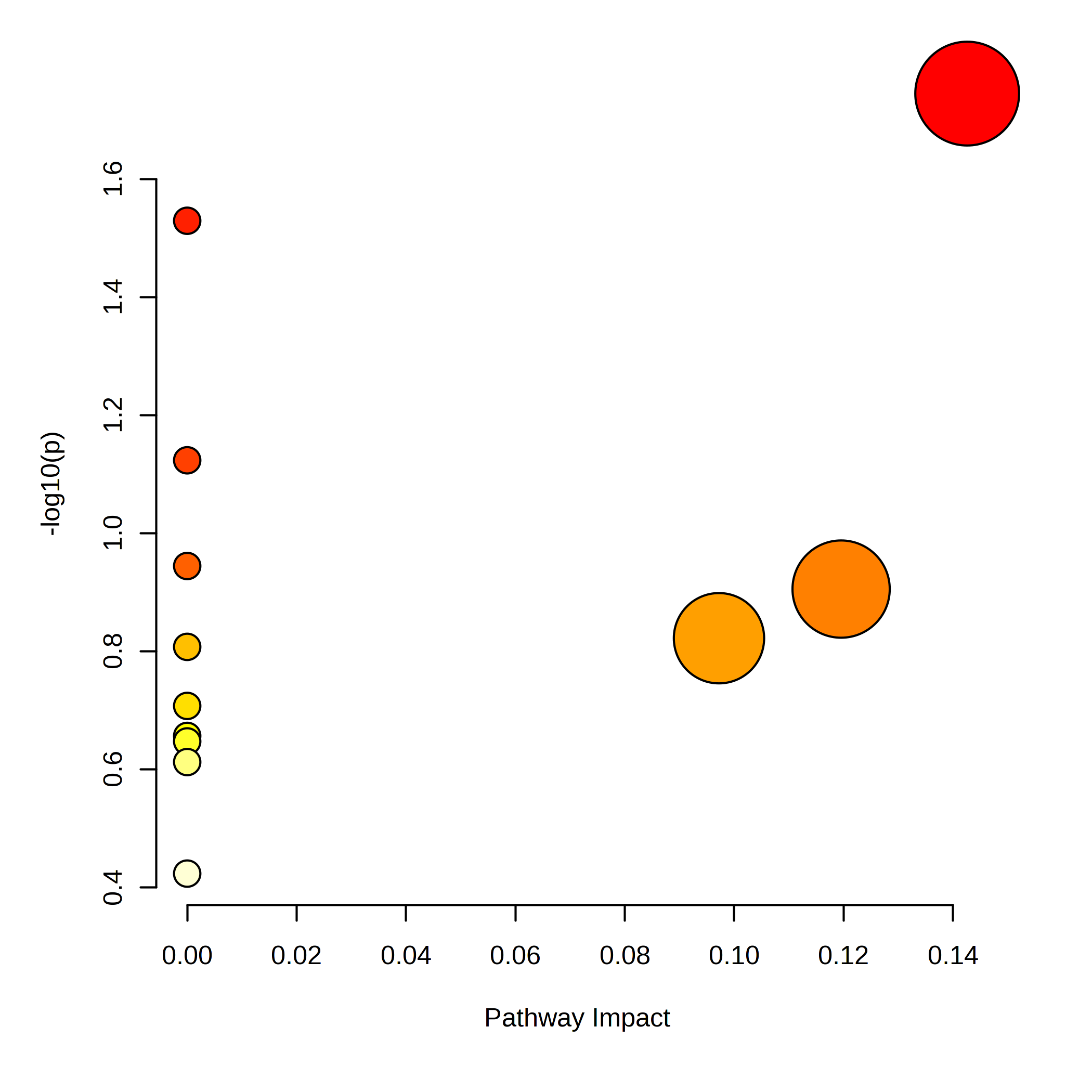

Supplement: Supplementary file 1 [file DataSheet3.zip › Plasma metabolomics analysis/KEGGpathway analysis/Download (2)/path_view_0_dpi300.png]

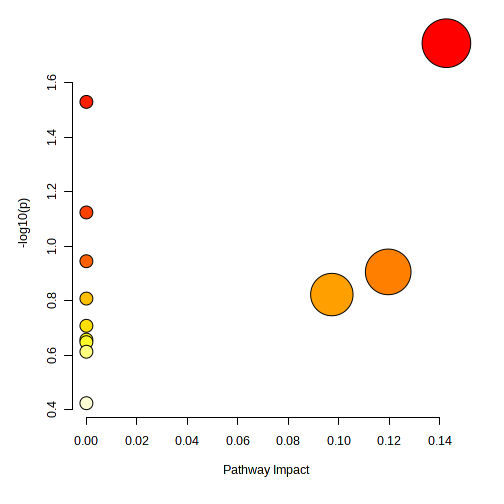

Supplement: Supplementary file 1 [file DataSheet3.zip › Plasma metabolomics analysis/KEGGpathway analysis/Download (2)/path_view_0_dpi72.png]

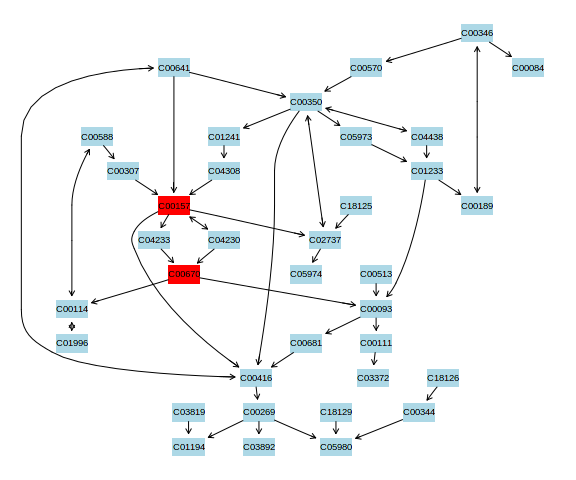

Supplement: Supplementary file 1 [file DataSheet3.zip › Plasma metabolomics analysis/KEGGpathway analysis/Download (2)/zoom1687446210604.png]

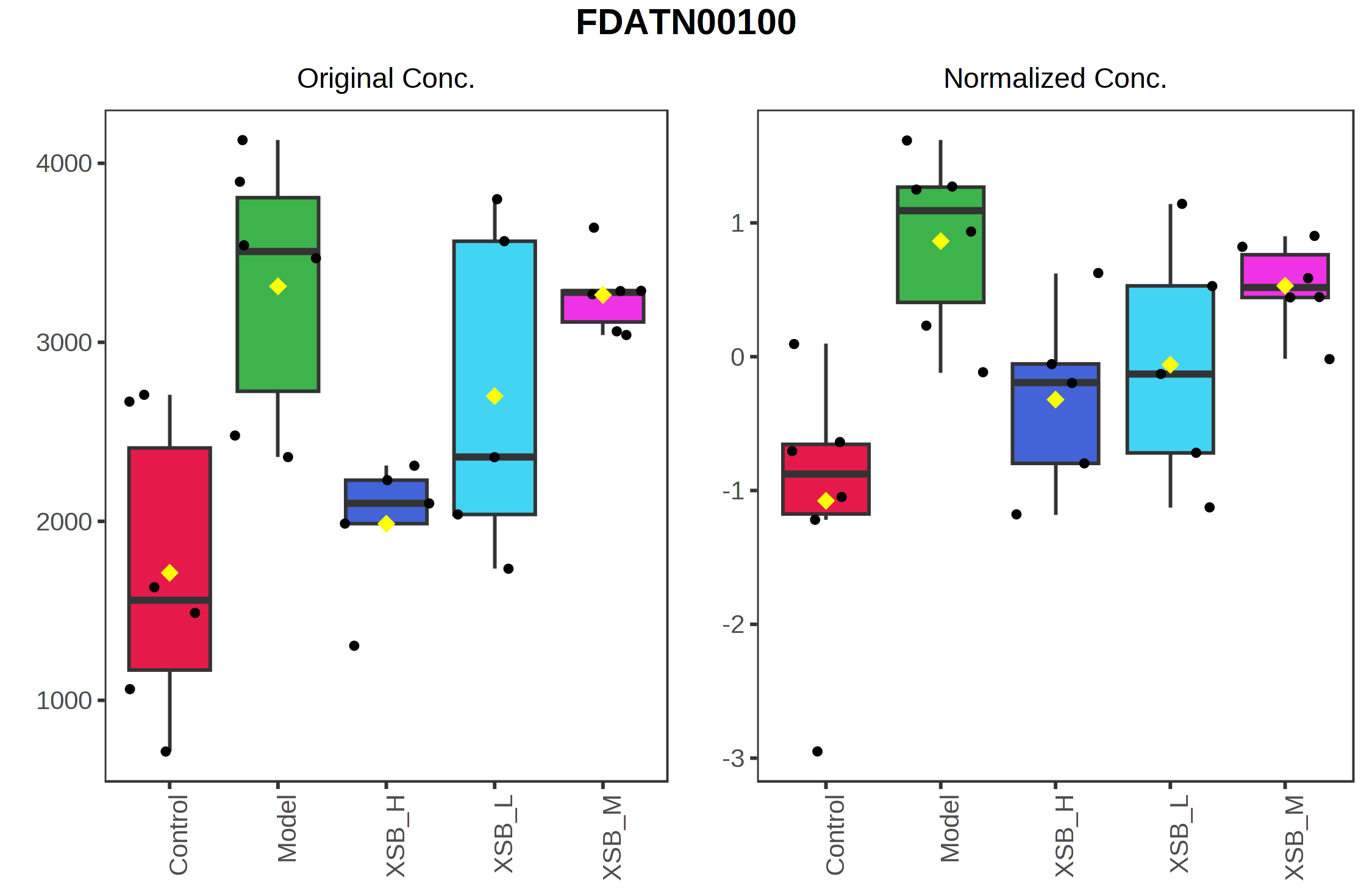

Supplement: Supplementary file 1 [file DataSheet3.zip › Plasma metabolomics analysis/PLS-DA/FDATN00100_100_summary_dpi300.png]

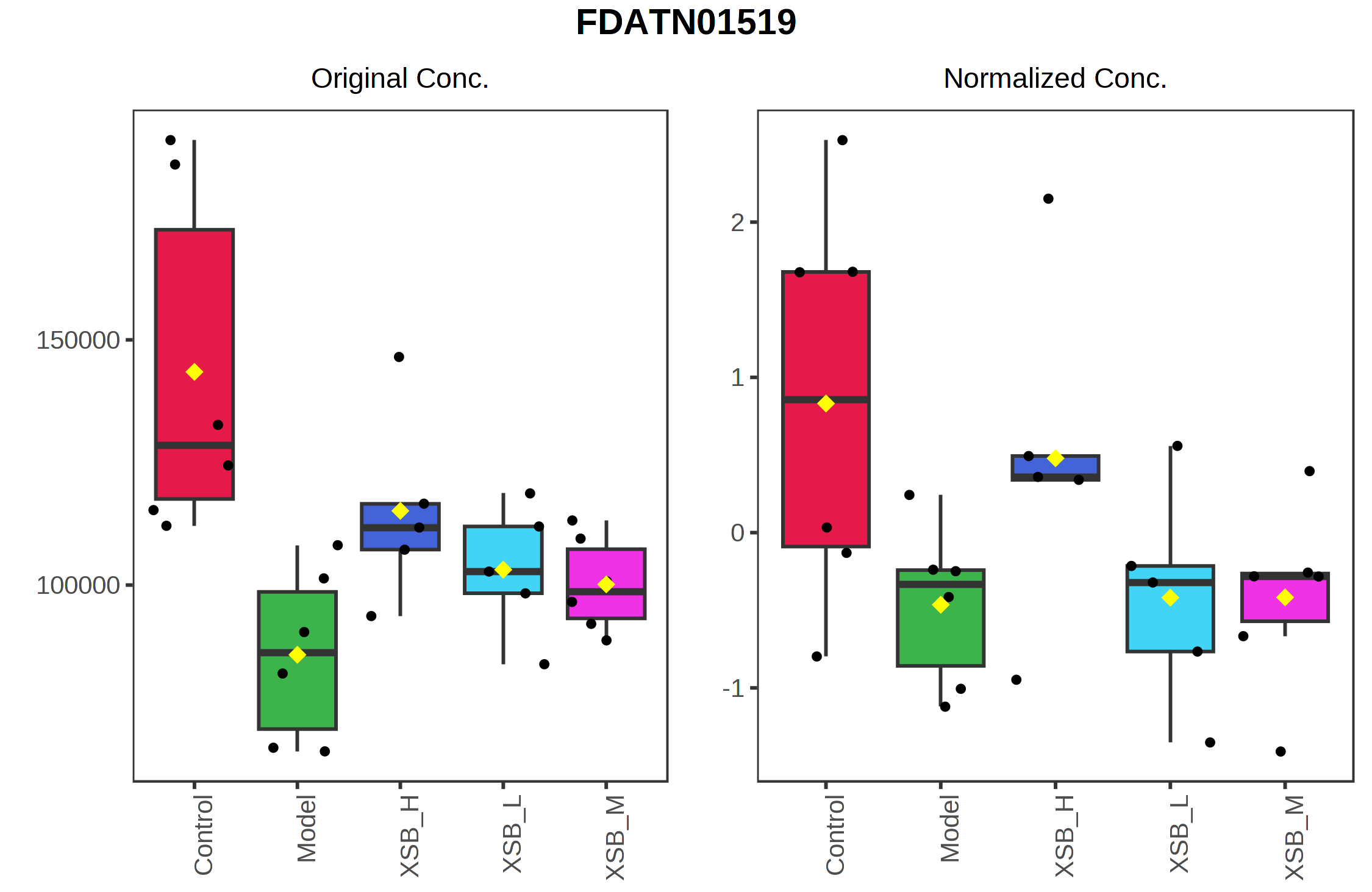

Supplement: Supplementary file 1 [file DataSheet3.zip › Plasma metabolomics analysis/PLS-DA/FDATN01519_100_summary_dpi300.png]

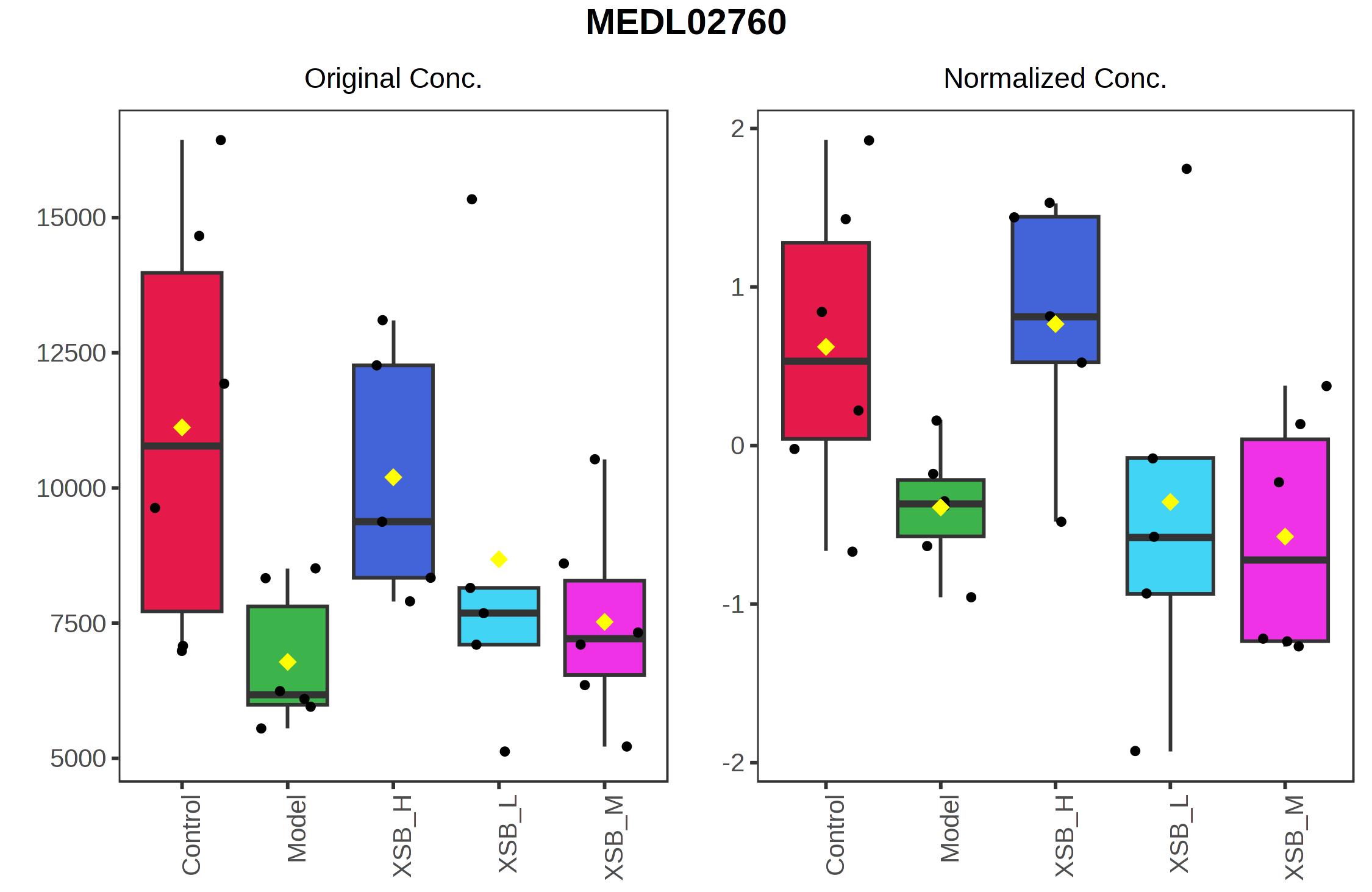

Supplement: Supplementary file 1 [file DataSheet3.zip › Plasma metabolomics analysis/PLS-DA/MEDL02760_100_summary_dpi300.png]

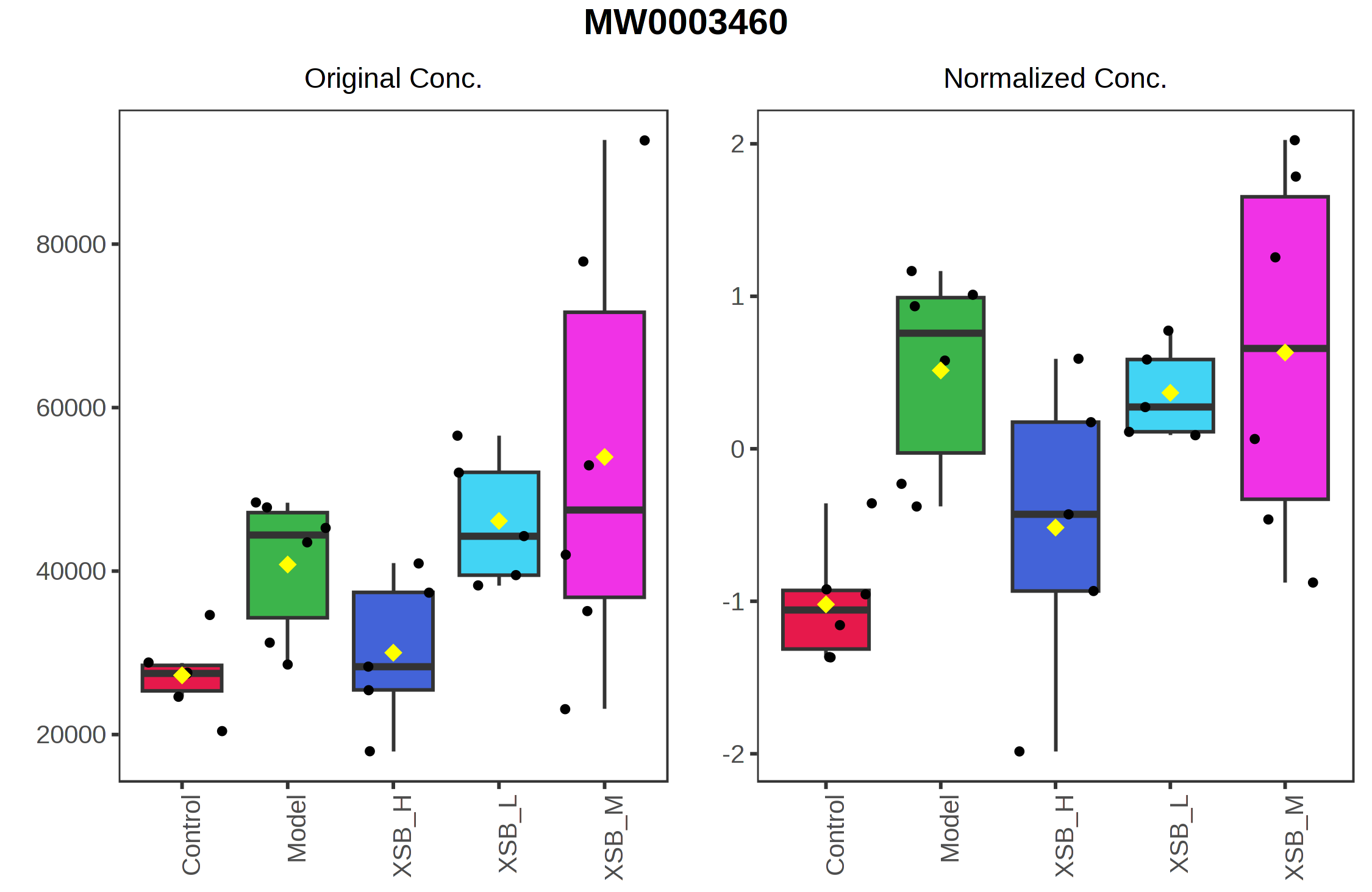

Supplement: Supplementary file 1 [file DataSheet3.zip › Plasma metabolomics analysis/PLS-DA/MW0003460_100_summary_dpi300.png]

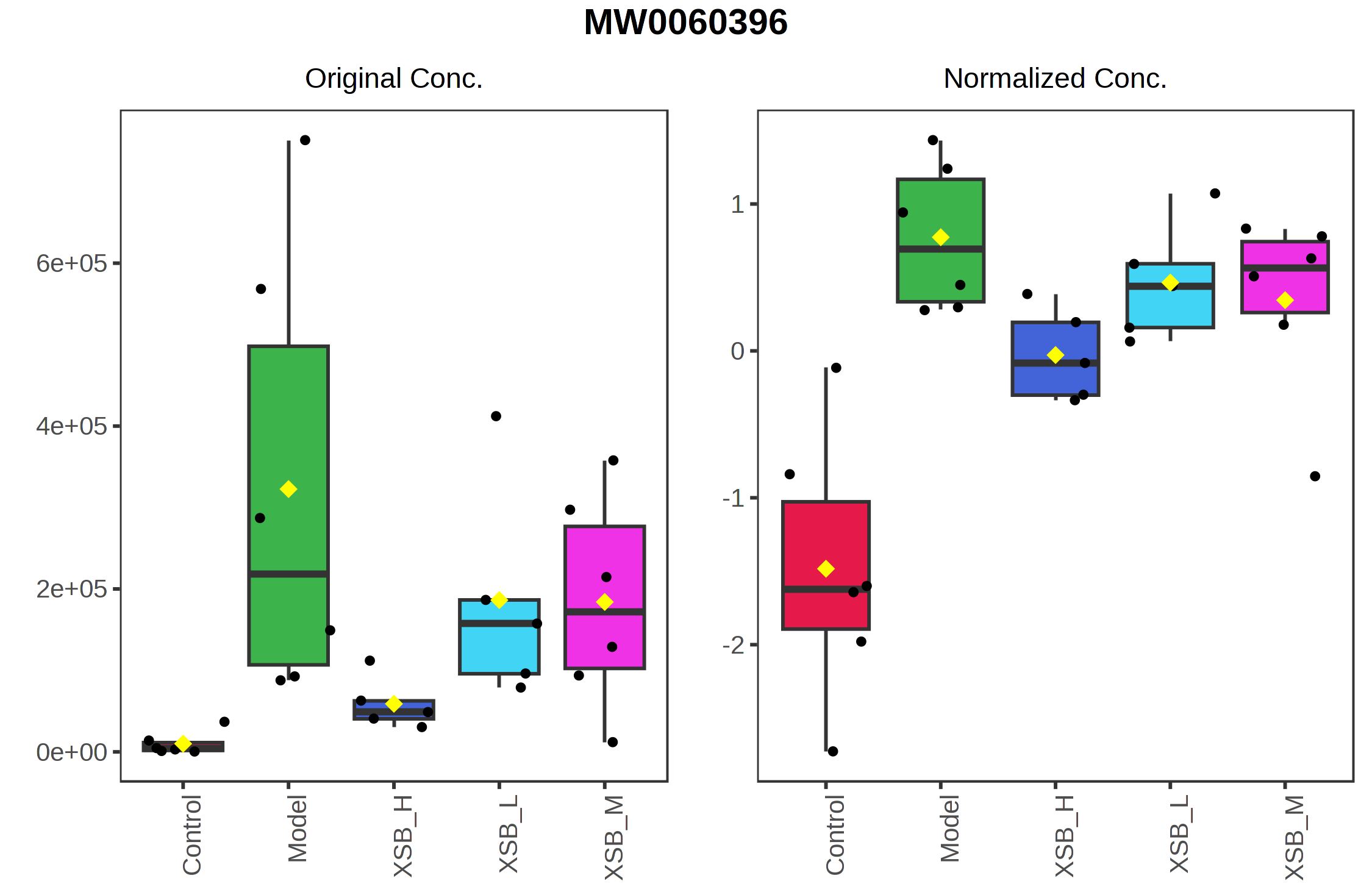

Supplement: Supplementary file 1 [file DataSheet3.zip › Plasma metabolomics analysis/PLS-DA/MW0060396_100_summary_dpi300.png]

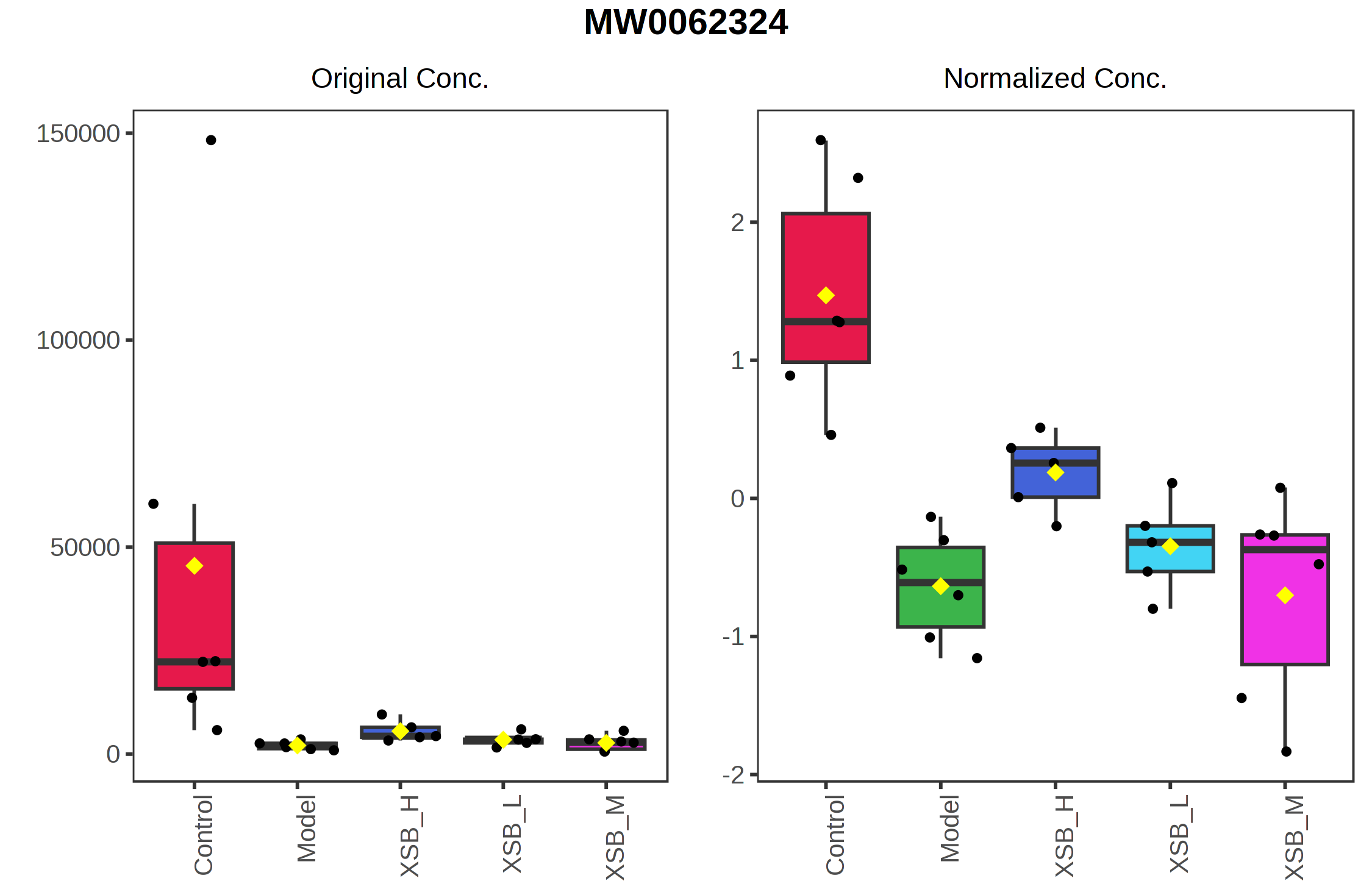

Supplement: Supplementary file 1 [file DataSheet3.zip › Plasma metabolomics analysis/PLS-DA/MW0062324_100_summary_dpi300.png]

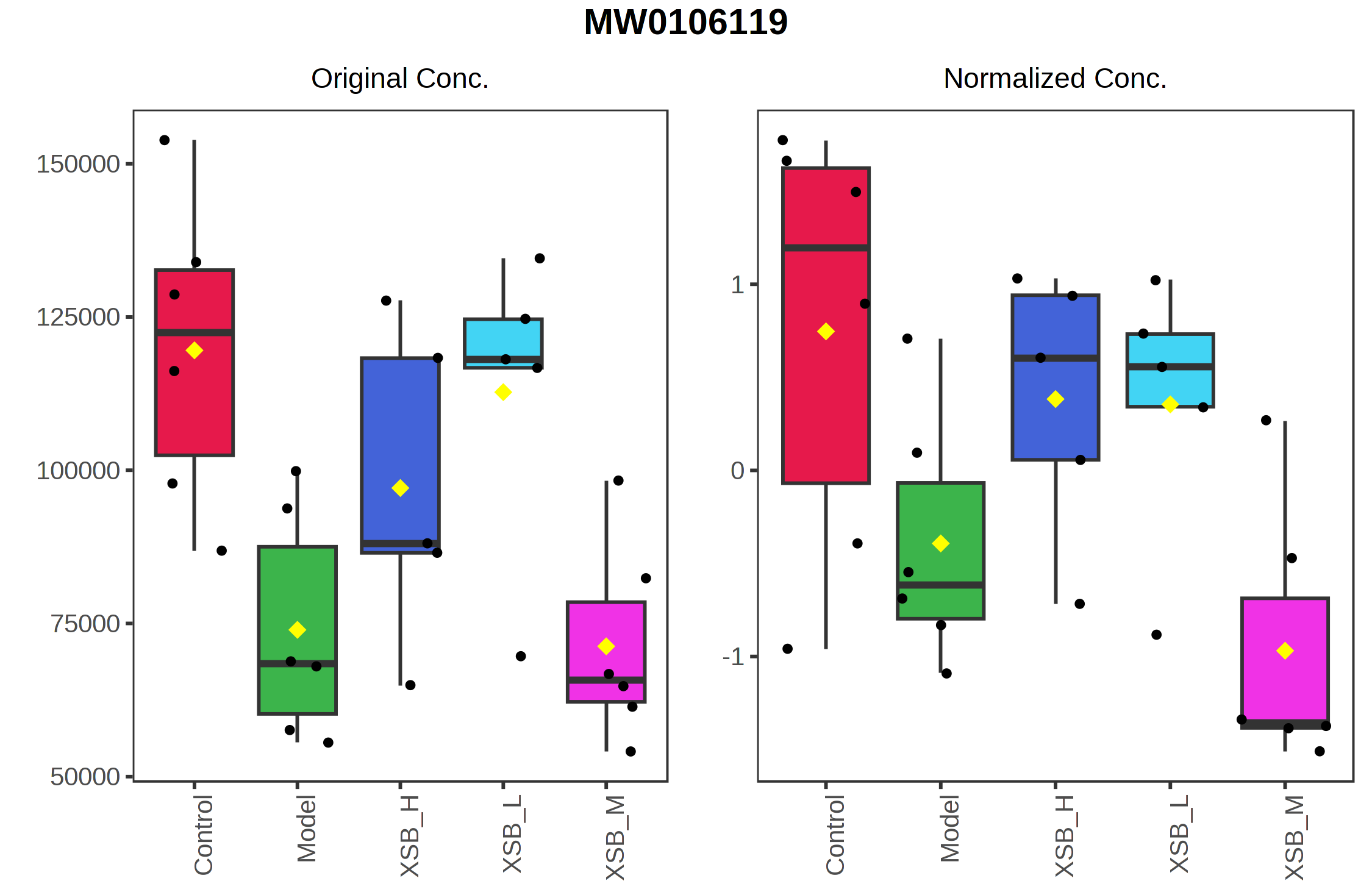

Supplement: Supplementary file 1 [file DataSheet3.zip › Plasma metabolomics analysis/PLS-DA/MW0106119_100_summary_dpi300.png]

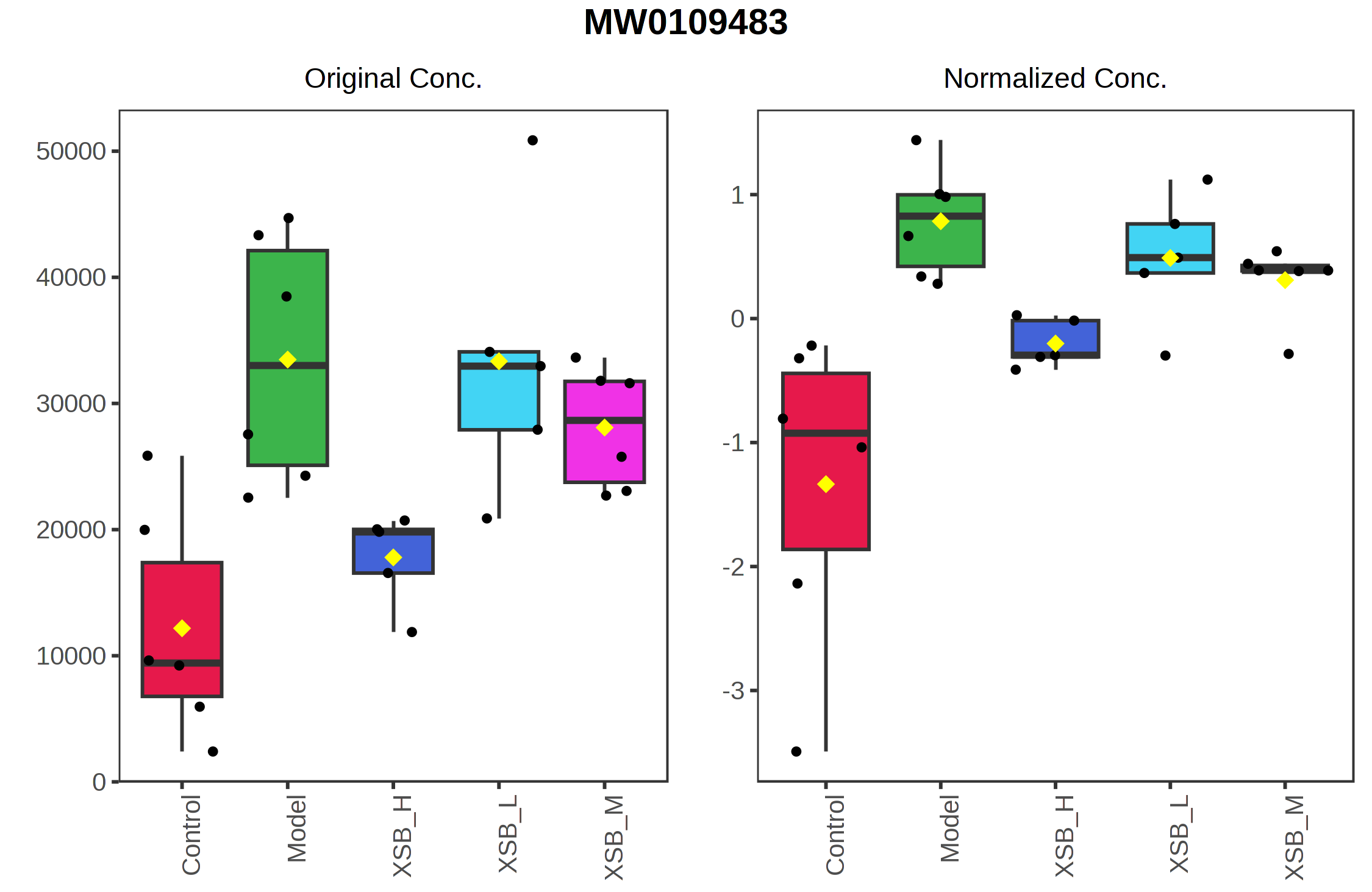

Supplement: Supplementary file 1 [file DataSheet3.zip › Plasma metabolomics analysis/PLS-DA/MW0109483_100_summary_dpi300.png]

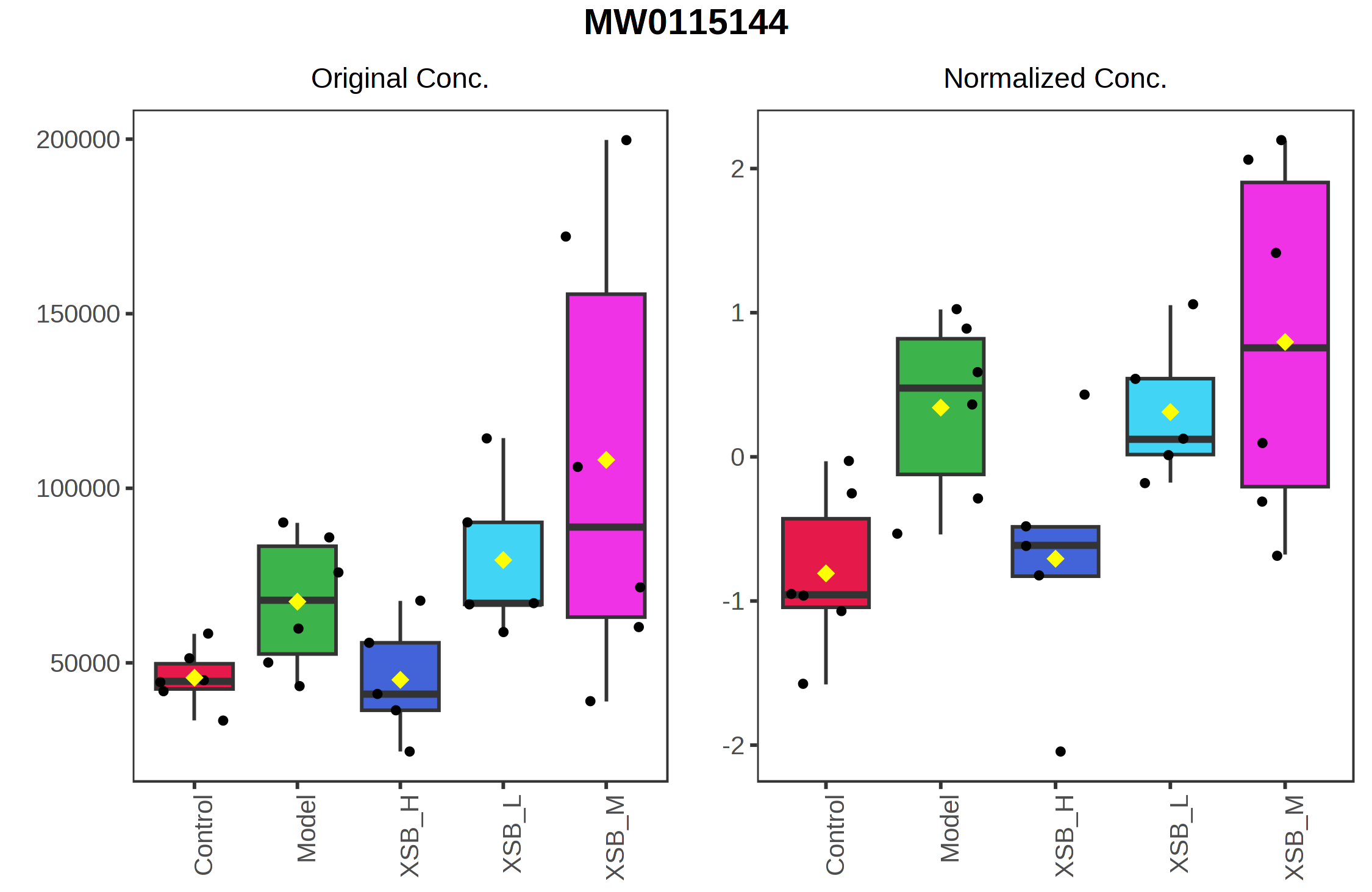

Supplement: Supplementary file 1 [file DataSheet3.zip › Plasma metabolomics analysis/PLS-DA/MW0115144_100_summary_dpi300.png]

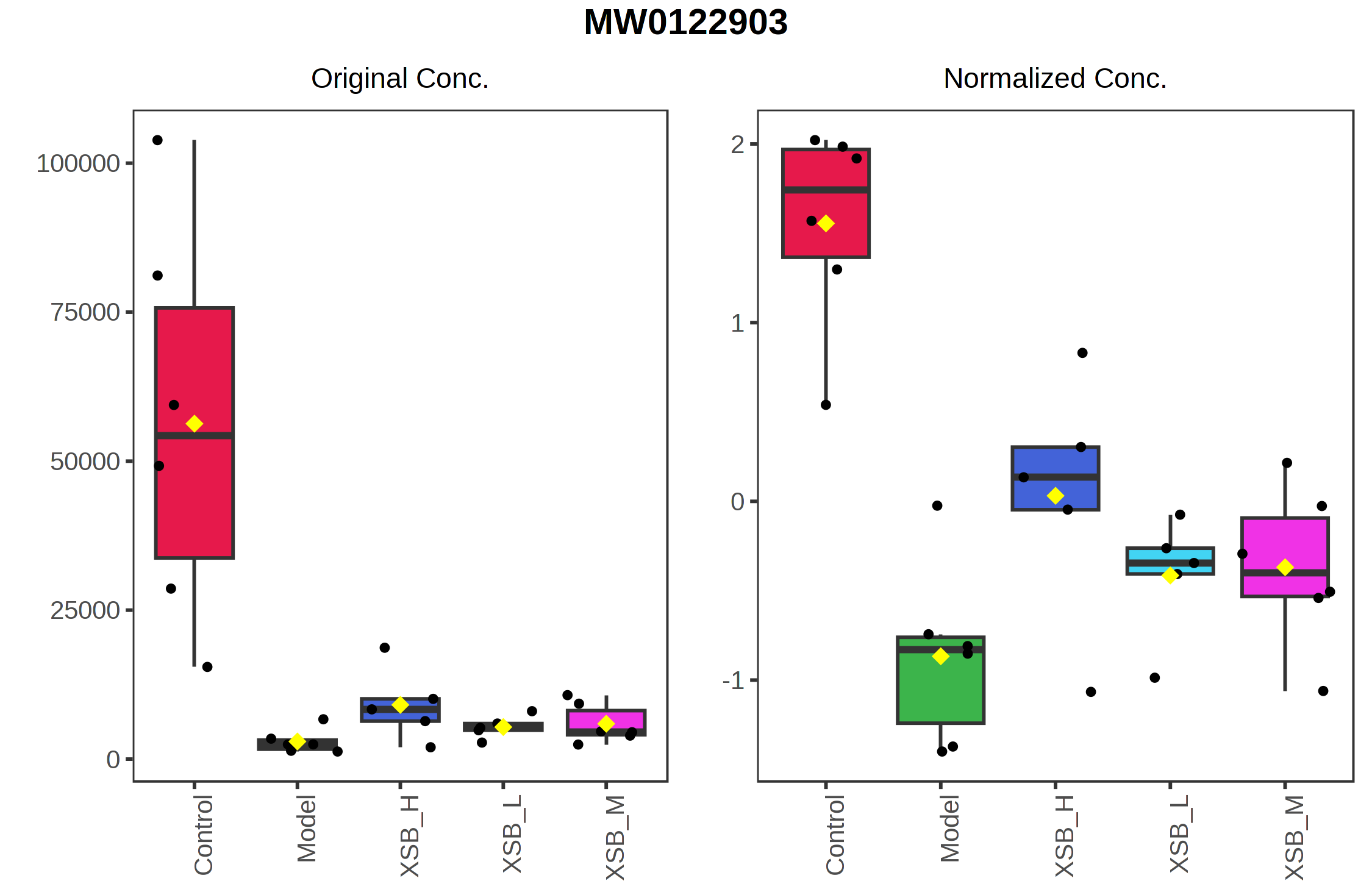

Supplement: Supplementary file 1 [file DataSheet3.zip › Plasma metabolomics analysis/PLS-DA/MW0122903_100_summary_dpi300.png]

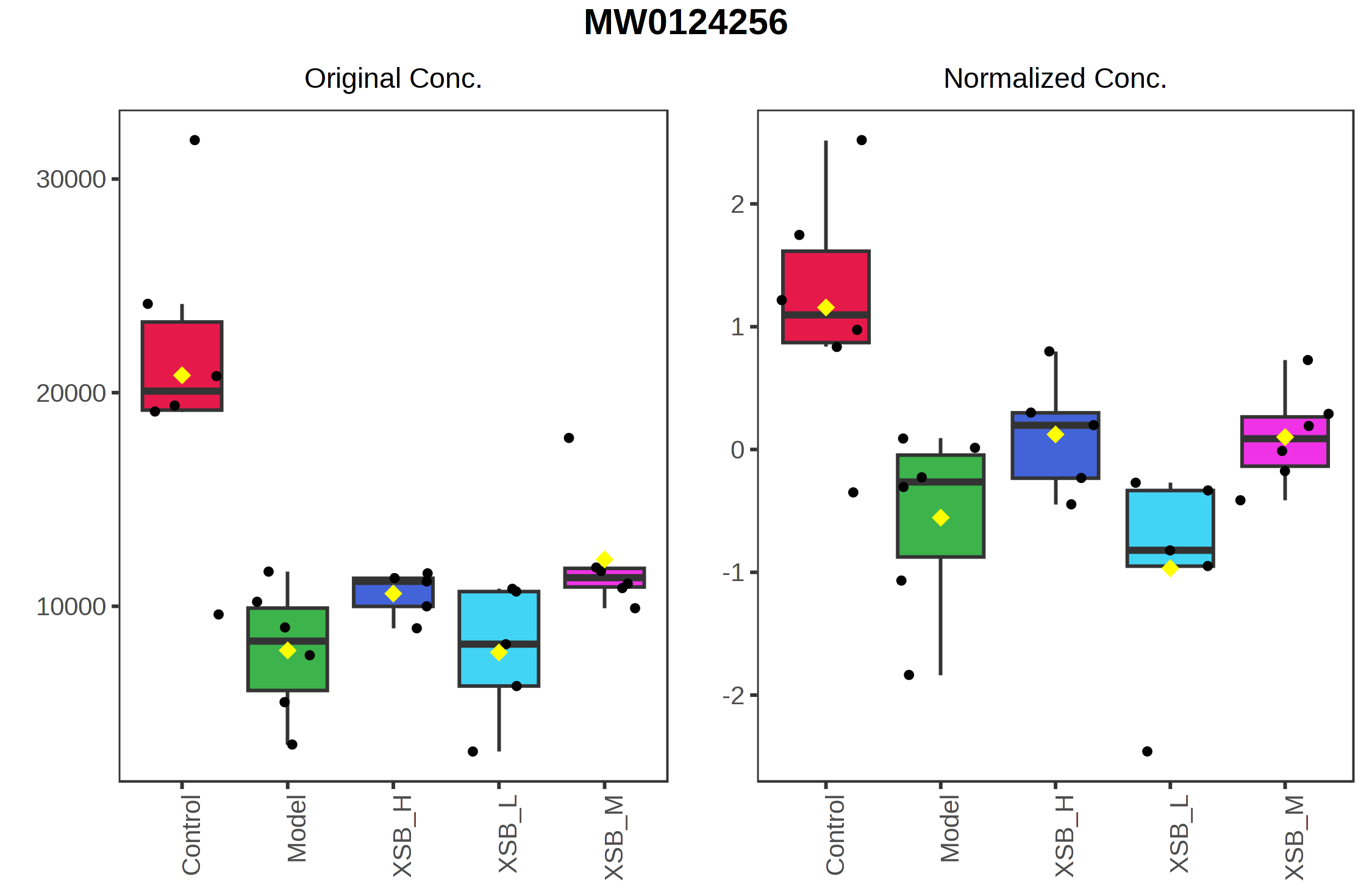

Supplement: Supplementary file 1 [file DataSheet3.zip › Plasma metabolomics analysis/PLS-DA/MW0124256_100_summary_dpi300.png]

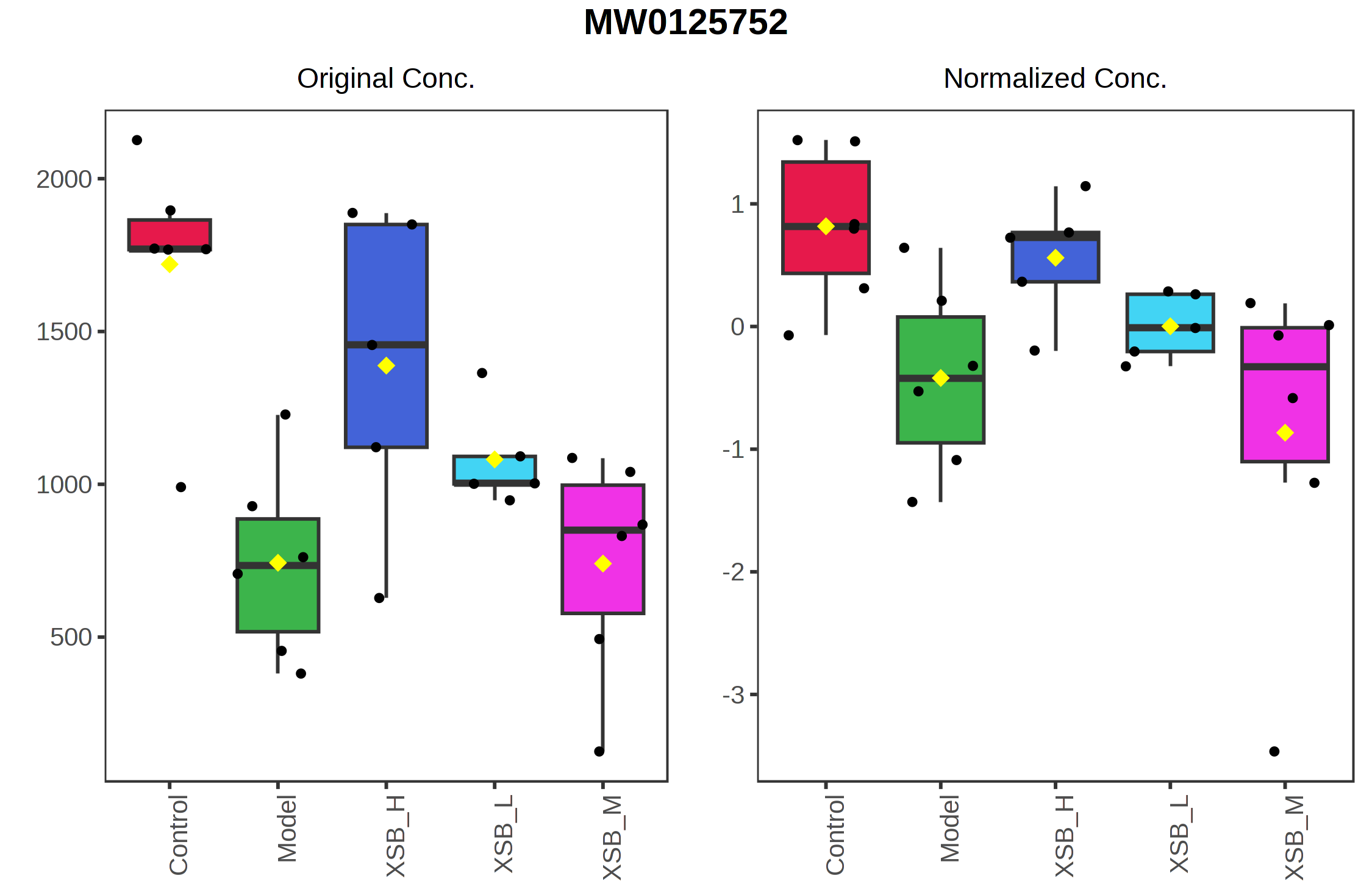

Supplement: Supplementary file 1 [file DataSheet3.zip › Plasma metabolomics analysis/PLS-DA/MW0125752_100_summary_dpi300.png]

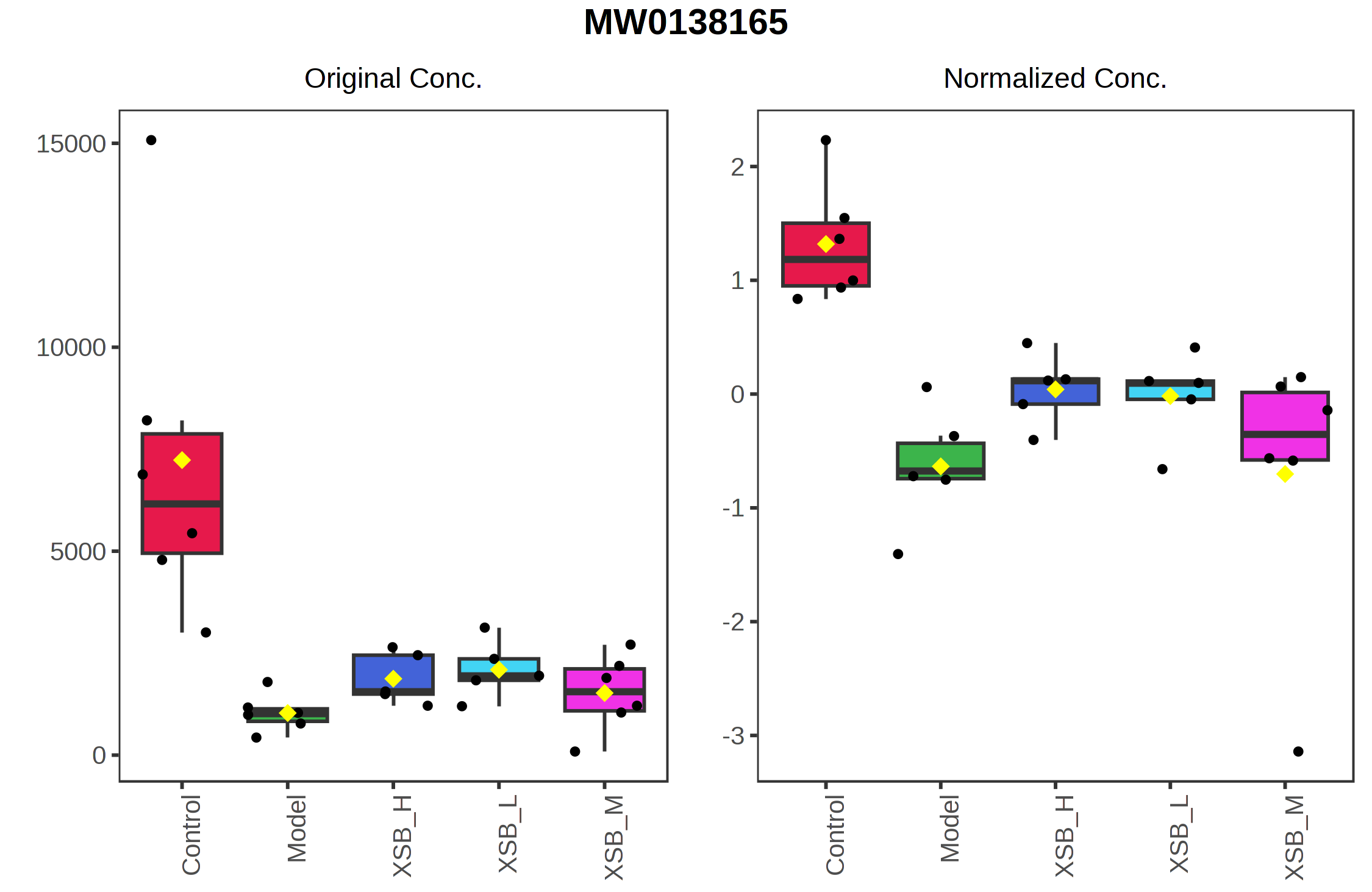

Supplement: Supplementary file 1 [file DataSheet3.zip › Plasma metabolomics analysis/PLS-DA/MW0138165_100_summary_dpi300.png]

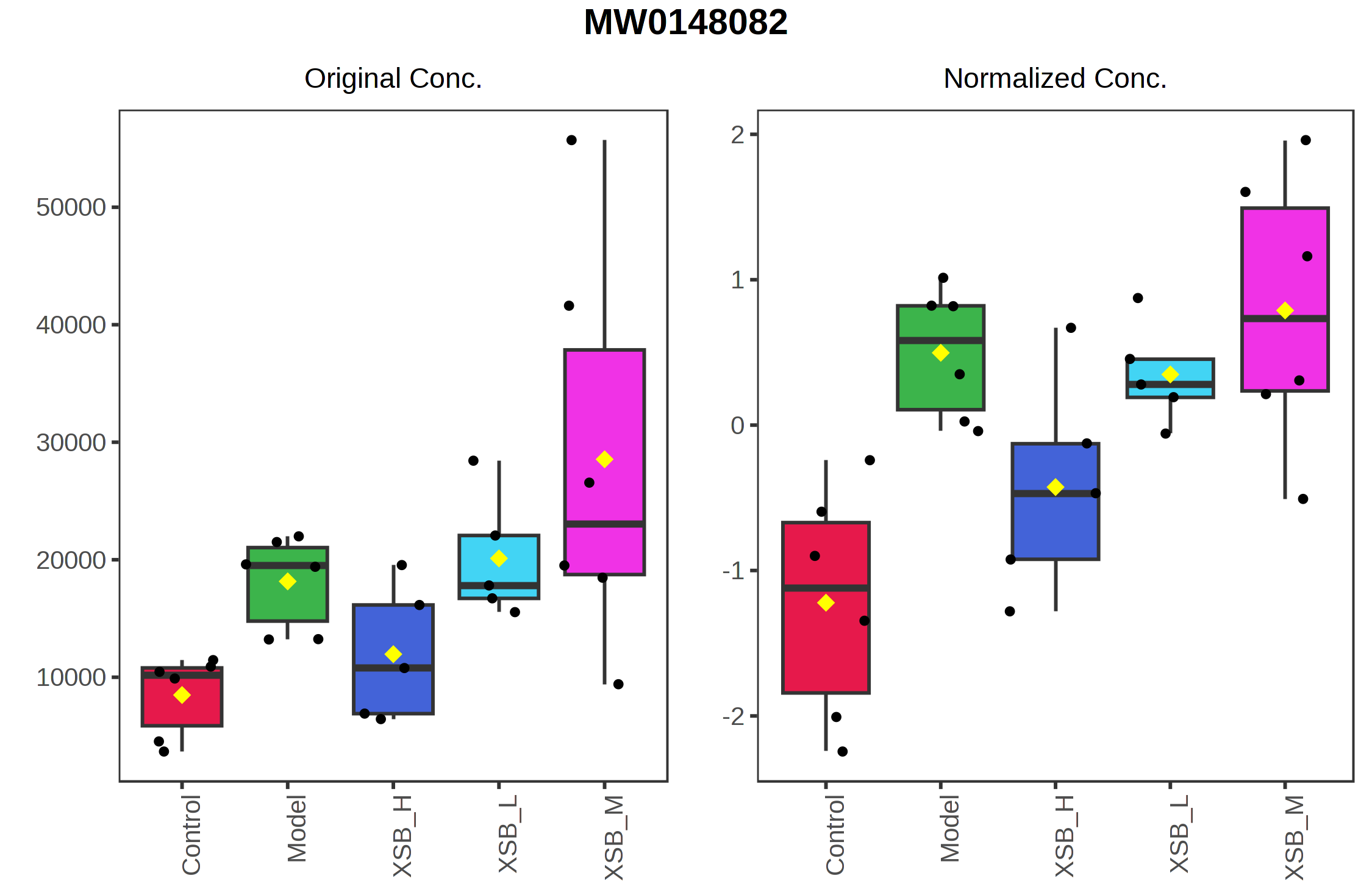

Supplement: Supplementary file 1 [file DataSheet3.zip › Plasma metabolomics analysis/PLS-DA/MW0148082_100_summary_dpi300.png]

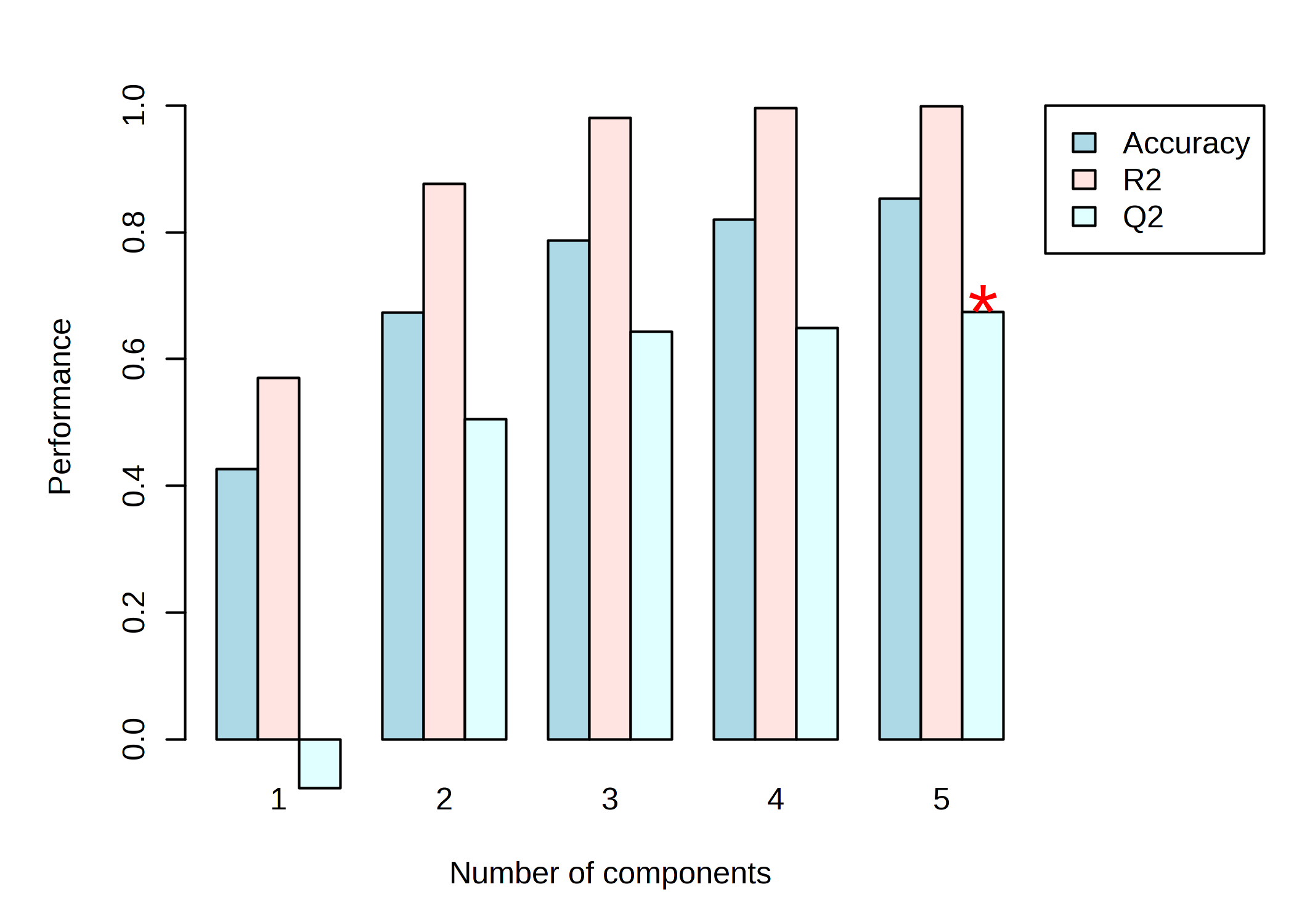

Supplement: Supplementary file 1 [file DataSheet3.zip › Plasma metabolomics analysis/PLS-DA/pls_cv_1_dpi300.png]

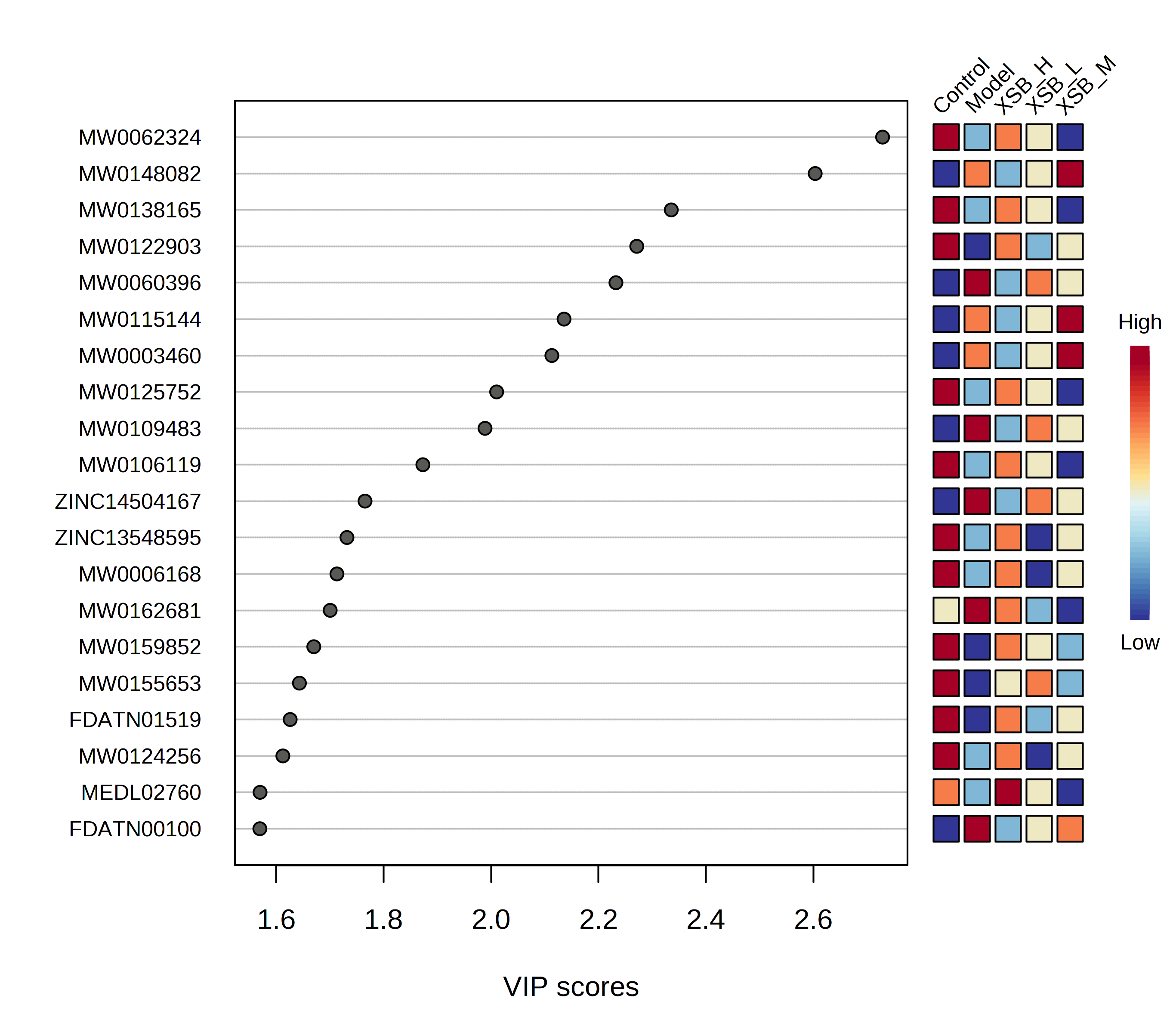

Supplement: Supplementary file 1 [file DataSheet3.zip › Plasma metabolomics analysis/PLS-DA/pls_imp_2_dpi300.png]

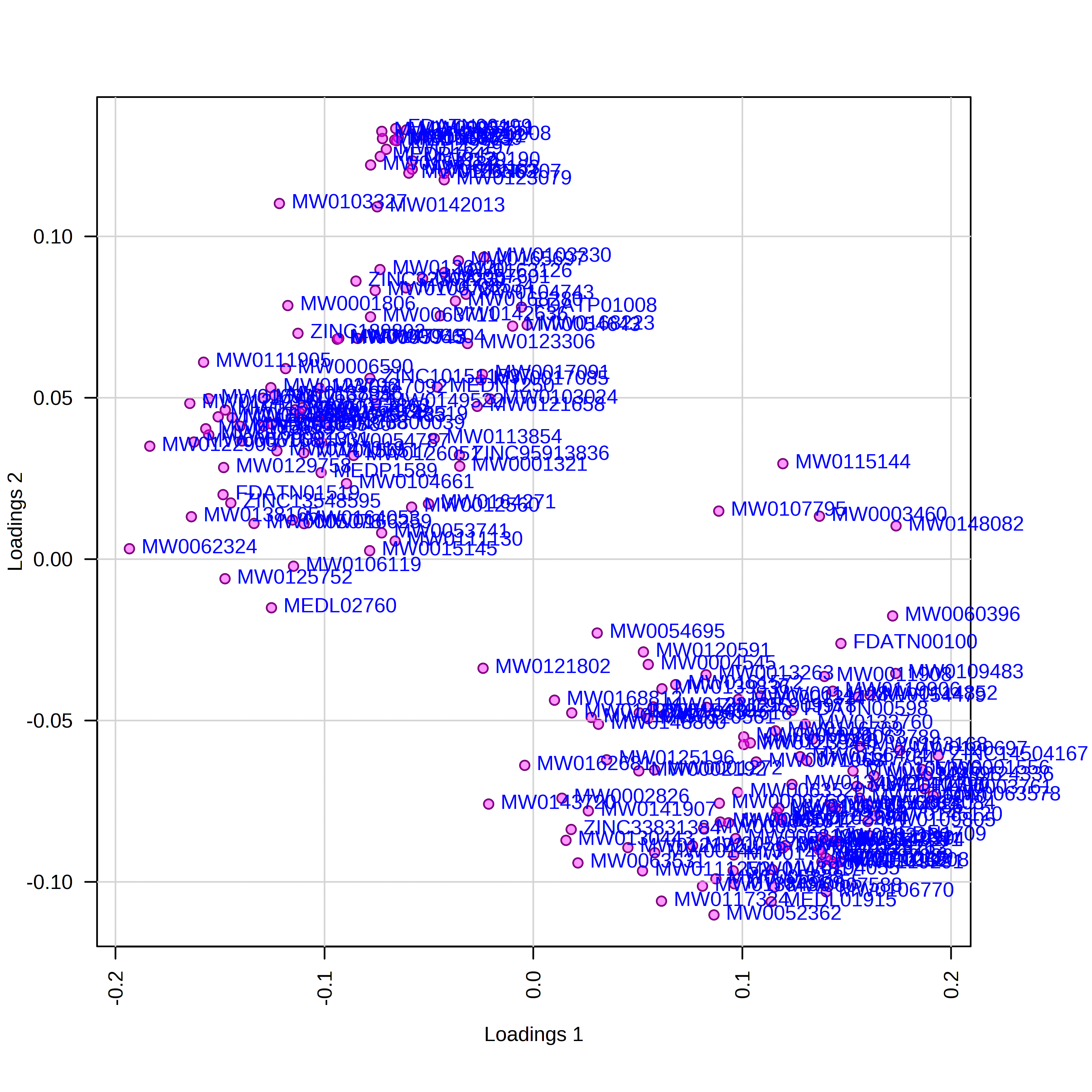

Supplement: Supplementary file 1 [file DataSheet3.zip › Plasma metabolomics analysis/PLS-DA/pls_loading_0_dpi300.png]

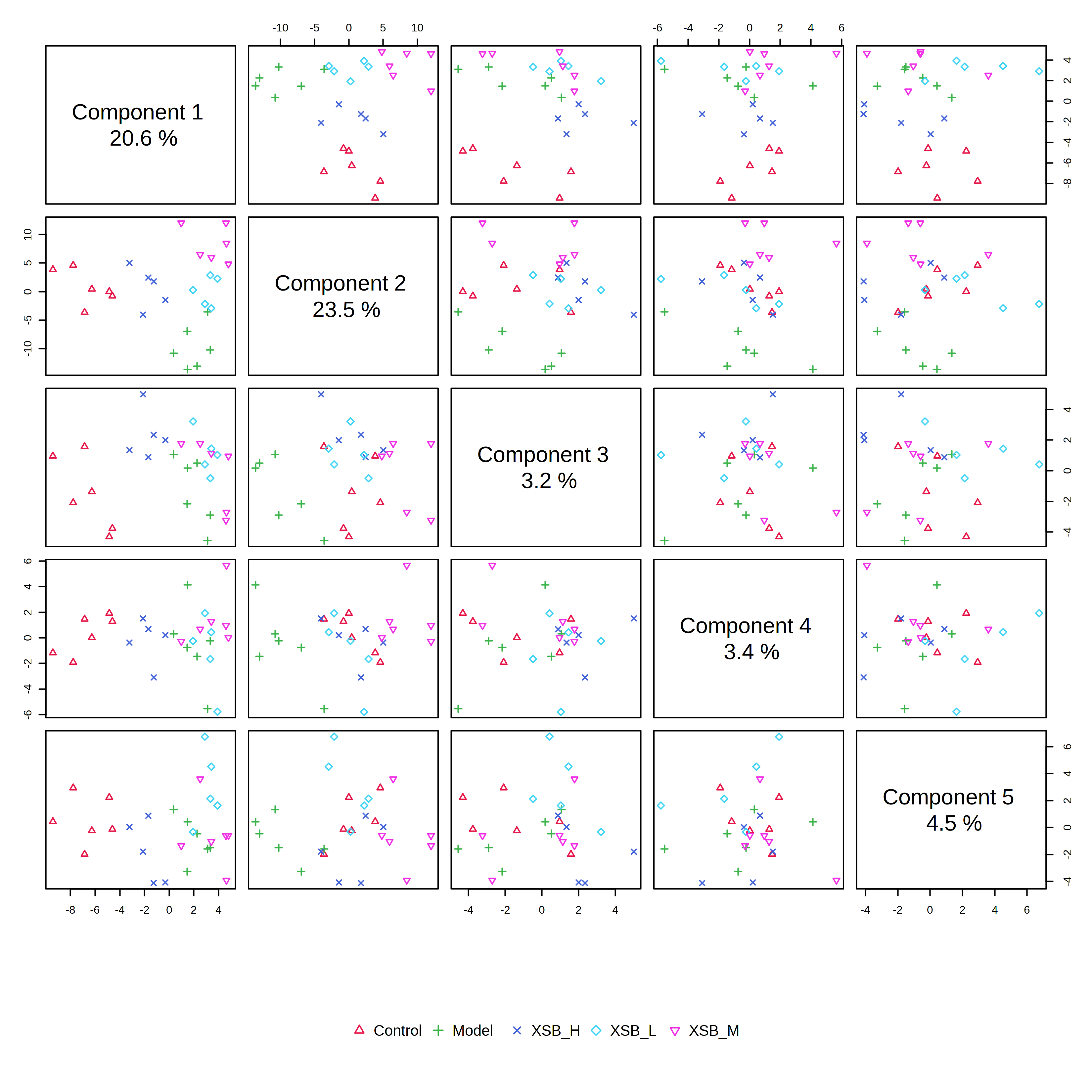

Supplement: Supplementary file 1 [file DataSheet3.zip › Plasma metabolomics analysis/PLS-DA/pls_pair_0_dpi300.png]

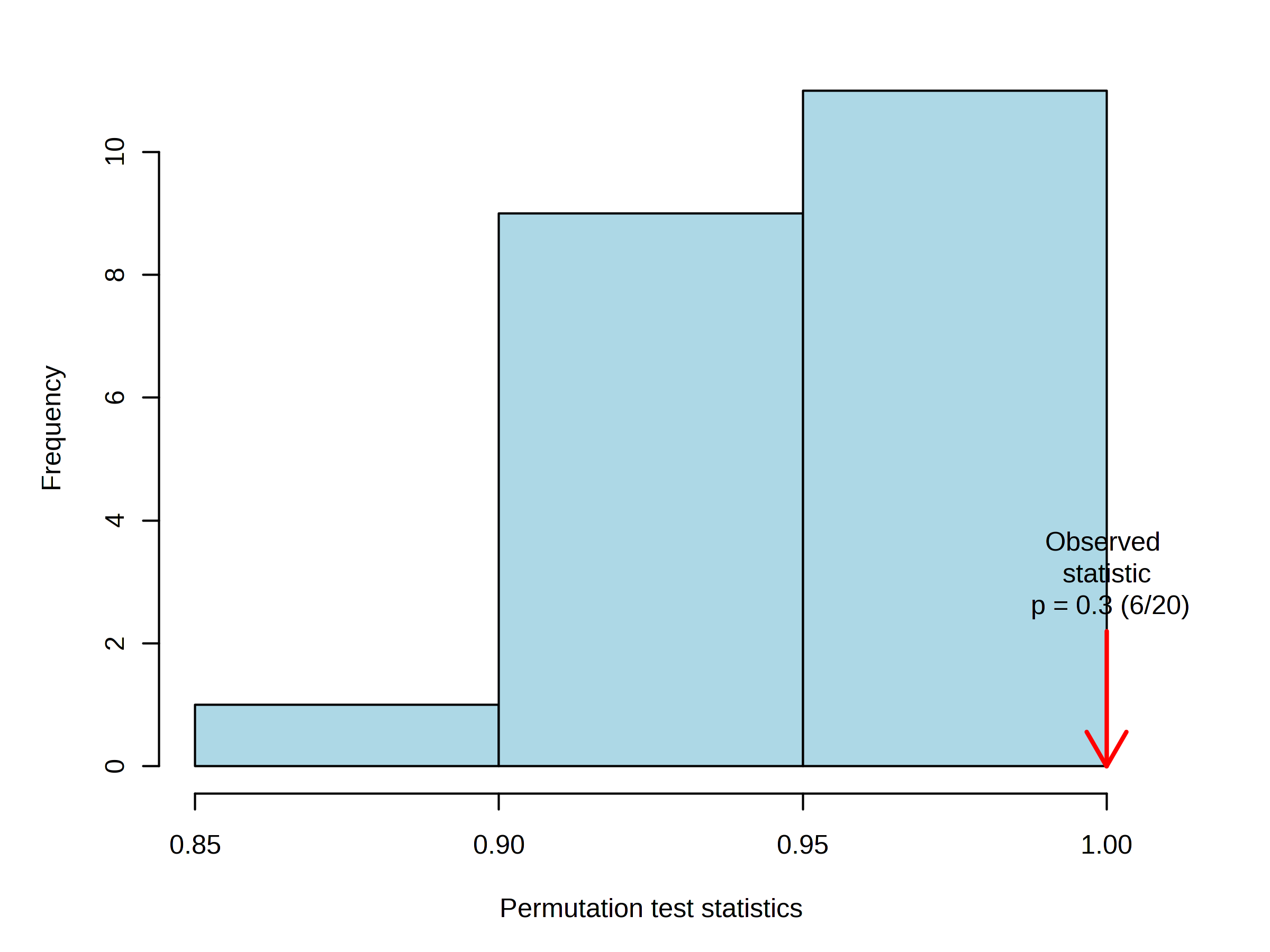

Supplement: Supplementary file 1 [file DataSheet3.zip › Plasma metabolomics analysis/PLS-DA/pls_perm_2_dpi300.png]

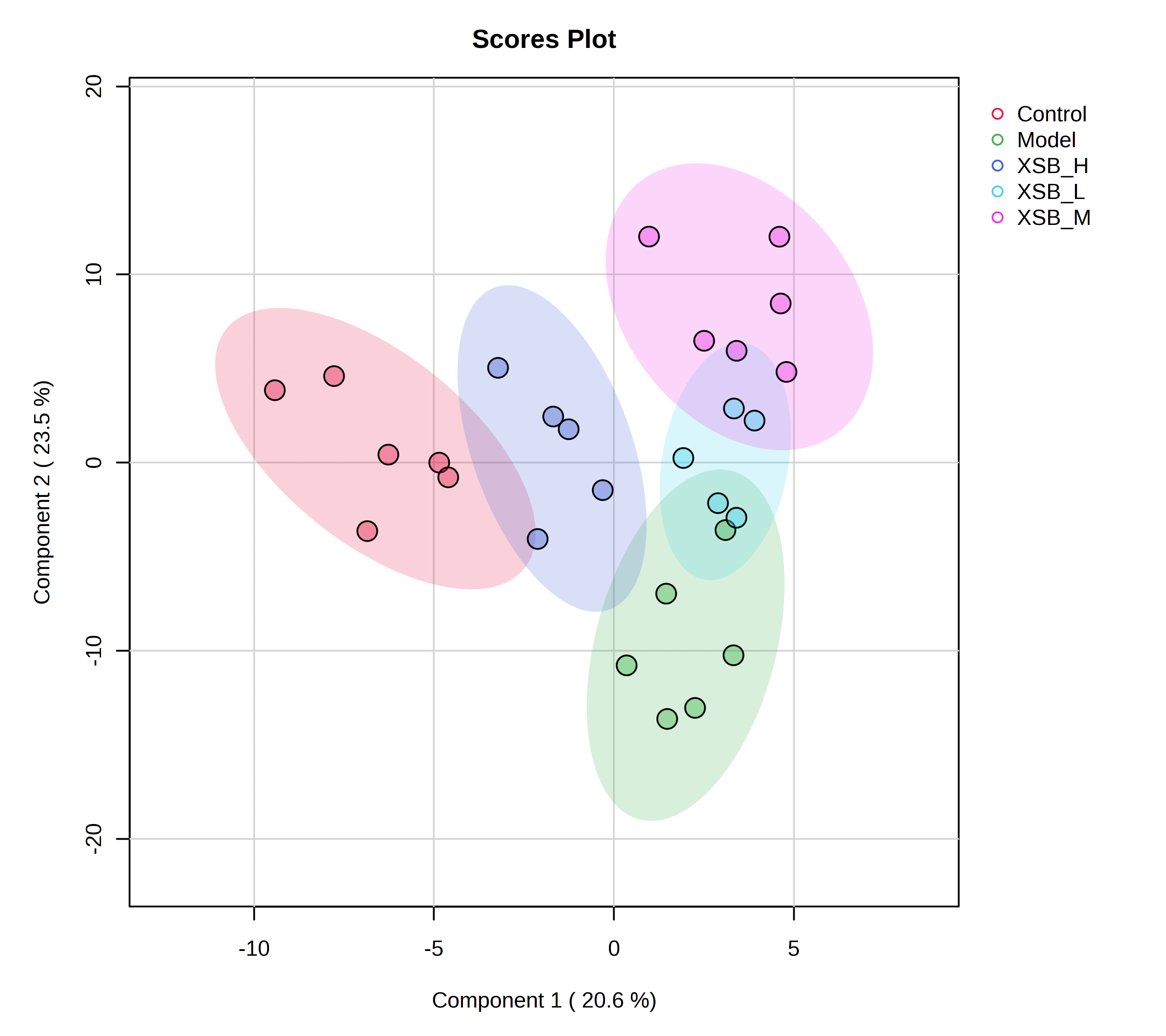

Supplement: Supplementary file 1 [file DataSheet3.zip › Plasma metabolomics analysis/PLS-DA/pls_score2d_0_dpi300.png]

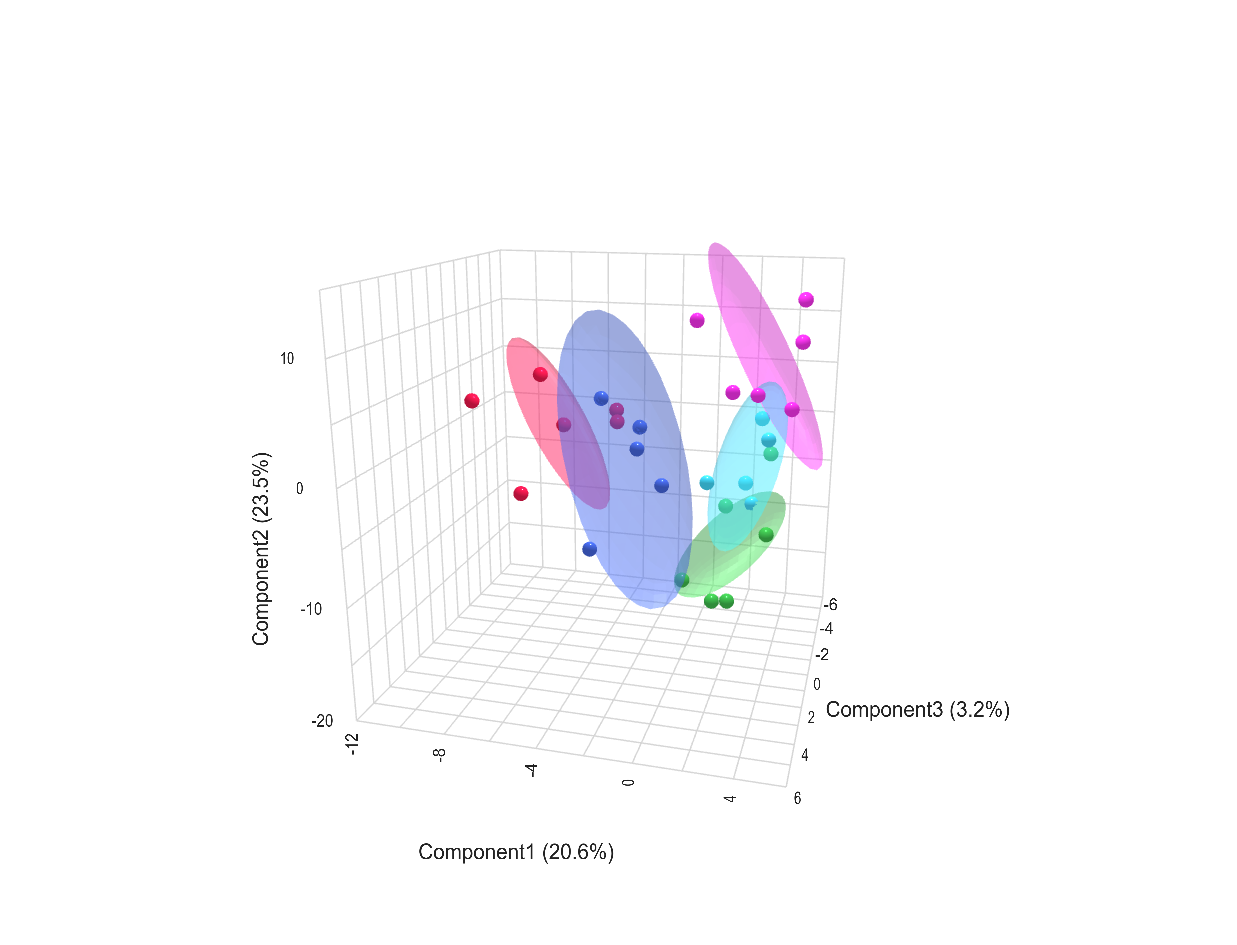

Supplement: Supplementary file 1 [file DataSheet3.zip › Plasma metabolomics analysis/PLS-DA/scatter3D (3).png]

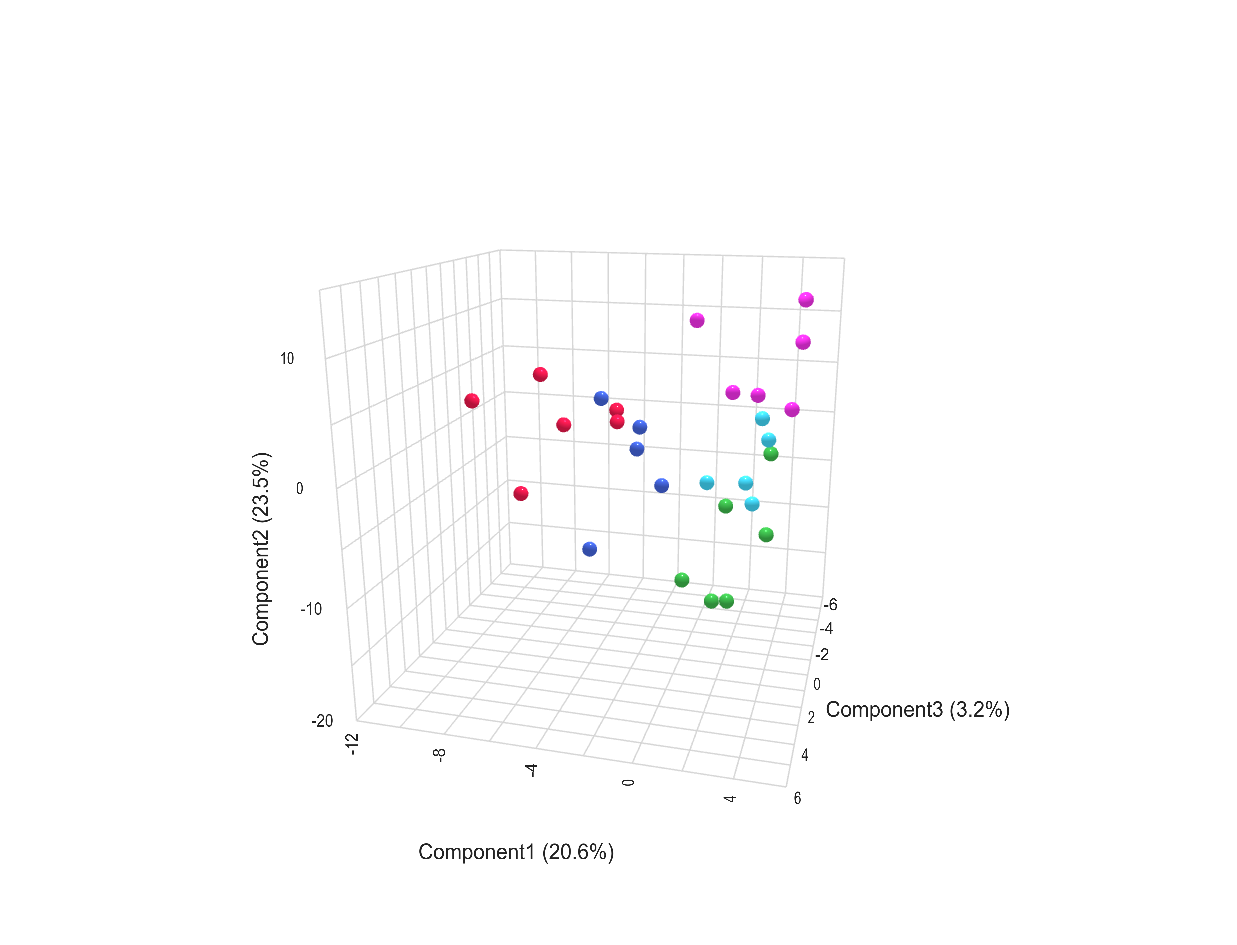

Supplement: Supplementary file 1 [file DataSheet3.zip › Plasma metabolomics analysis/PLS-DA/scatter3D (4).png]

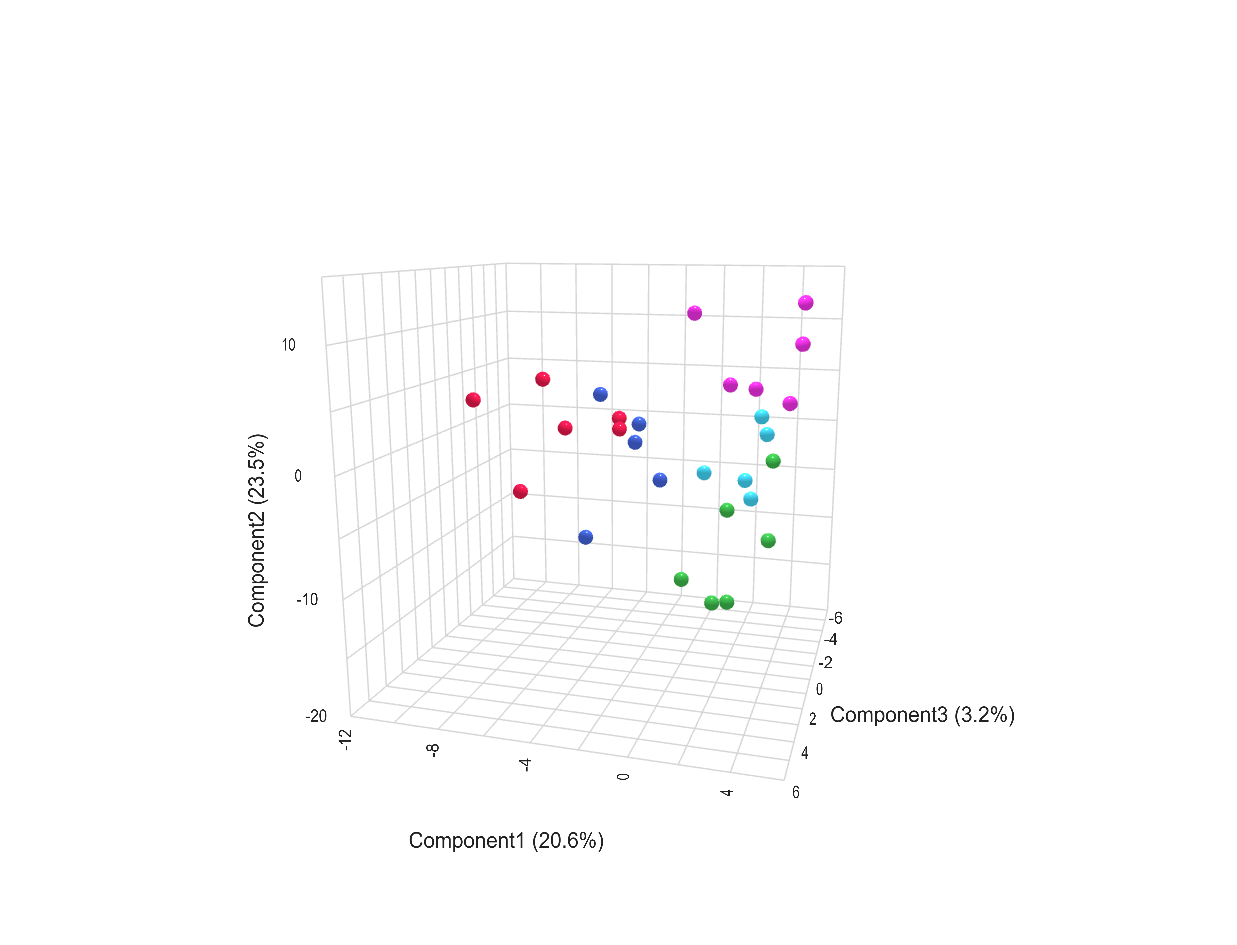

Supplement: Supplementary file 1 [file DataSheet3.zip › Plasma metabolomics analysis/PLS-DA/scatter3D (6).png]

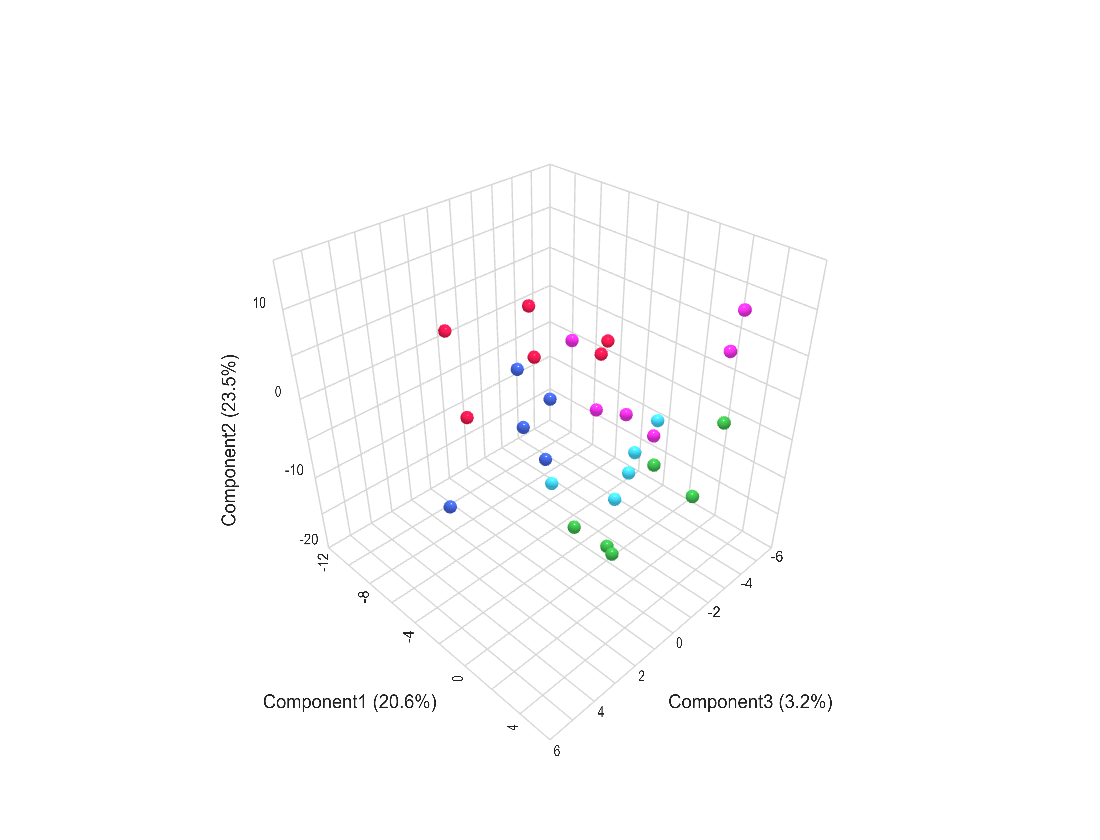

Supplement: Supplementary file 1 [file DataSheet3.zip › Plasma metabolomics analysis/PLS-DA/scatter3D -PLS-DA.png]

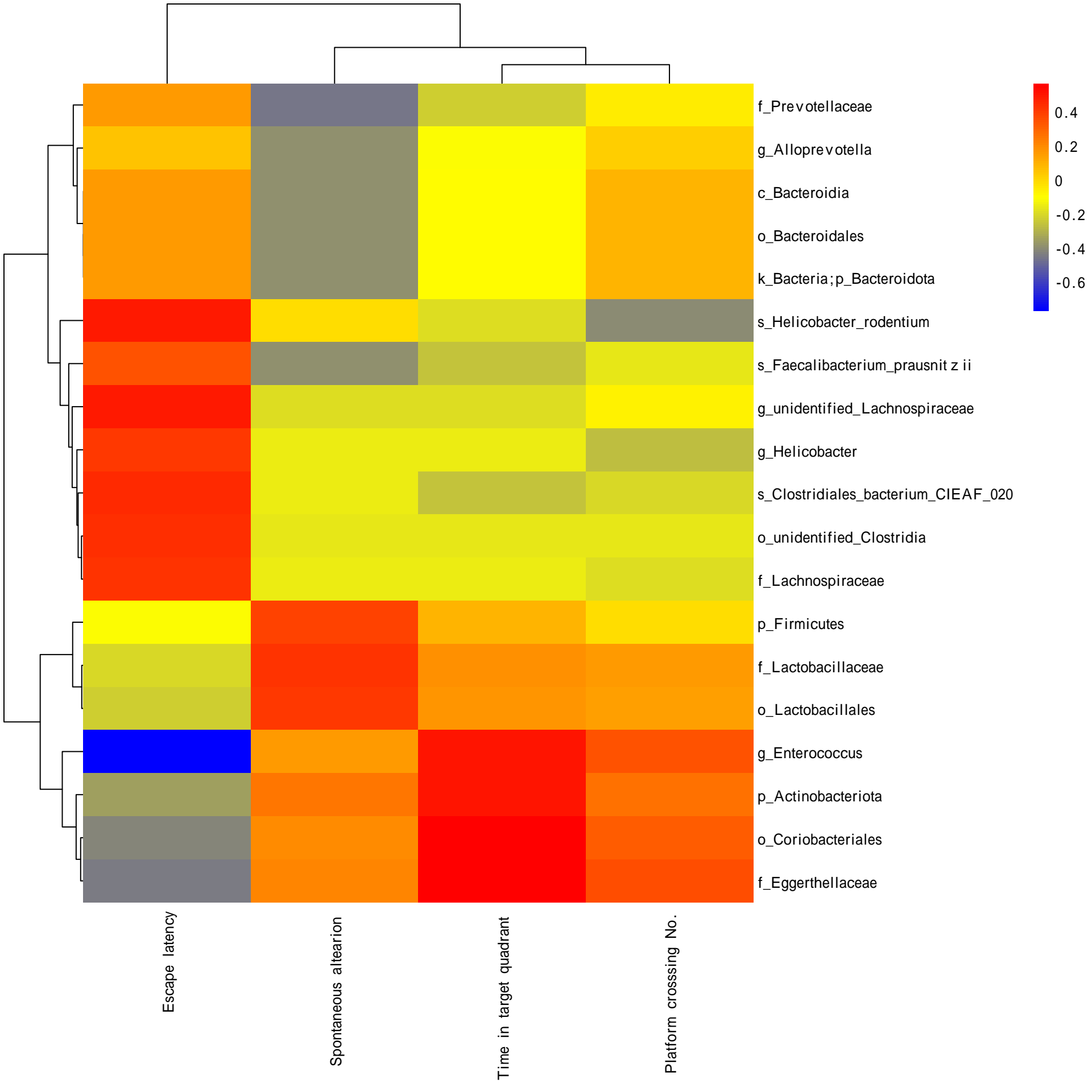

Supplement: Supplementary file 2 [file DataSheet8.zip › result.pdf]

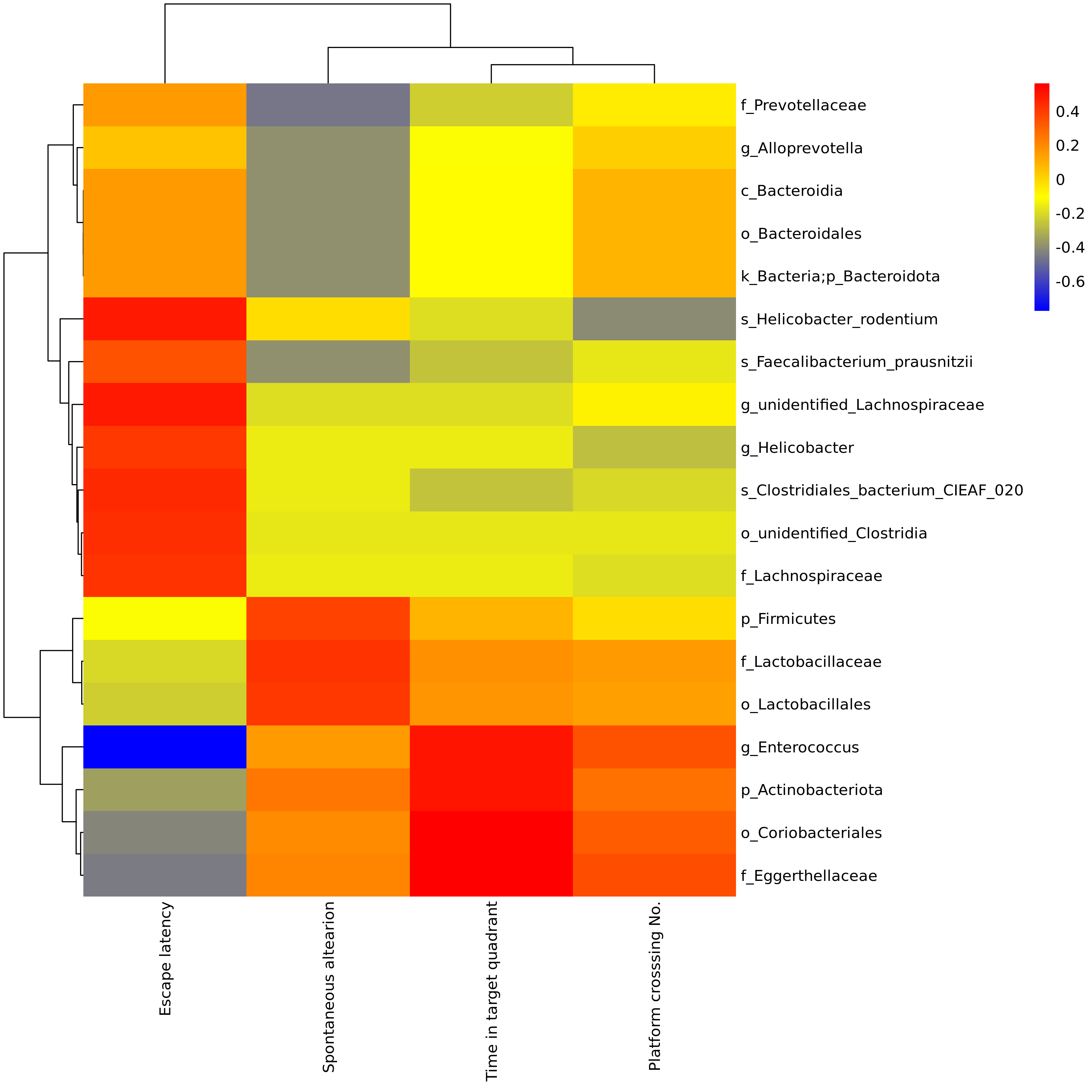

Supplement: Supplementary file 2 [file DataSheet8.zip › result.png]

**The original images of Western blot experiments**


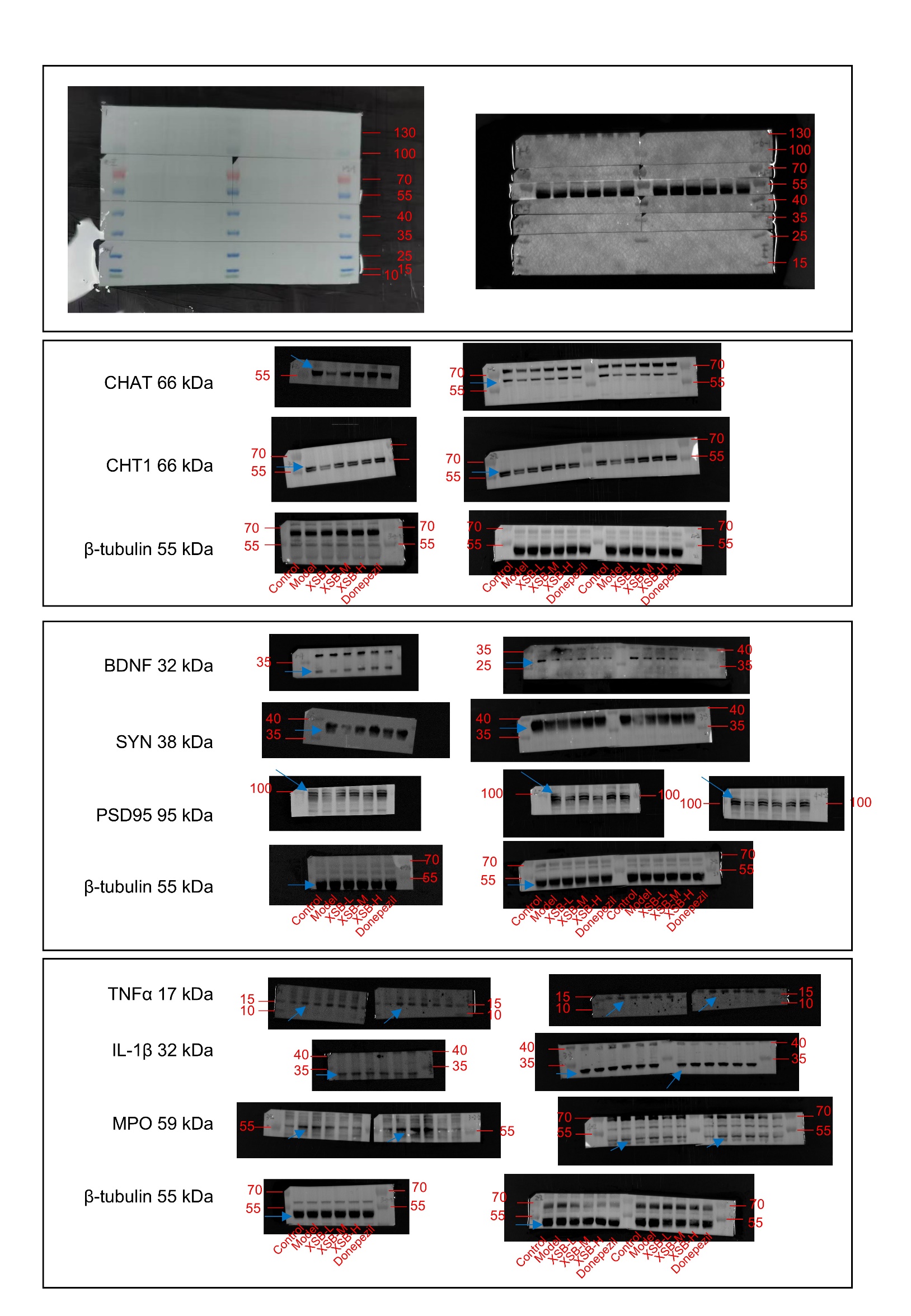

Supplement: Supplementary file 6 [file Supplementaryfile3.docx]

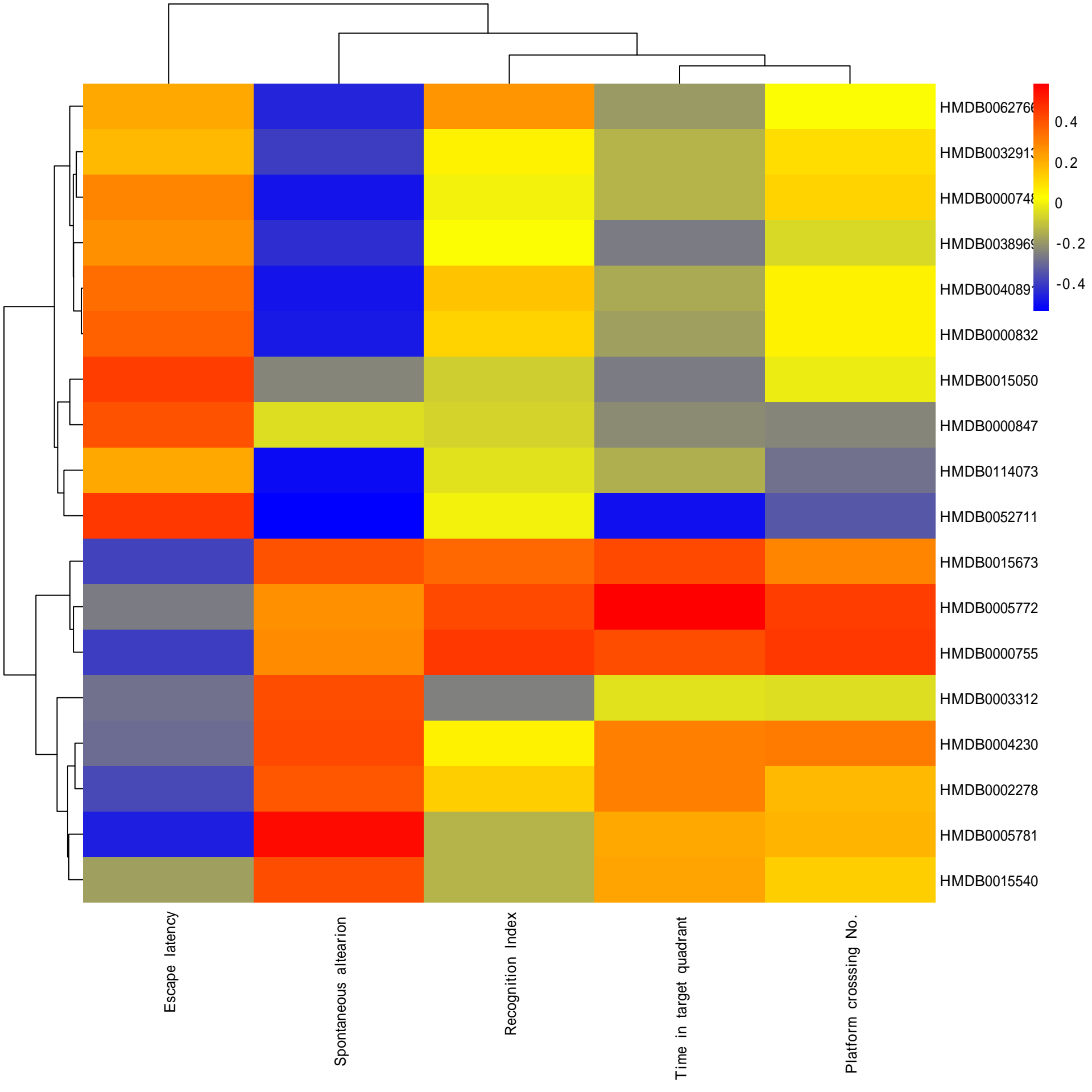

Supplement: Supplementary file 7 [file DataSheet6.zip › result.pdf]

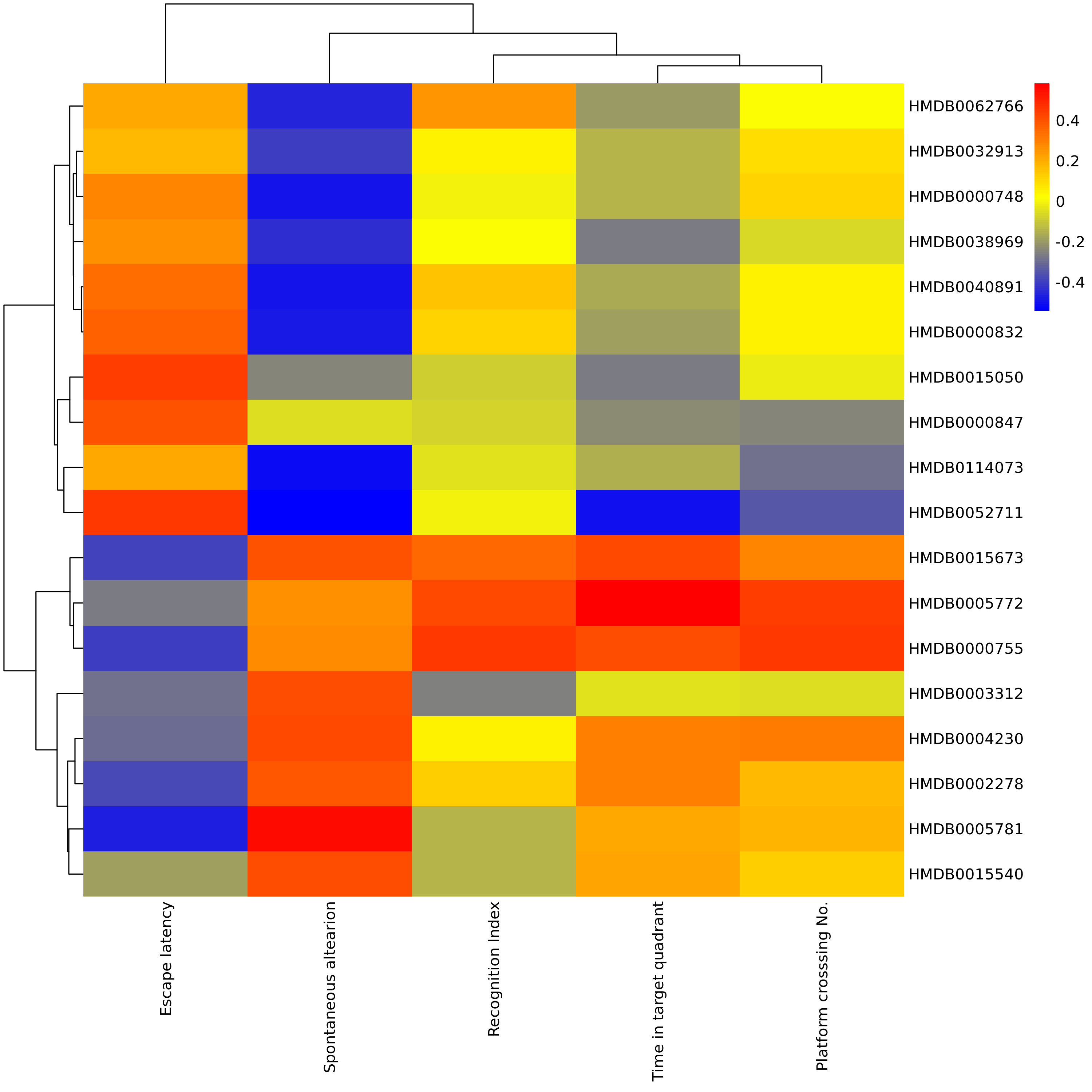

Supplement: Supplementary file 7 [file DataSheet6.zip › result.png]

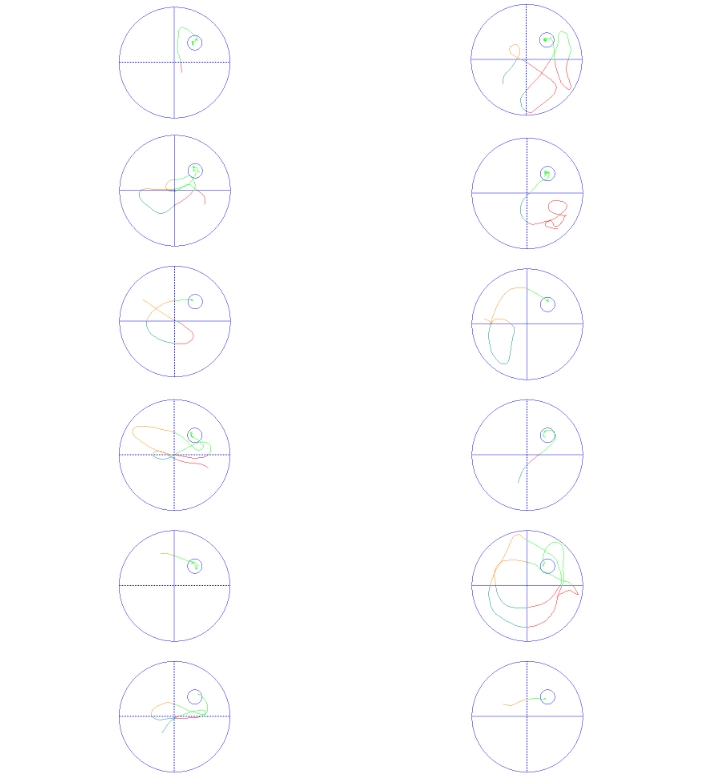

Supplement: Supplementary file 8 [file DataSheet2.zip › Behavior tests images/Morris water maze test/水迷宫-XSB-h.png]

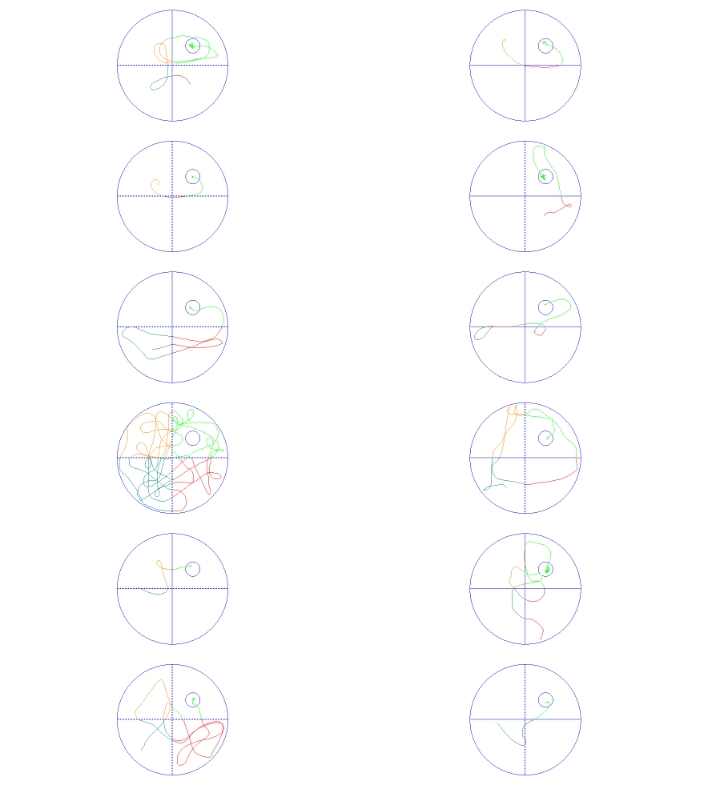

Supplement: Supplementary file 8 [file DataSheet2.zip › Behavior tests images/Morris water maze test/水迷宫-XSB-l.png]

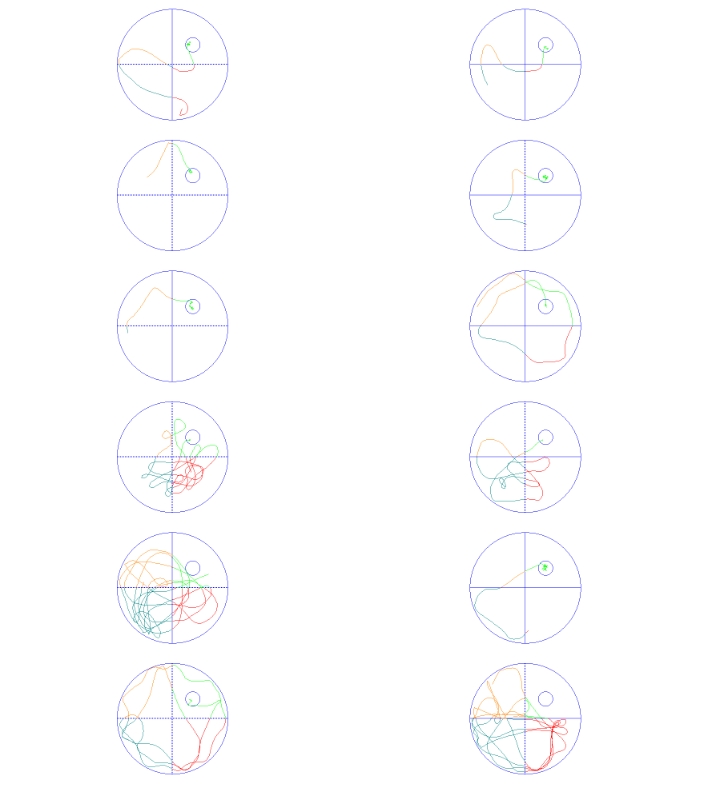

Supplement: Supplementary file 8 [file DataSheet2.zip › Behavior tests images/Morris water maze test/水迷宫-XSB-m.png]

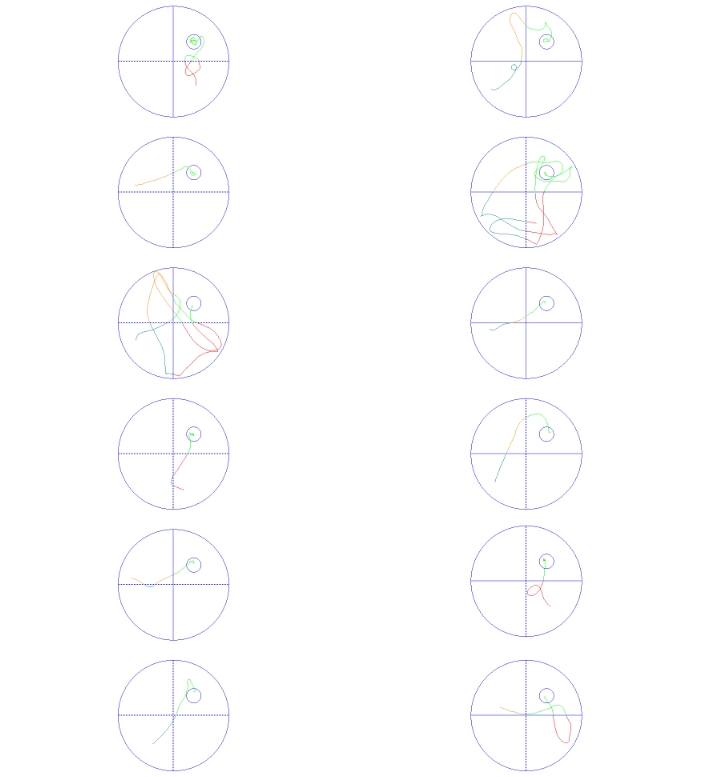

Supplement: Supplementary file 8 [file DataSheet2.zip › Behavior tests images/Morris water maze test/水迷宫-control.png]

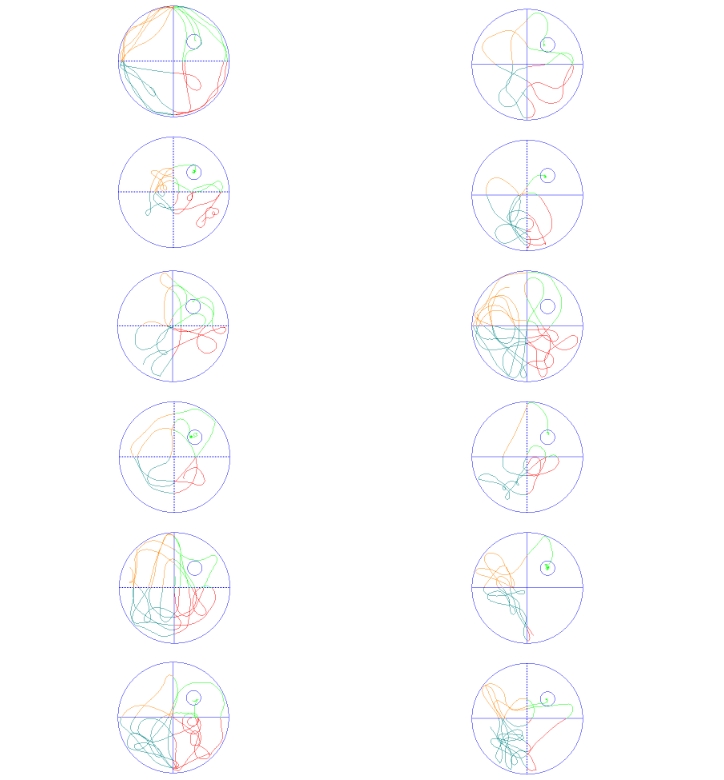

Supplement: Supplementary file 8 [file DataSheet2.zip › Behavior tests images/Morris water maze test/水迷宫-model.png]

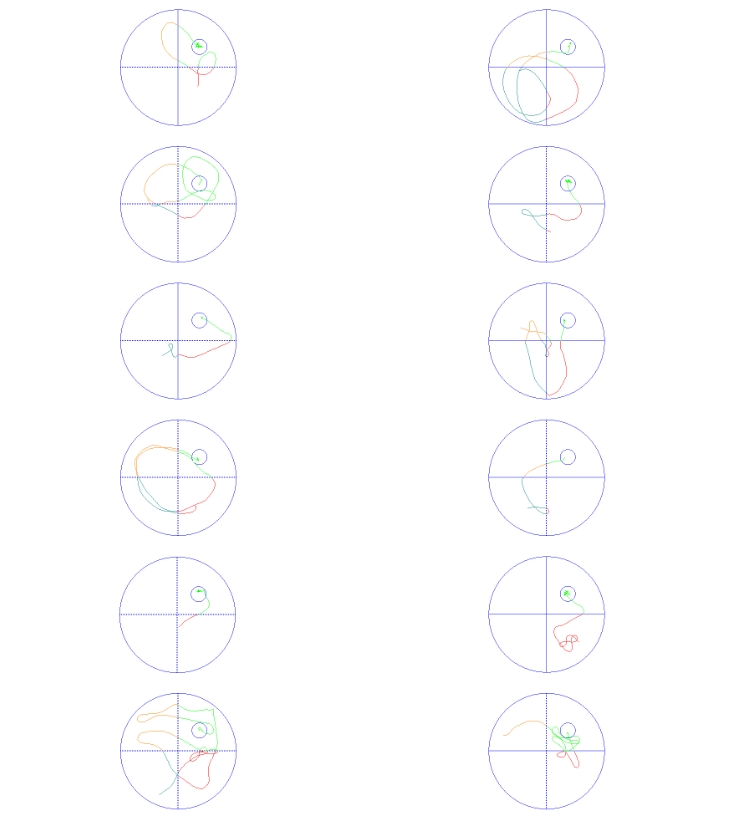

Supplement: Supplementary file 8 [file DataSheet2.zip › Behavior tests images/Morris water maze test/水迷宫-安理申.png]

ALS


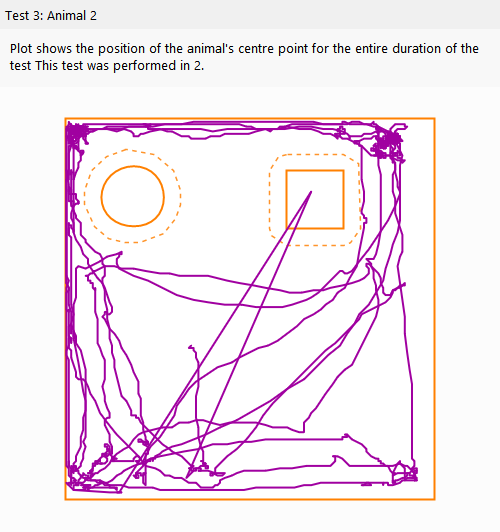

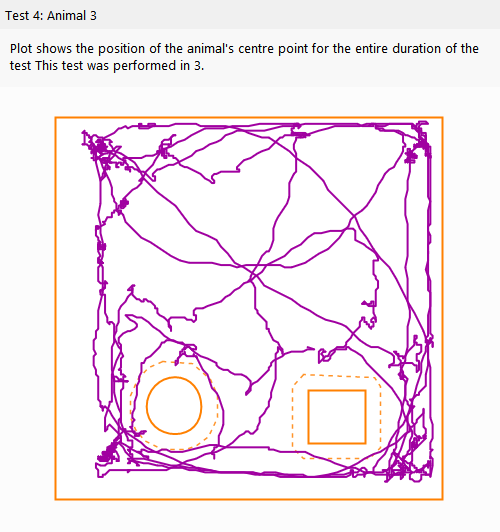

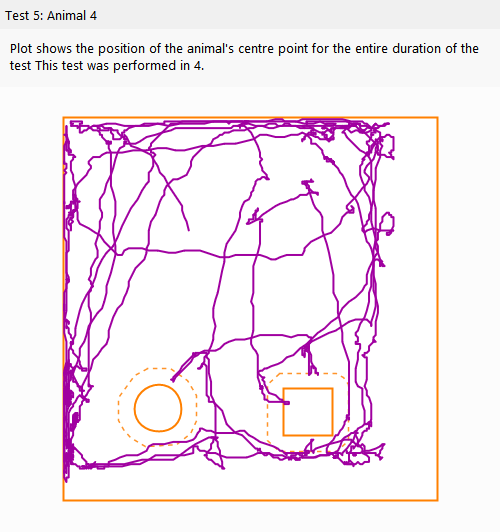

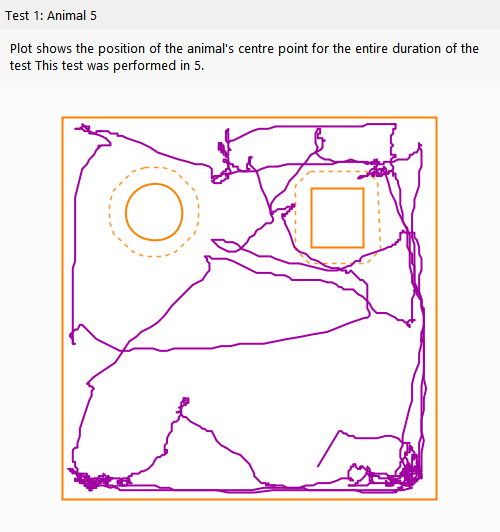

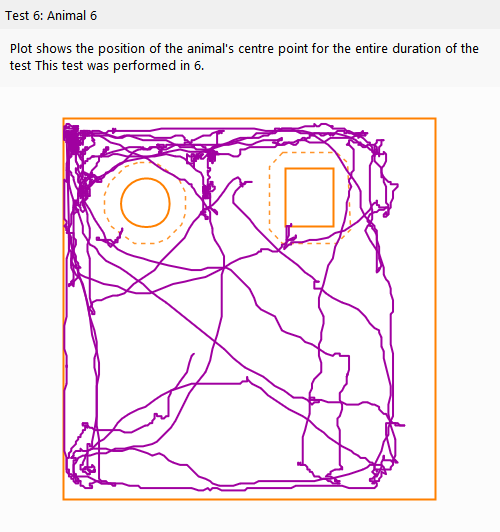

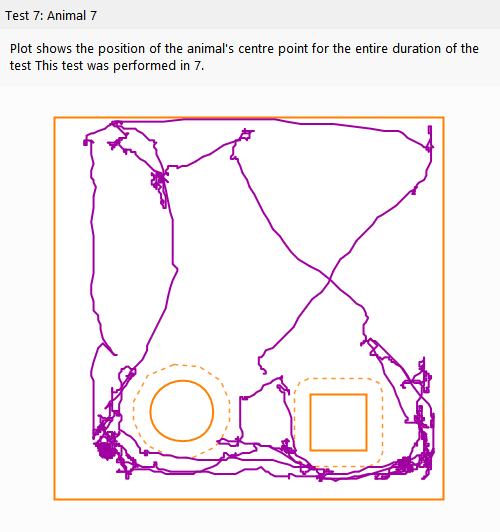

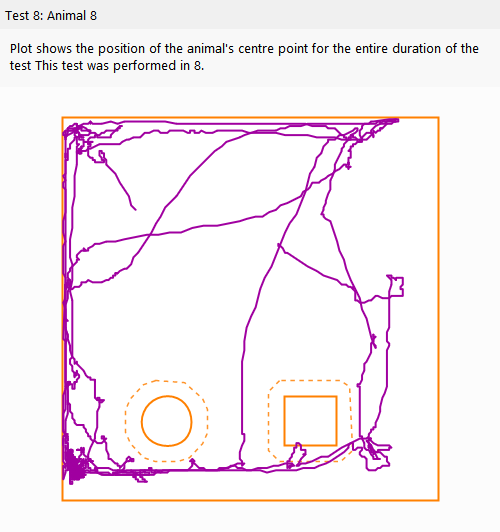

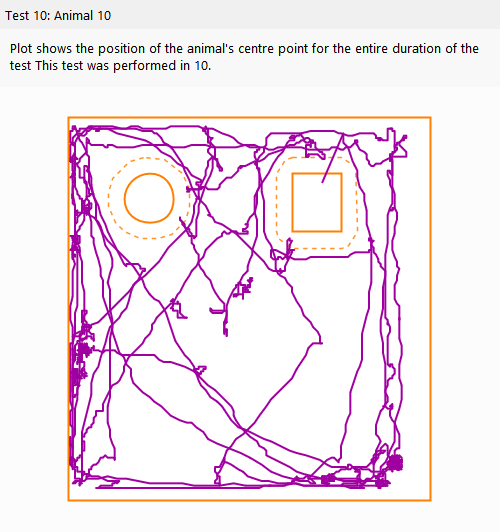

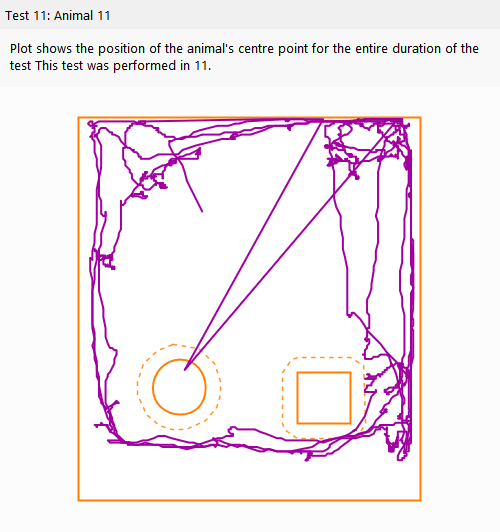

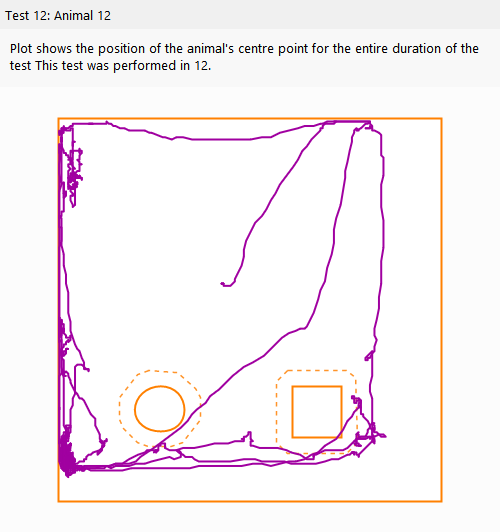

Supplement: Supplementary file 8 [file DataSheet2.zip › Behavior tests images/Novel Object Recognition/NOR-ALS-轨迹图.docx]

XSB-L


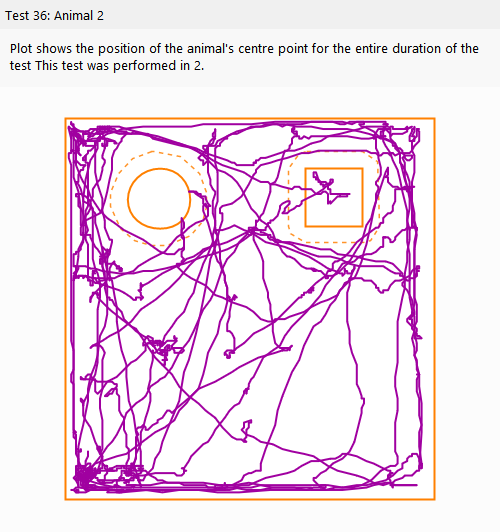

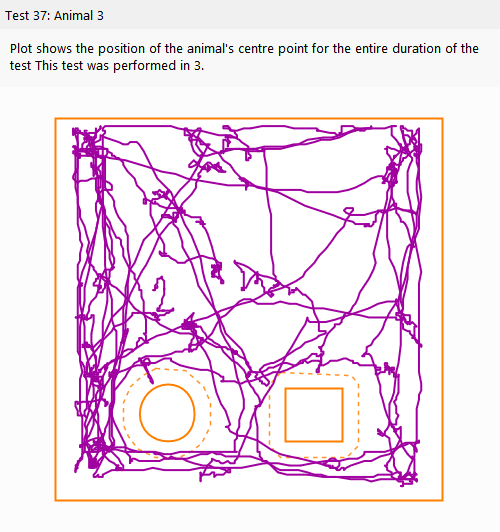

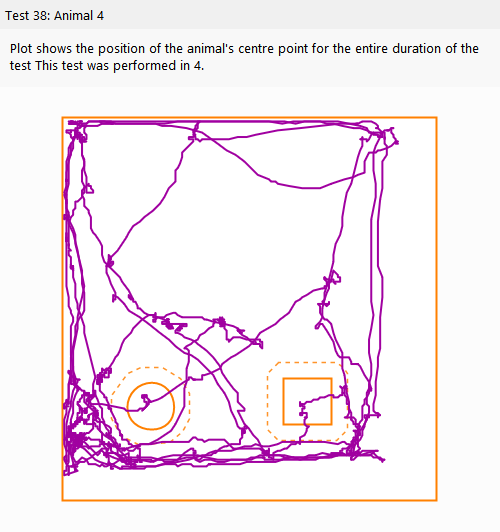

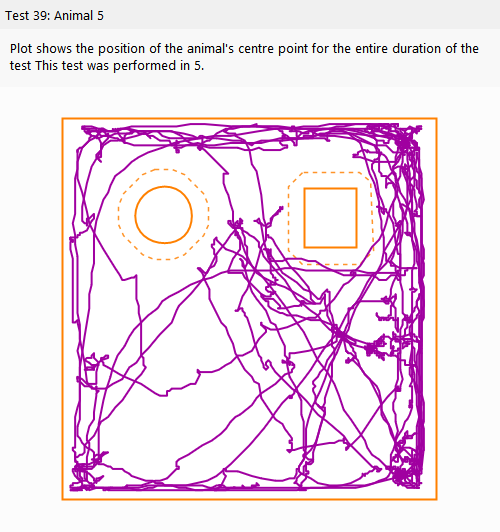

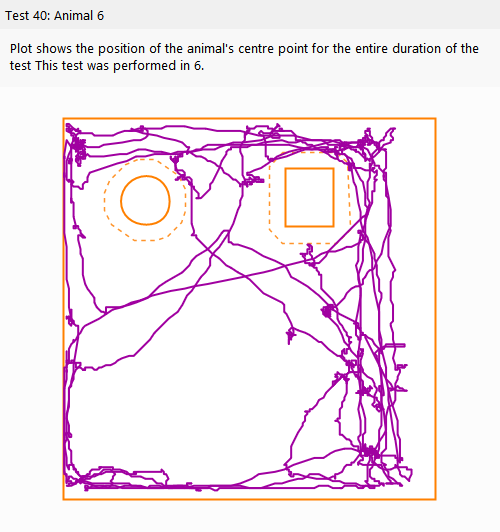

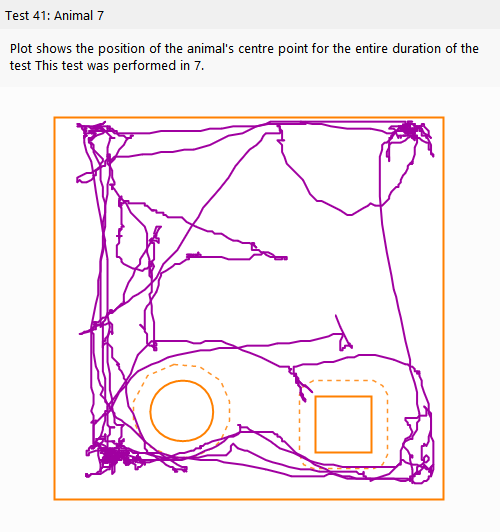

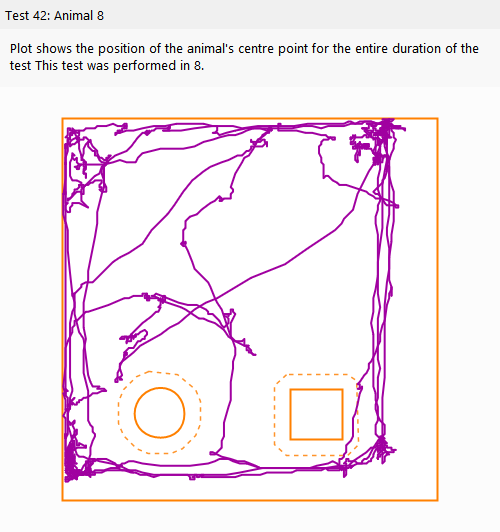

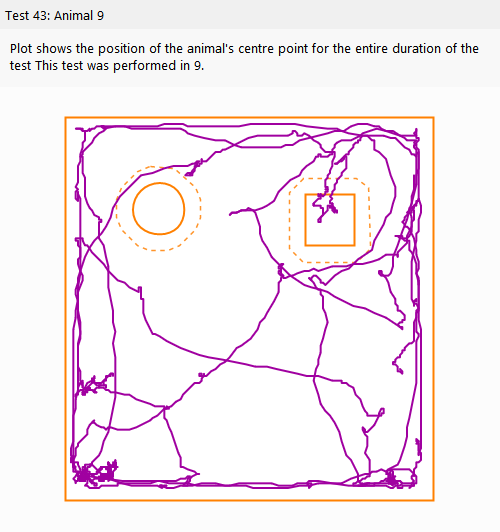

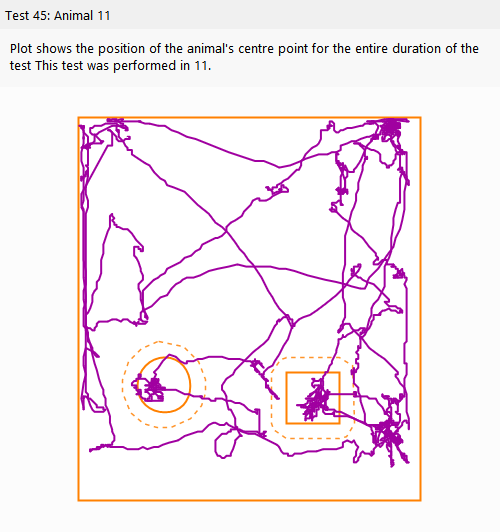

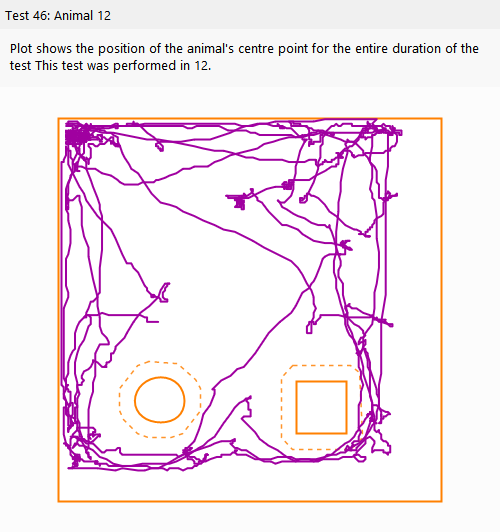

Supplement: Supplementary file 8 [file DataSheet2.zip › Behavior tests images/Novel Object Recognition/NOR-XSB-L-轨迹图.docx]

XSB-h


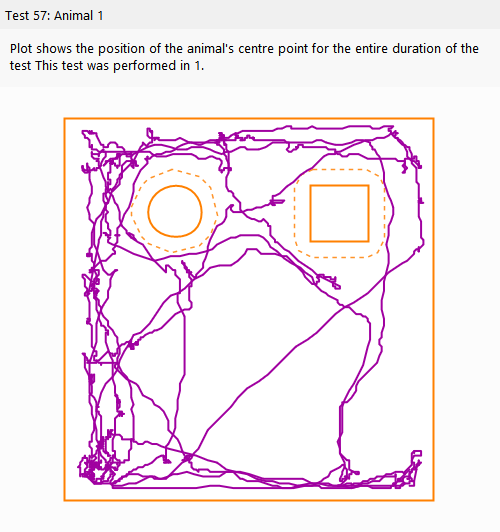

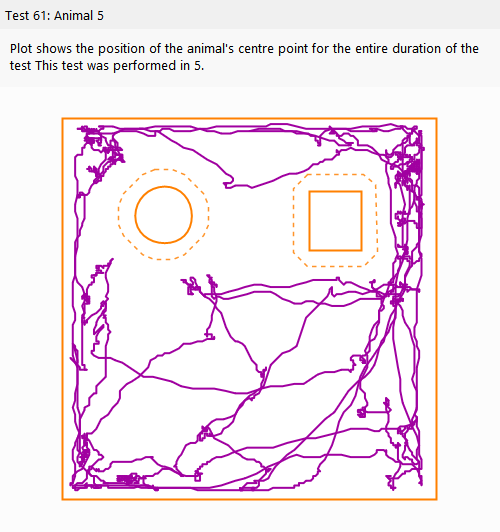

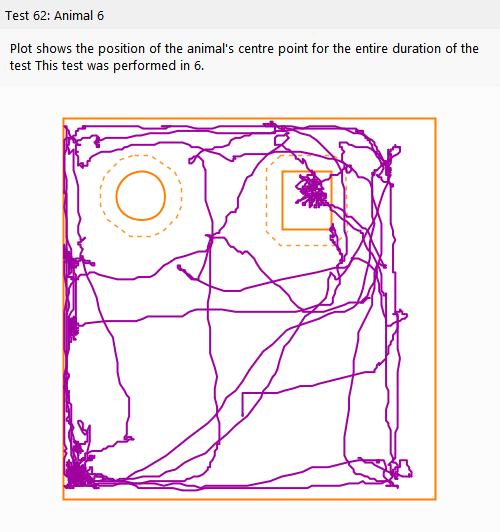

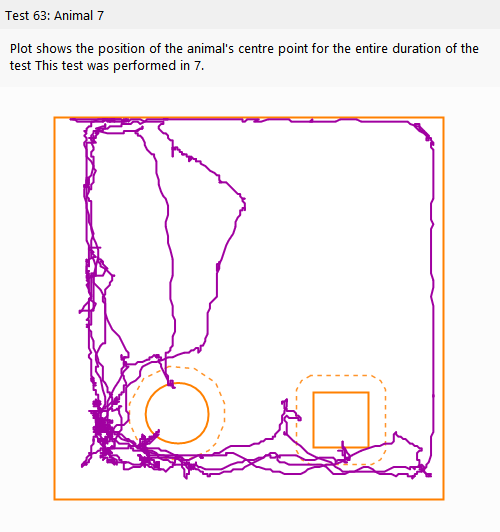

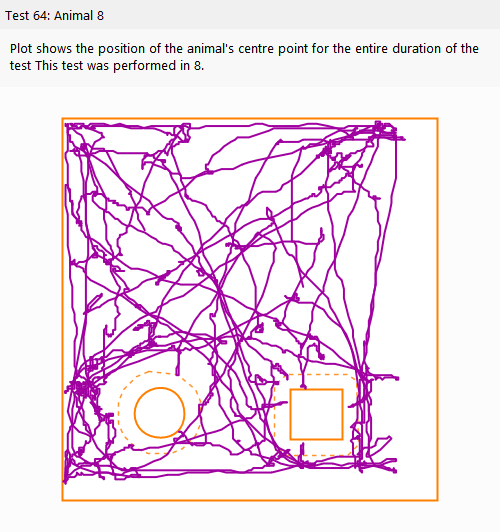

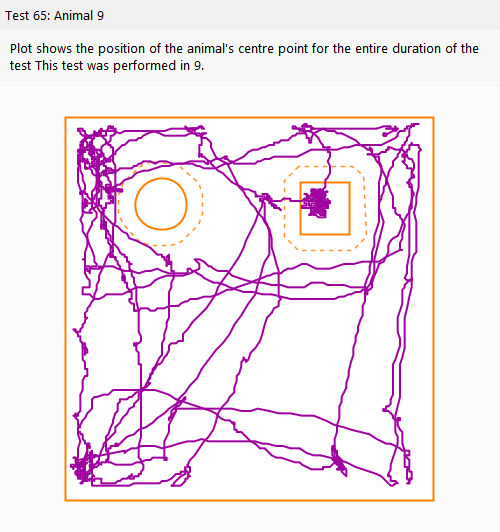

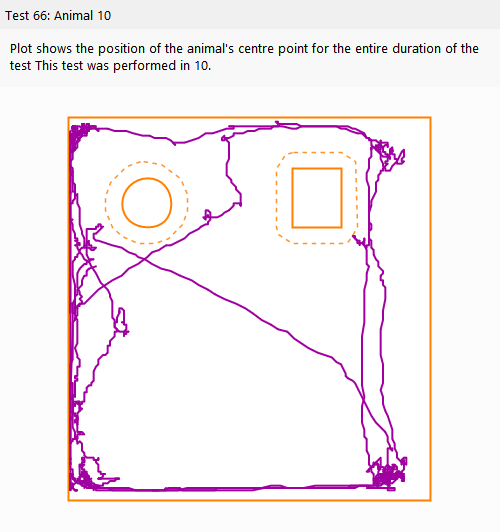

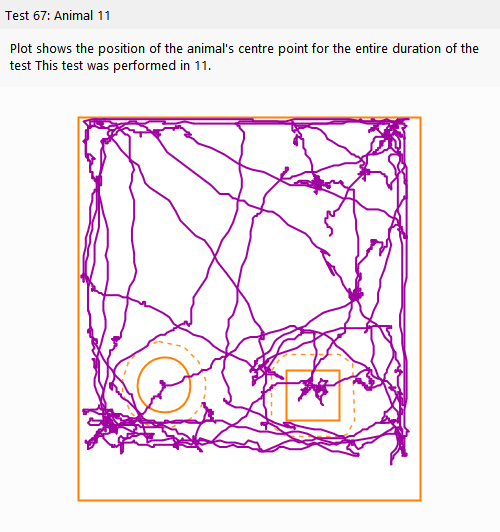

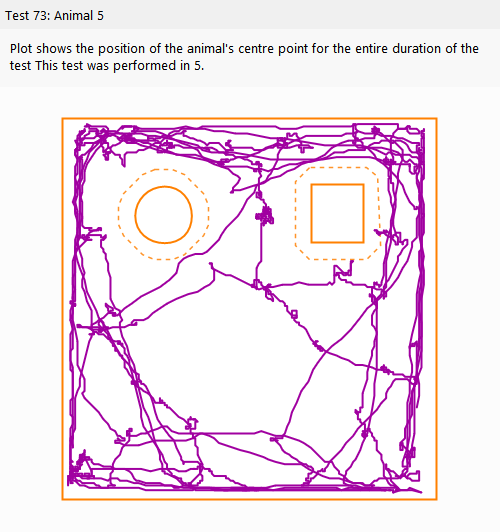

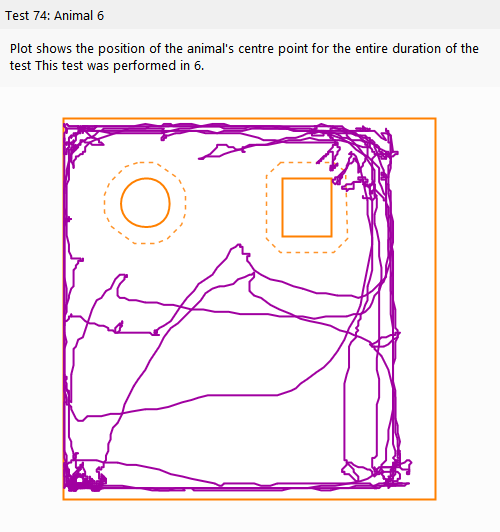

Supplement: Supplementary file 8 [file DataSheet2.zip › Behavior tests images/Novel Object Recognition/NOR-XSB-h-轨迹图.docx]

XSB-L


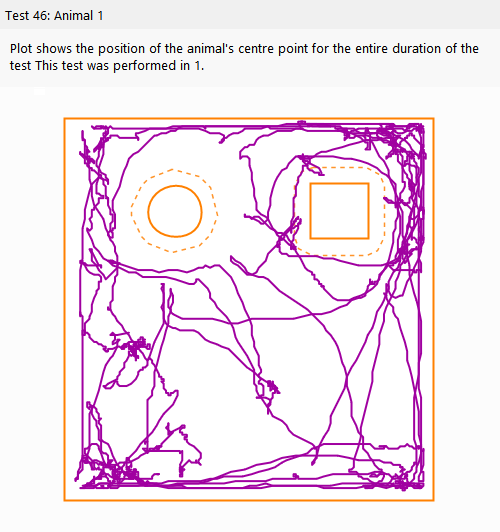

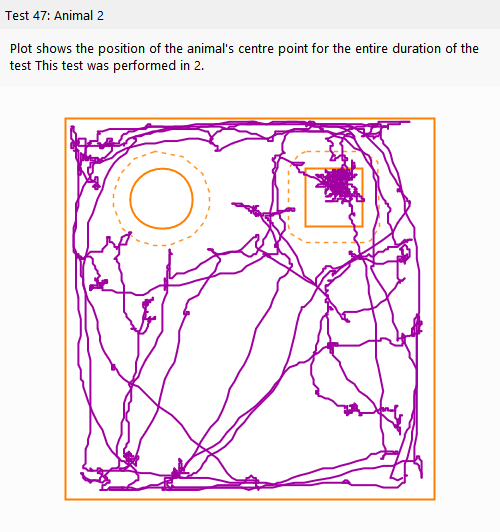

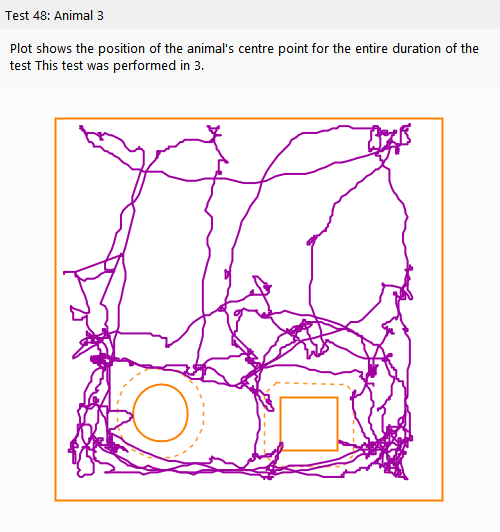

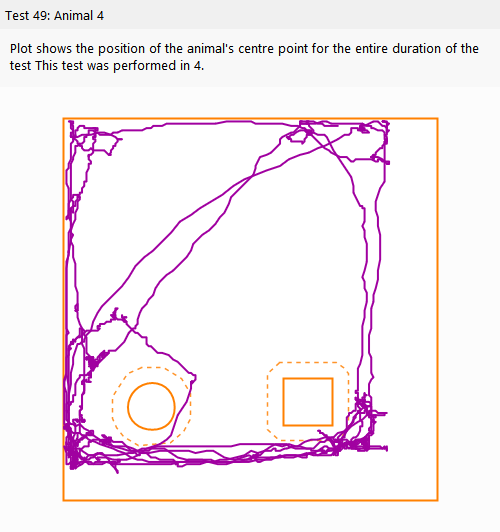

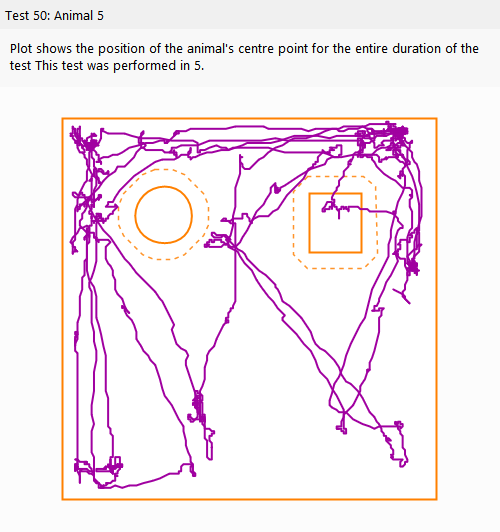

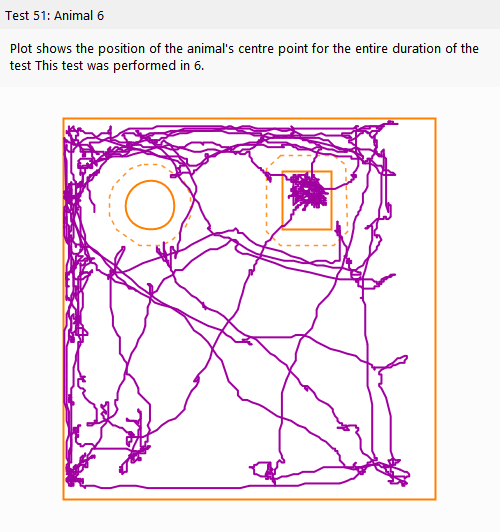

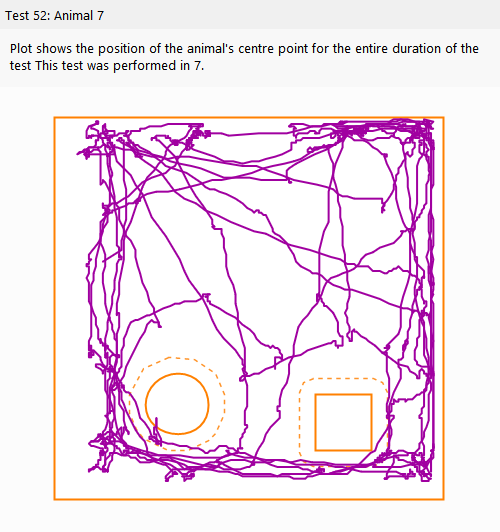

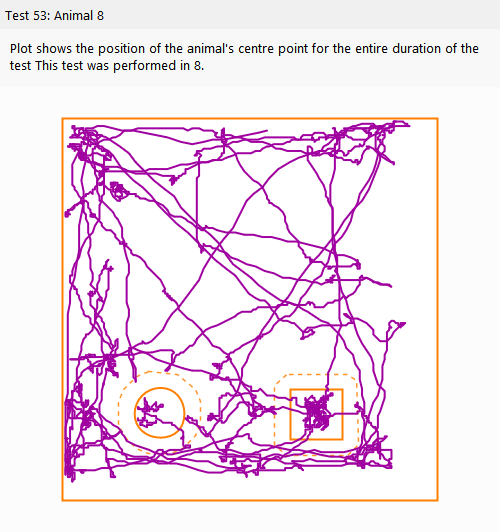

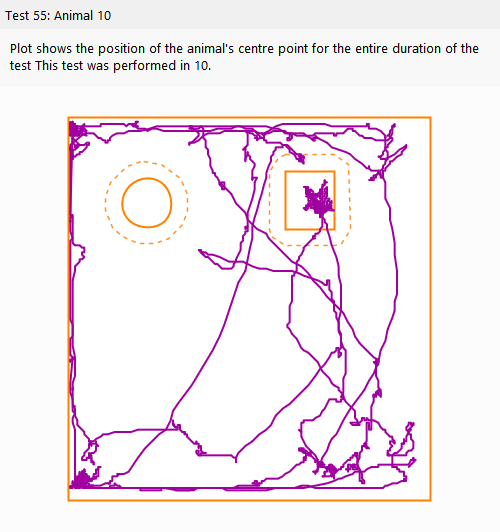

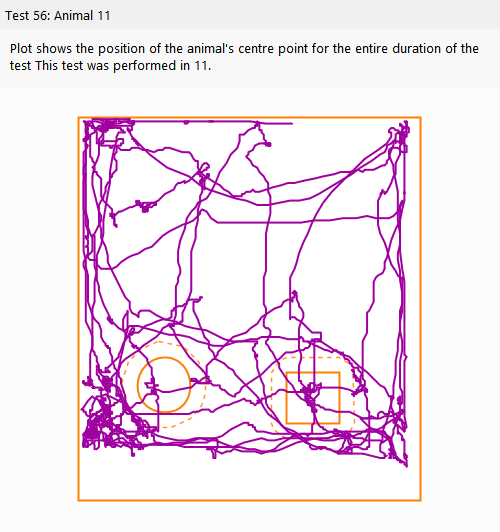

Supplement: Supplementary file 8 [file DataSheet2.zip › Behavior tests images/Novel Object Recognition/NOR-XSB-m-轨迹图.docx]

Con


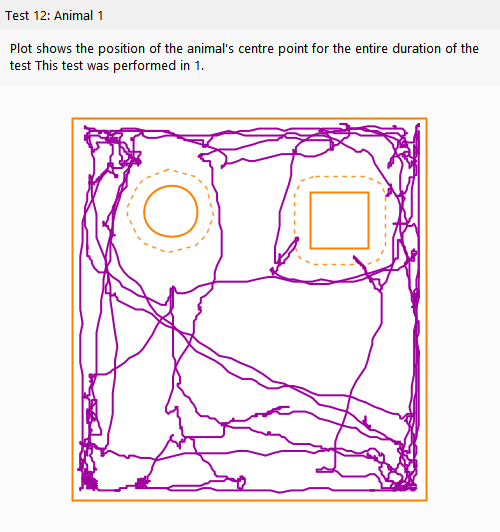

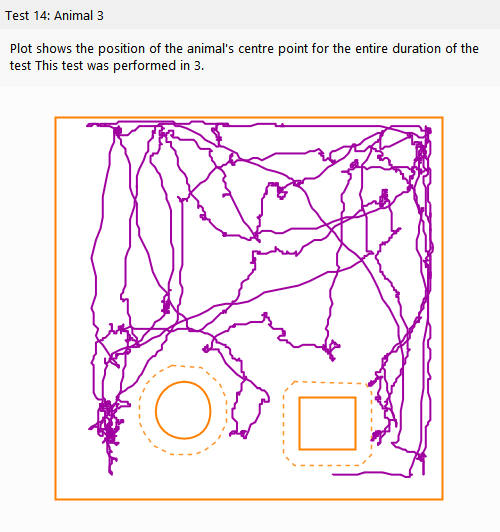

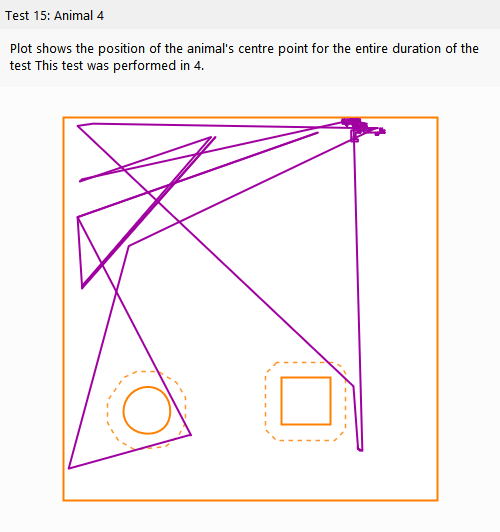

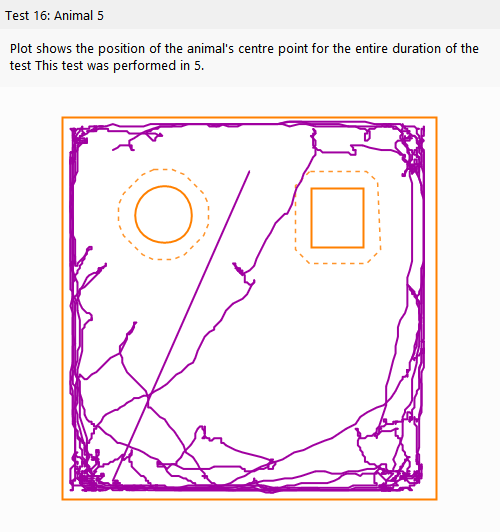

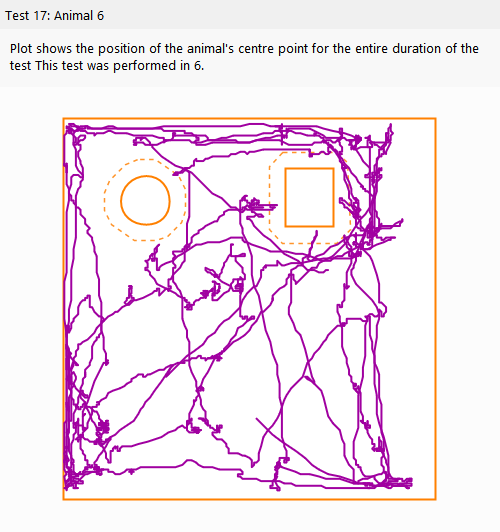

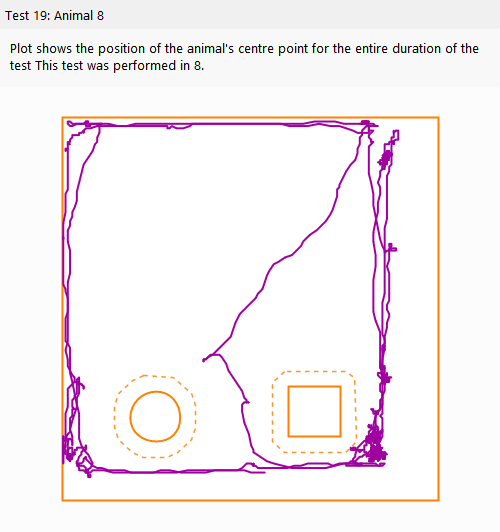

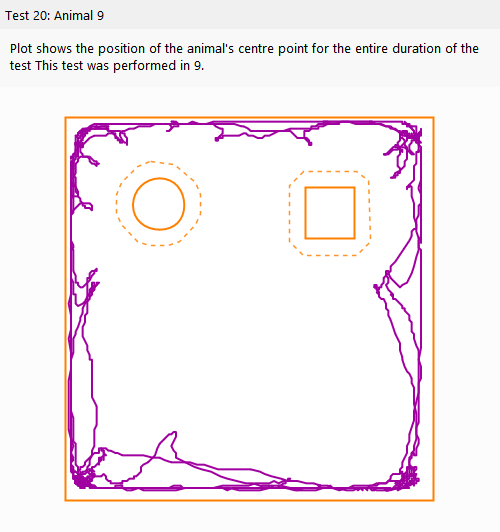

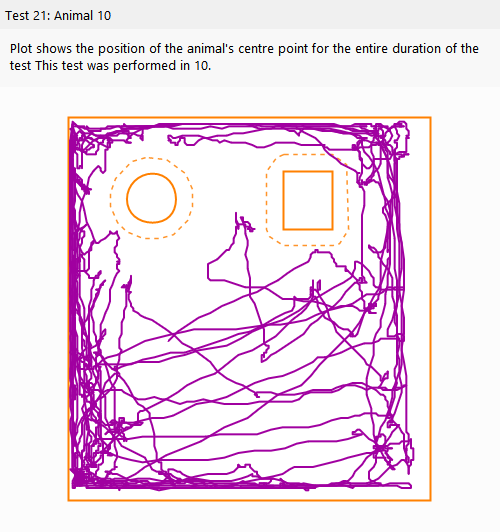

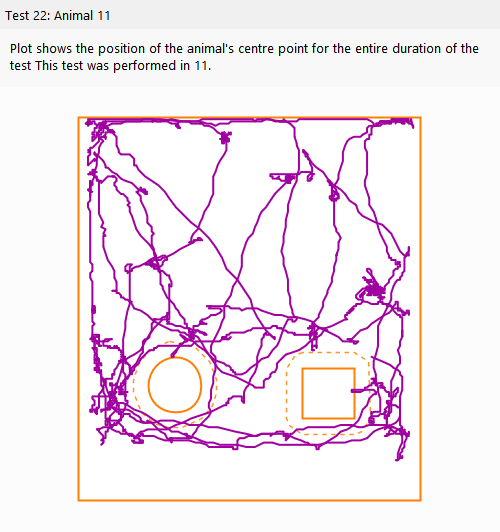

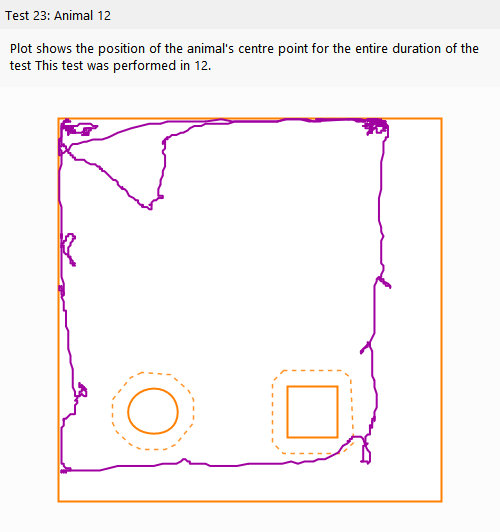

Supplement: Supplementary file 8 [file DataSheet2.zip › Behavior tests images/Novel Object Recognition/NOR-con-轨迹图.docx]

model


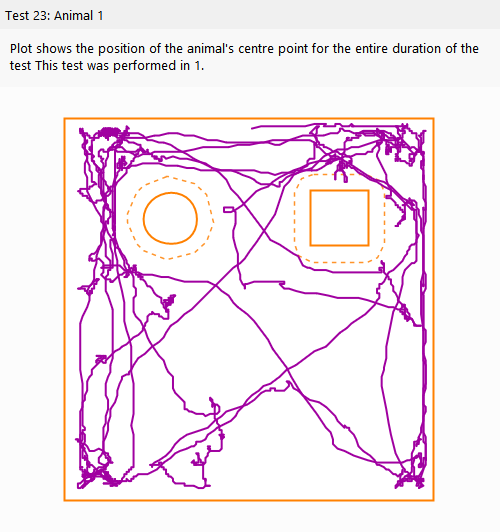

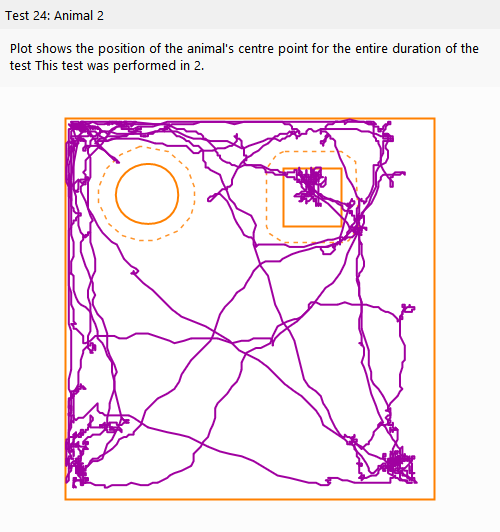

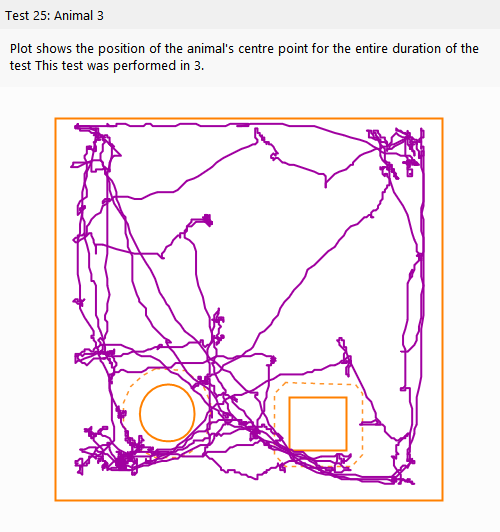

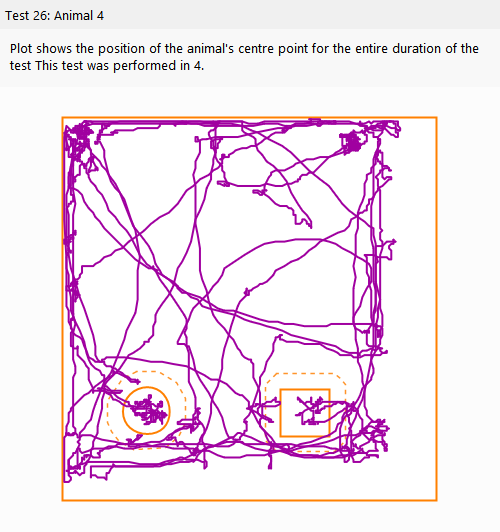

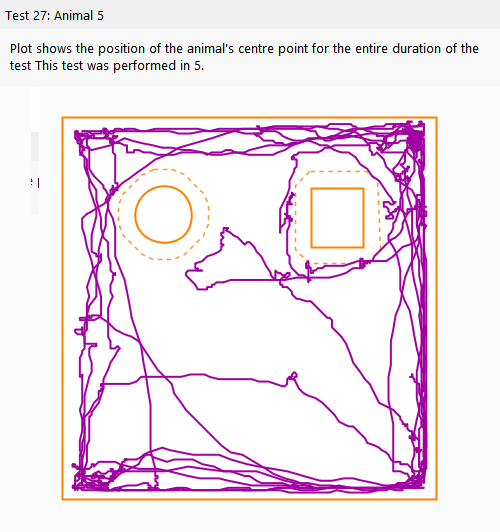

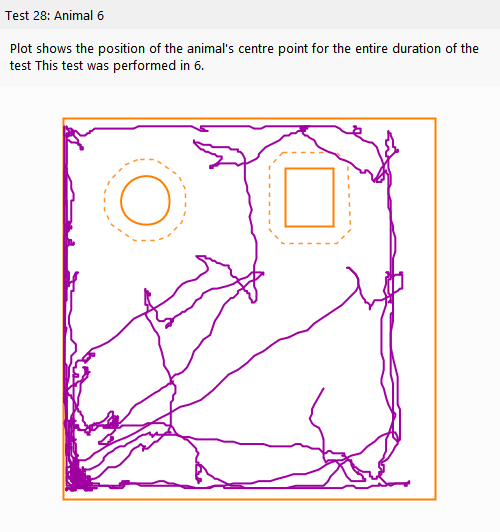

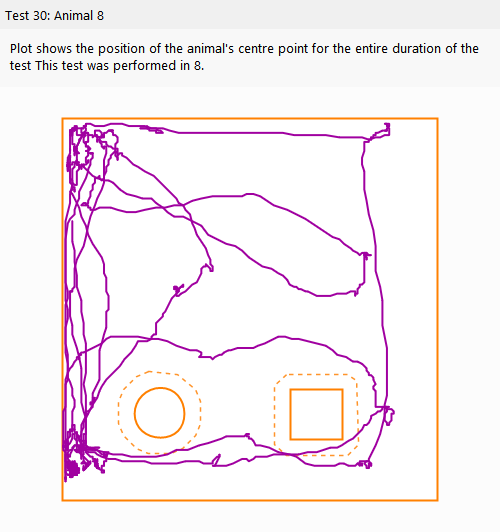

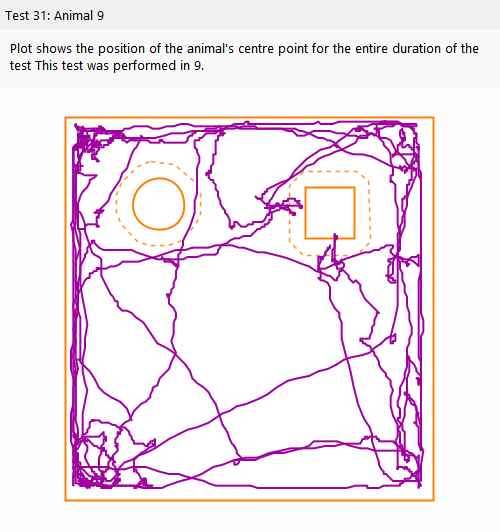

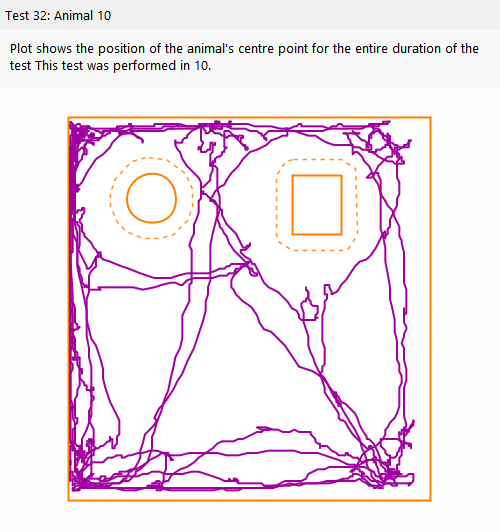

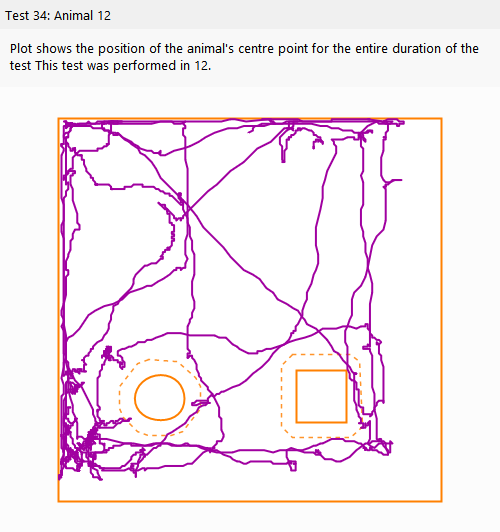

Supplement: Supplementary file 8 [file DataSheet2.zip › Behavior tests images/Novel Object Recognition/NOR-model-轨迹图.docx]

als


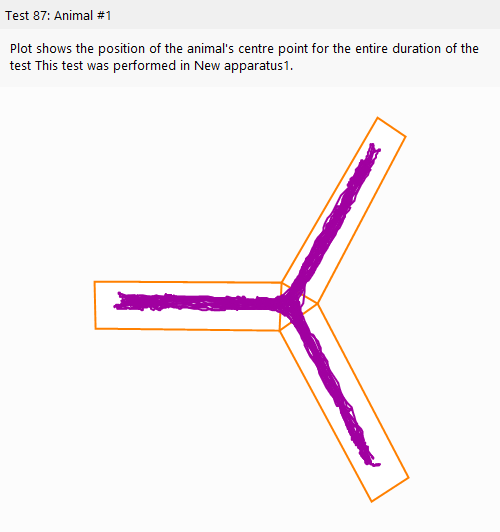

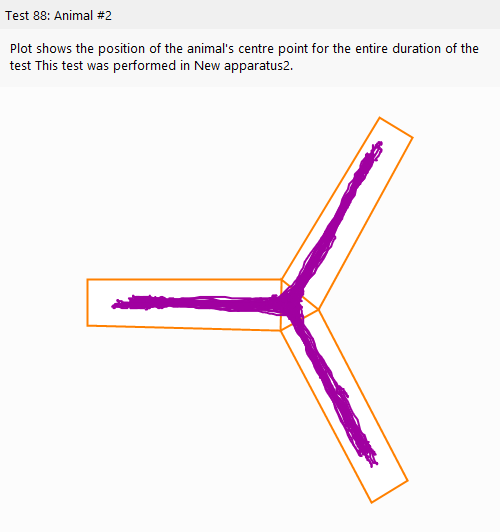

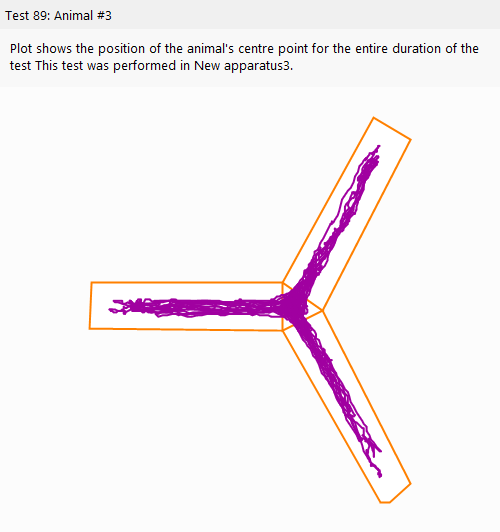

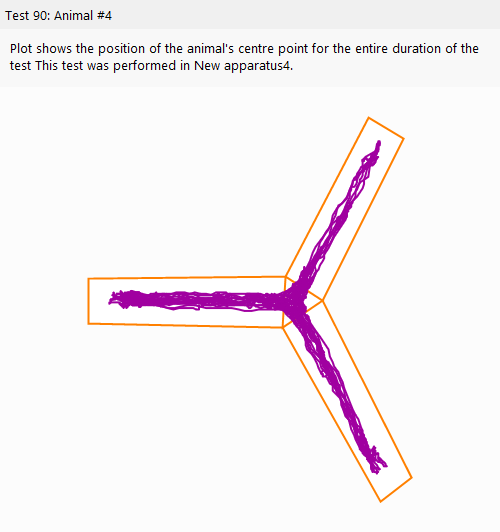

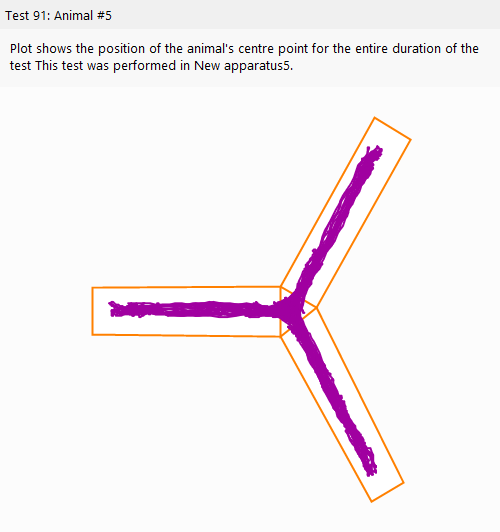

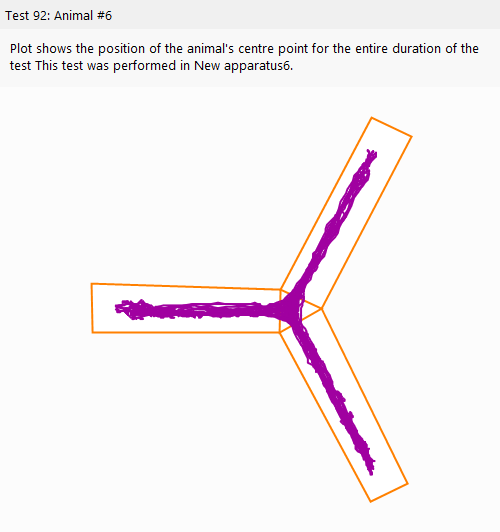

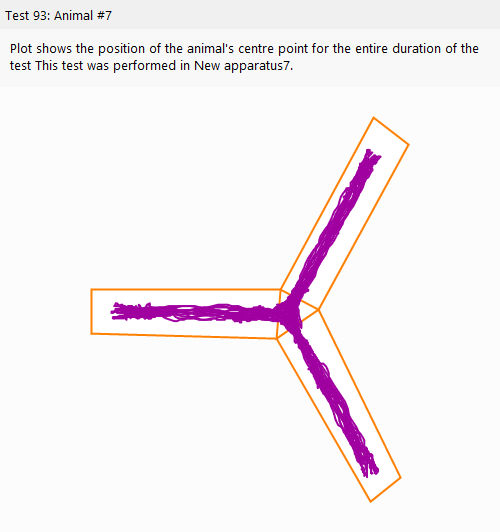

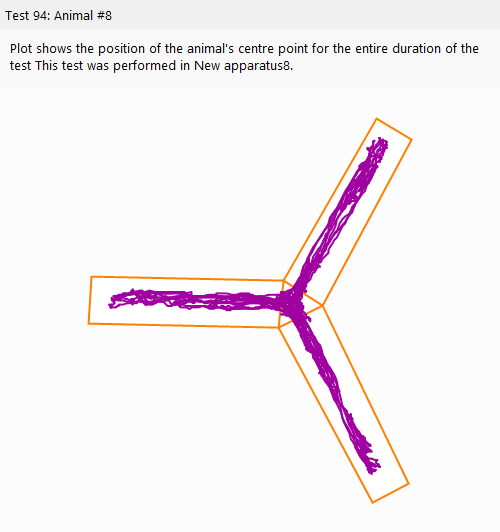

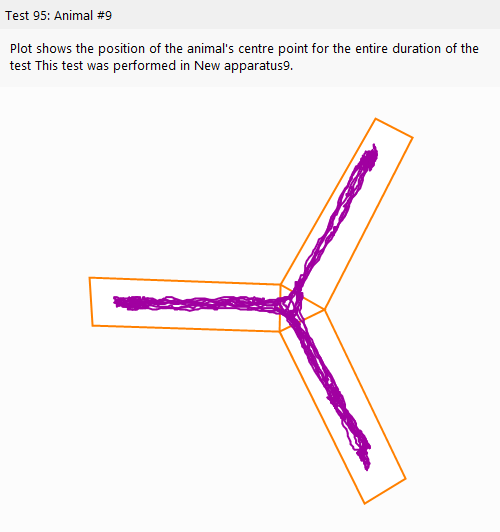

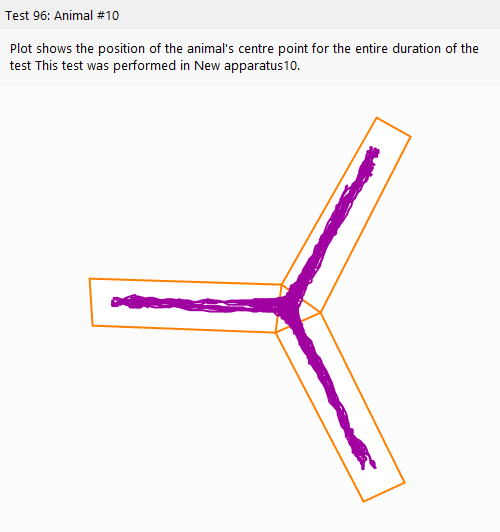

Supplement: Supplementary file 8 [file DataSheet2.zip › Behavior tests images/Y maze test/Y-maze-als-轨迹图.docx]

control


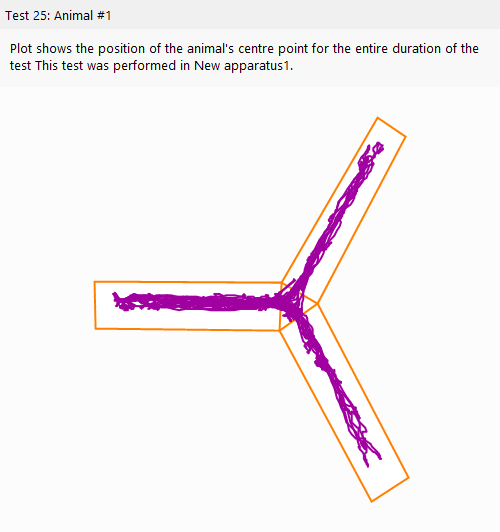

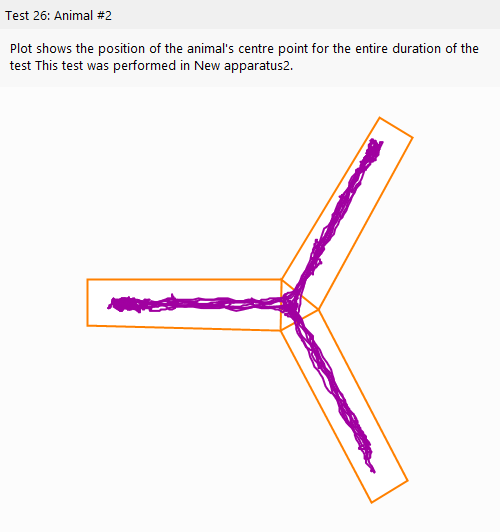

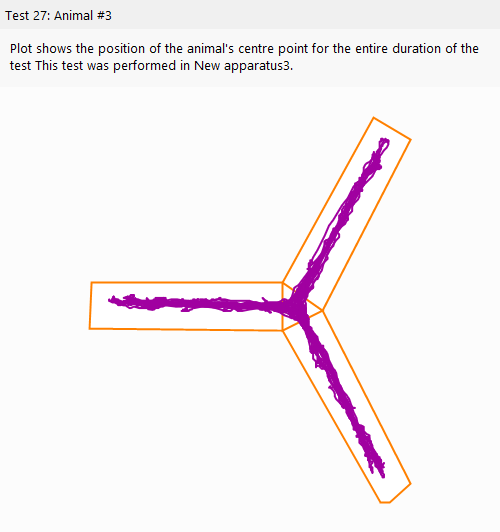

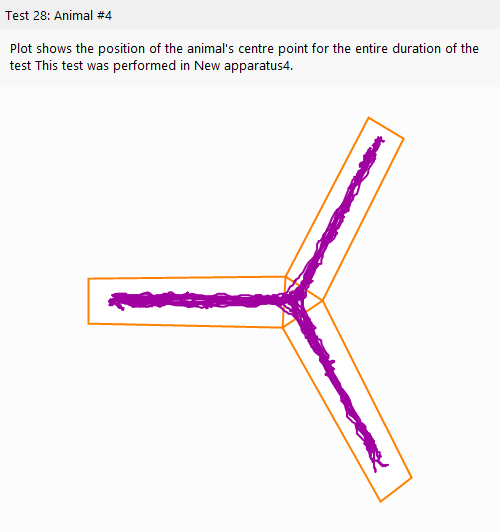

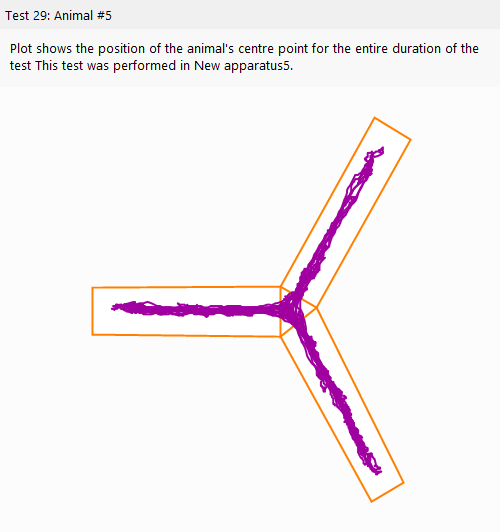

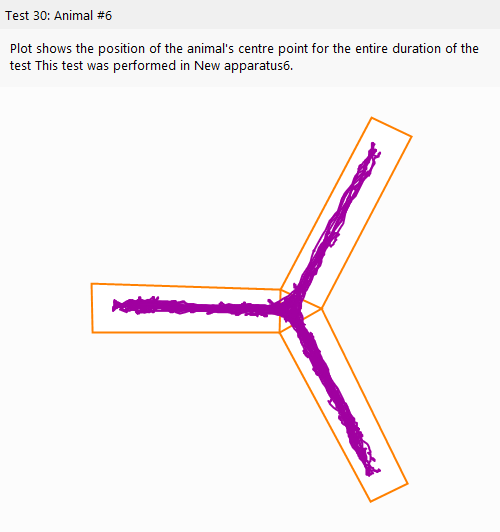

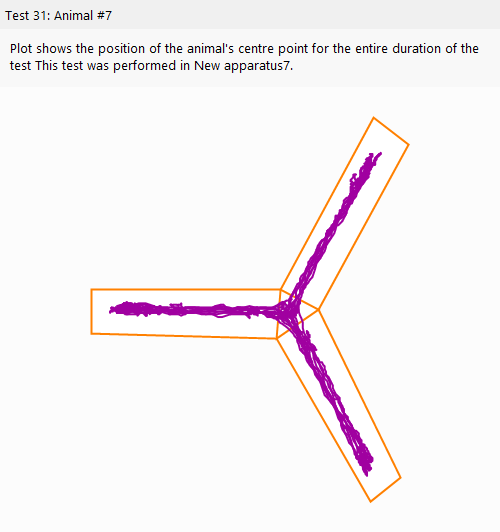

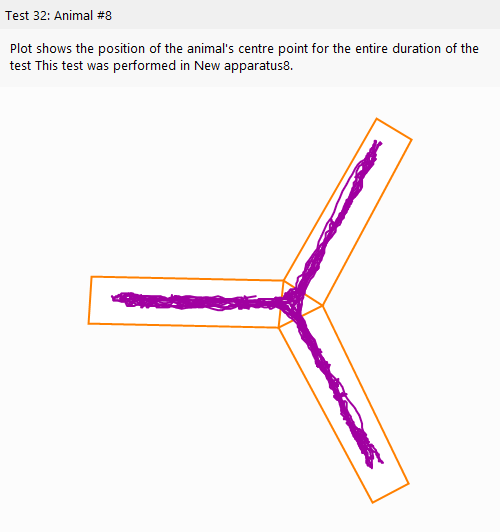

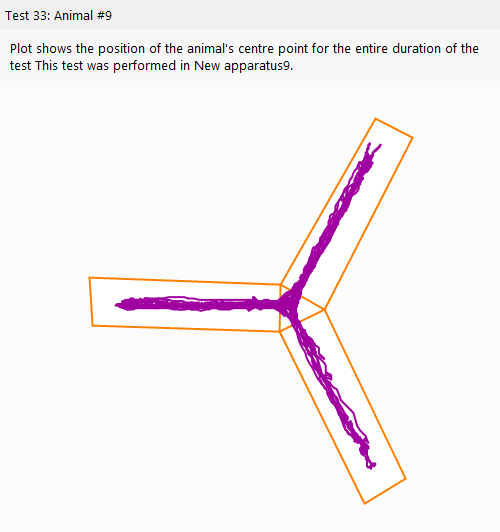

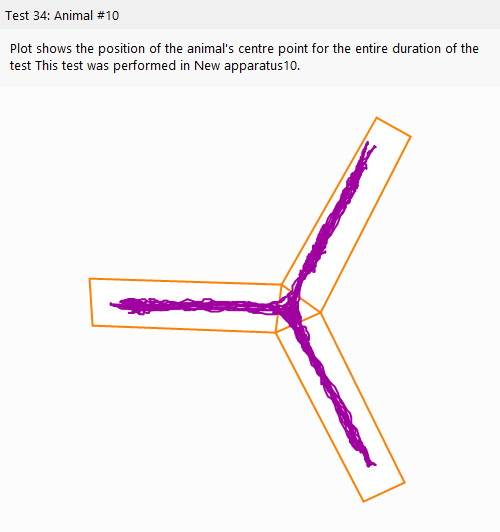

Supplement: Supplementary file 8 [file DataSheet2.zip › Behavior tests images/Y maze test/Y-maze-control-轨迹图.docx]

model


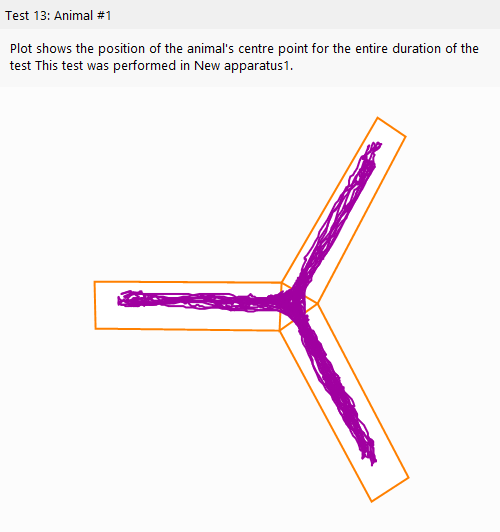

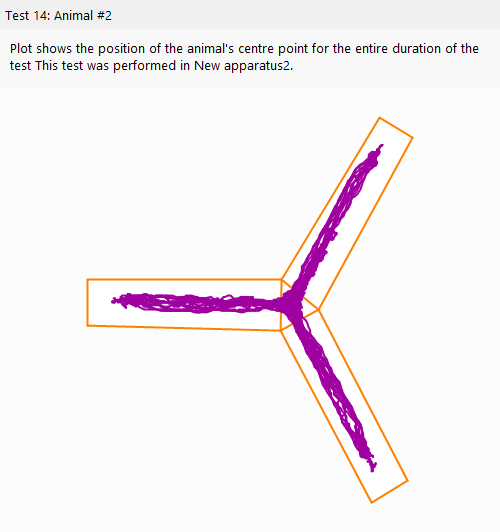

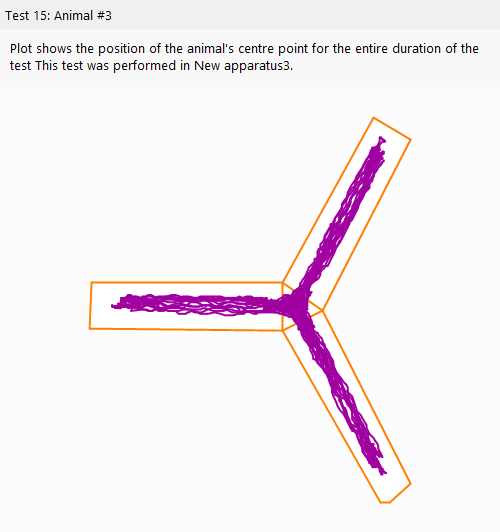

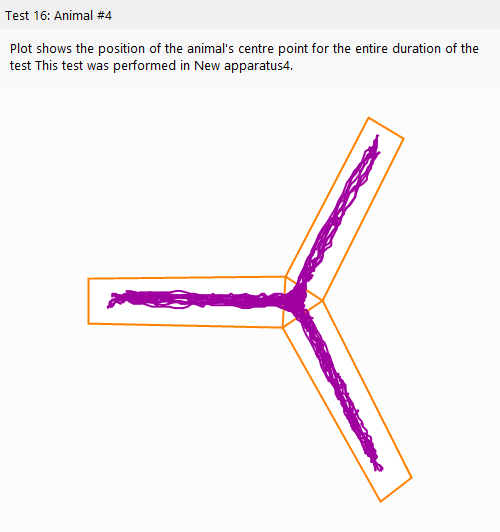

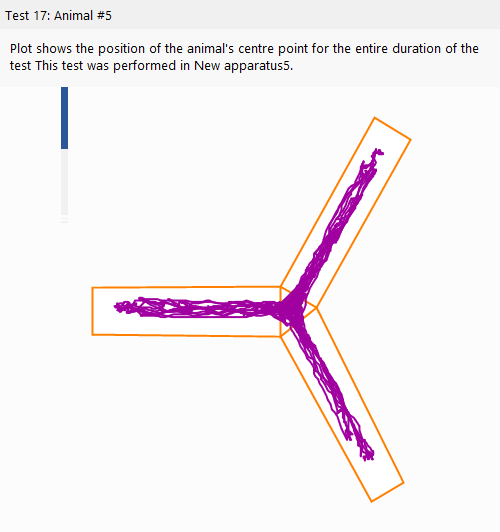

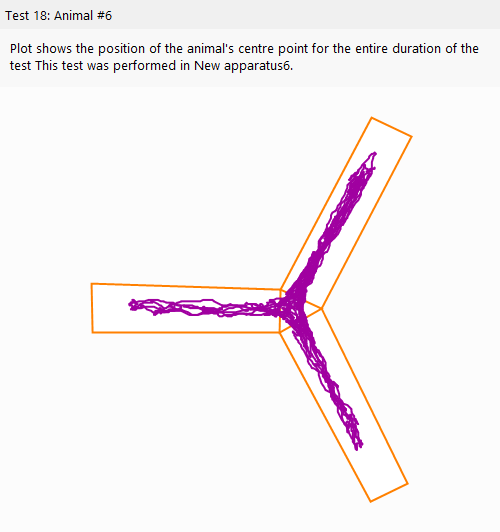

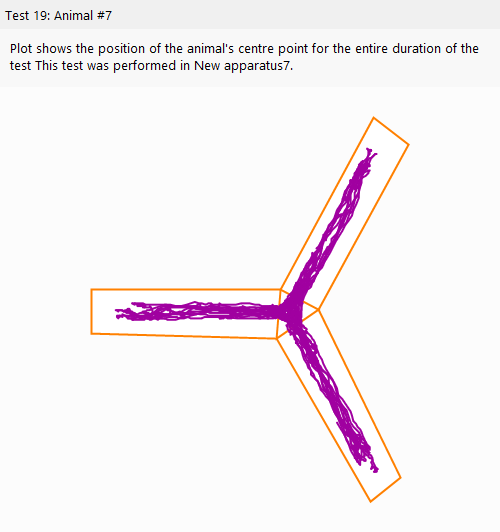

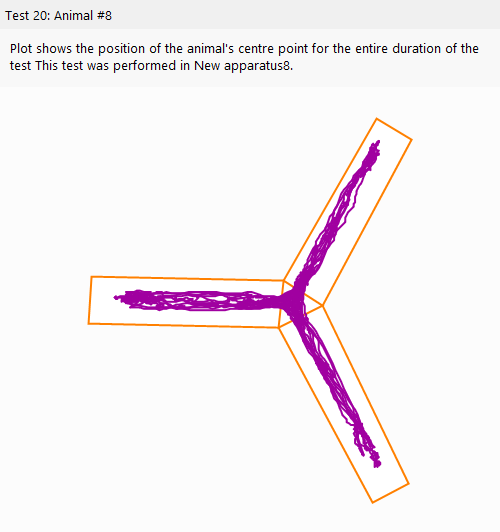

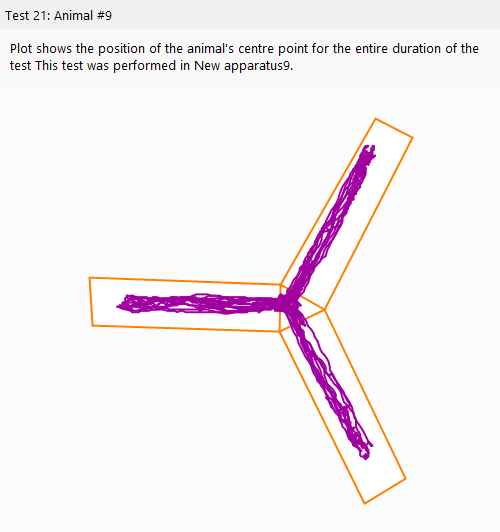

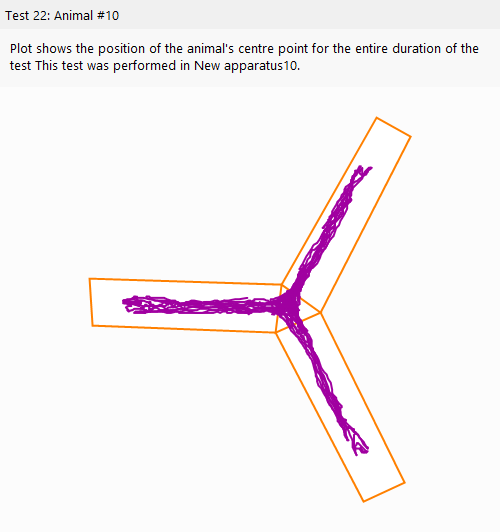

Supplement: Supplementary file 8 [file DataSheet2.zip › Behavior tests images/Y maze test/Y-maze-model-轨迹图.docx]

xsb-h


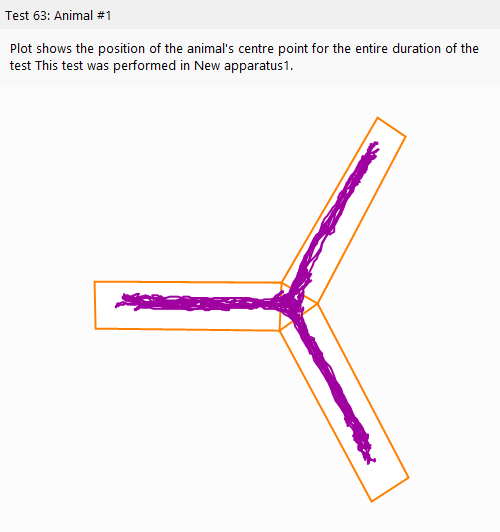

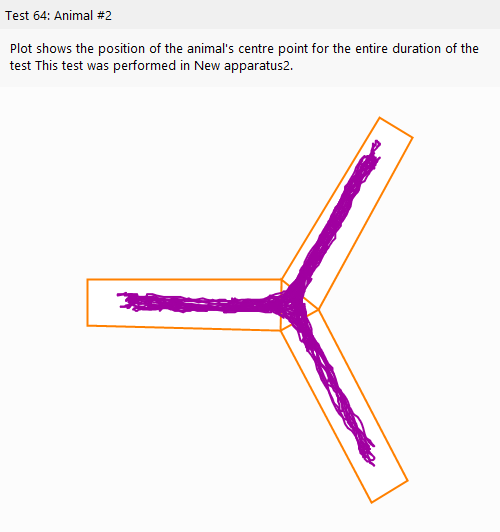

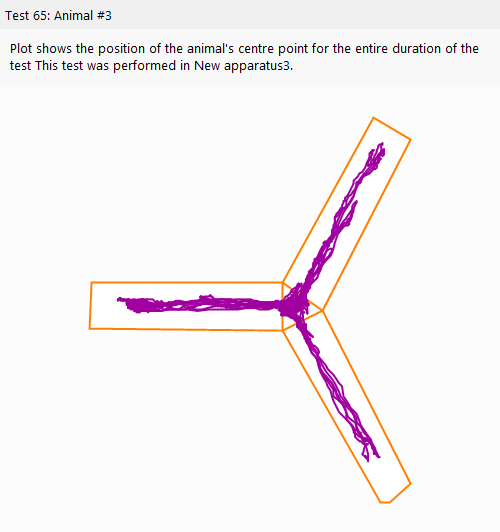

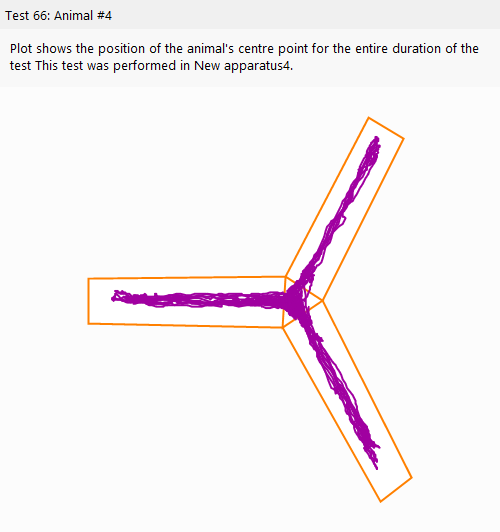

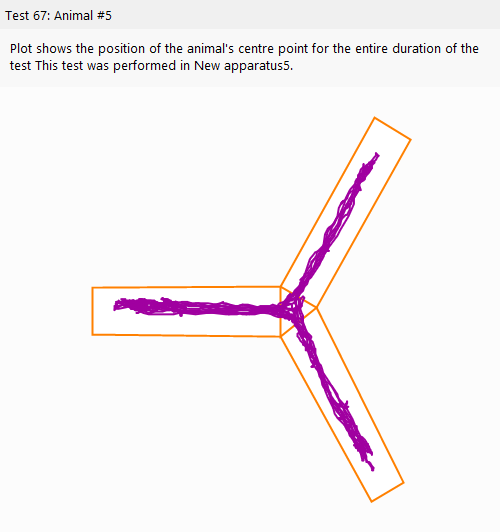

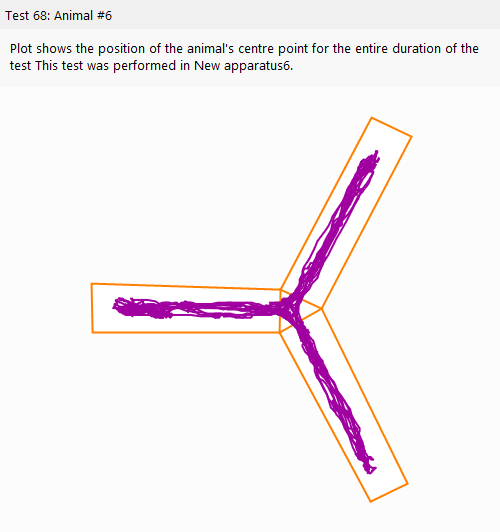

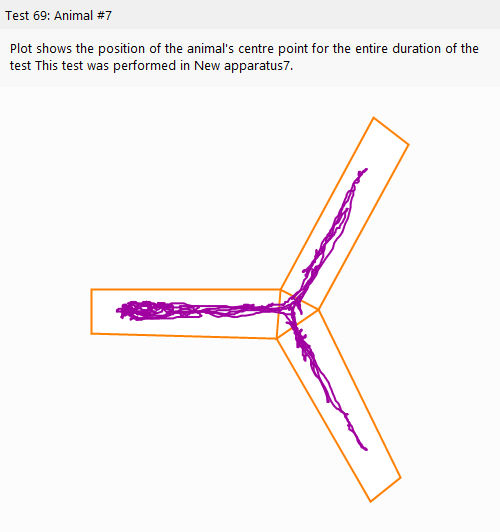

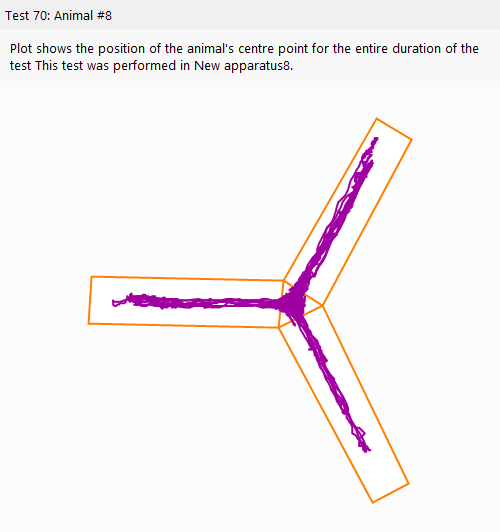

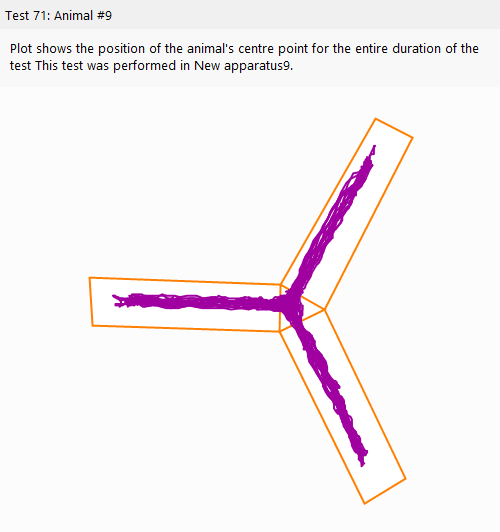

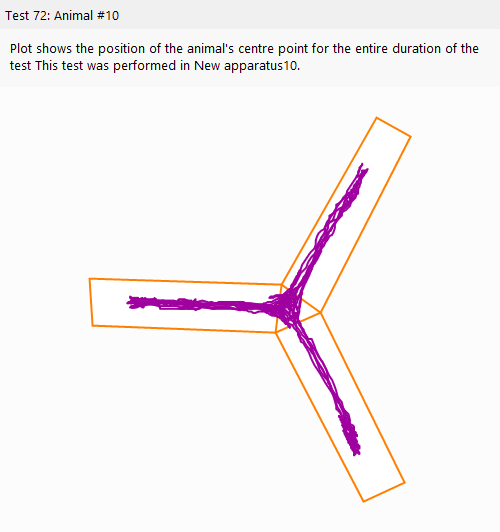

Supplement: Supplementary file 8 [file DataSheet2.zip › Behavior tests images/Y maze test/Y-maze-xsb-h-轨迹图.docx]

xsb-l


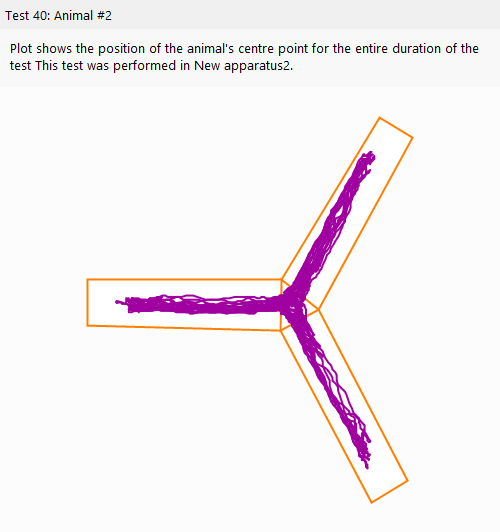

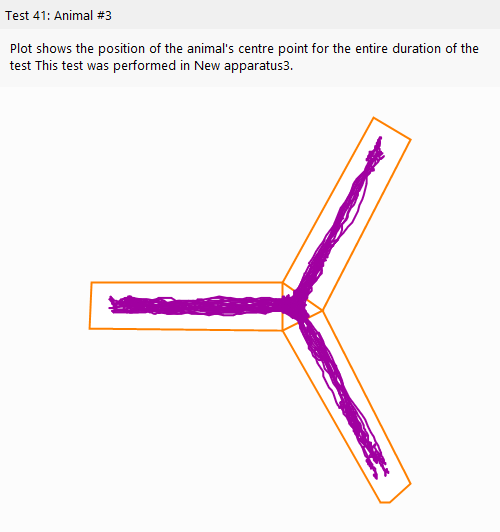

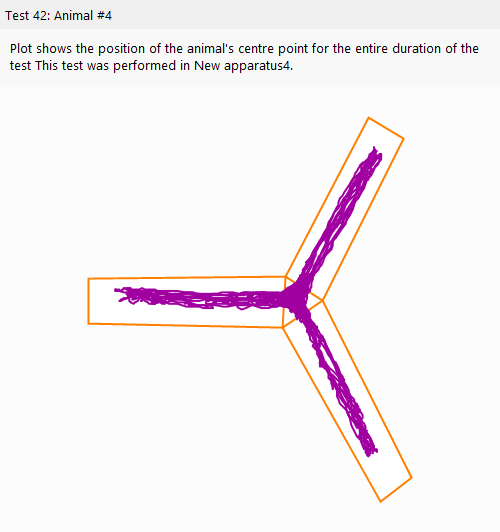

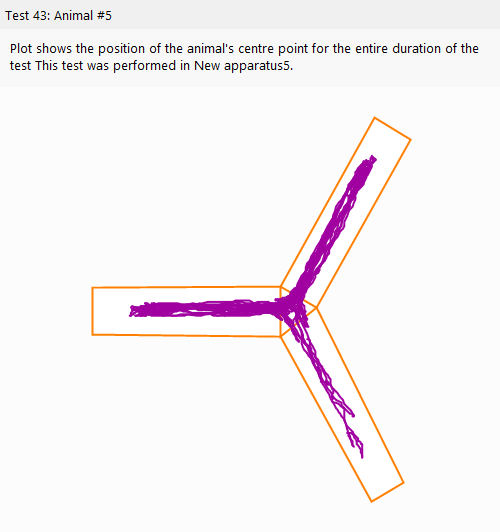

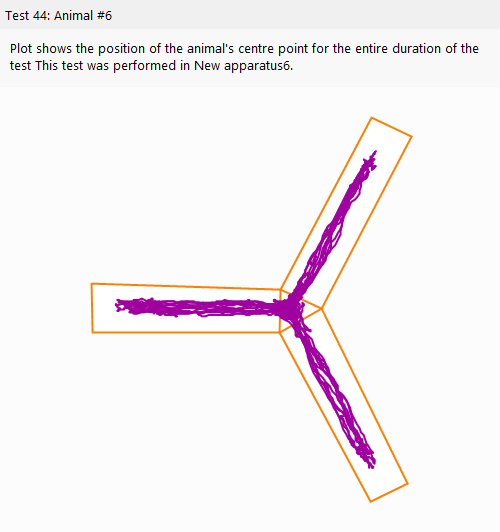

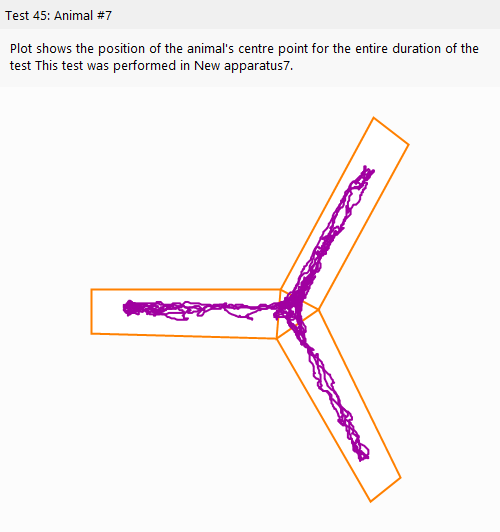

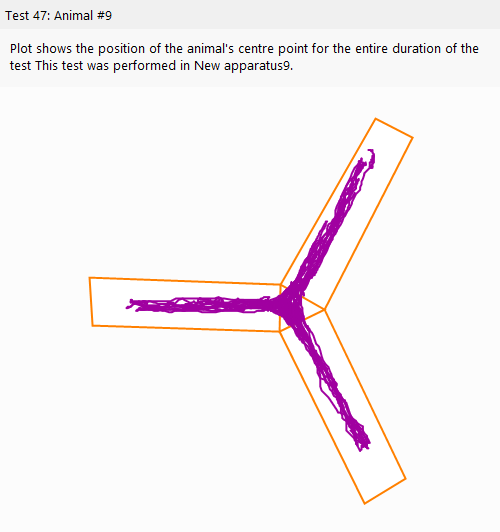

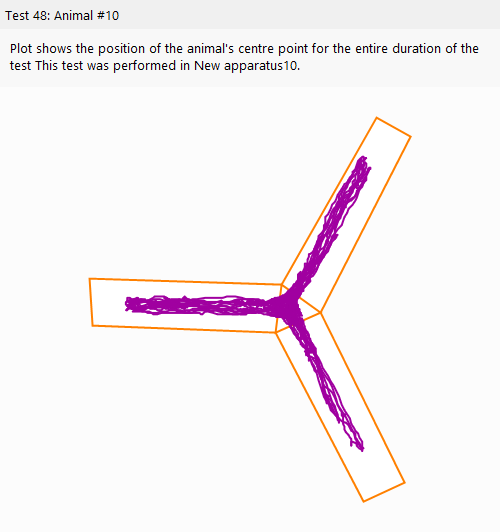

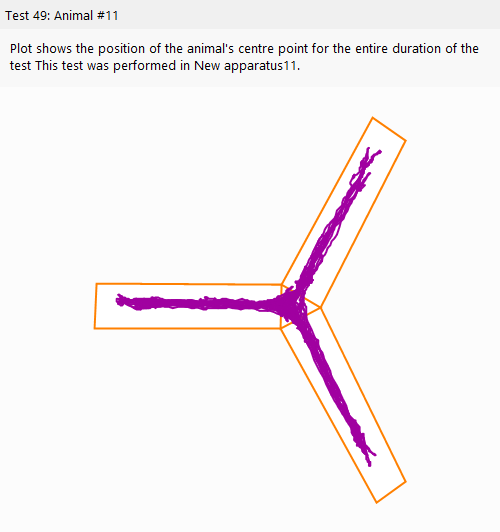

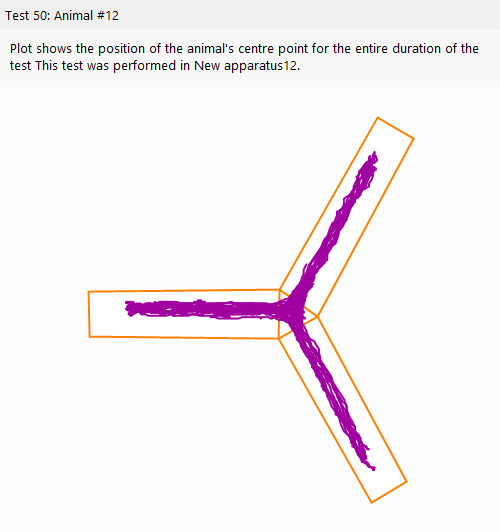

Supplement: Supplementary file 8 [file DataSheet2.zip › Behavior tests images/Y maze test/Y-maze-xsb-l-轨迹图.docx]

xsb-m


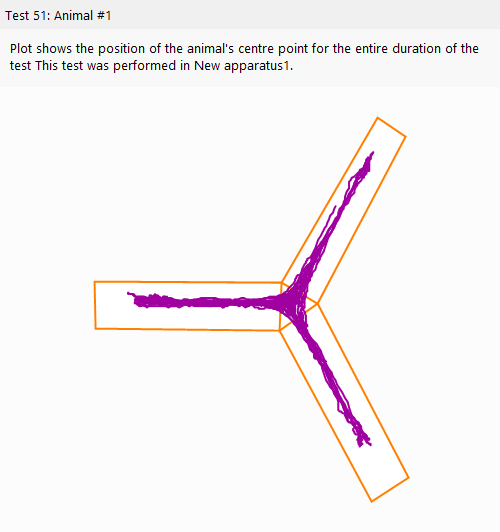

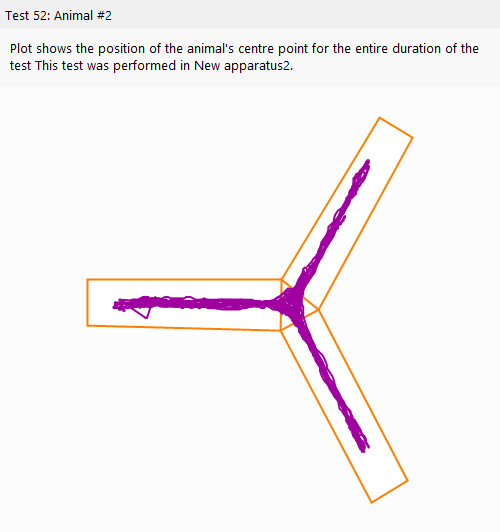

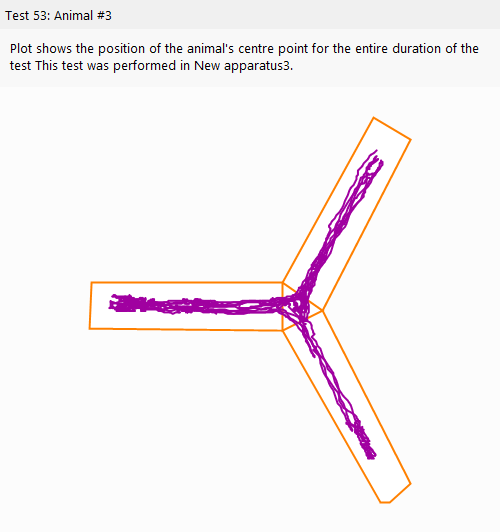

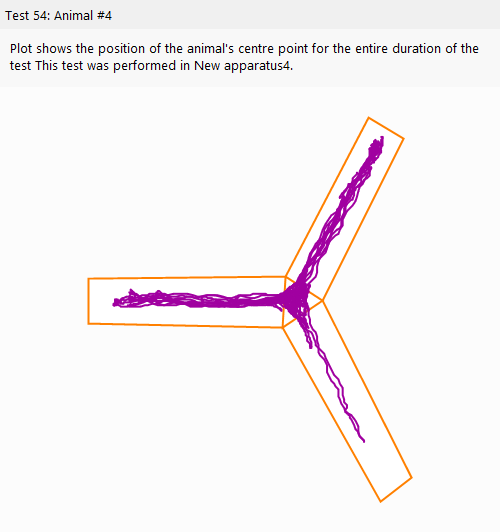

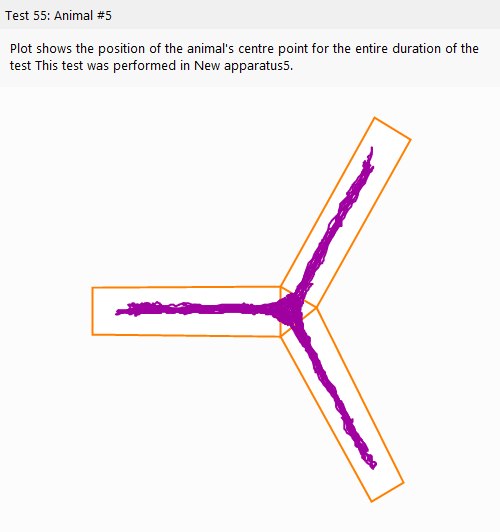

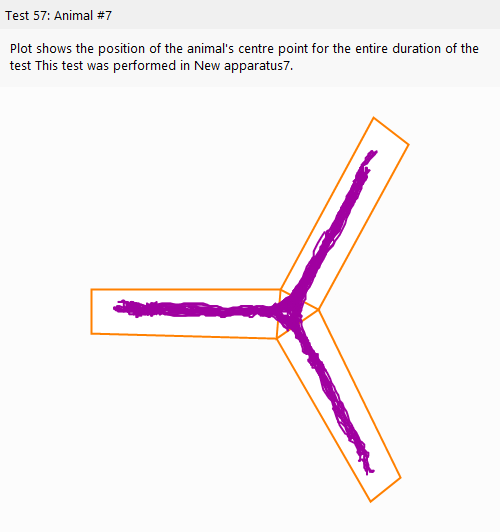

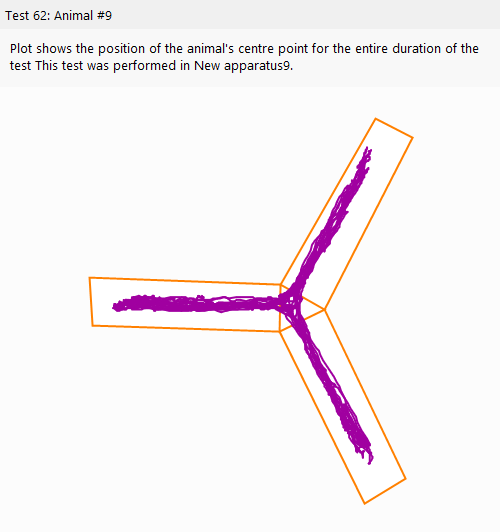

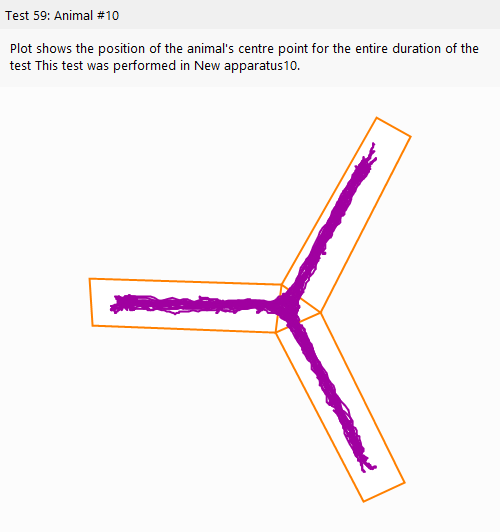

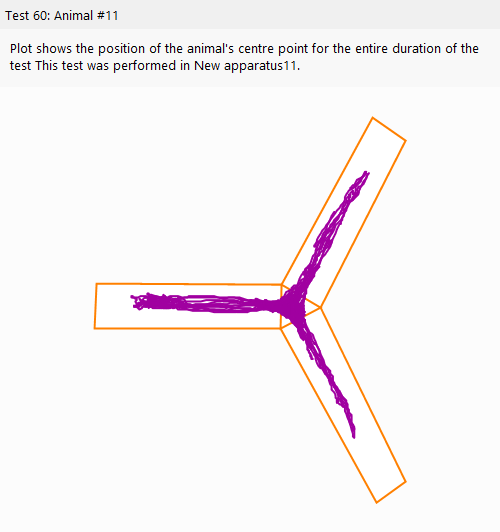

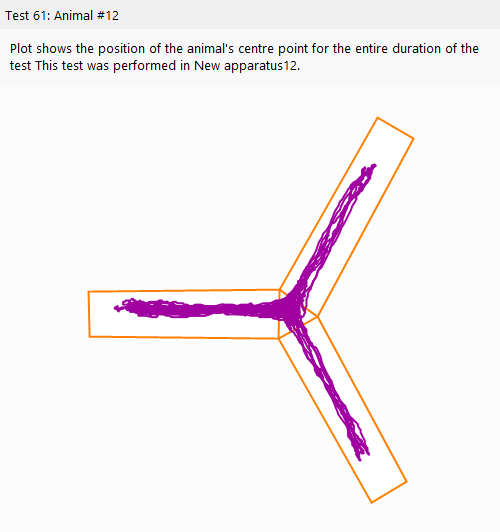

Supplement: Supplementary file 8 [file DataSheet2.zip › Behavior tests images/Y maze test/Y-maze-xsb-m-轨迹图.docx]

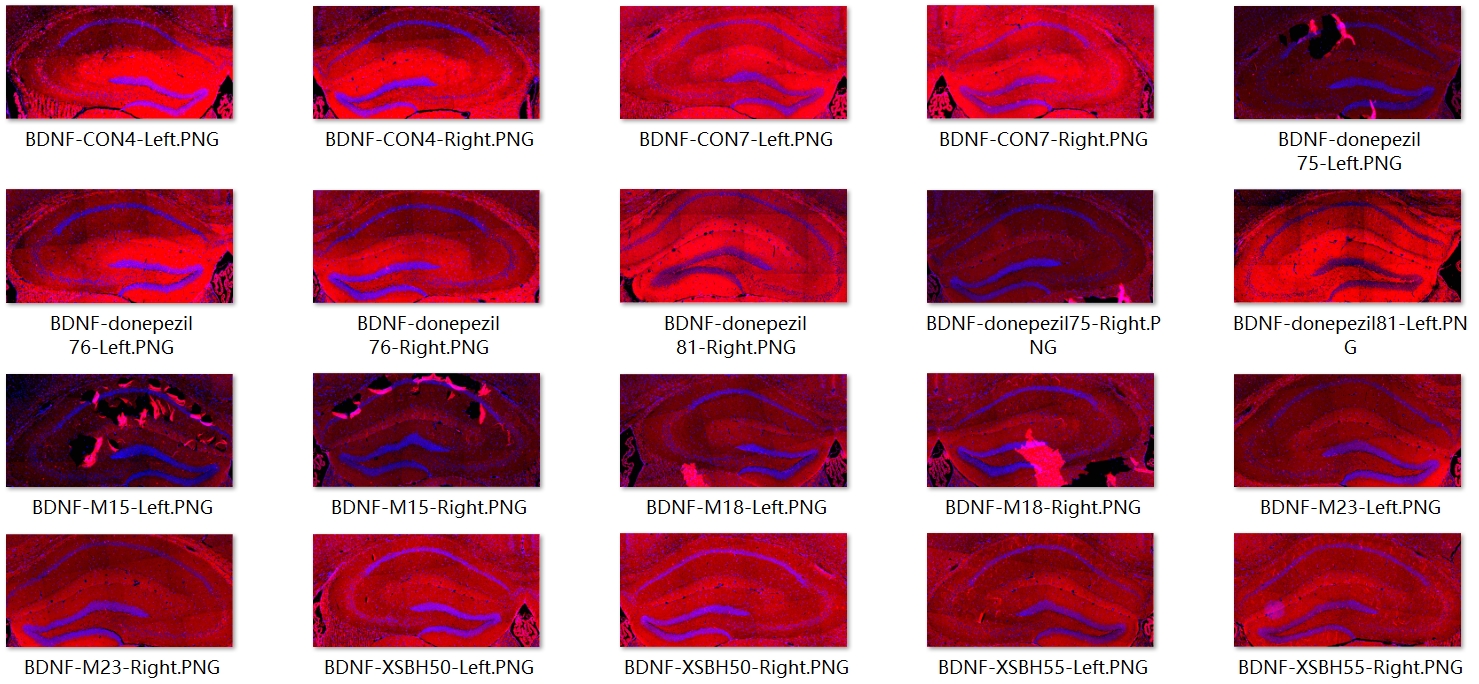

Supplement: Supplementary file 10 [file DataSheet5.zip › IF Staning/BDNF-1.png]

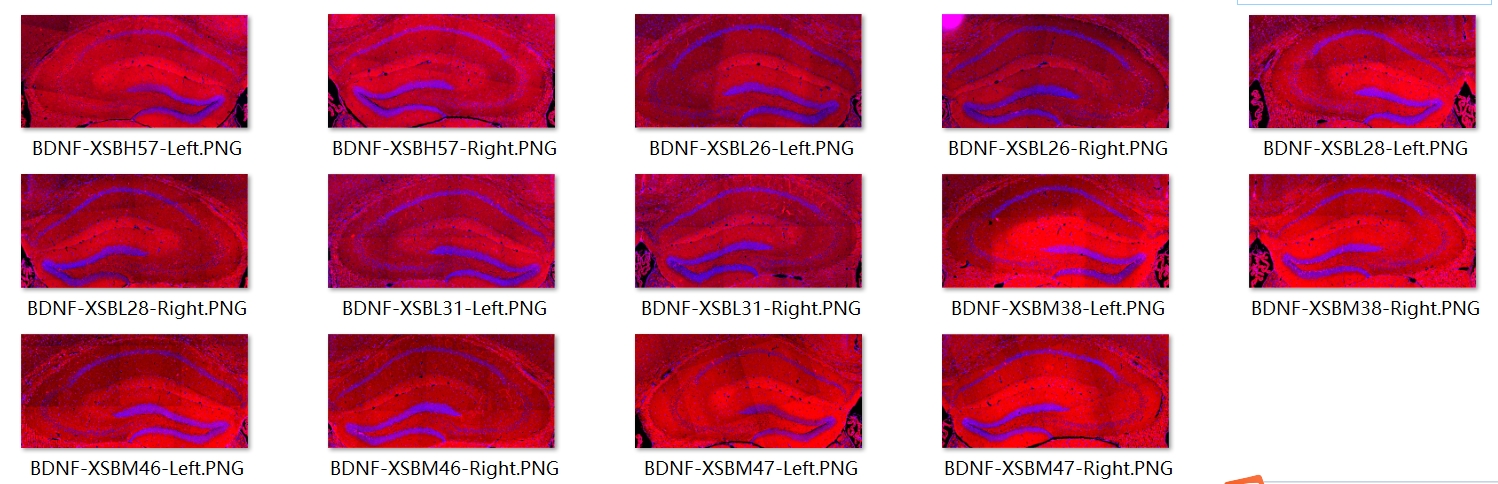

Supplement: Supplementary file 10 [file DataSheet5.zip › IF Staning/BDNF-2.png]

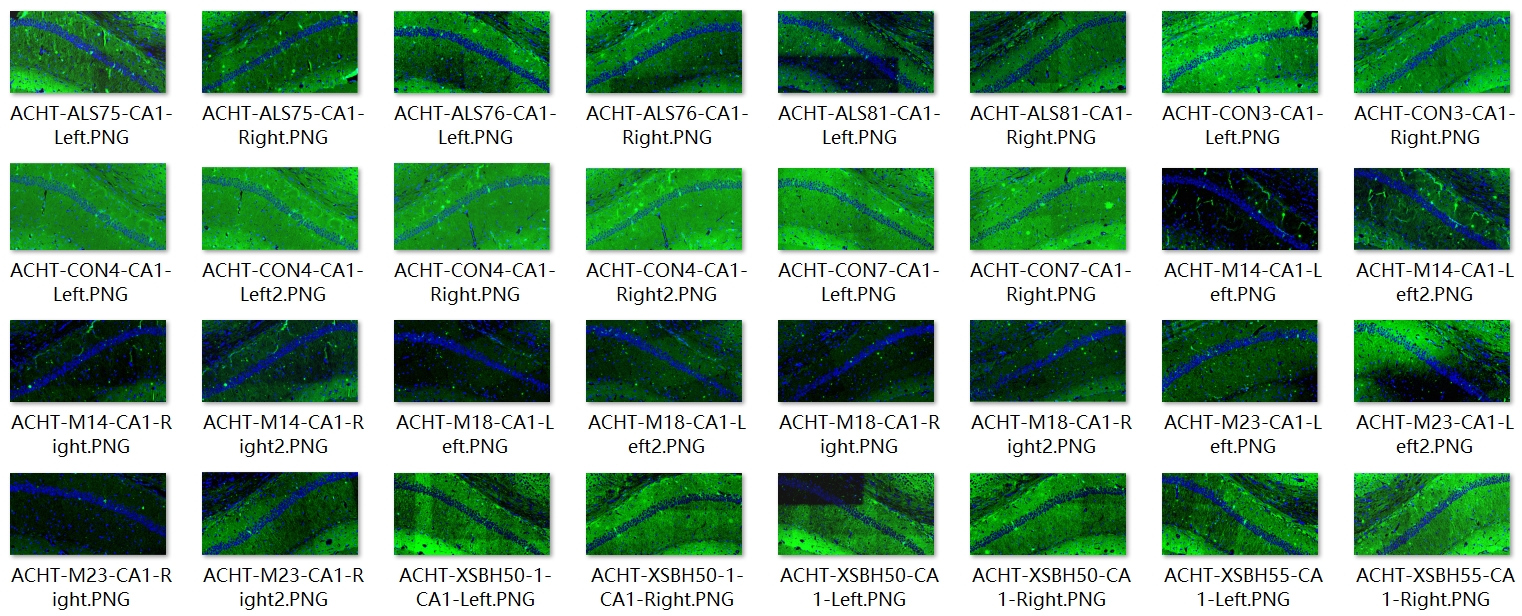

Supplement: Supplementary file 10 [file DataSheet5.zip › IF Staning/CHAT1-CA1-1.png]

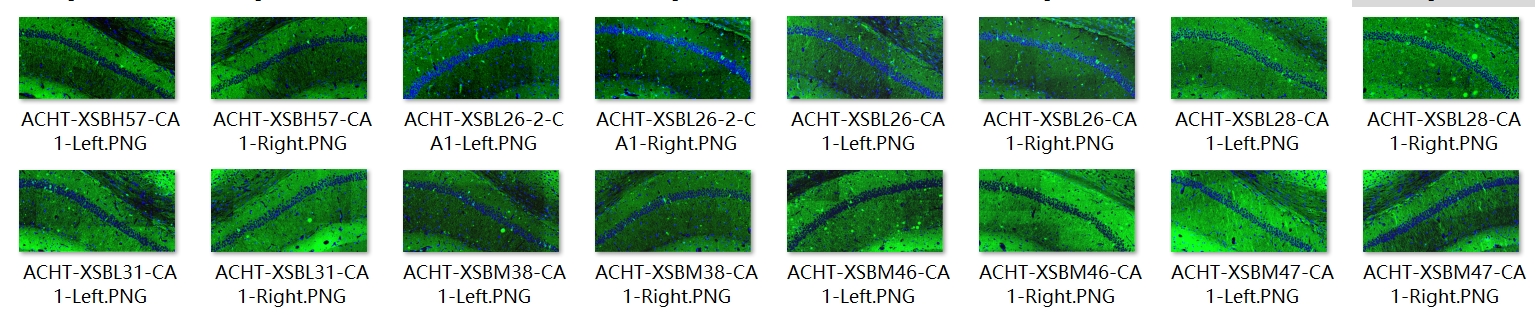

Supplement: Supplementary file 10 [file DataSheet5.zip › IF Staning/CHAT1-CA1-2.png]

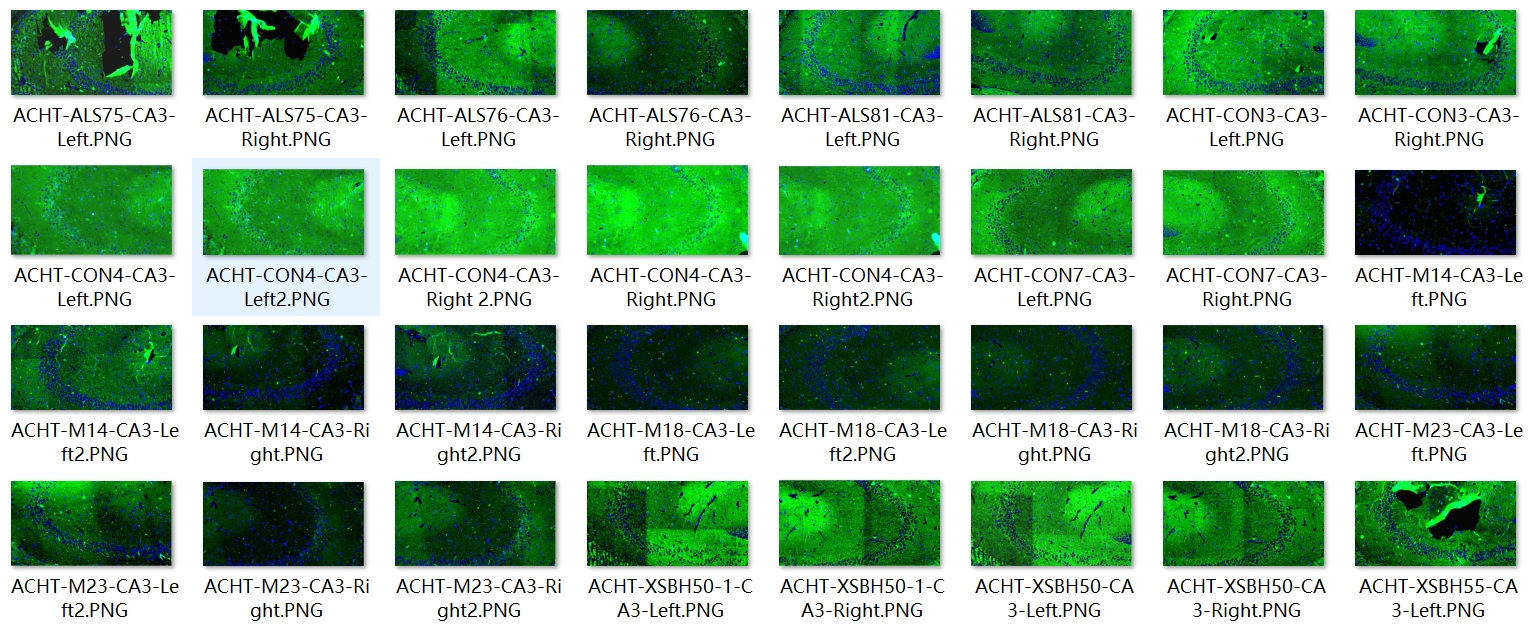

Supplement: Supplementary file 10 [file DataSheet5.zip › IF Staning/CHAT1-CA3-1.png]

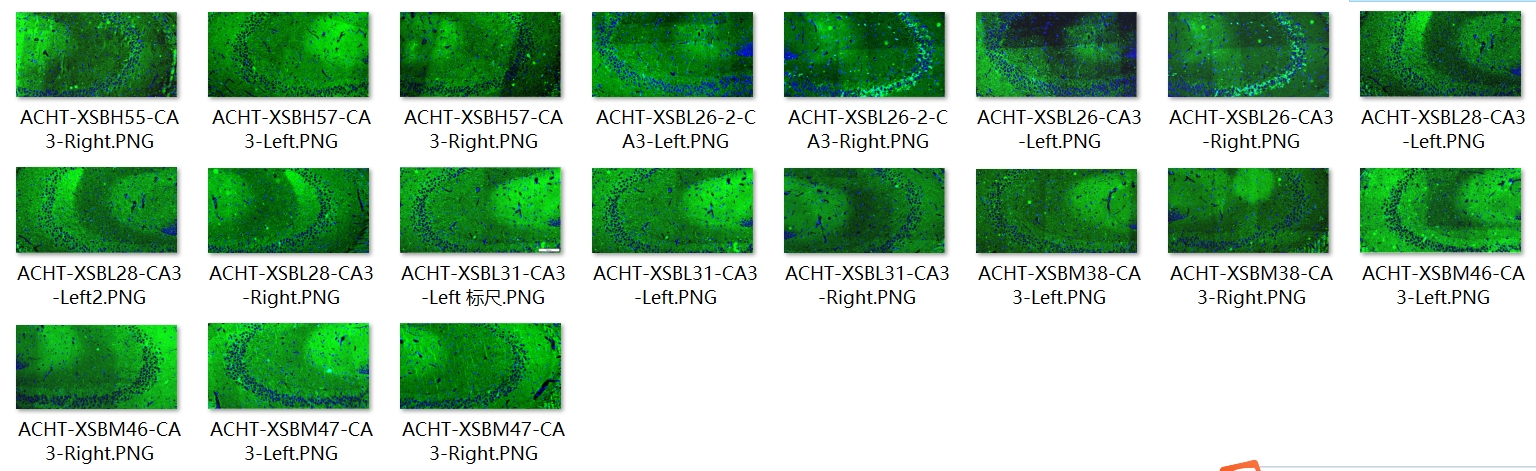

Supplement: Supplementary file 10 [file DataSheet5.zip › IF Staning/CHAT1-CA3-2.png]

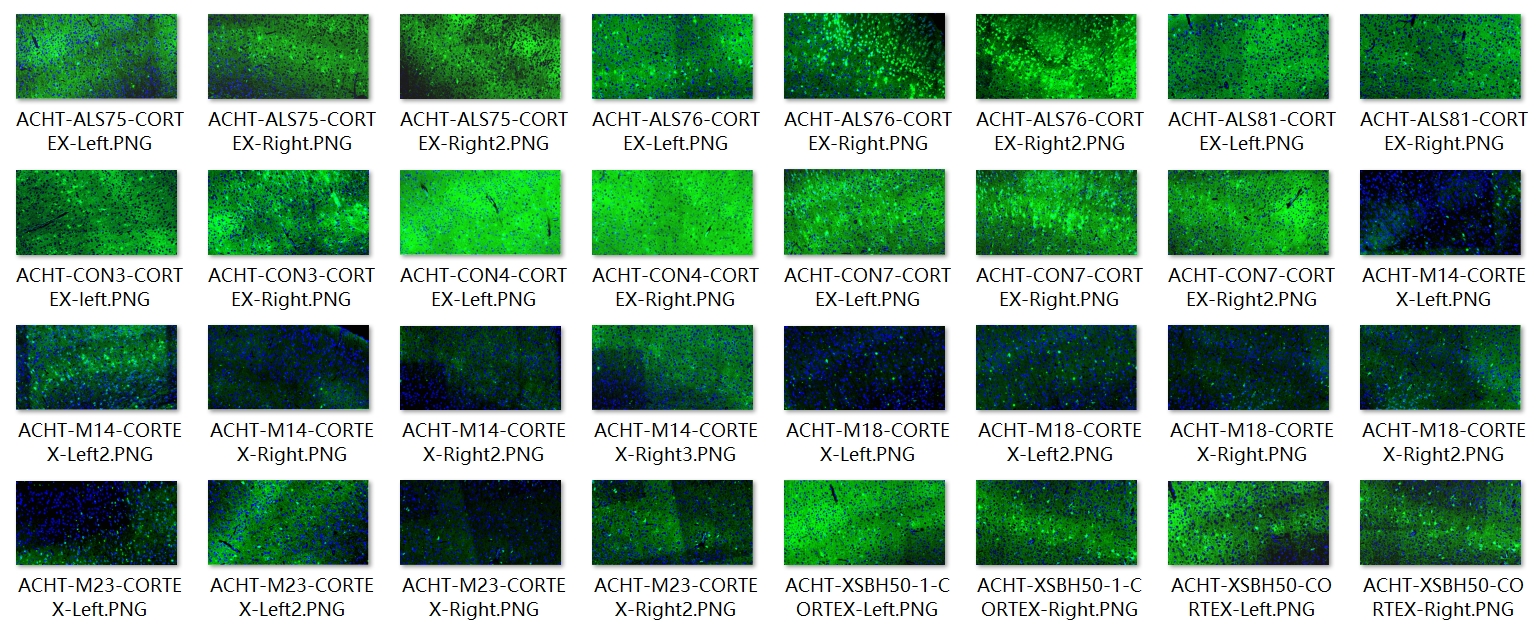

Supplement: Supplementary file 10 [file DataSheet5.zip › IF Staning/CHAT1-Cortex-1.png]

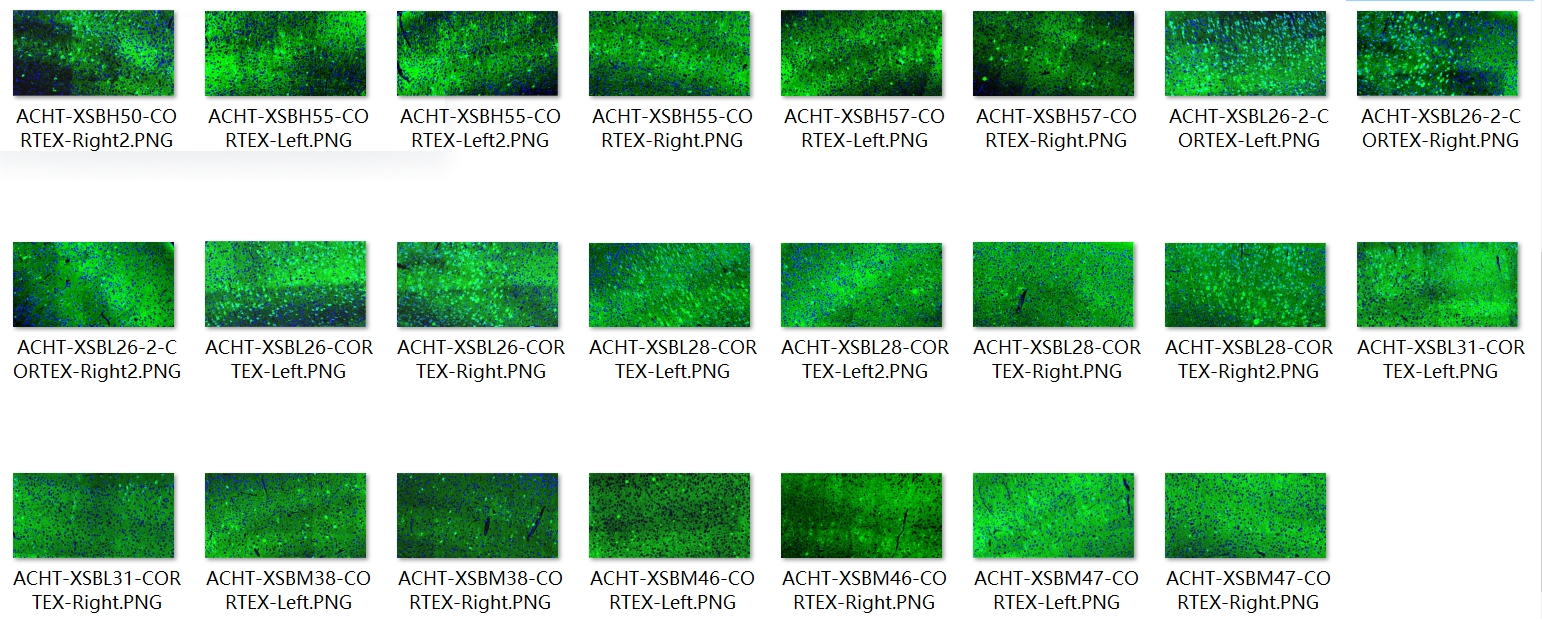

Supplement: Supplementary file 10 [file DataSheet5.zip › IF Staning/CHAT1-Cortex-2.png]

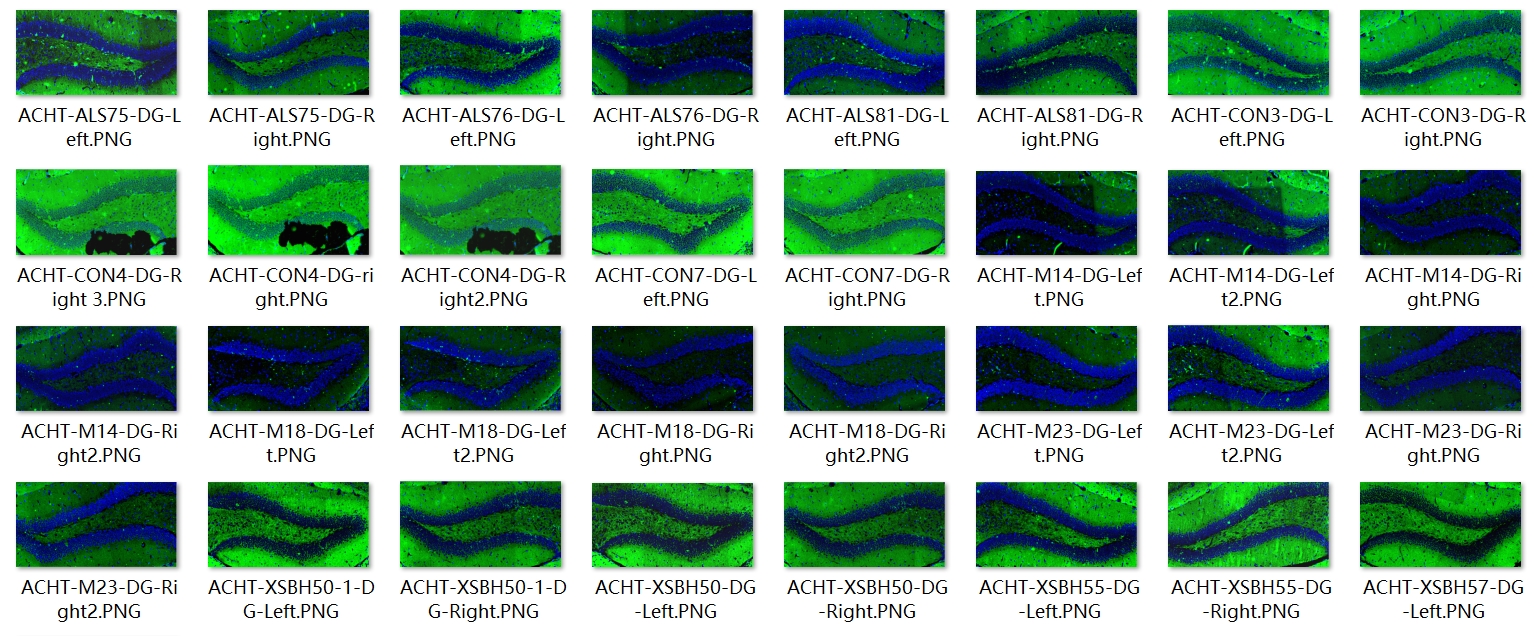

Supplement: Supplementary file 10 [file DataSheet5.zip › IF Staning/CHAT1-DG-1.png]

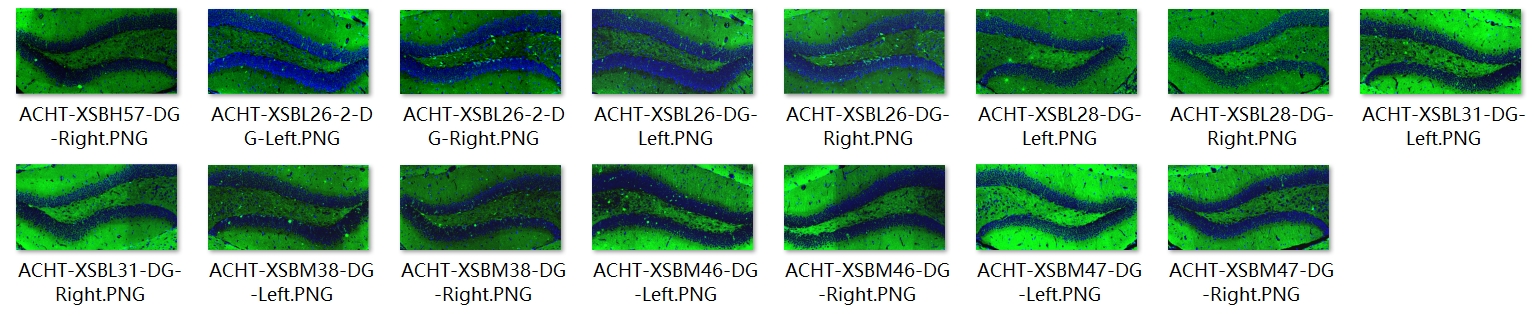

Supplement: Supplementary file 10 [file DataSheet5.zip › IF Staning/CHAT1-DG-2.png]

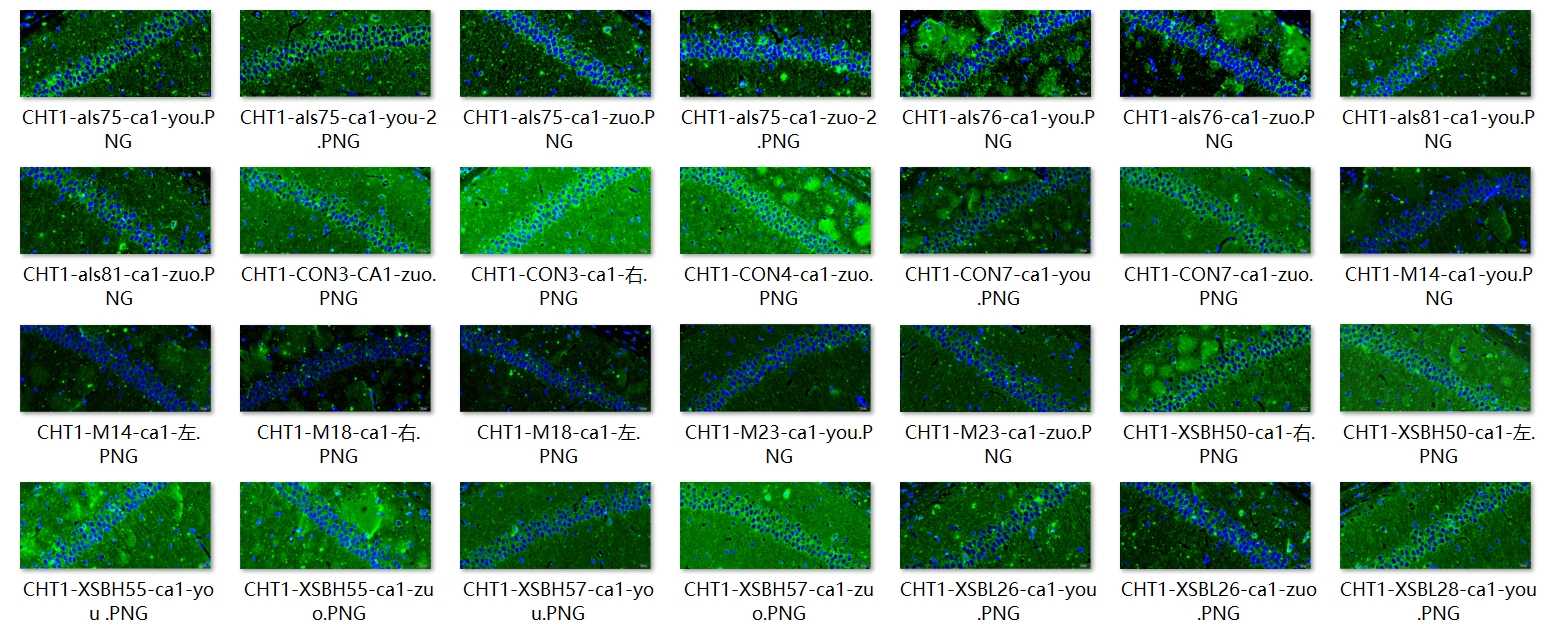

Supplement: Supplementary file 10 [file DataSheet5.zip › IF Staning/CHT1-CA1-1.png]

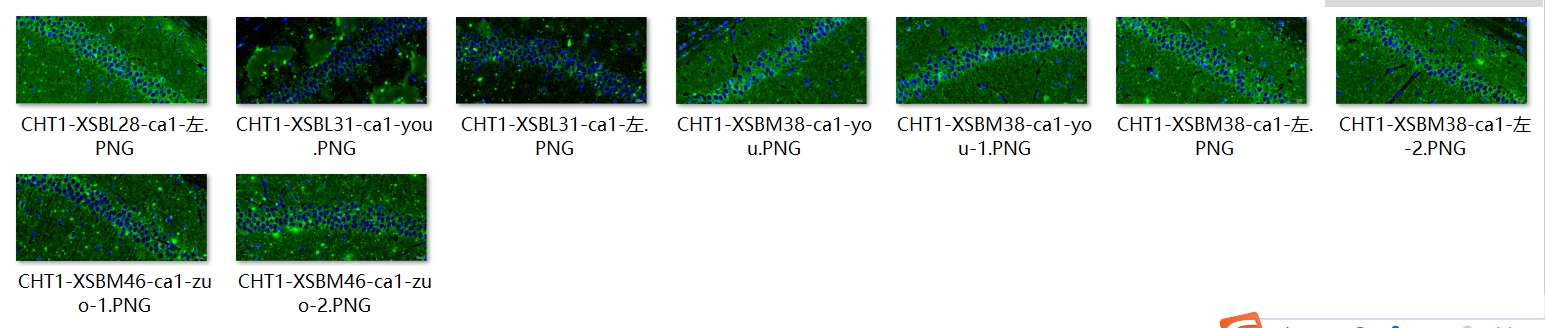

Supplement: Supplementary file 10 [file DataSheet5.zip › IF Staning/CHT1-CA1-2.png]

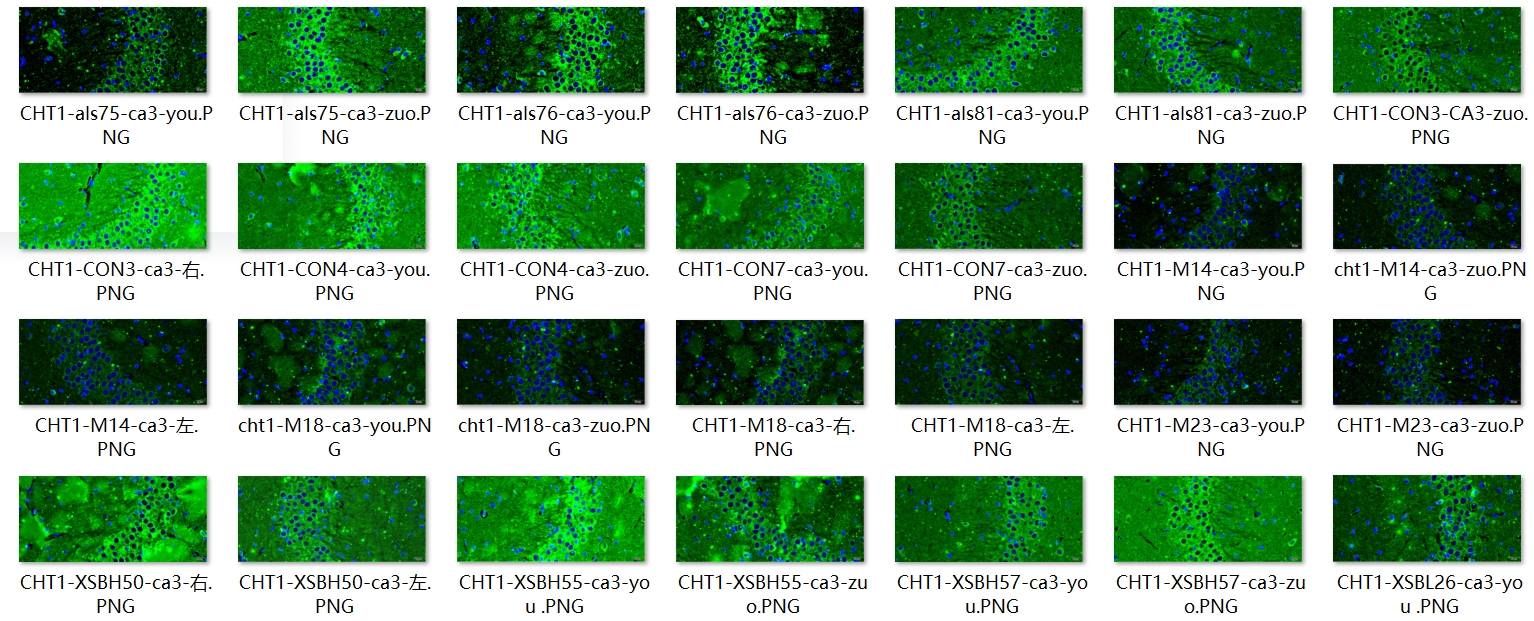

Supplement: Supplementary file 10 [file DataSheet5.zip › IF Staning/CHT1-CA3-1.png]

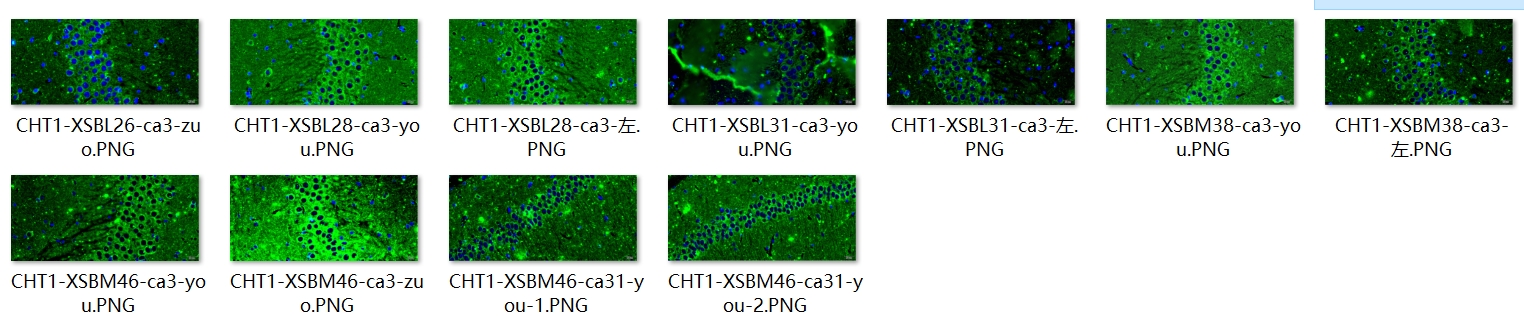

Supplement: Supplementary file 10 [file DataSheet5.zip › IF Staning/CHT1-CA3-2.png]

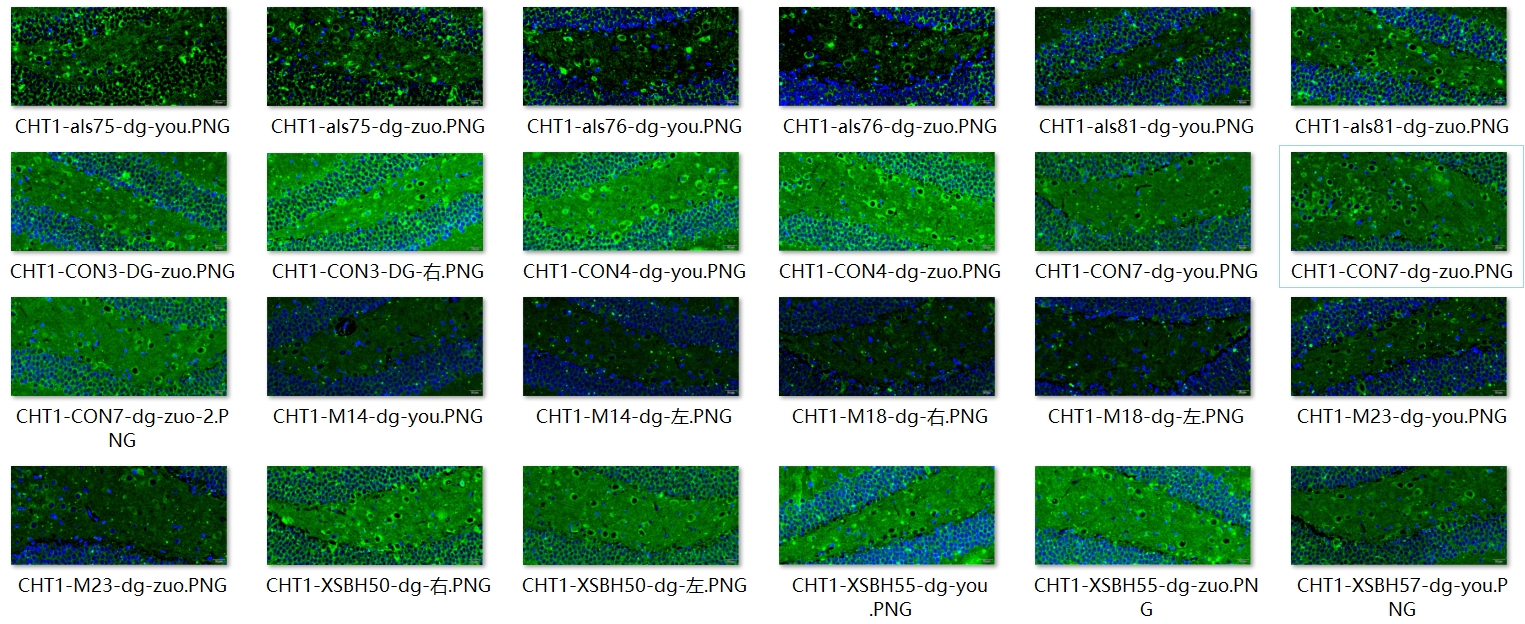

Supplement: Supplementary file 10 [file DataSheet5.zip › IF Staning/CHT1-DG-1.png]

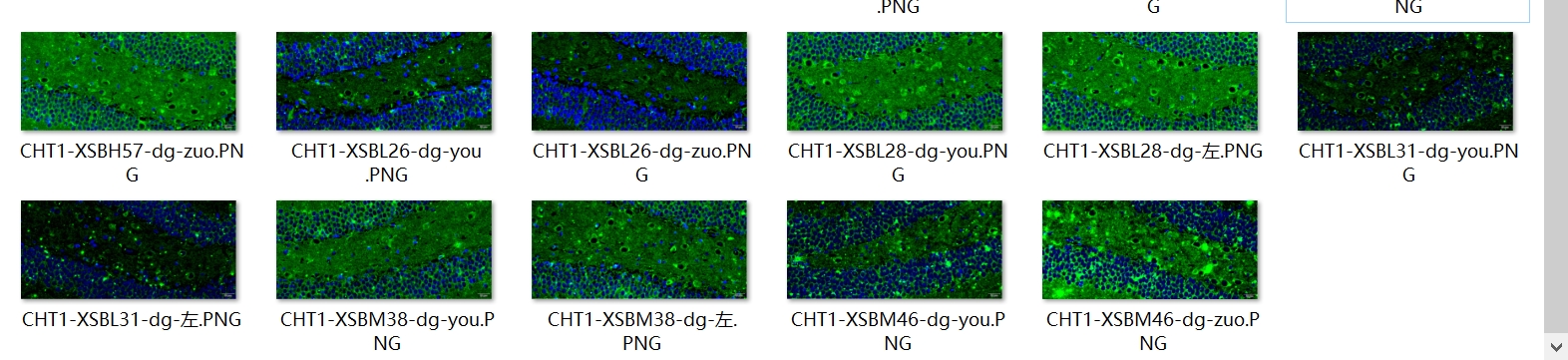

Supplement: Supplementary file 10 [file DataSheet5.zip › IF Staning/CHT1-DG-2.png]

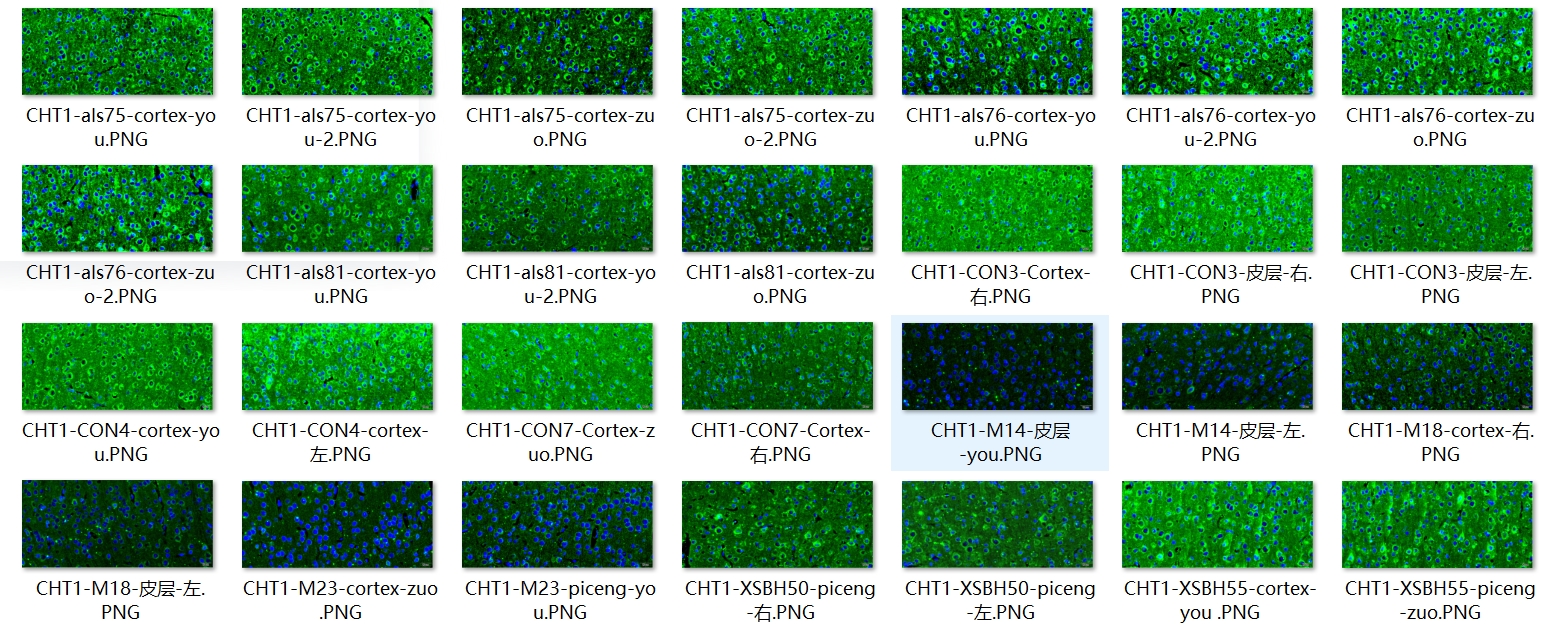

Supplement: Supplementary file 10 [file DataSheet5.zip › IF Staning/CHT1-cortex-1.png]

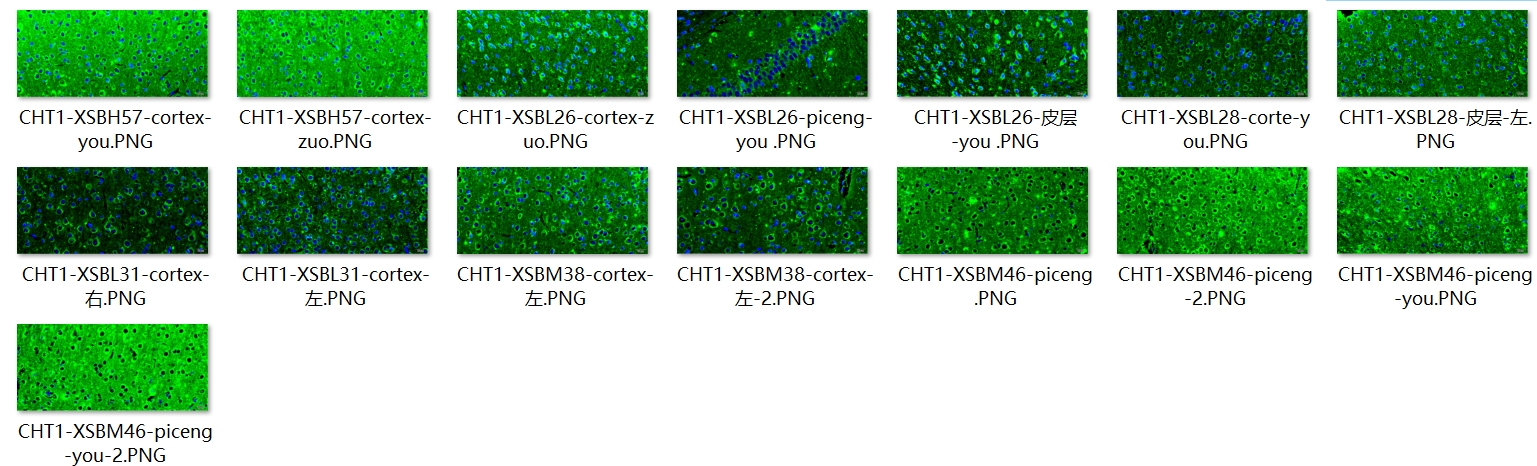

Supplement: Supplementary file 10 [file DataSheet5.zip › IF Staning/CHT1-cortex-2.png]

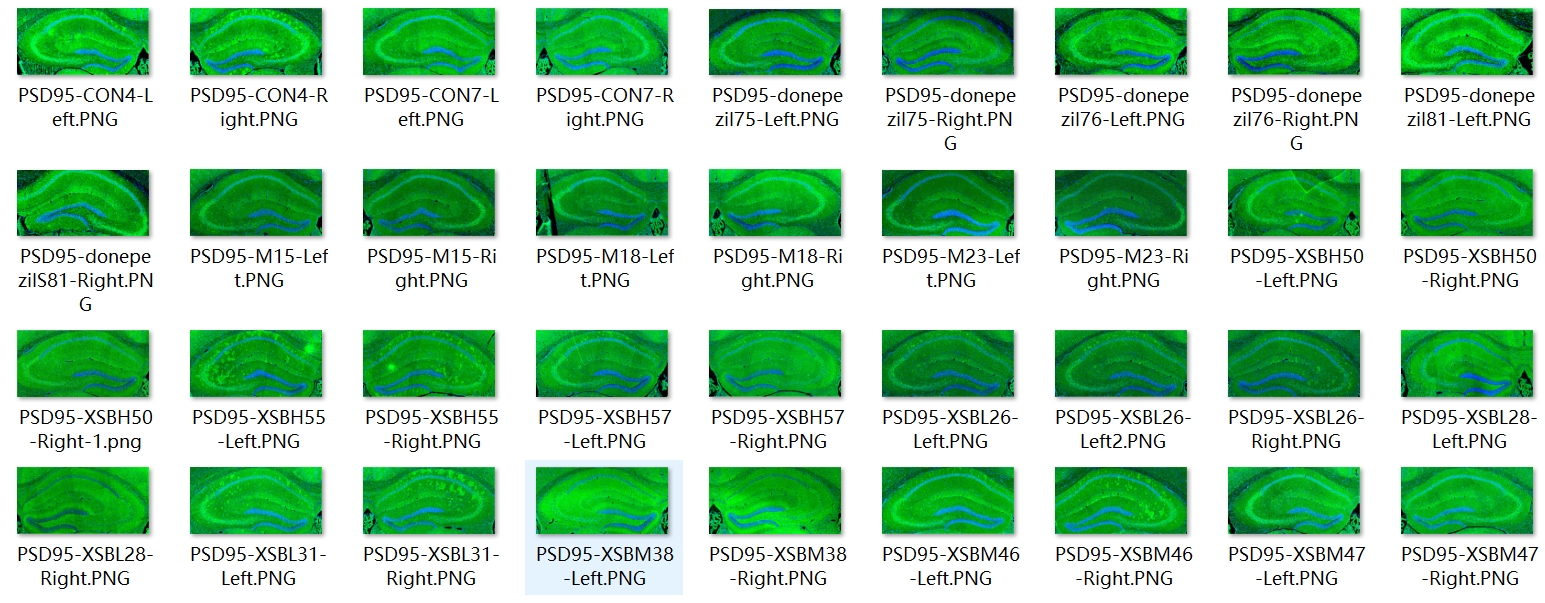

Supplement: Supplementary file 10 [file DataSheet5.zip › IF Staning/PSD-95.png]

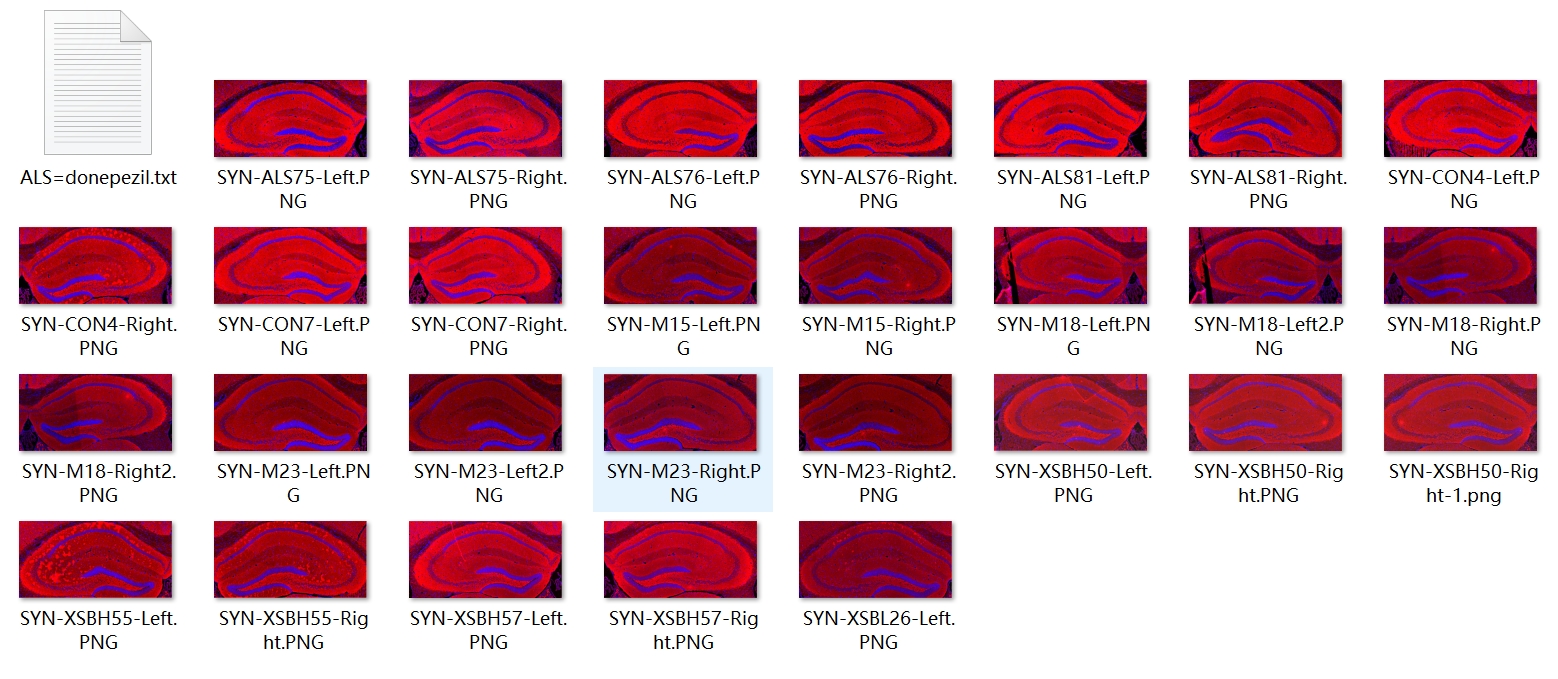

Supplement: Supplementary file 10 [file DataSheet5.zip › IF Staning/SYN.png]

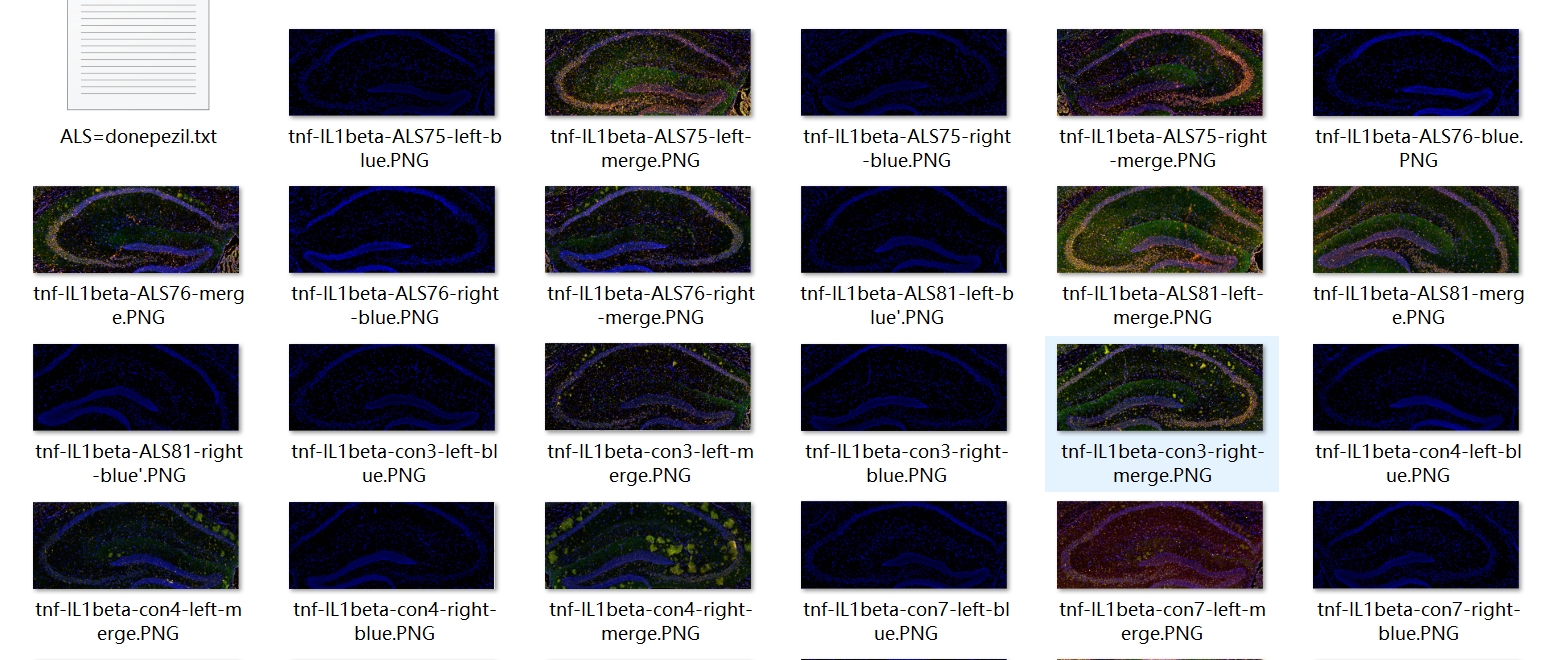

Supplement: Supplementary file 10 [file DataSheet5.zip › IF Staning/TNF-α-IL1β-1.png]

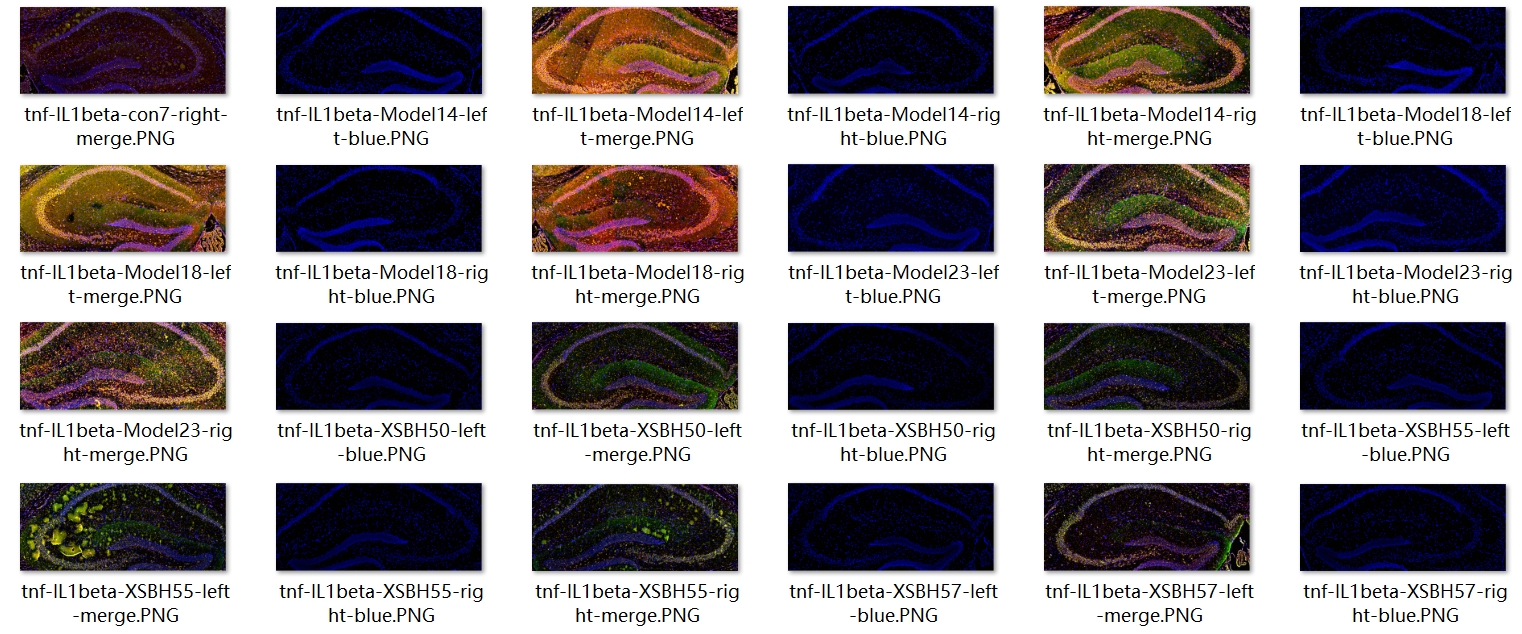

Supplement: Supplementary file 10 [file DataSheet5.zip › IF Staning/TNF-α-IL1β-2.png]

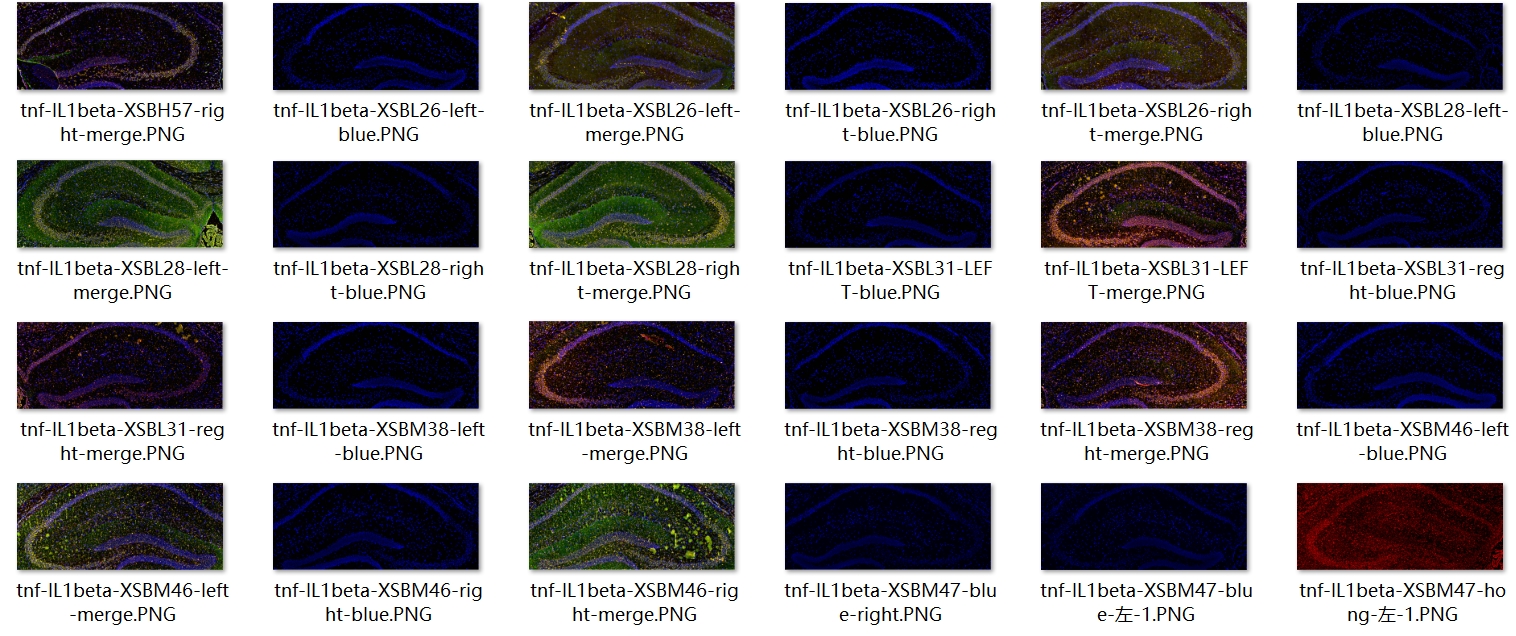

Supplement: Supplementary file 10 [file DataSheet5.zip › IF Staning/TNF-α-IL1β-3.png]

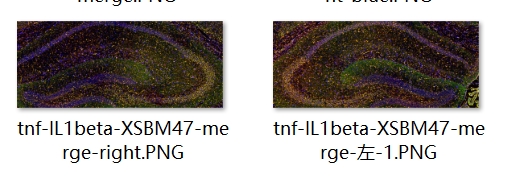

Supplement: Supplementary file 10 [file DataSheet5.zip › IF Staning/TNF-α-IL1β-4.png]

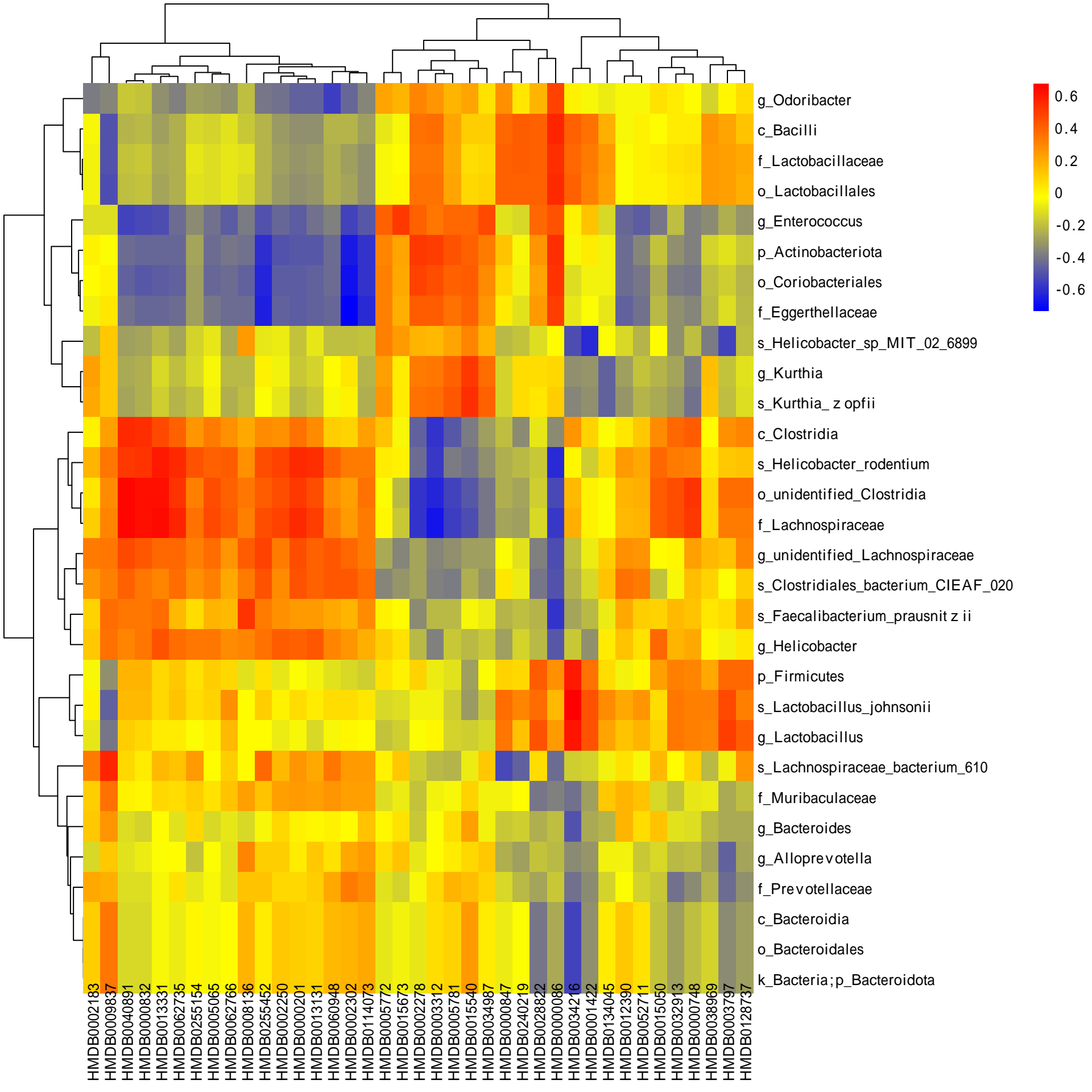

Supplement: Supplementary file 12 [file DataSheet7.zip › result.pdf]

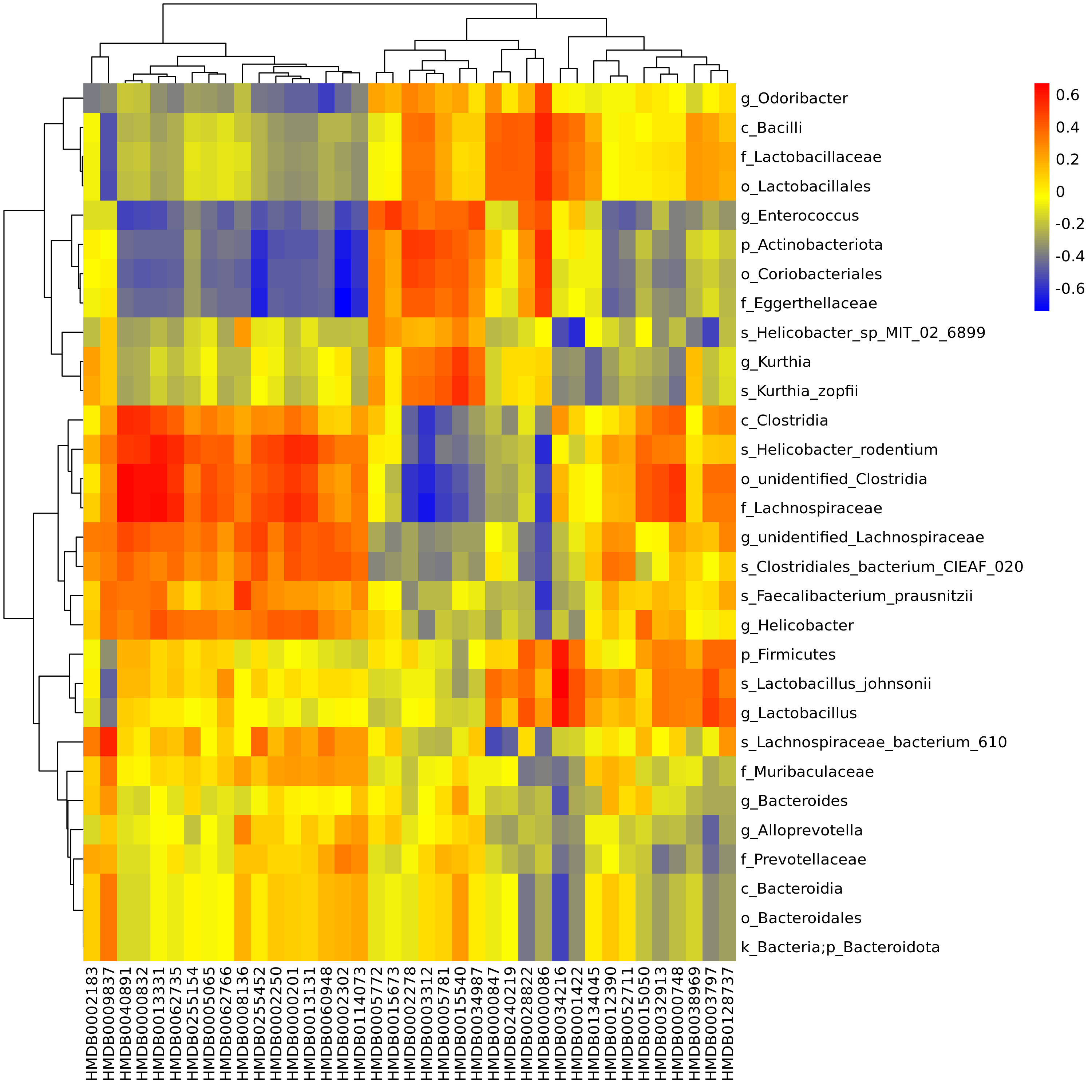

Supplement: Supplementary file 12 [file DataSheet7.zip › result.png]
