# Supplementary material for: Multi-omics reveal the neuroprotective mechanisms of Xinshubao tablet against scopolamine-induced cognitive dysfunction in mice
Source: Front Pharmacol. 2025 Jul 4;16:1596728. doi: 10.3389/fphar.2025.1596728 (PMC12271746; doi:10.3389/fphar.2025.1596728)
Supplement: Supplementary file 3 [file Supplementaryfile1.docx]

**Identification of chemical components in XSB**

The chemical profile of XSB was characterized using an ultra-high performance liquid chromatography (UPLC) coupled with quadrupole-time-of-flight mass spectrometer (UPLC-Q-TOF MS/MS) and the main constitutes of XSB were quantitatively analyzed using the standard curve method. Chromatography was performed using an Dionex™ UltiMate™ 3000 system (ThermoFisher Scientific, USA). The separation was carried out on a Waters ACQUITY UPLC HSS T3 column (1.8 μm，2.1 mm×100 mm）at 40 ℃. The mobile phase consisted of 0.1% formic acid–water (solvent A) and acetonitrile (solvent B). The linear gradient elution program was as follows: 0-1 min, 98%-98% B; 1-41 min, 0% B, 41-50 min, 100% B, 50-52 min, 98% B. The injection volume was 10 μl, and the flow rate was 0.3 ml/min. The high-mass resolution experiments were performed on Q Exactive™ Q-TOF (ThermoFisher Scientific, USA) system equipped with an electrospray ionization interface. Both negative and positive ionization modes were performed. The mass range was scanned from 100 to 1500 Da. The raw data were acquired and processed by Xcalibur 2.2 SP1.48 system and analyzed using MassLynx 4.1 software. The total ion chromatogram (TIC) of mixed standard solution and XSB were as shown in supplementary Fig. 1. The chemical components in XSB were as shown in supplementary table 1-3, and the quantitative analysis primary chemical components of XSB were as shown in supplementary table 4.


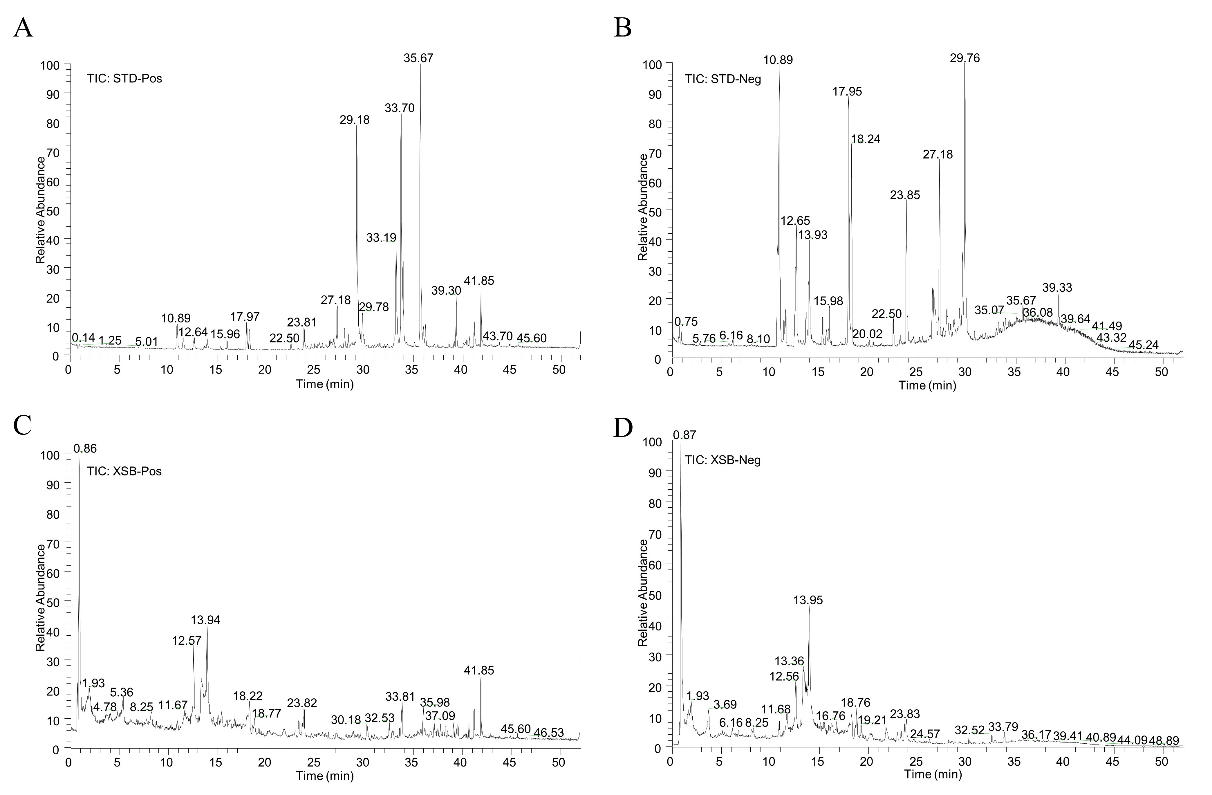


Supplementary Fig. 1 Total ion chromatogram (TIC) of mixed standard compounds and XSB. A: TIC of mixed standard compounds in positive-ion mold; B: TIC of mixed standard compounds in negative-ion mold; A: TIC of XSB in positive-ion mold; A: TIC of XSB in negative-ion mold.

Supplementary Table 1 Identification of chemical components in XSB.

| No. | RT (min) | Name | Formula | m/z | Adducts | Error  (ppm) | Instensity | Origin |
| --- | --- | --- | --- | --- | --- | --- | --- | --- |
| 1 | 1.93 | Citric acid | C6H8O7 | 191.0188 | M-H | -4.6 | 11359544.20 | Shanzha |
| 2 | 2.32 | 2-O,3-O-[4,4',5,5',6,6'-Hexahydroxy-1,1'-biphenyl-2,2'-diylbis(carbonyl)]-beta-D-glucopyranose | C20H18O14 | 481.0623 | M-H | -0.1 | 75729.44 | Baishao |
| 3 | 3.31 | 1-Galloyl-β-D-glucose | C13H16O10 | 331.0671 | M-H | 0.2 | 484962.43 | Shanzha |
| 4 | 3.41 | eleutheroside C | C8H16O6 | 207.0867 | M-H | -3.4 | 167040.20 | Ciwujia |
| 5 | 4.59 | Shanzhiside methyl ester | C17H26O11 | 405.1400 | M-H | -0.6 | 1522866.50 | Danshen |
| 6 | 4.89 | Theogallin | C14H16O10 | 343.0671 | M-H | 0.1 | 38106.70 | Baishao |
| 7 | 5.21 | beta-Glucogallin | C13H16O10 | 331.0673 | M-H | 0.7 | 1029226.92 | Baishao |
| 8 | 6.16 | danshensu | C9H10O5 | 197.0448 | M-H | -3.7 | 2075698.00 | Danshen |
| 9 | 7.55 | Vanillic Acid Glucoside | C14H18O9 | 329.0880 | M-H | 0.7 | 507863.30 | Ciwujia |
| 10 | 7.76 | Strictinin | C27H22O18 | 633.0738 | M-H | 0.7 | 159896.27 | Baishao |
| 11 | 8.25 | dimethyl citrate | C8H12O7 | 219.0505 | M-H | -2.4 | 5783070.09 | Shanzha |
| 12 | 9.04 | Ethyl gallate | C9H10O5 | 197.0448 | M-H | -3.8 | 17030.22 | Baishao |
| 13 | 9.74 | Oxypaeoniflora | C23H28O12 | 495.1508 | M-H | 0.1 | 4789148.95 | Baishao |
| 14 | 10.89 | chlorogenic acid | C16H18O9 | 353.0877 | M-H | -0.2 | 10555079.68 | Ciwujia |
| 15 | 11.77 | (Z)-3-[2-[(E)-2-(3,4-dihydroxyphenyl)vinyl]-3,4-dihydroxy-phenyl]acrylic acid | C17H14O6 | 313.0717 | M-H | -0.1 | 3774.30 | Danshen |
| 16 | 11.87 | Eleutheroside B1 | C17H20O10 | 383.0982 | M-H | -0.3 | 18044.44 | Ciwujia |
| 17 | 12.57 | albiflorin | C23H28O11 | 479.1556 | M-H | -0.6 | 4448149.23 | Baishao |
| 18 | 12.66 | Neochlorogenic acid | C16H18O9 | 353.0877 | M-H | -0.4 | 64638.97 | Shanzha |
| 19 | 12.80 | 1,2,6-Trigalloyl-beta-D-glucopyranose | C27H24O18 | 635.0893 | M-H | 0.5 | 819935.55 | Baishao |
| 20 | 13.33 | 4-Hydroxy-3-methoxycinnamaldehyde | C10H10O3 | 179.0700 | M+H | -1.7 | 8467251.64 | Ciwujia |
| 21 | 13.94 | Benzyl methyl ether | C8H10O | 123.0804 | M+H | 0.0 | 1138393.75 | Shanzha |
| 22 | 13.94 | eugenol | C10H12O2 | 165.0908 | M+H | -1.3 | 1645438.48 | Yujin |
| 23 | 13.94 | (Z)-p-Methoxycinnamic acid | C10H10O3 | 179.0700 | M+H | -1.6 | 14445462.57 | Yujin |
| 24 | 14.24 | 1,2,3-Tri-O-galloyl-beta-D-glucose | C27H24O18 | 635.0898 | M-H | 1.3 | 85152.36 | Baishao |
| 25 | 14.55 | pinoresinol diglucoside | C32H42O16 | 681.2405 | M-H | 0.7 | 153994.40 | Ciwujia |
| 26 | 15.09 | Coniferin | C16H22O8 | 341.1240 | M-H | -0.5 | 24247.12 | Ciwujia |
| 27 | 15.34 | Ferulic acid | C10H10O4 | 193.0499 | M-H | -4.0 | 9475.23 | Ciwujia |
| 28 | 15.39 | Methylgallate | C8H8O5 | 183.0291 | M-H | -4.6 | 3839.92 | Baishao |
| 29 | 15.41 | isofraxidin | C11H10O5 | 221.0450 | M-H | -2.4 | 694716.74 | Ciwujia |
| 30 | 15.63 | Eriodictyol-7-glucoside | C21H22O11 | 449.1094 | M-H | 1.1 | 47158.56 | Shanzha |
| 31 | 16.00 | Eleutheroside E | C34H46O18 | 741.2613 | M-H | 0.3 | 251991.93 | Ciwujia |
| 32 | 16.49 | Kaempferol 3,7-diglucoside | C27H30O16 | 611.1591 | M+H | -2.5 | 97902.65 | Baishao |
| 33 | 17.10 | salvianolic acid j | C27H22O12 | 537.1042 | M-H | 0.7 | 570897.63 | Danshen |
| 34 | 17.36 | Zedoalactone A | C15H22O4 | 267.1582 | M+H | -3.2 | 453213.19 | Yujin |
| 35 | 17.86 | Astragalin | C21H20O11 | 447.0935 | M-H | 0.5 | 12830.03 | Baishao |
| 36 | 17.93 | hyperoside | C21H20O12 | 463.0878 | M-H | -0.8 | 12215384.18 | Ciwujia |
| 37 | 17.99 | Hyperin | C21H19O12- | 462.0795 | M-H | -1.9 | 1150.72 | Shanzha |
| 38 | 18.06 | galloylpaeoniflorin | C30H32O15 | 631.1667 | M-H | -0.2 | 16058243.72 | Baishao |
| 39 | 18.13 | syringaresinol | C22H26O8 | 417.1551 | M-H | -0.9 | 140594.29 | Ciwujia |
| 40 | 18.24 | Rutin | C27H30O16 | 609.1458 | M-H | -0.6 | 1038973.53 | Shanzha |
| 41 | 18.26 | paeoniflorin | C23H28O11 | 479.1557 | M-H | -0.5 | 28803979.43 | Baishao |
| 42 | 18.54 | salvianolic acid d | C20H18O10 | 417.0828 | M-H | 0.1 | 207922.08 | Danshen |
| 43 | 18.74 | coniferaldehyde glucoside | C16H20O8 | 339.1083 | M-H | -0.8 | 40943.18 | Ciwujia |
| 44 | 18.78 | luteolin-7-o-glucoside | C21H20O11 | 447.0931 | M-H | -0.4 | 237732.21 | Danshen |
| 45 | 18.79 | Zedoalactone B | C15H20O5 | 303.1216 | M+Na | 4.7 | 693524.35 | Yujin |
| 46 | 18.90 | przewaquinone f | C18H16O5 | 311.0924 | M-H | -0.4 | 5133.19 | Danshen |
| 47 | 18.90 | ciwujiatone | C22H26O9 | 435.1637 | M+H | -2.9 | 269948.94 | Ciwujia |
| 48 | 19.21 | (2R)-3-(3,4-dihydroxyphenyl)-2-[(Z)-3-(3,4-dihydroxyphenyl)acryloyl]oxy-propionic acid | C18H16O8 | 359.0774 | M-H | 0.5 | 7058389.30 | Danshen |
| 49 | 19.24 | Cinarina | C25H24O12 | 515.1188 | M-H | -1.4 | 342062.42 | Ciwujia |
| 50 | 19.54 | Zedoarolide B | C15H22O5 | 281.1394 | M-H | -0.2 | 12467.13 | Yujin |

Supplementary Table 2 (*continued*)

| No. | RT (min) | Name | Formula | m/z | Adducts | Error  (ppm) | Instensity | Origin |
| --- | --- | --- | --- | --- | --- | --- | --- | --- |
| 51 | 19.97 | luteolin | C15H10O6 | 285.0405 | M-H | 0.0 | 11270.55 | Danshen |
| 52 | 20.22 | salvianolic acid g | C18H12O7 | 341.0647 | M+H | -2.7 | 71758.97 | Danshen |
| 53 | 20.25 | salvianolic acid e | C36H30O16 | 717.1467 | M-H | 0.8 | 45402565.15 | Danshen |
| 54 | 20.26 | salvianolic acid b | C36H30O16 | 741.1406 | M+H | -2.8 | 460084.83 | Danshen |
| 55 | 20.29 | lithospermic acid | C27H22O12 | 537.1043 | M-H | 0.9 | 222429.38 | Danshen |
| 56 | 20.43 | isoimperatorin | C16H14O4 | 269.0819 | M-H | -0.1 | 13394.06 | Danshen |
| 57 | 20.73 | Melafolone | C22H24O7 | 401.1584 | M+H | -2.7 | 1029512.06 | Ciwujia |
| 58 | 20.94 | 1-(4-hydroxy-3-methoxyphenyl)-7-(4-hydroxyphenyl)heptane-3,5-dione | C20H22O5 | 341.1391 | M-H | -0.9 | 19643.32 | Yujin |
| 59 | 21.24 | (Z)-3,4-Dimethoxycinnamic acid | C11H12O4 | 207.0657 | M-H | -2.7 | 36034.04 | Shanzha |
| 60 | 21.47 | Monomethyl lithospermate | C28H24O12 | 553.1329 | M+H | -2.1 | 1857590.75 | Danshen |
| 61 | 21.71 | (3S,3aR,5S,6S,7aR)-5,6-dihydroxy-3,6-dimethyl-3,3a,4,5,7,7a-hexahydrobenzofuran-2-one | C10H16O4 | 199.0969 | M-H | -3.3 | 18322.04 | Baishao |
| 62 | 21.84 | salvianolic acid n | C26H22O10 | 493.1141 | M-H | 0.1 | 41950258.67 | Danshen |
| 63 | 21.92 | Pentagalloylglucose | C41H32O26 | 939.1111 | M-H | 0.2 | 8965710.49 | Baishao |
| 64 | 21.96 | apigenin | C15H10O5 | 269.0458 | M-H | 0.9 | 9503.67 | Danshen |
| 65 | 22.04 | Calebin-A | C21H20O7 | 383.1140 | M-H | 1.1 | 974.64 | Yujin |
| 66 | 22.14 | 9'-Methyl lithospermate B | C37H32O16 | 731.1623 | M-H | 0.7 | 804060.22 | Danshen |
| 67 | 22.29 | Quercetin | C15H10O7 | 301.0352 | M-H | -0.5 | 2076131.80 | Shanzha |
| 68 | 23.18 | isosalvianolic acid c | C26H20O10 | 493.1121 | M+H | -1.6 | 163668.65 | Danshen |
| 69 | 23.22 | benzoyl paeoniflorin | C30H32O12 | 583.1826 | M-H | 0.8 | 291215.74 | Baishao |
| 70 | 23.49 | Zedoarol | C15H18O3 | 245.1180 | M-H | -1.2 | 11080.20 | Yujin |
| 71 | 23.66 | Pinen-10-yl vicianoside | C21H34O10 | 445.2075 | M-H | -1.0 | 2688791.81 | Baishao |
| 72 | 23.96 | (Z)-(1S,5R)-β-Pinen-10-yl-β-vicianoside | C21H34O10 | 445.2075 | M-H | -1.0 | 1935271.98 | Baishao |
| 73 | 24.22 | turmeronol A | C15H20O2 | 233.1531 | M+H | -2.3 | 115502.76 | Yujin |
| 74 | 24.45 | sesamin | C20H18O6 | 353.1034 | M-H | 0.9 | 4208.95 | Ciwujia |
| 75 | 24.55 | (-)-Epicatechin | C15H14O6 | 289.0717 | M-H | -0.1 | 207520.34 | Shanzha |
| 76 | 25.48 | salvianolic acid c | C26H20O10 | 491.0984 | M-H | 0.1 | 1130008.25 | Danshen |
| 77 | 25.49 | Curcumenolactone C | C15H20O4 | 263.1288 | M-H | -0.3 | 19332.87 | Yujin |
| 78 | 26.34 | Neoprocurcumenol | C15H22O2 | 235.1688 | M+H | -1.8 | 2000880.48 | Yujin |
| 79 | 27.08 | Isoprocurcumenol | C15H22O2 | 235.1688 | M+H | -2.0 | 851528.29 | Yujin |
| 80 | 27.17 | neocryptotanshinone ii | C17H18O3 | 269.1183 | M-H | -0.1 | 1024.08 | Danshen |
| 81 | 27.47 | Gibberellin A21 | C19H22O7 | 361.1295 | M-H | 0.7 | 5510.50 | Baishao |
| 82 | 27.57 | 4-methylenemiltirone | C18H18O2 | 267.1375 | M+H | -1.8 | 1964.87 | Danshen |
| 83 | 29.00 | Procurcumenol | C15H22O2 | 235.1689 | M+H | -1.6 | 574876.18 | Yujin |
| 84 | 29.20 | Dihydrocurcumenone | C15H24O2 | 237.1844 | M+H | -2.3 | 139405.44 | Yujin |
| 85 | 29.20 | Germacron | C15H22O | 219.1740 | M+H | -1.7 | 904353.72 | Yujin |
| 86 | 29.31 | 1,5-Bis(4-hydroxy-3-methoxyphenyl)-1,4-pentadien-3-one | C19H18O5 | 349.1035 | M+H | -3.4 | 536273.89 | Yujin |
| 87 | 29.33 | miltionone I | C19H20O4 | 313.1427 | M+H | -2.2 | 24359.96 | Danshen |
| 88 | 29.71 | curcumin | C21H20O6 | 367.1182 | M-H | -1.5 | 58297.28 | Yujin |
| 89 | 29.89 | 1,2,5,6-tetrahydrotanshinone | C18H16O3 | 279.1025 | M-H | -0.8 | 500.82 | Danshen |
| 90 | 29.94 | Furanogermenone | C15H20O2 | 233.1531 | M+H | -2.3 | 35383.54 | Yujin |
| 91 | 30.20 | neocryptotanshinone | C19H22O4 | 313.1444 | M-H | -0.5 | 2990089.99 | Danshen |
| 92 | 30.29 | Cadalenequinone | C15H16O2 | 229.1218 | M+H | -2.3 | 1228971.70 | Yujin |
| 93 | 30.67 | przewaquinone c | C18H16O4 | 297.1115 | M+H | -2.2 | 52255.56 | Danshen |
| 94 | 30.94 | Octahydrocurcumin | C21H28O6 | 399.1774 | M+Na | -1.2 | 9075.27 | Yujin |
| 95 | 30.99 | 1,2-Dihydrotanshinquinone | C18H14O3 | 279.1010 | M+H | -2.0 | 2008669.31 | Danshen |
| 96 | 31.26 | ar-turmerone | C15H20O | 217.1583 | M+H | -1.7 | 779529.74 | Yujin |
| 97 | 32.18 | 7beta-Hydroxy-8,13-abietadiene-11,12-dione | C20H28O3 | 315.1965 | M-H | -0.4 | 24119.55 | Danshen |
| 98 | 32.23 | przewalskin | C18H24O2 | 271.1703 | M-H | -0.3 | 9960.33 | Danshen |
| 99 | 32.27 | 13-Hydroxygermacrone | C15H22O2 | 233.1542 | M-H | -2.0 | 249023.60 | Yujin |
| 100 | 32.44 | microstegiol | C20H26O2 | 297.1857 | M-H | -1.0 | 50366.68 | Danshen |

Supplementary Table 3 (*continued*)

| No. | RT (min) | Name | Formula | m/z | Adducts | Error  (ppm) | Instensity | Origin |
| --- | --- | --- | --- | --- | --- | --- | --- | --- |
| 101 | 32.53 | tigogenin | C27H44O3 | 439.3196 | M+Na | 3.2 | 1356747.27 | Danshen |
| 102 | 32.71 | salviolone | C18H20O2 | 269.1532 | M+H | -1.6 | 435.63 | Danshen |
| 103 | 32.84 | Palbinone | C22H30O4 | 357.2070 | M-H | -0.3 | 53863.09 | Baishao |
| 104 | 32.89 | Euscaphic acid | C30H48O5 | 487.3424 | M-H | -1.1 | 6868167.51 | Danshen |
| 105 | 33.18 | dihydrotanshinone I | C18H14O3 | 277.0868 | M-H | -0.8 | 221.83 | Danshen |
| 106 | 33.20 | tanshinone i | C18H12O3 | 277.0853 | M+H | -2.3 | 303503.45 | Danshen |
| 107 | 33.69 | deoxyneocryptotanshinone | C19H22O3 | 297.1493 | M-H | -1.1 | 5788.83 | Danshen |
| 108 | 33.71 | cryptotanshinone | C19H20O3 | 297.1478 | M+H | -2.5 | 1027044.89 | Danshen |
| 109 | 33.75 | Isotanshinone IIA | C19H18O3 | 295.1323 | M+H | -2.0 | 5625.51 | Danshen |
| 110 | 33.88 | Przewaquinone A | C19H18O4 | 311.1272 | M+H | -1.8 | 1833.25 | Danshen |
| 111 | 33.97 | Glucosol | C30H48O4 | 473.3628 | M+H | 0.6 | 30157.54 | Danshen |
| 112 | 34.25 | sugiol | C20H28O2 | 299.2014 | M-H | -0.7 | 196976.21 | Danshen |
| 113 | 34.56 | Chiisanogenin | C30H44O5 | 483.3112 | M-H | -0.9 | 2489325.63 | Ciwujia |
| 114 | 35.06 | Maslinic acid | C30H48O4 | 471.3476 | M-H | -0.9 | 328116.78 | Shanzha |
| 115 | 35.43 | Officinalic acid | C30H44O6 | 499.3064 | M-H | -0.1 | 65657.76 | Shanzha |
| 116 | 35.70 | 3beta-Hydroxyoleana-11,13(18)-dien-28-oic acid | C30H46O3 | 455.3512 | M+H | -1.8 | 752829.20 | Baishao |
| 117 | 35.70 | tanshinone iia | C19H18O3 | 295.1321 | M+H | -2.6 | 79455.74 | Danshen |
| 118 | 36.15 | Linolenic acid | C18H30O2 | 279.2312 | M+H | -2.3 | 262770.31 | Shanzha |
| 119 | 36.77 | Hederagenol | C30H48O4 | 471.3476 | M-H | -0.9 | 1147721.66 | Baishao |
| 120 | 37.13 | 3-epicorosolic acid | C30H48O4 | 473.3619 | M+H | -1.4 | 761715.42 | Danshen |
| 121 | 37.57 | Fomefficinic Acid E | C33H48O5 | 525.3549 | M+H | -4.9 | 28322.85 | Shanzha |
| 122 | 37.77 | (4aS,6aR,6aS,6bR,8aR,10S,12aR,14bS)-2,2,6a,6b,9,9,12a-heptamethyl-10-[(2S,3R,4S,5S)-3,4,5-trihydroxyoxan-2-yl]oxy-1,3,4,5,6,6a,7,8,8a,10,11,12,13,14b-tetradecahydropicene-4a-carboxylic acid | C35H56O7 | 611.3906 | M+Na | -2.0 | 235626.18 | Ciwujia |
| 123 | 38.06 | Ursolic acid | C30H48O3 | 457.3669 | M+H | -1.6 | 887986.48 | Shanzha |
| 124 | 38.34 | Fomefficinic acid C | C31H50O4 | 487.3768 | M+H | -2.9 | 37337.49 | Shanzha |
| 125 | 38.56 | uvaol | C30H50O2 | 443.3877 | M+H | -1.4 | 71056.56 | Danshen |
| 126 | 39.37 | Betulinic acid | C30H48O3 | 455.3526 | M-H | -1.1 | 2603307.25 | Ciwujia |
| 127 | 41.50 | 3-O-Acetyloleanolic acid | C32H50O4 | 521.3594 | M+H | -1.4 | 180174.32 | Danshen |
| 128 | 43.11 | daucosterol | C35H60O6 | 599.4275 | M+Na | -1.2 | 1038057.83 | Danshen |
| 129 | 44.85 | Lanosterol | C30H50O | 449.3742 | M+H | -2.7 | 1456718.40 | Shanzha |

Supplementary Table 4 Quantitative analysis of the primary compounds in XSB

| No. | Compound | Content (mg/g) | Origin |
| --- | --- | --- | --- |
| 1 | Paeoniflorin | 49.2523 | Baishao |
| 2 | Albiflorin | 25.1392 | Baishao |
| 3 | Benzoylpaeoniflorin | 1.3827 | Baishao |
| 4 | Eleutheroside B | 0.2348 | Ciwujia |
| 5 | Eleutheroside E | 0.5043 | Ciwujia |
| 6 | Salvianic acid A | 2.4912 | Danshen |
| 7 | Salvianolic acid A | 1.2937 | Danshen |
| 8 | Salvianolic acid B | 5.8455 | Danshen |
| 9 | Tanshinone I | 0.0106 | Danshen |
| 10 | Cryptotanshinone | 0.0020 | Danshen |
| 11 | Oleanolic acid | 1.0515 | Shanzha |
| 12 | Hyperoside | 0.2239 | Shanzha |
| 13 | Chlorogenic acid | 0.8334 | Shanzha |
| 14 | Rutinum | 0.0418 | Shanzha |
