# Supplementary material for: Multi-omics reveal the neuroprotective mechanisms of Xinshubao tablet against scopolamine-induced cognitive dysfunction in mice
Source: Front Pharmacol. 2025 Jul 4;16:1596728. doi: 10.3389/fphar.2025.1596728 (PMC12271746; doi:10.3389/fphar.2025.1596728)
Supplement: Supplementary file 9 [file Supplementaryfile2.docx]

**Gut microbiota 16S rDNA amplicon sequencing analysis**

Fecal samples from six mice per group were aseptically collected and promptly stored at -80°C for subsequent analysis. Total genomic DNA was extracted using a commercial DNA isolation kit following the manufacturer’s protocol. The concentration and purity of the extracted DNA were assessed using a NanoDrop spectrophotometer. The V3-V4 hypervariable regions of the bacterial 16S rDNA were amplified by PCR using specific primers with barcodes. The PCR products were purified using Agencourt AMPure XP beads and quantified with a Qubit fluorometer.

Purified amplicons were pooled in equimolar concentrations and subjected to paired-end sequencing (2 × 250 bp) on the Illumina NovaSeq platform. Raw sequencing data were processed using QIIME2, including demultiplexing, quality filtering, and the removal of chimeric sequences. Amplicon sequence variants (ASVs) were identified through exact sequence variants, and taxonomic classification was performed against the SILVA 16S rDNA gene reference database. Alpha diversity indices, such as Chao1, Shannon, and Simpson, were calculated to assess species richness and diversity. Beta diversity was evaluated using weighted UniFrac distances. Differentially abundant taxa were identified using linear discriminant analysis effect size (LEfSe), with an LDA score threshold of 3.0. Statistical significance was determined using the Kruskal-Wallis test, with a threshold of *P<* 0.05 for all comparisons. Spearman correlation analysis was performed to investigate the associations between differentially abundant taxa and cognitive function-related indicators, including escape latency, platform crossing number, time spent in the target quadrant, and spontaneous alternation. A correlation coefficient threshold of |R| > 0.3 and a significance level of *P<* 0.05 were used to identify potential associations.

**Serum metabolomics analysis**

Serum samples from six mice per group were collected under fasting conditions and stored at -80°C until further analysis. Metabolites were extracted from serum using a methanol/chloroform (1:1, v/v) mixture, with internal standards incorporated to ensure analytical consistency. Following extraction, samples were vortexed, centrifuged, and the supernatant dried under nitrogen. The resulting metabolites were reconstituted in an appropriate solvent for analysis. Metabolomics profiling was conducted using liquid chromatography coupled with mass spectrometry (LC-MS). Chromatographic separation was performed on an ACQUITY UPLC system, with mass spectrometric detection carried out on a Q Exactive Plus Orbitrap mass spectrometer. Data acquisition was conducted in both positive and negative ion modes, and full-scan mass spectra were recorded across a specified m/z range. Data were processed using XCMS (https://xcmsonline.scripps.edu) and MetaboAnalyst (https://www.metaboanalyst.ca) for peak identification, alignment, and normalization. Multivariate statistical analyses, including principal component analysis (PCA) and partial least squares discriminant analysis (PLS-DA), were performed to identify distinct metabolic signatures among groups. Differential metabolites (DMs) were determined based on the variable importance in projection (VIP) scores from the PLS-DA model, with a threshold of VIP > 1.0. Metabolites were annotated by matching to the HMDB (https://hmdb.ca) and KEGG (https://www.kegg.jp) databases. Pathway enrichment analysis was performed using KEGG to identify the potential metabolic networks influenced by the experimental treatment. Pearson correlation analysis was conducted to explore the relationships between DMs and cognitive function-related indicators, including escape latency, platform crossing number, time spent in the target quadrant, and spontaneous alternation. A correlation coefficient threshold of |R| > 0.5 and statistical significance of *P<* 0.05 were used to identify significant associations.

**Hippocampal transcriptomic analysis**

mRNA-seq was conducted to analyze transcriptomic variations in the hippocampal tissues, following previously published methodologies. Total RNA was extracted using a commercial RNA extraction kit (RC101-01, Vazyme Biotech Co., Ltd, Nanjing, China). RNA quality was assessed via spectrophotometry (NanoDrop) and electrophoresis (Agilent 2100 Bioanalyzer), ensuring a RIN value greater than 7.0. Library preparation was performed using the NEBNext® Ultra™ II RNA Library Prep Kit (New England BioLabs, USA), and sequencing was carried out on the Illumina NovaSeq 6000 platform. Raw sequencing reads were filtered and trimmed using Trimmomatic, and clean reads were mapped to the reference genome via HISAT2. Gene expression levels were quantified by calculating the TPM value. Differentially expressed genes (DEGs) were identified using DESeq2 with an absolute log_2_ fold change (log_2_ FC) ≥ 1 and *P* value < 0.05. Functional annotation of DEGs was conducted through Gene Ontology (GO) and KEGG pathway enrichment using DAVID database (https://davidbioinformatics.nih.gov/tools.jsp). The Volcano, Heatmap, GO and KEGG pathway enrichment plots were generated using the online tool available at https://www.bioinformatics.com.cn.
